# Siloxide tripodal ligands as a scaffold for stabilizing lanthanides in the +4 oxidation state†

**DOI:** 10.1039/d4sc00051j

**Published:** 2024-04-02

**Authors:** Maxime Tricoire, Fang-Che Hsueh, Megan Keener, Thayalan Rajeshkumar, Rosario Scopelliti, Ivica Zivkovic, Laurent Maron, Marinella Mazzanti

**Affiliations:** a Group of Coordiantion Chemistry, Institut des Sciences et Ingénierie Chimiques, École Polytechnique Fédérale de Lausanne (EPFL) 1015 Lausanne Switzerland marinella.mazzanti@epfl.ch; b Laboratoire de Physique et Chimie des Nano-objets, Institut National des Sciences Appliquées Cedex 4 31077 Toulouse France laurent.maron@irsamc.ups-tlse.fr; c Institut des Sciences et Ingénierie Chimiques, École Polytechnique Fédérale de Lausanne (EPFL) 1015 Lausanne Switzerland; d Laboratory for Quantum Magnetism, Institute of Physics, Ecole Polytechnique Fédérale de Lausanne (EPFL) CH-1015 Lausanne Switzerland

## Abstract

Synthetic strategies to isolate molecular complexes of lanthanides, other than cerium, in the +4 oxidation state remain elusive, with only four complexes of Tb(iv) isolated so far. Herein, we present a new approach for the stabilization of Tb(iv) using a siloxide tripodal trianionic ligand, which allows the control of unwanted ligand rearrangements, while tuning the Ln(iii)/Ln(iv) redox-couple. The Ln(iii) complexes, [Ln^III^((OSiPh_2_Ar)_3_-arene)(THF)_3_] (1-Ln^Ph^) and [K(toluene){Ln^III^((OSiPh_2_Ar)_3_-arene)(OSiPh_3_)}] (2-Ln^Ph^) (Ln = Ce, Tb, Pr), of the (HOSiPh_2_Ar)_3_-arene ligand were prepared. The redox properties of these complexes were compared to those of the Ln(iii) analogue complexes, [Ln^III^((OSi(O^*t*^Bu)_2_Ar)_3_-arene)(THF)] (1-Ln^O^*^t^*^Bu^) and [K(THF)_6_][Ln^III^((OSi(O^*t*^Bu)_2_Ar)_3_-arene)(OSiPh_3_)] (2-Ln^O^*^t^*^Bu^) (Ln = Ce, Tb), of the less electron-donating siloxide trianionic ligand, (HOSi(O^*t*^Bu)_2_Ar)_3_-arene. The cyclic voltammetry studies showed a cathodic shift in the oxidation potential for the cerium and terbium complexes of the more electron-donating phenyl substituted scaffold (1-Ln^Ph^) compared to those of the *tert*-butoxy (1-Ln^O^*^t^*^Bu^) ligand. Furthermore, the addition of the –OSiPh_3_ ligand further shifts the potential cathodically, making the Ln(iv) ion even more accessible. Notably, the Ce(iv) complexes, [Ce^IV^((OSi(O^*t*^Bu)_2_Ar)_3_-arene)(OSiPh_3_)] (3-Ce^O^*^t^*^Bu^) and [Ce^IV^((OSiPh_2_Ar)_3_-arene)(OSiPh_3_)(THF)_2_] (3-Ce^Ph^), were prepared by chemical oxidation of the Ce(iii) analogues. Chemical oxidation of the Tb(iii) and Pr(iii) complexes (2-Ln^Ph^) was also possible, in which the Tb(iv) complex, [Tb^IV^((OSiPh_2_Ar)_3_-arene)(OSiPh_3_)(MeCN)_2_] (3-Tb^Ph^), was isolated and crystallographically characterized, yielding the first example of a Tb(iv) supported by a polydentate ligand. The versatility and robustness of these siloxide arene-anchored platforms will allow further development in the isolation of more oxidizing Ln(iv) ions, widening the breadth of high-valent Ln chemistry.

## Abstract 2

Robust arene-anchored polydentate siloxide ligands allow to control unwanted ligand rearrangements for the isolation of Tb(iv) complexes thus enabling the use of the Tb(iii)/Tb(iv) couple for the separation of Tb from the neighboring Dy ion.

## Graphical abstract

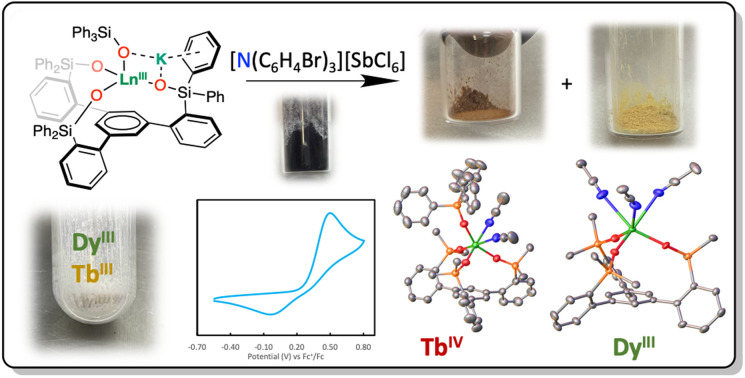

## Introduction

**Scheme 1:**
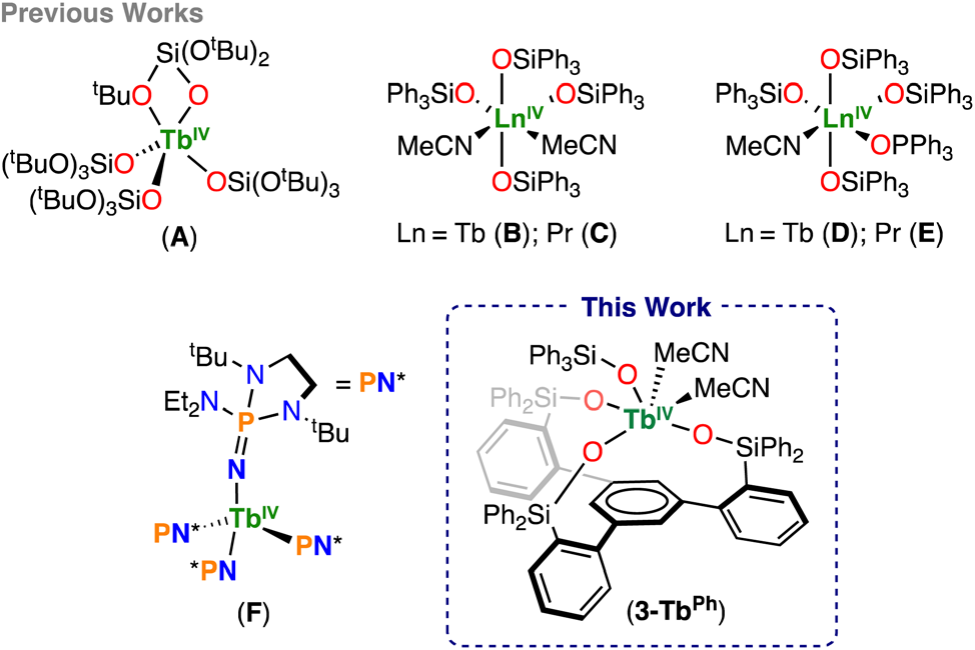
Structurally characterized Tb(iv) and Pr(iv) molecular complexes.

Until 2019, molecular complexes containing lanthanides in the +4 oxidation state have been limited to cerium (Ce),^3–8^ for which the readily accessible Ce(iii)/Ce(iv) couple has resulted in a broad range of applications in organic synthesis^9^ and in catalysis.^10–17^ In contrast, although the +4 oxidation state had been identified for Pr, Nd, Tb, and Dy in solid state compounds,^18–24^ and in the gas phase (*via* mass spectrometry and/or vibrational spectroscopy in matrix isolation) for Pr, Nd, Tb, and Dy,^25–27^ where even Pr(v) was observed,^28–30^ the high Ln(iii)/Ln(iv) oxidation potential of these ions prevented their isolation in molecular complexes. The electrochemical oxidation of Tb(iii) and Pr(iii) to Tb(iv) and Pr(iv) in concentrated aqueous carbonate solutions was reported in the 1980s,^31^ but seminal attempts to isolate molecular complexes of Tb(iv) and Pr(iv) using chemical oxidation in organic solutions were unsuccessful.^32–34^ In 2019, the first molecular complexes of Tb(iv) were isolated using the bulky monoanionic σ and π donor supporting ligands tris(*tert*-butoxy)siloxide (–OSi(O^*t*^Bu)_3_)^35^ and tris(amidyl)imidophosphorane ([NP(pip)_3_], pip = piperidinyl) (Scheme 1).^36^ Despite the very small difference in oxidation potentials between Tb(iii) and Pr(iii) (0.1 V),^19^ attempts to prepare complexes of Pr(iv) in the solid state using the same ligands^37,38^ resulted in the formation of highly reactive Pr(iv) species.

These species were thoroughly characterized in solution for the tris(amidyl)imidophosphorane system,^38^ but could not be isolated in the solid state. Ln(iii) complexes of the triphenylsiloxide (–OSiPh_3_) ligand had been reported 30 years ago,^39,40^ but only in 2020, our group demonstrated that such monodentate siloxide ligands can stabilize both Tb and Pr ions in the +4 oxidation state.^37,41^ The isostructural complexes, [Ln^IV^(OSiPh_3_)_4_(MeCN)_2_]^37,41^ were isolated by the productive chemical oxidation of the analogous Ln(iii) complexes and showed reasonable solution stability, which could be improved by the addition of the neutral triphenylphosphine oxide ligand (Ph_3_PO)^42^ or more recently by the addition of the neutral bidentate ligands, 2,2′-bipyridine (bpy), 2,2′-bipyrimidine (bpym), and 1,10-phenanthroline (phen).^43^ The higher stability of the –OSiPh_3_ complexes compared to the –OSi(O^*t*^Bu)_3_ complexes was reflected in their lower oxidation potentials, which was attributed to the stronger electron-donating ability of the –OSiPh_3_ ligands. However, preliminary attempts to prepare Ln(iv) complexes with more electron-donating, monoanionic supporting ligands have so far been unsuccessful.

Surprisingly, since 2020, no other anionic supporting ligands capable of stabilizing Tb(iv) or Pr(iv) have been identified, demonstrating the difficulty in identifying ligands and conditions that are capable of stabilizing lanthanides, other than Ce, in the +4 oxidation state.

Tripodal trianionic ligand frameworks are ideal for effectively preventing unwanted ligand rearrangements that can pose a significant synthetic challenge in the isolation of Ln^IV^ coordination complexes through oxidative routes.^44–48^ Moreover, Schelter and co-workers showed that tripodal ligands are effective for gaining better control of the Ln(iii)/Ln(iv) redox-couple by providing a single coordination site.^45^ Schelter and coworkers also demonstrated that tripodal ligands allow the implementation of simple solubility-based separation of early and late f-elements.^49,50^

In contrast, despite their potential relevance for the implementation of redox based separation of f-elements, synthetic routes for incorporating Ln(iv), other than cerium, in tripodal-based ligands remain unidentified.

Recently, we reported a new tripodal ligand, (HOSi(O^*t*^Bu)_2_Ar)_3_-arene, and its corresponding Ce complexes, demonstrating that four different redox states were accessible with this ligand scaffold.^48^

Herein, we show that by combining a more electron-donating tripodal ligand, (HOSiPh_2_Ar)_3_-arene,^1,2^ with the monodentate –OSiPh_3_ ligand, the redox potential can be tuned to access Ln(iv), enabling the isolation of a new Tb(iv) complex containing a trianionic polydentate ligand.

## Results and discussion

### Synthesis of cerium(iii), terbium(iii), and praseodymium(iii) complexes

Recently, we reported the synthesis of the neutral tris(di-*tert*-butoxy)siloxide arene Ce(iii) complex, [Ce^III^((OSi(O^*t*^Bu)_2_Ar)_3_-arene)(THF)] (1-Ce^O^*^t^*^Bu^).^48^ Utilizing a similar approach, we first synthesized the analogous Tb(iii) complex, [Tb^III^((OSi(O^*t*^Bu)_2_Ar)_3_-arene)(THF)] (1-Tb^O^*^t^*^Bu^), by the same protonolysis route. Addition of 1.0 equiv. [Tb^III^(N(SiMe_3_)_2_)_3_]^51,52^ to 1.0 equiv. of the (HOSi(O^*t*^Bu)_2_Ar)_3_-arene ligand in THF at room temperature resulted in a colorless solution. Analysis of the reaction mixture by ^1^H NMR spectroscopy indicated the disappearance of the Tb(iii) precursor, [Tb^III^(N(SiMe_3_)_2_)_3_], and formation of a new species (Fig. S16†). X-ray quality crystals of complex 1-Tb^O^*^t^*^Bu^ were obtained from a concentrated toluene solution at −40 °C in 66% yield.

Due to its high oxidation potential (*E*_pa_) (*vide infra*) direct oxidation of the tris(di-*tert*-butoxy)siloxide arene Ce(iii) complex 1-Ce^O^*^t^*^Bu^ was not possible, and the Ce(iv) analogue, 1-Ce^O^*^t^*^Bu^Cl, could only be prepared from the Ce(iv) precursor [Ce^IV^Cl(N(SiMe_3_)_2_)_3_].

Instead, we postulated that direct Ce(iii) to Ce(iv) oxidation may become possible by coordination of an additional –OSiPh_3_ ligand to 1-Ce^O^*^t^*^Bu^, and/or by incorporation of phenyl substituents on the tripodal backbone, which would shift the potential cathodically.^3,4^ This approach could then be utilized to access Tb(iv) and Pr(iv) species.

Therefore, we next investigated the synthesis of the phenyl-substituted Ln(iii) (Ln = Ce, Tb, Pr) tripodal complexes.

The addition of 1.0 equiv. of [Ln^III^(N(SiMe_3_)_2_)_3_] (Ln = Ce, Tb, Pr) to the previously reported (HOSiPh_2_Ar)_3_-arene ligand^1,2^ in THF at room temperature yielded the Ln(iii) complexes, [Ce^III^((OSiPh_2_Ar)_3_-arene)(THF)_3_] (1-Ce^Ph^), [Tb^III^((OSiPh_2_Ar)_3_-arene)(THF)_3_] (1-Tb^Ph^), and [Pr^III^((OSiPh_2_Ar)_3_-arene)(THF)_3_] (1-Pr^Ph^) in 82%, 82%, and 73% yields, respectively. The synthesis *via* salt metathesis of complexes 1-Ce^Ph^, 1-Tb^Ph^, and 1-Pr^Ph^ was reported after the first submission of this manuscript, and was performed by reacting CeI_3_(THF)_4_, TbCl_3_, and PrI_3_(THF)_4_ with the ligand salt.^53^ Single crystals suitable for XRD analysis of complexes 1-Ln^Ph^ (Ln = Ce, Tb, Pr) were obtained from concentrated toluene solutions at −40 °C.

Comparison of the oxidation potentials for complexes 1-Ce^O^*^t^*^Bu^ and 1-Ce^Ph^ (*vide infra*) displays a significant cathodic shift (Δ*E*_pa_ = 0.87 V), suggesting that 1-Ce^Ph^ is significantly more electron-rich than 1-Ce^O^*^t^*^Bu^. For complexes 1-Tb^Ph^ and 1-Pr^Ph^, no oxidation events were observed in the window permitted by THF and the [NBu_4_][B(C_6_F_5_)_4_] electrolyte. Based on these electrochemical results, we next postulated that coordination of an additional monoanionic siloxide ligand could further shift the oxidation potential cathodically, allowing access to the desired high-valent Ln(iv) species.

At first, we explored the addition of a –OSiPh_3_ ligand to the complexes 1-Ce^O^*^t^*^Bu^ and 1-Tb^O^*^t^*^Bu^. The addition of 1.0 equiv. of KOSiPh_3_ to the complexes 1-Ln^O^*^t^*^Bu^ (Ln = Ce, Tb) in THF led to the formation of new species and full consumption of the starting materials as indicated by ^1^H NMR spectroscopy (Fig. S3 and S20†). Crystals suitable for XRD analysis of complexes [K(THF)_6_][Ce^III^((OSi(O^*t*^Bu)_2_Ar)_3_-arene)(OSiPh_3_)] (2-Ce^O^*^t^*^Bu^) and [K(THF)_6_][Tb^III^((OSi(O^*t*^Bu)_2_Ar)_3_-arene)(OSiPh_3_)] (2-Tb^O^*^t^*^Bu^) were isolated from concentrated THF solutions in 90% and 82% yields, respectively.

As anticipated, the addition of the –OSiPh_3_ ligand led to a significant shift in the oxidation potential (Δ*E*_pa_ = 1.57 V) of the Ce(iii) complexes 1-Ce^O^*^t^*^Bu^ and 2-Ce^O^*^t^*^Bu^ suggesting that a further shift could be expected by the addition of KOSiPh_3_ to the 1-Ln^Ph^ (Ln = Ce, Tb, Pr) complexes.

The complexes [K(toluene){Ln^III^((OSiPh_2_Ar)_3_-arene)(OSiPh_3_)}], 2-Ln^Ph^ (Ln = Ce, Tb, Pr), were obtained by the same approach. Addition of 1.0 equiv. of KOSiPh_3_ to 1.0 equiv. of 1-Ln^Ph^ (Ln = Ce, Tb, Pr) in THF solutions led to the isolation of complexes 2-Ce^Ph^, 2-Tb^Ph^ and 2-Pr^Ph^ in 86%, 67%, and 86% yields, respectively. Interestingly, we found that addition of 1.0 equiv. of 2.2.2-cryptand to 2-Ln^Ph^ (Ln = Ce, Tb, Pr) in THF and MeCN solutions, respectively, led to new resonances in the ^1^H NMR spectra, suggesting that the K^+^ cation is bound in solution (Fig. S7, S24 and S34†).

### Solid state structure and electronic structure of Ln(iii) complexes

**Fig. 1:**
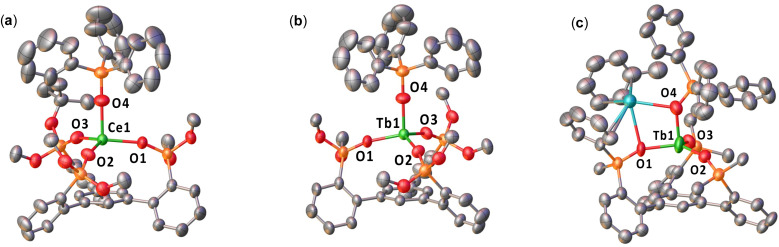
Molecular structures of (a) the anion [Ce^III^((OSi(O^*t*^Bu)_2_Ar)_3_-arene)(OSiPh_3_)]^−^ in (2-Ce^O^*^t^*^Bu^), (b) the anion [Tb^III^((OSi(O^*t*^Bu)_2_Ar)_3_-arene)(OSiPh_3_)]^−^ in (2-Tb^O^*^t^*^Bu^), and (c) [K(toluene){Tb^III^((OSiPh_2_Ar)_3_-arene)(OSiPh_3_)}] (2-Tb^Ph^), with thermal ellipsoids drawn at the 50% probability level. Hydrogen atoms, methyl groups on the –OSi(O^*t*^Bu)_2_, some disordered substituents and the five carbon atoms of each phenyl have been omitted for clarity.

The solid-state molecular structures of complexes 1-Ce^Ph^·2(toluene), 2-Ce^OtBu^·0.9(THF), 1-Tb^O^*^t^*^Bu^, 1-Tb^Ph^·2(toluene), 2-Tb^OtBu^·0.9(THF), 2-Tb^Ph^·0.5(toluene), 2-Pr^Ph^-MeCN·4(MeCN), and 2-Tb^Ph^-MeCN·(Et_2_O) were determined by X-ray diffraction studies (Fig. 1, S47–S54†).

The solid-state molecular structure of complex 1-Ce^Ph^ (Fig. S47†) shows the presence of a neutral [Ce^III^(OSiPh_2_Ar)_3_-arene)(THF)_3_] complex, in which the Ce(iii) ion is bound by three –OSiPh_2_ oxygens of the tripodal ligand. Additionally, there are three coordinated THF molecules in 1-Ce^Ph^, while only one was found in [Ce^III^((OSi(O^*t*^Bu)_2_Ar)_3_-arene)(THF)], 1-Ce^O^*^t^*^Bu^ suggesting that the phenyl substituted ligand is significantly less bulky than the *tert*-butoxy ligand. The average Ce–O_siloxide_ distances (2.2187(7) Å) are comparable to those reported for the Ce(iii) complex, [Ce(OSiPh_3_)_3_(THF)_3_] (2.222(4) Å).^54^ The Ce–C_centroid_ distance (3.9234(9) Å) is significantly longer than that found in the tripodal (di-*tert*-butoxy)siloxide analogue, [Ce^III^((OSi(O^*t*^Bu)_2_Ar)_3_-arene)(THF)] (1-Ce^O^*^t^*^Bu^); (2.730(1) Å), indicating the absence of any cerium–arene interaction.

This was confirmed by DFT calculations (B3PW91 functional) including dispersion corrections (see the ESI† for computational details). The optimized geometry of 1-Ce^Ph^ compares well within the experimental data (Table S4†) with an average Ce–O_siloxide_ bond distance of 2.2187(7). The SOMO is a pure 4f orbital in line with a Ce(iii) complex. The Ce–C_centroid_ is 4.01 Å and is in good agreement with the experimental data. The Ce–C_centroid_ in 1-Ce^Ph^ is longer than that in 1-Ce^O^*^t^*^Bu^ (2.72 Å) and is consistent with a lack of a Ce–arene δ bonding interaction. Indeed, computational studies show that the LUMO is a fully delocalized π* without any Ce contribution.

Complex 2-Ce^O^*^t^*^Bu^ displays a mononuclear structure where the Ce(iii) ion is bound by three –OSiPh_2_ oxygens of the tripodal ligand, and one –OSiPh_3_ ligand in a pseudo-tetrahedron geometry (Fig. 1). The structure is completed by an outer-sphere [K(THF)_6_]^+^ cation. The Ce–O_siloxide_ distances (2.216(8)–2.234(4) Å) are comparable to those reported for the precursor, [Ce^III^((OSi(O^*t*^Bu)_2_Ar)_3_-arene)(THF)] (1-Ce^O^*^t^*^Bu^; (2.237(3) Å), but shorter than that of the Ce(iii) complex, [Ce^III^(OSi(O^*t*^Bu)_3_)_3_(THF)_2_] (2.308(3) Å).^55^ The Ce–O_OSiPh_3__ distance (2.216(8) Å) is similar to those found in the Ce(iii) complex supported by three triphenylsiloxide ligands, [Ce^III^(OSiPh_3_)_3_(THF)_3_] (2.222(4) Å).^54^ Notably, replacement of the axial THF molecule by a –OSiPh_3_ ligand in 2-Ce^O^*^t^*^Bu^ leads to a significantly longer Ce–C_centroid_ distance (3.284(5) Å) compared to that found in the precursor, 1-Ce^O^*^t^*^Bu^ (2.730(1) Å). This difference indicates the absence of any cerium–arene interaction.^48^

The solid-state molecular structure of complex 2-Ce^Ph^ could be determined by XRD studies; however, the crystal quality was not sufficient to define explicit metrical parameters. Complex 2-Ce^Ph^ displays a similar coordination geometry and ligand environment to 2-Ce^O^*^t^*^Bu^, but contains one inner-sphere K^+^ cation, which is bound by an oxygen from the tripodal ligand, the monodentate –OSiPh_3_ ligand, and a coordinated toluene molecule.

Complexes 1-Tb^O^*^t^*^Bu^, 1-Tb^Ph^, and 2-Tb^O^*^t^*^Bu^ (Fig. S48, S49† and 1) were found to be isostructural to the cerium analogs, 1-Ce^O^*^t^*^Bu^, 1-Ce^Ph^, and 2-Ce^O^*^t^*^Bu^, respectively. The average Tb–O_siloxide_ distances in 1-Tb^O^*^t^*^Bu^ (2.147(7) Å) and the Tb–C_centroid_ distance (average of the two molecules present in the asymmetric unit: 2.674(1) Å) are shorter than those found in 1-Ce^O^*^t^*^Bu^, indicative of a terbium–arene interaction.^48^ In 1-Tb^Ph^, the average Tb–O_siloxide_ distances (2.139(3) Å) and the Tb–C_centroid_ distance (3.972(1) Å) are shorter than those found in the Ce(iii) complexes, which can be attributed to the smaller ionic radii of the Tb(iii) ion. The Tb–O_siloxide_ distances are shorter in 2-Tb^O^*^t^*^Bu^ (2.115(8)–2.141(4) Å) compared to 1-Tb^O^*^t^*^Bu^, and fall within the range of the previously reported Tb(iii) complex, [KTb^III^(OSi(O^*t*^Bu)_3_)_4_(THF)_2_] (2.103(3)–2.152(2) Å).^35^ As observed in 2-Ce^O^*^t^*^Bu^, the Tb–C_centroid_ (3.516(6) Å) is elongated in 2-Tb^O^*^t^*^Bu^ due to the replacement of the axial THF with a –OSiPh_3_ ligand. Similarly, the Tb–O_siloxide_ distances in 2-Tb^Ph^ (2.128(6)–2.191(8) Å) and in the dimeric 2-Tb^Ph^-MeCN (2.163(2)–2.217(2) Å) are elongated compared to that in 1-Tb^Ph^, and are similar to those found in [KTb^III^(OSiPh_3_)_4_(THF)] (2.138(2)–2.194(2) Å),^41^ while the Tb–C_centroid_ distance (3.232(4) Å) in 2-Tb^Ph^ and 3.151(1) Å in 2-Tb^Ph^-MeCN) is reduced compared to that of the precursor 1-Tb^Ph^ (3.972(1) Å). The Pr–O_siloxide_ distances in 2-Pr^Ph^ (2.228(3)–2.286(3) Å) (Fig. S52†) were also found to be increased compared to that of 1-Pr^Ph^ (Fig. S51†) and the Pr–C_centroid_ distance (3.284(1) Å) is reduced.

Calculations were carried out on 2-Tb^Ph^, as a precursor of 3-Tb^Ph^, using the same computational method. The optimized geometry is in agreement with the experimental data (Table S10†), and the Tb–O_siloxide_ distances are reproduced with a maximum deviation of 0.05 Å. The Tb–O_siloxide_ WBI (Table S12†) are in the 0.40 range indicative of primarily ionic interactions, as expected for lanthanide complexes. The unpaired spin density is fully located at the Tb center in line with a Tb(iii) complex.

### Synthesis of the lanthanide(iv) complexes

With the tetrakis Ln(iii) complexes in hand, we next investigated accessing high-valent Ln(iv) species by use of chemical oxidants.

First, the oxidation of complexes 2-Ce^O^*^t^*^Bu^ and 2-Ce^Ph^ was investigated with the oxidant AgBPh_4_ (0.41 V *vs.* Fc^0^/Fc^+^ in THF).^56^

**Scheme 2:**
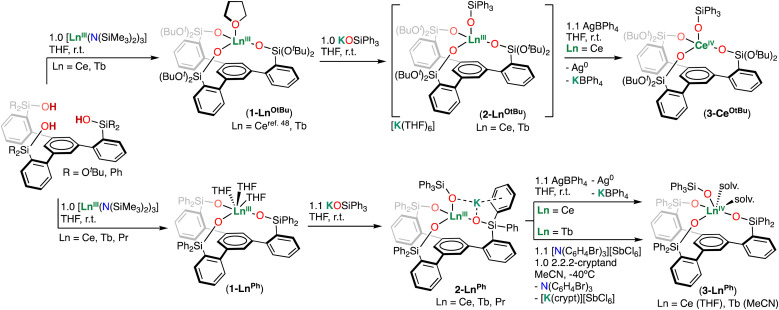
Synthesis of Ln(iii) and Ln(iv) complexes supported by trianionic tripodal ligands.

Addition of 1.1 equiv. of AgBPh_4_ to complexes 2-Ce^O^*^t^*^Bu^ and 2-Ce^Ph^ in THF at room temperature resulted in the isolation of the Ce(iv) complexes [Ce^IV^((OSi(O^*t*^Bu)_2_Ar)_3_-arene)(OSiPh_3_)] (3-Ce^O^*^t^*^Bu^) and [Ce^IV^ ((OSiPh_2_Ar)_3_-arene)(OSiPh_3_)(THF)_2_] (3-Ce^Ph^), in 81% and 85% yields, respectively (Scheme 2). In contrast to the paramagnetic Ce(iii) precursors, the resonances of complexes 3-Ce^O^*^t^*^Bu^ and 3-Ce^Ph^ appear within the diamagnetic region of the ^1^H NMR spectra (Fig. S9 and S13†), confirming that oxidation of the Ce(iii) center has occurred. Single crystals suitable for X-ray diffraction studies of 3-Ce^O^*^t^*^Bu^ and 3-Ce^Ph^ were grown from concentrated toluene and THF solutions at −40 °C, respectively.

Since a quasi-reversible redox event was observed for 2-Tb^Ph^, with an oxidation potential of *E*_pa_ = 0.53 V *vs.* Fc^0^/Fc^+^ (*vide infra*), the oxidation of complex 2-Tb^Ph^ was investigated with the commercially available oxidant [N(C_6_H_4_Br)_3_][SbCl_6_], commonly known as “magic blue” (0.70 V *vs.* Fc^0^/Fc^+^ in MeCN).^56^

The addition of 1.1 equiv. of [N(C_6_H_4_Br)_3_][SbCl_6_] to complex 2-Tb^Ph^ and 2.2.2-cryptand in MeCN at −40 °C resulted in the immediate precipitation of an orange powder identified as the Tb(iv) complex, [Tb^IV^((OSiPh_2_Ar)_3_-arene)(OSiPh_3_)(MeCN)_2_] (3-Tb^Ph^) in 53% yield. Analysis of the colorless supernatant by ^1^H NMR spectroscopy indicated the formation of the diamagnetic by-product of oxidation, N(C_6_H_4_Br)_3_, including some trace unknown species.

Dark-orange X-ray quality crystals of 3-Tb^Ph^ were obtained from a dilute reaction mixture (7 mM) after standing overnight in MeCN at −40 °C. Bulk isolation of 3-Tb^Ph^ can also be carried out in the absence of 2.2.2-cryptand, but in a slightly lower yield (43% yield). The ^1^H NMR spectrum of the isolated complex 3-Tb^Ph^ in toluene is silent, consistent with that of a Tb(iv) 4f^7^ ion (Fig. S27†).

Complex 3-Tb^Ph^ is stable in solution for 2 days at room temperature in toluene, but decomposes rapidly in THF at room temperature, as shown by ^1^H NMR studies, leading to the formation of 1-Tb^Ph^ and unknown species immediately after dissolution (Fig. S28†). The decomposition of 3-Tb^Ph^ into 1-Tb^Ph^ in THF, as measured by ^1^H NMR spectroscopy (using CH_2_Cl_2_ as the internal standard), leads to the formation of 1-Tb^Ph^ with 83% yield (Fig. S29†).

Variable-temperature magnetic and EPR data were measured on the isolated complex 3-Tb^Ph^ in order to confirm the presence of the Tb(iv) ion (Fig. 3). The measured *χ*_M_*T* value for the 4f^7^ complex 3-Tb^Ph^ at 300 K (8.06 emu K mol^−1^) is consistent with the values found in the previously reported Tb(iv) complexes [Tb^IV^(OSi(O^*t*^Bu)_3_)_4_] (7.77 emu K mol^−1^),^35^ [Tb^IV^(OSiPh_3_)_4_(MeCN)_2_] (7.82 emu K mol^−1^),^41^ and [Tb^IV^(NP(1,2-bis-^t^Bu-diamidoethane)_4_(NEt_2_))] (8.55 emu K mol^−1^),^36^ and are in agreement with the value of *χ*_M_*T* predicted for a 4f^7^ complex using the LS coupling for a 4f^7^ ion (*L* = 0, *S* = 7/2).^19^ The X-band EPR spectrum of 3-Tb^Ph^ measured at 6 K in the solid-state or toluene, displays strong features with *g*-values of [7.90, 5.00, 3.35] and [7.45, 4.55, 3.75], respectively, consistent with previously reported Tb(iv) complexes.^35,36,41^

The UV/vis spectrum of 3-Tb^Ph^ measured immediately after dissolution in toluene (1 mM) shows two absorption bands with *λ*_max_ at 285 and 355 nm, in which the absorption at 355 nm is most consistent with that of the previously reported siloxide-supported Tb(iv) complexes [Tb^IV^(OSi(O^*t*^Bu)_3_)_4_] (*λ*_max_ = 371 nm, toluene)^35^ and [Tb^IV^(OSiPh_3_)_4_(MeCN)_2_] (*λ*_max_ = 386 nm, THF).^41^

Monitoring the UV/vis spectra of 3-Tb^Ph^ in toluene (Fig. S61†) over time showed a high solution stability, where 82% of the complex remained after 48 hours. This high stability is consistent with the ^1^H NMR studies in toluene, and compares well to the stability of the previously reported Tb(iv) complex, [Tb^IV^(OSiPh_3_)_4_(Ph_3_PO)(MeCN)], where 80% of the complex is present after 96 hours at room temperature.^42^ Complex 3-Tb^Ph^ is the first example of an isolated molecular complex of Tb(iv) supported by a trianionic tripodal ligand.

Although the event observed in the cyclic voltammogram for 2-Pr^Ph^ was found to be irreversible at slow scan rates (50–400 mV s^−1^) (*vide infra*), chemical oxidation with “magic blue” was investigated. The addition of 1.1 equiv. of [N(C_6_H_4_Br)_3_][SbCl_6_] to complex 2-Pr^Ph^ with or without 2.2.2-cryptand in MeCN at −40 °C led to a dark brown-orange solution, which immediately faded to pale-yellow with the precipitation of a white solid. Colour-less crystals were isolated from the reaction mixture and characterized by X-ray diffraction studies as the MeCN adduct of complex 1-Pr^Ph^ (Fig. S53†), indicating that upon oxidation with [N(C_6_H_4_Br)_3_][SbCl_6_], the desired Pr(iv) readily decomposes at −40 °C.

We reasoned that the facile oxidation of 2-Tb^Ph^, leading to an insoluble Tb(iv) complex (3-Tb^Ph^), could provide a pathway for the separation of the Tb ion from a neighboring lanthanide (for example Dy), which presents a more positive oxidation potential (5.0 V calculated). Preliminary experiments were conducted on a MeCN reaction mixture containing 2-Tb^Ph^ and its Dy(iii) analogue, 2-Dy^Ph^, prepared *in situ*, in a 1 : 1 ratio.

Addition of [N(C_6_H_4_Br)_3_][SbCl_6_] to a 1 : 1 reaction mixture of 2-Tb^Ph^ : 2-Dy^Ph^ in the presence of 2.2.2-cryptand in MeCN at −40 °C led to the immediate precipitation of an orange solid. Extraction of the orange solid into toluene-*d*_8_ resulted in a dark orange solution, which displayed featureless ^1^H NMR spectra, suggesting that the Tb(iv) complex, 3-Tb^Ph^ was the major species in solution. The ^1^H NMR spectrum of the residue obtained after removal of toluene was measured in THF-*d*_8_ after 24 hours, allowing the Tb(iv) to decompose, showing the presence of 1-Tb^Ph^ as the major species and of a small amount of 1-Dy^Ph^ (6%). In contrast, the ^1^HNMR spectrum of the THF-*d*_8_ solution obtained upon dissolution of the residual solid fraction, after toluene extraction, shows a Tb : Dy ratio of 0.44 : 1.

The molar ratios of the two metals in the two fractions were determined both by ^1^H NMR and ICP-MS analyses. From these data, the separation factor, *S*_Tb/Dy_, was calculated from the enrichment factor (*S* = *D*_residual solid_·*D*_extracted solid_), *D*, which were determined from the molar ratios.

Overall, a separation factor of 10.1 was determined by ^1^H NMR spectroscopy and a similar value was obtained by ICP-MS measurements (8.56). These values compare well with the separation factor reported for neighboring lanthanides ranging from 1 to 4 using size sensitive supramolecular encapsulation and precipitation techniques.^50,57^ These preliminary separation trials suggest that the Tb(iv)/Tb(iii) redox couple may be used to implement effective separation of Tb from other lanthanides.

### Solid state structure and electronic structure of Ln(iv) complexes

**Fig. 2:**
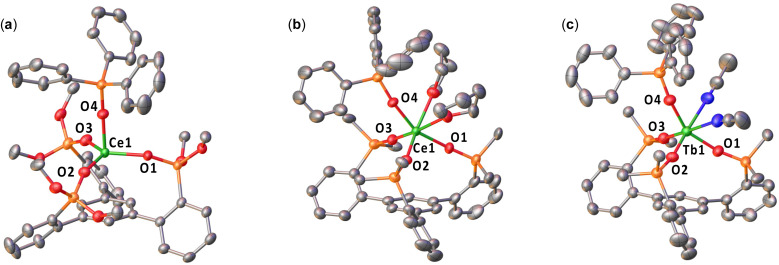
Molecular structures of (a) [Ce^IV^((OSi(O^*t*^Bu)_2_Ar)_3_-arene)(OSiPh_3_)] (3-Ce^O^*^t^*^Bu^), (b) [Ce^IV^((OSiPh_2_Ar)_3_-arene)(OSiPh_3_)(THF)_2_] (3-Ce^Ph^), and (c) [Tb^IV^((OSiPh_2_Ar)_3_-arene)(OSiPh_3_)(MeCN)_2_] (3-Tb^Ph^), with thermal ellipsoids drawn at the 50% probability level. Hydrogen atoms, methyl groups on –OSi(O^*t*^Bu)_2_, some disordered substituents and the five carbon atoms of each phenyl have been omitted for clarity.

The solid-state structures of complexes 3-Ce^OtBu^·3.5(toluene), 3-Ce^Ph^·4(THF), and 3-Tb^Ph^·2.5(MeCN) were determined by X-ray diffraction studies (Fig. 2 and Table 1).

**Fig. 3:**
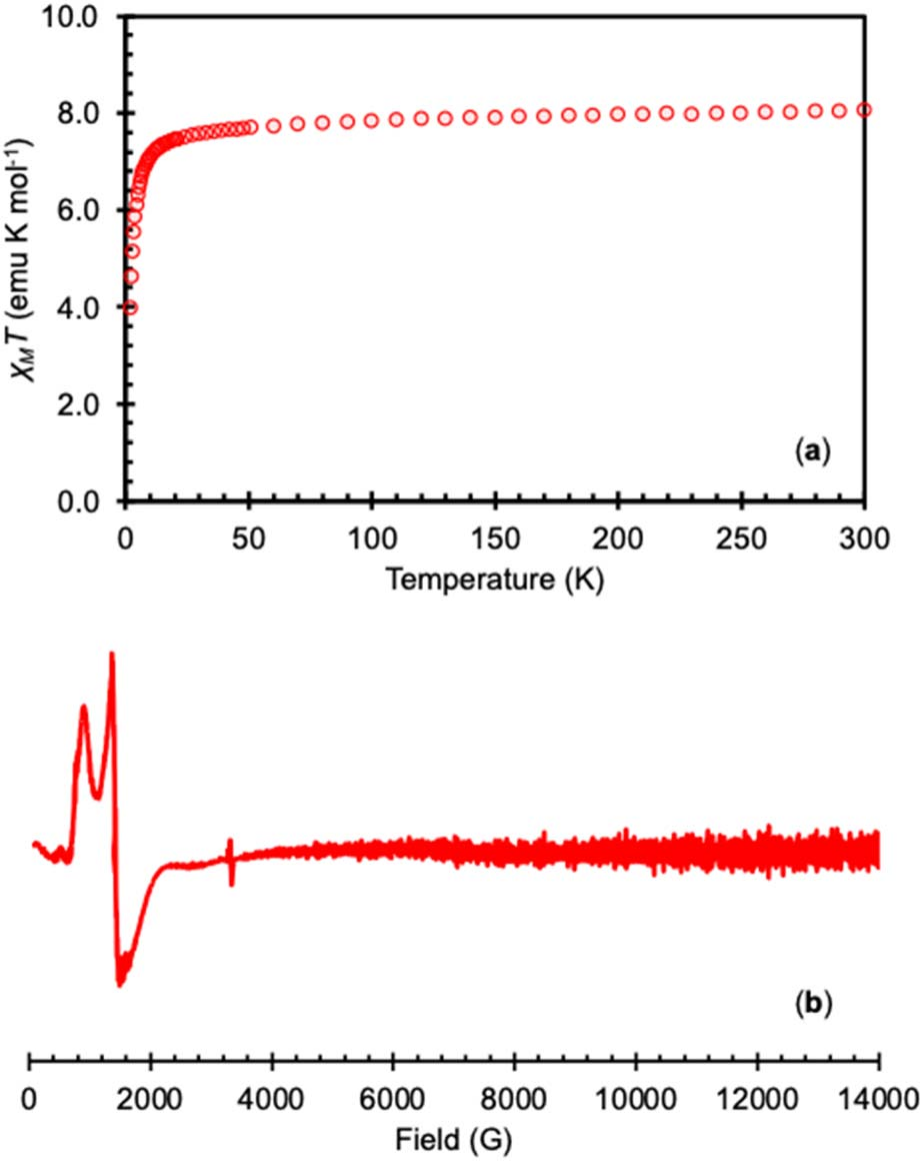
(a) Plot of *χ*_M_*T versus* temperature for isolated 3-Tb^Ph^ under an applied field of 1 T and (b) the X-band (9.4 GHz) EPR spectrum of complex 3-Tb^Ph^ in toluene (20 mM) at 6 K (bottom).

**Table 1:** Selected structural parameters of 2-Ln and 3-Ln

Complex	2-Ce^O^*^t^*^Bu^	2-Tb^O^*^t^*^Bu^	2-Tb^Ph^	2-Pr^Ph^	3-Ce^O^*^t^*^Bu^	3-Ce^Ph^	3-Tb^Ph^
Ln–O (Å)	2.216(8)–2.235(4)	2.115(8)–2.141(4)	2.129(6)–2.190(5)	2.228(3)–2.286(3)	2.0789(18)–2.1145(18)	2.121(4)–2.170(4)	2.026(2)–2.076(2)
Ln–C_centroid_ (Å)	3.284(5)	3.516(6)	3.232(4)	3.2835(16)	3.1010(11)	4.127(2)	4.0450(12)

The molecular structure of 3-Ce^O^*^t^*^Bu^ shows the presence of a mononuclear complex with the Ce bound by the three siloxides from the tripodal ligand and a monodentate triphenylsiloxide. The range of Ce–O_siloxide_ distances (2.0789(18)–2.1145(18) Å) is significantly shorter than those found in Ce(iii) precursor 2-Ce^O^*^t^*^Bu^ (2.216(8)–2.235(4) Å) and in the previously reported Ce(iii) [Ce(OSi(O^*t*^Bu)_3_Ar)_3_-arene)(THF)] (1-Ce^O^*^t^*^Bu^; 2.2344(18)–2.2377(18) Å),^48^ and is comparable to those found for the previously reported Ce(iv) complexes [Ce(OSi(O^*t*^Bu)_3_)_4_] (2.084(2)–2.160(2) Å)^58^ and [CeCl(OSi(O^*t*^Bu)_3_Ar)_3_-arene)] (2.097(4)–2.105(3) Å),^48^ which are in agreement with the presence of Ce(iv). Moreover, the Ce–C_centroid_ distance (3.1010(11) Å) is shorter than that in 2-Ce^O^*^t^*^Bu^ (3.284(5) Å).

The molecular structure of 3-Ce^Ph^ reveals the presence of a mononuclear Ce(iv) complex with a metal center bound by three –OSiPh_2_ groups of the tripodal ligand, one –OSiPh_3_, and two THF molecules, adopting a distorted octahedral geometry.

The range of Ce–O_siloxide_ distances (2.121(4)–2.170(4) Å) is consistent with the Ce–O_siloxide_ length found in the previously reported Ce(iv) complexes [Ce^IV^(OSiPh_3_)_4_(THF)_2_] (2.109(3)–2.154(3) Å)^59^ and [Ce^IV^(OSiPh_3_)_4_(DME)] (2.10(1)–2.13(1) Å).^60^ The Ce–O_siloxide_ and Ce–O_THF_ bond distances in 3-Ce^Ph^ are shorter compared to those found in the Ce(iii) complex 1-Ce^Ph^ (0.078 Å and 0.100 Å, respectively), and are in agreement with the difference in ionic radii between Ce(iii) and Ce(iv). Furthermore, the Ce–C_centroid_ distance has a significant increase in 3-Ce^Ph^ (4.127(2) Å) compared to 1-Ce^Ph^ (3.9234(9) Å).

The molecular structure of 3-Tb^Ph^ is similar to that of the Ce(iv) analogue 3-Ce^Ph^, with two coordinated MeCN molecules instead of THF. The range of Tb–O_siloxide_ distances (2.026(2)–2.035(2) Å) is consistent with the Tb–O_siloxide_ bond lengths found in the two previously reported Tb(iv) complexes [Tb^IV^(OSiPh_3_)_4_(MeCN)_2_] (2.028(5)–2.087(5) Å)^41^ and [Tb^IV^(OSi(O^*t*^Bu)_3_)_4_] (2.023(3)–2.093(3) Å).^35^ The shorter Tb–O_siloxide_ bond distances in 3-Tb^Ph^, compared to those of the Tb(iii) complex 1-Tb^Ph^ (2.139(3) Å) and 2-Tb^Ph^ (2.16(1) Å), are in agreement with the +4 oxidation state of the terbium metal center. Furthermore, the Tb–C_centroid_ distance has a significant increase in 3-Tb^Ph^ (4.045(1) Å) compared to 1-Tb^Ph^ (3.972(1) Å) and 2-Tb^Ph^ (3.232(4) Å).

Calculations were carried out on complex 3-Tb^Ph^. In order to ensure the formation of a Tb(iv) complex, the geometry optimization was carried out using f-in-core RECP for Tb which is adapted to the +4 oxidation state of Tb. This computational methodology has been shown to provide excellent geometric parameters.^61^ The optimized geometry of 3-Tb^Ph^ is in excellent agreement with the experimental data (Table S15†). The Tb–O_siloxide_ distances are reproduced with an accuracy of 0.02 Å. Therefore, one can safely conclude that complex 3-Tb^Ph^ implies the presence of a Tb(iv) center. As found experimentally and as expected with a stronger Lewis acid, the Tb–O_siloxide_ distances in 3-Tb^Ph^ are 0.15–0.18 Å shorter than in 2-Tb^Ph^, further corroborating the presence of a Tb(iv) center. Finally, the Tb–O_siloxide_ WBI (Table S17†) are in the 0.45–0.50 range indicating mainly ionic bonds, but a slightly greater covalency in Tb(iv) than in Tb(iii), as expected with the shorter distances that allow better orbital overlap. Computational studies carried out on an analogous complex, replacing the monodentate triphenylsiloxide with a *tert*-butoxide ligand (Table S19†), were also in agreement with the presence of a Tb(iv) center, suggesting that it may be possible to find the appropriate experimental conditions for its isolation in the solid state.

### Electrochemical studies

**Fig. 4:**
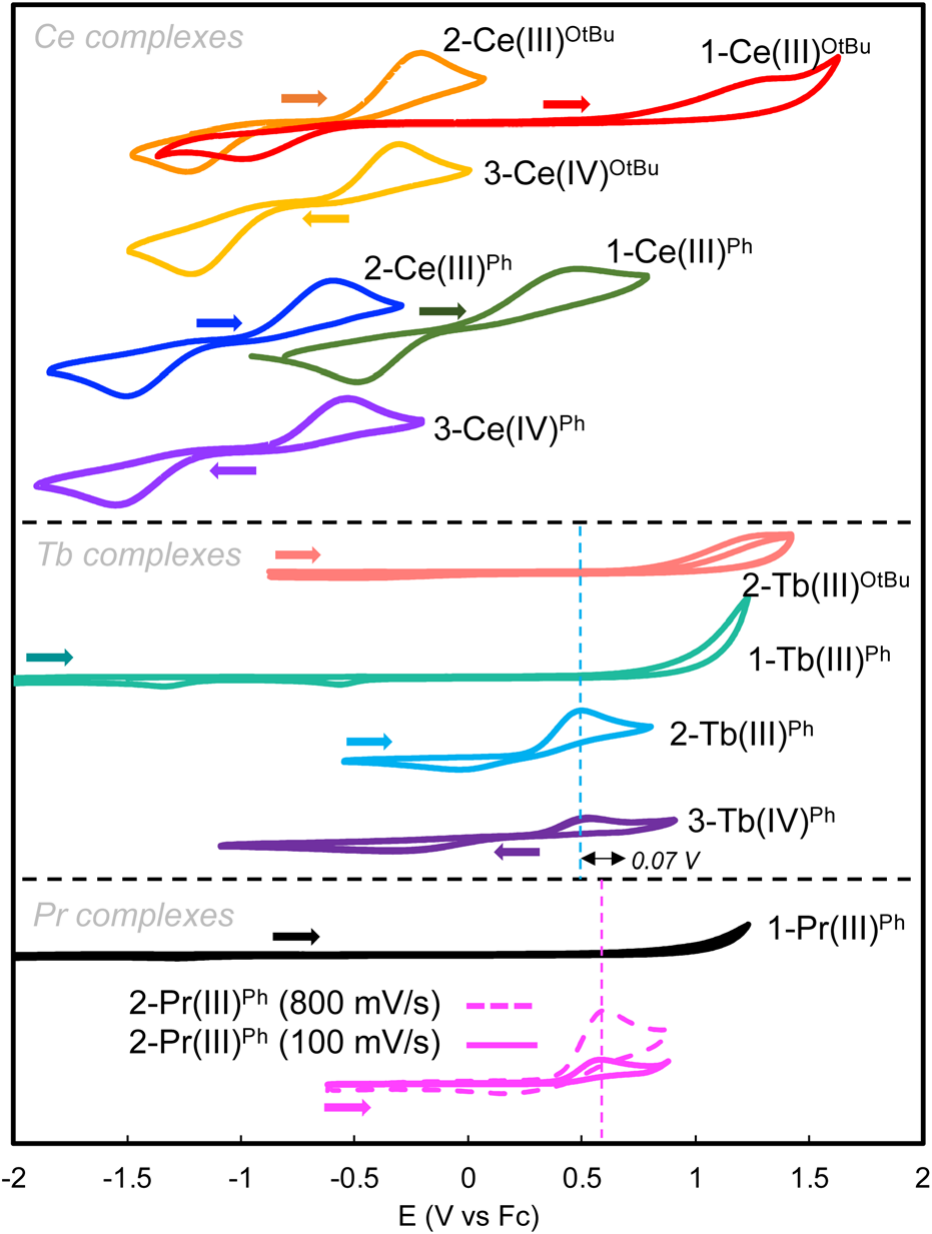
Cyclic voltammograms (CVs) for complexes 1-Ce^O^*^t^*^Bu^ (red); 2-Ce^O^*^t^*^Bu^ (orange); 3-Ce^O^*^t^*^Bu^ (yellow); 1-Ce^Ph^ (green); 2-Ce^Ph^ (blue); 3-Ce^Ph^ (purple), 2-Tb^O^*^t^*^Bu^ (peach); 1-Tb^Ph^ (teal), 2-Tb^Ph^ (light blue); 3-Tb^Ph^ (plum); 1-Pr^Ph^ (black) 2-Pr^Ph^ (pink); 0.1 M [NBu_4_][B(C_6_F_5_)_4_] electrolyte in THF. Conditions: Pt disk working electrode, referenced to the Fc^0^/Fc^+^ couple, scan rate for Ce (250 mV s^−1^) and Tb (100 mV s^−1^) Pr (100 mV s^−1^ solid line; 800 mV s^−1^ dotted line).

Cyclic voltammograms (CVs) were obtained for 3 mM THF solutions of complexes 1-Ce^O^*^t^*^Bu/Ph^, 2-Ce^O^*^t^*^Bu/Ph^, 3-Ce^O^*^t^*^Bu/Ph^, 1-Tb^O^*^t^*^Bu/Ph^, 2-Tb^O^*^t^*^Bu/Ph^, 3-Tb^Ph^, and 2-Pr^Ph^. The measurements were performed with 0.1 M [NBu_4_][B(C_6_F_5_)_4_] as the supporting electrolyte with decamethylferrocene (Fc*) as the internal reference (Fig. 4 and Table 2).

**Table 2:** Electrochemical data in V *vs.* Fc^0^/Fc^+^ for the 2-Ce^O^*^t^*^Bu^/^Ph^, 3-Ce^O^*^t^*^Bu^/^Ph^, 2-Tb^OtBu/Ph^, 3-Tb^Ph^, and 2-Pr^Ph^ complexes

Complex	2-Ce(iii)^O^*^t^*^Bu^	2-Ce(iii)^Ph^	3-Ce(iv)^O^*^t^*^Bu^	3-Ce(iv)^Ph^	2-Tb(iii)^O^*^t^*^Bu^	2-Tb(iii)^Ph^	3-Tb(iv)^Ph^	2-Pr(iii)^Ph^
*E*_pc_	−1.24	−1.50	−1.22	−1.54	—	−0.08	−0.33	—a
*E*_pa_	−0.21	−0.60	−0.30	−0.53	1.36	0.53	0.54	0.60

a The reduction wave was found to depend on the scan rate and occurs at 0.17 V *vs.* Fc^0^/Fc^+^ at 800 mV s^−1^ (Fig. S79).

The cyclic voltammogram of 1-Ce^O^*^t^*^Bu^ shows an oxidation feature at *E*_pa_ = 1.36 V *vs.* Fc^0^/Fc^+^, which is very close to the solvent window of THF, and a related reduction feature at *E*_pc_ = −0.98 V *vs.* Fc^0^/Fc^+^, with a peak separation of Δ*E*_pa_ = 2.33 V. Interestingly, addition of the –OSiPh_3_ ligand, forming 2-Ce^O^*^t^*^Bu^, significantly shifts the oxidation potential cathodically (*E*_pa_ = −0.21 V *vs.* Fc^0^/Fc^+^) compared to that of 1-Ce^O^*^t^*^Bu^ (*E*_pa_ = 1.36 V *vs.* Fc^0^/Fc^+^). The complexes of the –OSiPh_2_ substituted tripodal ligand, 1-Ce^Ph^ and 2-Ce^Ph^, exhibit a similar trend, in which the oxidation potential for 2-Ce^Ph^ (*E*_pa_ = −0.60 V *vs.* Fc^0^/Fc^+^) shifted 1.09 V more negative compared to that of 1-Ce^Ph^ (*E*_pa_ = 0.49 V *vs.* Fc^0^/Fc^+^). The cyclic voltammograms of the isolated Ce(iv) species, 3-Ce^O^*^t^*^Bu^ and 3-Ce^Ph^, exhibit comparable redox events (3-Ce^O^*^t^*^Bu^: *E*_pa_ = −0.30 V and *E*_pc_ = −1.22 V; 3-Ce^Ph^: *E*_pa_ = −0.53 V and *E*_pc_ = −1.54 V) to their Ce(iii) precursors, 2-Ce^O^*^t^*^Bu^ and 2-Ce^Ph^. In the case of complexes 1-Tb^Ph^ and 1-Pr^Ph^, no redox processes were observed in the window permitted by THF and the [NBu_4_][B(C_6_F_5_)_4_] electrolyte. This is in agreement with the oxidation potentials previously reported for Tb(iii) and Pr(iii) siloxide complexes,^35,37^ which where ∼1.1 V higher than those observed for the analogous Ce(iii) derivatives. Therefore, electrochemical oxidation of the complexes 1-Ln^Ph^ (Ln = Tb, Pr) is inaccessible in this potential range. However, when the –OSiPh_3_ ligand was added to form complexes 2-Ln^Ph^ (Ln = Tb, Pr), the cyclic voltammograms display redox events that are quasi-reversible for 2-Tb^Ph^, but appear to be irreversible at slow scan rates for 2-Pr^Ph^. The Tb(iii) derivatives follow the same trend as the analogous Ce(iii) complexes, where the oxidation potential shifts cathodically in 2-Tb^Ph^ (*E*_pa_ = 0.53 V *vs.* Fc^0^/Fc^+^) compared to 2-Tb^O^*^t^*^Bu^ (1.36 V *vs.* Fc^0^/Fc^+^).

**Fig. 5:**
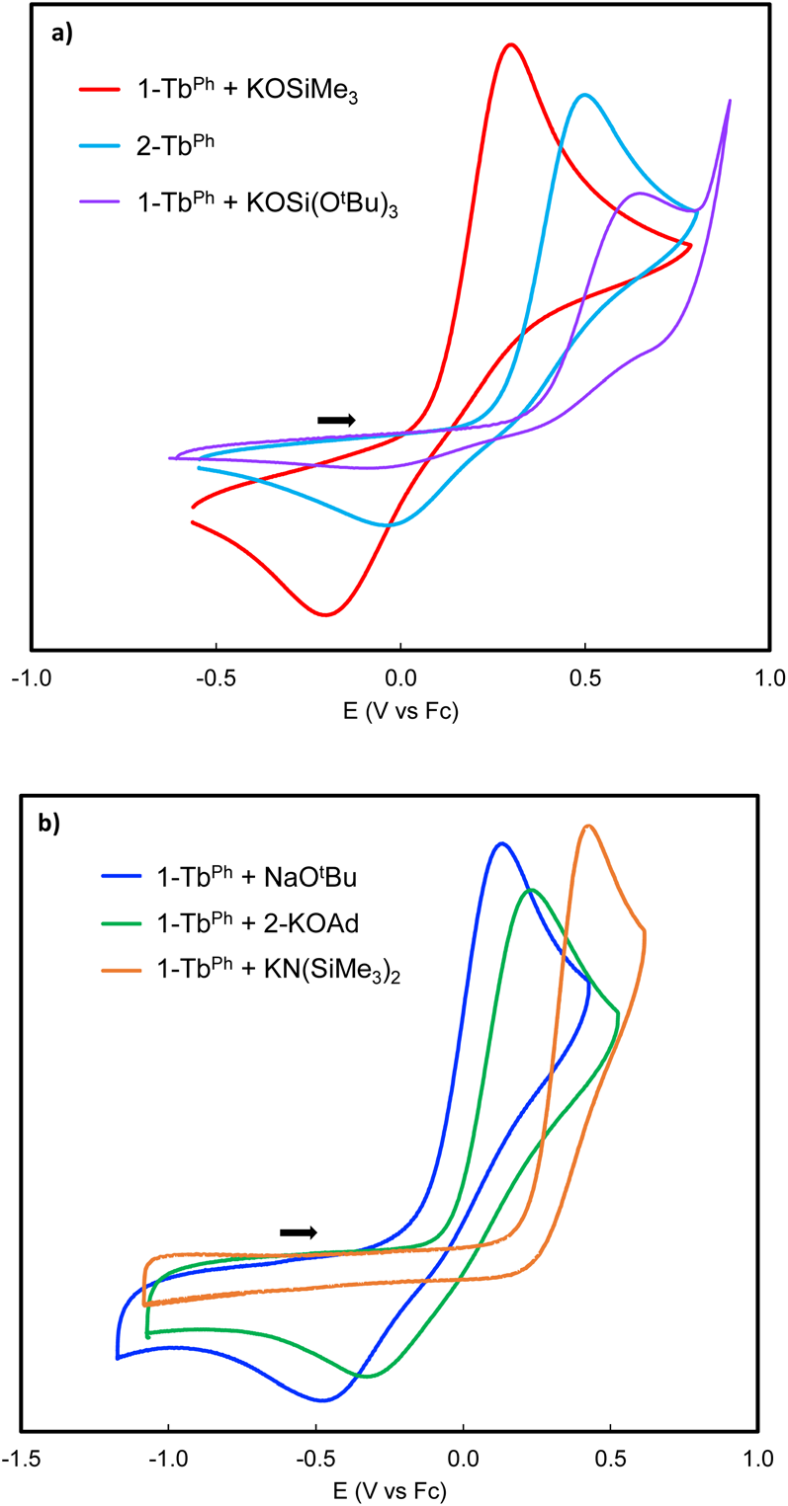
Cyclic voltammograms (CVs) of (a) complex 2-Tb^Ph^ (light blue), and 1-Tb^Ph^ after addition of 1 equiv. KOSiMe_3_ (red) and1 equiv. KOSi(O^*t*^Bu)_3_ (purple); (b) cyclic voltammograms (CVs) of 1-Tb^Ph^ after addition of 1.1 equiv. NaO^*t*^Bu (blue), 1.1 equiv. of 2-KOAd (green) and 1.1 equiv. KN(SiMe_3_)_2_ (orange), in 0.1 M [NBu_4_][B(C_6_F_5_)_4_] electrolyte in THF. Conditions: Pt disk working electrode, referenced to the Fc^0^/Fc^+^ couple, scan rate (100 mV s^−1^ (a), 1000 mV s^−1^ (b)).

Interestingly, the oxidation potential of complex 2-Tb^Ph^ is 1.13 V higher than the one found for 2-Ce^Ph^, and is only 0.12 V higher than that of the previously reported Tb(iii) complex, [KTb^III^(OSiPh_3_)_4_] (0.41 V *vs.* Fc^0^/Fc^+^).^41^ A reduction wave is also observed in 2-Tb^Ph^ (*E*_pc_ = −0.08 V *vs.*, Fc^0^/Fc^+^), suggesting that the Tb(iv) species is stable in solution at least within the electrochemical timeframe. Interestingly, changing the nature of the additional siloxide ligand by reacting 1.0 equiv. of KOSi(O^*t*^Bu)_3_ or KOSiMe_3_ with 1-Tb^Ph^, after recording their cyclic voltammograms, allowed significant shifts of the oxidation potentials (0.14 V higher and 0.19 V lower, respectively) (Fig. 5a). We also investigated the effect of adding different alkoxide (NaO^*t*^Bu and 2-KOAd (AdOH = 2-adamantanol), and amide (KN(SiMe_3_)_2_) ligands on the redox properties of the terbium complex (see the ESI†). The addition of NaO^*t*^Bu, 2-KOAd, and KN(SiMe_3_)_2_ to 1-Tb^Ph^ resulted in the *in situ* formation of the respective ate-complexes, as indicated by ^1^H NMR studies. The –O^*t*^Bu, –OAd, and –N(SiMe_3_)_2_ complexes showed *E*_pa_ values of 0.11 V, 0.23 V, and 0.35 V, respectively, at a scan rate of 100 mV s^−1^ (Fig. S82–S84†).

Only for the adducts of 1-Tb^Ph^ with NaO^*t*^Bu and 2-KOAd, the associated reduction processes could be observed at higher scan rates (Fig. 5b and S82–S83†). Preliminary attempts to isolate Tb(iv) products were unsuccessful due to their high reactivity in the solvents utilized, but these electrochemistry studies suggest that alkoxides may also provide suitable ligands for the isolation of Tb(iv) species. Although the Tb(iv) complex 3-Tb^Ph^ was found to be unstable in THF by ^1^H NMR studies, it was possible to record its cyclic voltammogram immediately after dissolution at room temperature. Scanning reductively, an event was observed (*E*_pc_ = −0.33 V *vs.* Fc^0^/Fc^+^), which is ∼0.3 V lower than that observed for the parent 2-Tb^Ph^ complex (*E*_pc_ = −0.08 V *vs.*, Fc^0^/Fc^+^). This suggests an enhanced kinetic stability of the Tb(iv) complex in the absence of K^+^ cations, similar to what was previously observed in the homoleptic Tb(iv) complex, [Tb^IV^(OSiPh_3_)_4_(MeCN)_2_].^41^

Compared to the complexes 2-Ln^Ph^ (Ln = Ce, Tb), the Pr(iii) derivative, 2-Pr^Ph^, displays an oxidative event (*E*_pa_ = 0.60 V *vs.* Fc^0^/Fc^+^) with no corresponding reduction wave at a slow scan rate (50–400 mV s^−1^). However, a reduction feature could be observed at faster scan rates (600–1200 mV s^−1^) (Fig. S79†). Additionally, for 1-Pr^Ph^, additional ligands, KOSiMe_3_ and 2-KOAd, were examined. They showed lower oxidation potentials (*E*_pa_ = 0.27 and 0.48 V, 100 mV s^−1^) (Fig. S85–S86†) compared to 2-Pr^Ph^. Addition of KOSiMe_3_ also allowed the observation of a reduction process at different scan rates (50–1000 mV s^−1^) (Fig. S84†). However, in the case of 2-KOAd, no reduction processes could be observed in the cyclic voltammograms at different scan rates (50–1000 mV s^−1^) (Fig. S86†). Attempts to isolate a molecular Pr(iv) species using different solvents and varying temperatures were not successful. The CV results suggest that transient, highly reactive Pr(iv) species are generated from the electrochemical oxidation of 2-Pr^Ph^, and of the –OSiMe_3_ adduct, but they are unstable under the solvent/electrolyte conditions employed, as observed for other Pr(iii) systems.^38^

Overall, the cyclic voltammograms of the Ce(iii) and Tb(iii) complexes show that replacing the tris(*tert*-butoxy) moieties for phenyl substituents shifts the oxidation potentials to more negative values. This may be attributed to the combination of two influences; (1) the presence of the more electron-donating –OSiPh_2_ groups, compared to the *tert*-butoxy moieties on the tripodal ligand, makes the oxidation to Ln(IV) (Ln = Ce, Tb) more accessible; and (2) the presence of a metal-arene δ bonding interaction in 1-Ln^O^*^t^*^Bu^, which is absent in 1-Ln^Ph^ as corroborated by structural and DFT studies, is likely to stabilize the Ln(III) metal center, rendering the oxidation in 1-Ln^O^*^t^*^Bu^ more difficult. Furthermore, the coordination of an additional ligand, –OSiPh_3_, –OSi(O^*t*^Bu)_3_, or –OSiMe_3_, further shifts the potential cathodically, making the Ln(iv) even more accessible. This may be attributed to the increased electron donation from the additional siloxide ligand and to the formation of neutral Ln(iv) ions, which should be more favorable than the formation of a positively charged Ln(iv) ion in complexes 1-Ln^O^*^t^*^Bu/Ph^ (Ln = Ce, Tb).

## Conclusions

Herein, we developed a new synthetic strategy to isolate molecular complexes of lanthanide ions, in the +4 oxidation state, which combined (1) the use of polydentate siloxide-derived ligands in order to control unwanted ligand rearrangements; and (2) utilizing different ancillary substituents on the tripodal framework and coordination of an additional monodentate ligand to tune the Ln(iii)/Ln(iv) redox-couple. The Ln(iii) complexes, [Ln^III^((OSiPh_2_Ar)_3_-arene)(THF)_3_] (1-Ln^Ph^) and [K(toluene){Ln^III^((OSiPh_2_Ar)_3_-arene)(OSiPh_3_)}] (2-Ln^Ph^), (Ln = Ce, Tb, Pr), of the (HOSiPh_2_Ar)_3_-arene ligand^1,2^ were prepared and crystallographically characterized. The redox properties of these complexes where determined by cyclic voltammetry studies, and compared to those of the Ln(iii) analogue complexes, [Ln^III^((OSi(O^*t*^Bu)_2_Ar)_3_-arene)(THF)] (1-Ln^O^*^t^*^Bu^) and [K(THF)_6_][Ln^III^((OSi(O^*t*^Bu)_2_Ar)_3_-arene)(OSiPh_3_)] (2-Ln^O^*^t^*^Bu^) (Ln = Ce, Tb), of the less electron-donating *tert*-butoxy siloxide tripodal ligand, (HOSi(O^*t*^Bu)_2_Ar)_3_-arene. The cyclic voltammetry studies showed a cathodically shifted oxidation potential for the Ce(iii) and Tb(iii) complexes of the more electron-donating phenyl-substituted tripodal ligand (1-Ln^Ph^), compared to those of the *tert*-butoxy tripodal (1-Ln^O^*^t^*^Bu^). Moreover, *in situ* electrochemical studies showed that the coordination of an additional monoanionic siloxide (–OSiPh_3_, –OSi(O^*t*^Bu)_3_, and –OSiMe_3_) or alkoxide (–O^*t*^Bu and –OAd) ligand further shifts the potential cathodically. The nature of the added monoanionic ligand is crucial in tuning the potential cathodically, rendering the isolation and characterization of the Ln(IV) complexes possible. Notably, the Ce(iv) complexes [Ce^IV^((OSi(O^*t*^Bu)_2_Ar)_3_-arene)(OSiPh_3_)] (3-Ce^O^*^t^*^Bu^) and [Ce^IV^ ((OSiPh_2_Ar)_3_-arene)(OSiPh_3_)(THF)_2_] (3-Ce^Ph^) were prepared by chemical oxidation of the Ce(iii) analogues. Chemical oxidation of the Tb(iii) complex was also possible, and the Tb(iv) complex [Tb^IV^((OSiPh_2_Ar)_3_-arene)(OSiPh_3_)(MeCN)_2_] (3-Tb^Ph^) was isolated and crystallographically characterized yielding the first example of a Tb(iv) supported by a tripodal ligand. The versatility and robustness of the siloxide arene-anchored scaffolds presented here will allow further development in the isolation of more oxidizing Ln(IV) ions, widening the breadth of high-valent Ln chemistry. Preliminary experiments also anticipate the possibility of using the Ln(iv)/Ln(iii) couple for the separation of neighboring lanthanides.

## Data availability

Synthetic details, analytical data including depictions of all spectra and coordinate data of all computationally optimised species, are documented in the ESI.† Crystallographic data is made available *via* the CCDC. The data that support the findings of this study are openly available in the Zenodo repository at https://doi.org/10.5281/zenodo.10890239.

## Author contributions

M. T., F.-C. H. designed and carried out all the experiments and analyzed the data; M. K. Isolated and characterised the Tb(iv) complex. M. M. designed and supervised the project; T. R. and L. M. carried out the computational study; R. S. measured and analyzed the X-ray data; I. Z. measured and analysed the magnetic data. M. T., F.-C. H., M. K., L. M., and M. M. wrote the manuscript with contributions of all authors, and all authors have given approval for the final version of the manuscript.

## Conflicts of interest

The authors declare no conflict of interest.

## Supplementary Material

SC-015-D4SC00051J-s001

SC-015-D4SC00051J-s002
