# Supplementary material for: Siloxide tripodal ligands as a scaffold for stabilizing lanthanides in the +4 oxidation state
Source: Chem Sci. 2024 Apr 2;15(18):6874–83. doi: 10.1039/d4sc00051j (PMC11077534; doi:10.1039/d4sc00051j)
Supplement: SC-015-D4SC00051J-s001 [file SC-015-D4SC00051J-s001.pdf]

Electronic Supplementary Material (ESI) for Chemical Science.
This journal is © The Royal Society of Chemistry 2024

# Siloxide tripodal ligands as a scaffold for stabilizing lanthanides in the +IV oxidation state.

Maxime Tricoire,[a§] Fang-Che Hsueh,[a§] Megan Keener,[a§] Thayalan Rajeshkumar,[b] Rosario Scopelliti,[c] Ivica Zivkovic,[d] Laurent Maron[b] and Marinella Mazzanti [a*]

[a]Group of Coordination Chemistry, Institut des Sciences et Ingénierie Chimiques, École Polytechnique Fédérale de Lausanne (EPFL), CH-1015 Lausanne, Switzerland.
[b] Laboratoire de Physique et Chimie des Nano-objets, Institut National des Sciences Appliquées, 31077 Toulouse, Cedex 4, France.
[c]X-Ray Diffraction and Surface Analytics Platform, Institut des Sciences et Ingénierie Chimiques, École Polytechnique Fédérale de Lausanne (EPFL), CH-1015 Lausanne, Switzerland.
[d]Laboratory for Quantum Magnetism, Institute of Physics, Ecole Polytechnique Fédérale de Lausanne (EPFL), CH-1015 Lausanne, Switzerland

*Email to whom correspondence should be addressed: marinella.mazzanti@epfl.ch, laurent.maron@irsamc.ups-tlse.fr

## Table of Contents

S1. Materials and Physical Measurements ........ 2
S2. Synthesis ........ 4
S2.1. Synthesis and reactivity of cerium complexes ........ 4
S2.2. Synthesis and reactivity of terbium complexes ........ 5
S2.3. Synthesis and reactivity of praseodymium complexes ........ 7
S2.4. Separation experiments ........ 8
S3. NMR Spectroscopic Data ........ 9
S3.1. NMR spectra for cerium complexes ........ 9
S3.2. NMR spectra for terbium complexes ........ 24
S3.3. NMR spectra for praseodymium complexes ........ 38
S3.4. NMR spectra of in-situ addition of $KOSi(O^tBu)_3$, $KOSiMe_3$ and 2-KOAd and $K(N(SiMe_3)_2)$ to 1-$Tb^{Ph}$ ... 43
S3.5. NMR spectra of in-situ addition of $KOSiMe_3$ and 2-KOAd to 1-$Pr^{Ph}$ ........ 48
S3.6. NMR spectra of the Tb/Dy separation trials ........ 50
S4. X-ray Crystallography Data ........ 55
S5. EPR Data ........ 63
S6. Magnetization Data ........ 64
S7. UV/Vis Data ........ 67
S8. Electrochemistry Data ........ 73
S9. Infrared (IR) spectra ........ 82
S10. Determination of Enrichment and Separation factors ........ 88
S11. Computational details ........ 89
S12. References ........ 108

## S1. Materials and Physical Measurements

**General Considerations**

Unless otherwise noted, all manipulations were carried out at ambient temperature under an inert argon or nitrogen atmosphere using Schlenk techniques and an MBraun glovebox equipped with a purifier unit. The water and oxygen levels were always kept at less than 0.1 ppm. Glassware was dried overnight at 140 °C before use.

**NMR experiments** were carried out using NMR tubes adapted with J-Young valves. NMR spectra were recorded on Bruker 400, 500, or 600 MHz spectrometers and referenced to residual solvent resonances of THF ($d_8$-THF), toluene ($d_8$-toluene), or Acetonitrile ($d_3$-MeCN) in Pyrex NMR tubes adapted with J-Young valves.

**Elemental analyses** were performed under an inert atmosphere of nitrogen with a ThermoScientific Flash 2000 Organic Elemental Analyzer.

**Cyclic voltammetry** data were carried out at room temperature in an argon-filled glovebox described above. Data were collected using a Biologic SP-300 potentiostat connected to a personal computer. All samples were measured with 0.1 M $[NBu_4][B(C_6F_5)_4]$[1] supporting electrolyte in THF solution. The experiments were carried out with a platinum disk (d = 5 mm) working electrode, a platinum wire counter electrode, and an Ag/AgCl reference electrode. Potential calibration was performed at the end of each data collection cycle using the decamethylferrocene/decamethylferrocenium couple as an internal standard.

**Starting materials** Unless otherwise noted, reagents were purchased from commercial suppliers and used without further purification. Anhydrous solvents were purchased from Aldrich and further distilled from K/benzophenone (THF, $Et_2O$ and toluene), sodium sand/benzophenone (n-hexane) or $CaH_2$ (MeCN). Deuterated solvents for NMR spectroscopy were purchased from Cortecnet, freeze-degassed and distilled over K/benzophenone (THF-$d_8$, toluene-$d_8$) or $CaH_2$ (MeCN-$d_3$). Potassium bis(trimethylsilyl)amide (KHMDS), cerium chloride, triphenylsilanol ($HOSiPh_3$) and sodium *tert*-butoxide ($NaO^tBu$) were purchased from Sigma-Aldrich and dried under high vacuum prior to use. *Tert*-butyllithium solution (1.7 M in pentane), imidazole, dimethoxydiphenylsilane, and $[N(C_6H_4Br)_3][SbCl_6]$ were purchased from Sigma-Aldrich and used as received. $AgBPh_4$[2], $KOSiPh_3$[3] $[Ln(N(SiMe_3)_2)_3]$[4,5] (Ln=Ce, Pr and Tb), $(HOSiPh_2Ar)_3$-arene[6], $(HOSi(O^tBu)_2Ar)_3$-arene)[7], 2-KOAd[22] (AdOH= 1-adamantanol) and $[Ce((OSi(O^tBu)_2Ar)_3\text{-arene})(THF)]$[7] (**1-Ce$^{OtBu}$**) were prepared according to the published procedure.

**EPR** analysis were performed on a Bruker Elexsys E500 spectrometer working at 9.4 GHz frequency with an Oxford ESR900 cryostat for 4-300 K operation. Simulations were performed with the Easyspin 5.1.3 program.[8]

**Magnetization** were performed using a QuantumDesign MPMS3 superconducting quantum interference device (SQUID) magnetometer in a temperature range 2-300 K. The powder sample was constrained in eicosane and enclosed in an evacuated quartz capsule and placed inside a plastic straw. The measurement was performed with applied magnetic field of 1 T in the zero-field cooled (ZFC) regime and performed on two independent samples for reproducibility. Diamagnetic corrections were applied using Pascal's constants.[9] The magnetic moment was calculated using the formula:

$$\mu_{eff} = \sqrt{8\chi T}$$

**UV/Vis** data were recorded using 1.0 mm cuvettes equipped with a J-Young valve and a Perkin Elmer 950 spectrometer.

**IR spectra** were recorded with a Perkin Elmer 1600 Series FTIR spectrophotometer flushed with nitrogen.

**ICP-MS** samples were submitted to acidic digestion with 5 mL of concentrated HNO3 (69%, ROTIPURAN Supra, Roth) in PP digestion vials using heating block system (DigiPREP Jr. 15ml, 40 Pos, SCP Science). Digestion program was the following: 10 min to heat up to 100°C and keep at 100°C for 60 min. After the digestion samples were further diluted 300 times with 2% HNO3 solution and their Tb & Dy content was analyzed by ICP-MS using KED mode with He as a collision gas on NexIon 350 D ICP-MS instrument (PerkinElmer). Hf was added as an internal standard at concentration of 2 ppb to all the solution and absolute quantitation was performed using external calibration curve with standards in 0.05-50 ppb range. All measurements were performed in triplicates.

**X-ray crystallography** data for the analyzed crystal structures were selected and mounted on various Rigaku diffractometers (XtaLAB Synergy R, DW system, HyPix-Arc 150 detector or SuperNova, Dual, Cu at home/near, AtlasS type detectors). The crystals were kept at a steady $T$ = 140.00(10) K during data collection. Data were measured using $\omega$ scans with Cu $K_\alpha$ radiation. The diffraction patterns were indexed and the total number of runs and images were based on the strategy calculation from the program CrysAlisPro 1.171.42.72a (Rigaku OD, 2022).[10] The unit cells were refined using CrysAlisPro 1.171.42.72a (Rigaku OD, 2022).[10] Data reduction, scaling and absorption corrections were performed using CrysAlisPro 1.171.42.72a (Rigaku OD, 2022).[10] The structures were solved with the **ShelXT** (Sheldrick, 2015)[11] solution program using dual methods and by using **Olex2** 1.5 (Dolomanov et al., 2009)[12] as the graphical interface. The models were refined with **ShelXL** 2018/3 (Sheldrick, 2015)[13] using full matrix least squares minimization on $\boldsymbol{F}^2$. All non-hydrogen atoms were refined anisotropically. The positions of the hydrogen atom were calculated geometrically and refined using the riding model. Several structures displayed problems dealing with disorder (disordered ligands or solvent) or twinning. The major employed technique was the split model combined with a series of restraints and constraints. The restraints and constraints are used in order to get acceptable bond lengths and angles and/or anisotropic behavior. In some cases, the twinning treatment has been used for real twins or for multi-crystals, in order to properly separate the different domains. Finally, in some structures the solvent molecules were difficult to handle and the mask algorithm (by Olex2) was used to squeeze them completely from the final model.

**CCDC numbers** are 2296828 (complex **1-Tb$^{\mathbf{OtBu}}$**), 2296833 (complex **1-Ce$^{\mathbf{Ph}}$**), 2296827 (complex **1-Tb$^{\mathbf{Ph}}$**), 2296886 (complex **1-Pr$^{\mathbf{Ph}}$**), 2296835 (complex **2-Ce$^{\mathbf{OtBu}}$**), 2296831 (complex **2-Tb$^{\mathbf{OtBu}}$**), 2296830 (complex **2-Tb$^{\mathbf{Ph}}$**), 2296887 (complex **2-Pr$^{\mathbf{Ph}}$**), 2291801 (complex **3-Ce$^{\mathbf{OtBu}}$**), 2296834 (complex **3-Ce$^{\mathbf{Ph}}$**), 2296832 (complex **3-Tb$^{\mathbf{Ph}}$**), 2296829 (MeCN adduct of **1-Pr$^{\mathbf{Ph}}$**) and 2330604 (complex **2-Tb$^{\mathbf{Ph}}$-MeCN·(Et$_2$O)**).

## S2. Synthesis

### S2.1. Synthesis and reactivity of cerium complexes

**Synthesis of [$Ce^{III}$(($OSiPh_2Ar)_3$-arene)$(THF)_3$] (1-$Ce^{Ph}$):** A cold (-40 °C) yellow solution of [Ce($N(SiMe_3)_2)_3$] (147.0 mg, 0.2366 mmol, 1.0 equiv.) in THF (1.0 mL) was added to a cold (-40 °C) stirring colorless solution of free ligand ($HOSiPh_2Ar)_3$-arene (213.3 mg, 0.2367 mmol, 1.0 equiv.) in THF (3.0 mL). The reaction mixture was then warmed to room temperature and stirred for 16 h, yielding a colorless solution. The resulting solution was stored at -40 °C overnight affording white powder of complex **1-$Ce^{Ph}$** (243.9 mg, 82%). H NMR (400 MHz, THF-$d_8$, 298 K): δ 12.54 ppm (br s, 12H, -$OSiPh_2$), δ 9.22 ppm (d, $J$ = 7.5 Hz, 3H, tripodal arene), δ 8.73 ppm (br s, 12H, -$OSiPh_2$), δ 8.15 ppm (br s, 6H, -$OSiPh_2$), δ 7.27 ppm (t, $J$ = 7.2 Hz, 3H, tripodal arene), δ 6.08 ppm (t, $J$ = 7.3 Hz, 3H, tripodal arene), δ 2.86 ppm (d, $J$ = 7.3 Hz, 3H, tripodal arene), δ -2.12 ppm (s, 3H, tripodal arene)(**Figure S2**). *Anal. Calcd.* for **1-$Ce^{Ph}$**•$(THF)_{1.5}$, $C_{78}H_{81}O_{7.5}Si_3Ce$: C: 68.74; H: 5.99; N: 0.00. *Found*: C: 68.17; H: 5.62; N: 0.00. The fractional THF content is residual solvent from the bulk synthesis. X-ray quality crystals of **1-$Ce^{Ph}$•2toluene** could be isolated from concentrated toluene solution at room temperature.

**Synthesis of [$K(THF)_6$][$Ce^{III}$((OSi($O^tBu)_2Ar)_3$-arene)($OSiPh_3$)](2-$Ce^{OtBu}$):** A colorless solution of $KOSiPh_3$ (15.3 mg, 0.0486 mmol, 1.0 equiv.) in THF (1.0 mL) was added to a light-yellow solution of complex [Ce((OSi($O^tBu)_2Ar)_3$-arene)(THF)](**1-$Ce^{OtBu}$**) (52.9 mg, 0.0487 mmol, 1.0 equiv.) in THF (2.0 mL). The reaction mixture was stirred for 30 mins at room temperature. The solution was concentrated under vacuum and the resulting solution was stored at -40 °C overnight affording pale yellow powder of complex **2-$Ce^{OtBu}$** (55.6 mg, 86%). X-ray quality crystals of **2-$Ce^{OtBu}$** could be isolated from concentrated THF solution at room temperature. $^1$H NMR (400 MHz, THF-$d_8$, 298 K): δ 8.86 ppm (s, 3H, tripodal arene), δ 7.24 ppm (t, $J$ = 7.4 Hz, 3H, tripodal arene), δ 6.68 – 6.61 ppm (m, 6H, tripodal arene and -$OSiPh_3$), δ 6.52 ppm (t, $J$ = 6.5 Hz, 6H, -$OSiPh_3$), δ 5.00 ppm (d, $J$ = 7.1 Hz, 3H, tripodal arene), δ 4.45 ppm (s, 6H, -$OSiPh_3$), δ 3.37 ppm (s, 3H, tripodal arene), δ 2.34 ppm (s, 54H, -OSi($O^tBu)_2$)(**Figure S4**). *Anal. Calcd.* for [$K(THF)_{0.4}$][$Ce^{III}$((OSi($O^tBu)_2Ar)_3$-arene)($OSiPh_3$), $C_{67.6}H_{87.2}KO_{10.4}Si_4Ce$: C: 59.80; H: 6.47; N: 0.00. *Found*: C: 59.44; H: 6.74; N: 0.00. The potassium bound THF was lost during the drying process.

**Synthesis of [K(toluene){$Ce^{III}$(($OSiPh_2Ar)_3$-arene)($OSiPh_3$)}](2-$Ce^{Ph}$):** A colorless solution of $KOSiPh_3$ (13.2 mg, 0.0420 mmol, 1.0 equiv.) in THF (1.0 mL) was added to a colorless solution of complex [Ce(($OSiPh_2Ar)_3$-arene)$(THF)_3$] (**1-$Ce^{Ph}$**) (52.0 mg, 0.0414 mmol, 1.0 equiv.) in THF (4.0 mL). The reaction mixture was stirred for 30 mins at room temperature. The volatiles were removed under vacuum and the residue was dissolved in toluene (0.2 mL). The resulting solution was stored at -40 °C overnight affording pale yellow powder of complex **2-$Ce^{Ph}$** (55.1 mg, 92%). X-ray quality crystals were obtained by cooling a concentrated toluene solution to -40 °C. $^1$H NMR (400 MHz, THF-$d_8$, 298 K): δ 9.65 ppm (s, 12H, -$OSiPh_2$), δ 8.19 ppm (d, $J$ = 7.4 Hz, 3H, tripodal arene), δ δ 7.56 – 7.45 ppm (m, 18H, -$OSiPh_2$ and -$OSiPh_3$), δ 7.42 ppm (t, $J$ = 7.2 Hz, 6H, -$OSiPh_3$), δ 7.05 – 6.98 ppm (m, 6H, tripodal arene), δ 6.81 ppm (s, 6H, -$OSiPh_2$), δ 6.49 ppm (t, $J$ = 7.3 Hz, 3H, -$OSiPh_3$), δ 4.86 ppm (d, $J$ = 7.5 Hz, 3H, tripodal arene), δ 3.49 ppm (s, 3H, tripodal arene)(**Figure S6**). *Anal. Calcd.* for [K{$Ce^{III}$(($OSiPh_2Ar)_3$-arene)($OSiPh_3$)}], $C_{78}H_{60}KO_4Si_4Ce$: C: 69.25; H: 4.47; N: 0.00. *Found*: C: 68.87; H: 4.78; N: 0.00. The potassium bound toluene was lost during the drying process.

**Reaction of complex 2-$Ce^{Ph}$ with 1.1 equiv. of 2.2.2-cryptand in THF:** A 2.2.2-cryptand (1.9 mg, 0.0050 mmol, 1.1 equiv.) solution in THF-$d_8$ (0.1 mL) was added to a light-yellow solution of **2-$Ce^{Ph}$** (6.7 mg, 0.0046 mmol, 1.0 equiv.) in THF-$d_8$ (0.4 mL). The $^1$H NMR spectrum of the resulting solution showed the appearance of a new set of resonances, indicating the $K^+$ ion was bound in the **2-$Ce^{Ph}$** in THF solution (**Figure S7**).

**Synthesis [$Ce^{IV}$((OSi($O^tBu)_2Ar)_3$-arene)($OSiPh_3$)](3-$Ce^{OtBu}$):** A white suspension of $AgBPh_4$ (23.0 mg, 0.0539 mmol, 1.1 equiv.) in THF (1.0 mL) was added to a light-yellow solution of complex **2-$Ce^{OtBu}$** (64.7 mg, 0.0489 mmol, 1.0 equiv.) in THF (2.0 mL). The reaction mixture was stirred at room temperature for 2 h, resulting in a gray suspension. The suspension was filtered on a porosity 4 glass frit and the volatiles of the yellow filtrate were removed under vacuum. The pale-yellow residue was dried under vacuum for 30 minutes, dissolved in toluene (1.0 mL) and filtered. The resulting solution was stored at -40 °C overnight affording yellow powder of complex **3-$Ce^{OtBu}$** (50.9 mg, 81%) that was thoroughly dried under vacuum to remove co-crystallized toluene. X-ray quality crystals were obtained by cooling a concentrated toluene solution to -40 °C. $^1$H NMR (500 MHz, THF-$d_8$, 298 K): δ 8.40 ppm (d, $J$ = 1.5 Hz, 3H, tripodal arene), δ 7.88 ppm (d, $J$ = 7.9 Hz, 3H, tripodal arene), δ 7.60 ppm (d, $J$ = 7.5 Hz, 6H, -$OSiPh_3$), δ 7.39 – 7.36 ppm (m, 6H, tripodal arene

and -OSiPh$_3$), δ 7.32 ppm – 7.28 ppm (m, 6H, tripodal arene), δ 7.22 ppm (t, $J$ = 7.6 Hz, 6H, -OSiPh$_3$), δ 1.12 ppm (s, 54H, -OSi(O$^t$Bu)$_2$))(**Figure S9**). $^{13}$C{$^1$H} NMR (126 MHz, THF-$d_8$, 298 K): δ 147.40 ppm, δ 145.52 ppm, δ 138.80 ppm, δ 138.06 ppm, δ 137.26 ppm, δ 136.18 ppm, δ 130.50 ppm, δ 130.29 ppm, δ 129.73 ppm, δ 129.62 ppm, δ 127.88 ppm, δ 126.82 ppm, δ 73.51 ppm, δ 32.36 ppm (**Figure S10**). $^{29}$Si{$^1$H} NMR (79.5 MHz, THF-$d_8$, 298 K): δ -80.62 ppm (-OSi(O$^t$Bu)$_2$ and -OSiPh$_2$)(**Figure S11**). *Anal. Calcd.* for **3-Ce$^{OtBu}$**, $C_{66}H_{84}O_{10}Si_4Ce$: C: 61.46; H: 6.56; N: 0.00. *Found*: C: 61.50; H: 6.77; N: 0.00.

**Synthesis [Ce$^{IV}$((OSiPh$_2$Ar)$_3$-arene)(OSiPh$_3$)(THF)$_2$](3-Ce$^{Ph}$):** A white suspension of $AgBPh_4$ (20.3 mg, 0.0475 mmol, 1.1 equiv.) in THF (1.0 mL) was added to a light-yellow solution of complex **2-Ce$^{Ph}$** (59.9 mg, 0.0414 mmol, 1.0 equiv.) in THF (2.0 mL). The reaction mixture was stirred at room temperature for 2 h, resulting in a gray suspension. The suspension was filtered on a porosity 4 glass frit and the volatiles of the yellow filtrate were removed under vacuum. The pale-yellow residue was dried under vacuum for 30 minutes, dissolved in toluene (1.5 mL) and filtered. The volatiles were removed under vacuum and the residue was dissolved in THF (0.2 mL) and $Et_2O$ (2 drops). The resulting solution was stored at -40 °C overnight affording yellow powder of complex **3-Ce$^{Ph}$** (51.4 mg, 85%). X-ray quality crystals were obtained by cooling a concentrated THF solution to -40 °C. $^1$H NMR (500 MHz, THF-$d_8$, 298 K): δ 7.80 ppm (d, $J$ = 7.4 Hz, 12H, -OSiPh$_2$), δ 7.51 ppm (dd, $J$ = 6.9, 2.2 Hz, 3H, tripodal arene), δ 7.25 – 7.20 ppm (m, 6H, tripodal arene and -OSiPh$_3$), δ 7.17 ppm (t, $J$ = 7.4 Hz, 6H, -OSiPh$_2$), δ 7.11 ppm (s, 3H, tripodal arene), δ 7.06 – 7.03 ppm (m, 9H, tripodal arene and -OSiPh$_3$), δ 6.95 ppm (t, $J$ = 7.4 Hz, 12H, -OSiPh$_2$), δ 6.84 ppm (dd, $J$ = 6.7, 2.0 Hz, 3H, tripodal arene), δ 6.79 ppm (t, $J$ = 7.5 Hz, 6H, -OSiPh$_3$)(**Figure S13**). $^{13}$C{$^1$H} NMR (126 MHz, THF-$d_8$, 298 K): δ 150.72 ppm, δ 144.81 ppm, δ 141.02 ppm, δ 139.45 ppm, δ 138.66 ppm, δ 138.00 ppm, δ 135.51 ppm, δ 136.08 ppm, δ 130.53 ppm, δ 129.53 ppm, δ 129.26 ppm, δ 129.21 ppm, δ 128.82 ppm, δ 127.91 ppm, δ 127.57 ppm, δ 125.76 ppm (**Figure S14**). $^{29}$Si{$^1$H} NMR (79.5 MHz, THF-$d_8$, 298 K): δ -24.13 ppm (-OSiPh$_2$ and -OSiPh$_3$)(**Figure S15**). *Anal. Calcd.* for **3-Ce$^{Ph}$**·(THF)$_{2.5}$, $C_{96}H_{96}O_{8.5}Si_4Ce$: C: 70.38; H: 5.91; N: 0.00. *Found*: C: 69.91; H: 5.45; N: 0.00. The fractional THF content is residual co-crystallized solvent left from partial drying.

### S2.2. Synthesis and reactivity of terbium complexes

**Synthesis of [Tb$^{III}$((OSi(O$^t$Bu)$_2$Ar)$_3$-arene)(THF)] (1-Tb$^{OtBu}$):** Following the procedure used for the synthesis of **1-Ce$^{OtBu}$**,[7] a cold (-40 °C) colorless solution of [Tb(N(SiMe$_3$)$_2$)$_3$] (53.4 mg, 0.0834 mmol, 1.0 equiv.) in THF (2 mL) was added to a cold (-40 °C) stirring colorless solution of the (HOSi(O$^t$Bu)$_2$Ar)$_3$-arene) ligand (73.2 mg, 0,0834 mmol, 1.0 equiv.) in THF (0.5 mL) and then warmed to room temperature to stir for 16 h, yielding a colorless solution. The volatiles were then removed under vacuum and the residue was dissolved in toluene (0.3 mL). The resulting solution was stored at -40 °C overnight affording colorless crystals of complex **1-Tb$^{OtBu}$** (61.1 mg, 66%). X-ray quality crystals of **1-Tb$^{OtBu}$** were obtained by cooling a concentrated toluene solution to -40 °C. $^1$H NMR (400 MHz, THF-d$_8$, 298 K): δ 65.47 ppm (s, 3H, tripodal arene), δ 63.00 ppm (br s, 54H, -OSi(O$^t$Bu)$_2$), δ 20.73 ppm (s, 3H, tripodal arene), δ -8.67 ppm (s, 3H, tripodal arene), δ -95.92 ppm (s, 3H, tripodal arene), δ -181.83 ppm (br s, 3H, tripodal arene) (**Figure S17**). *Anal. Calcd.* for **1-Tb$^{OtBu}$**·(toluene)$_{0.7}$, $C_{56.9}H_{82.6}O_{10}Si_3Tb$: C: 58.16; H: 7.10; N: 0.00. *Found*: C: 58.41; H: 7.13; N: 0.00. The fractional toluene content is residual solvent from the bulk synthesis.

**Synthesis of [Tb$^{III}$((OSiPh$_2$Ar)$_3$-arene)(THF)$_3$] (1-Tb$^{Ph}$):** Following the procedure used for the synthesis of **1-Ce$^{Ph}$**, addition of [Tb(N(SiMe$_3$)$_2$)$_3$] (142.8 mg, 0.223 mmol, 1.0 equiv.) in THF (2.0 mL) to the (HOSiPh$_2$Ar)$_3$-arene ligand (201.1 mg, 0.223 mmol, 1.0 equiv.) in THF (2.0 mL) afforded analytically pure **1-Tb$^{Ph}$** as a white powder (232.8 mg, 82%). X-ray quality crystals of **1-Tb$^{Ph}$** were obtained by cooling a concentrated toluene solution at room temperature. $^1$H NMR (400 MHz, THF-d$_8$, 298 K): δ 122.79 ppm (s, 6H, -OSiPh$_2$), δ 97.58 ppm (br s, 6H, -OSiPh$_2$), δ 47.08 ppm (s, 6H, -OSiPh$_2$), δ 40.68 ppm (s, 3H), δ 36.94 ppm (br s, 6H, -OSiPh$_2$), δ 32.10 ppm (s, 3H), δ 24.51 ppm (s, 3H), δ 9.03 ppm (s, 3H), -13.47 ppm (s, 3H), δ -71.14 ppm (s, 3H), δ -180.42 ppm (s, 3H) (**Figure S19**). *Anal. Calcd.* for **1-Tb$^{Ph}$**·(toluene)$_{1.7}$, $C_{83.9}H_{82.6}O_6Si_3Tb$: C: 70.46; H: 5.82; N: 0.00. *Found*: C: 70.28; H: 5.82; N: 0.00. The fractional toluene content is residual co-crystallized solvent.

**Synthesis of [K(THF)$_6$][Tb$^{III}$((OSi(O$^t$Bu)$_2$Ar)$_3$-arene)(OSiPh$_3$)] (2-Tb$^{OtBu}$):** Following the procedure used for the synthesis of **2-Ce$^{OtBu}$**, addition of KOSiPh$_3$ (21.7 mg, 0.069 mmol, 1.15 equiv.) in THF (1 mL) to **1-Tb$^{OtBu}$** (61.9 mg, 0.060 mmol, 1.0 equiv.) in THF (1.0 mL) afforded analytically pure **2-Tb$^{OtBu}$** as a white powder (66.5 mg, 82%). X-ray quality crystals of **2-Tb$^{OtBu}$** were obtained by cooling a concentrated THF solution to -40 °C. $^1$H NMR (400 MHz, THF-d$_8$, 298

K): δ 30.37-26.77 ppm (br m, -OSi(O$^t$Bu)$_2$), δ 9.55 ppm (br s), δ -0.74 ppm (s), -1.29 ppm (br s), -2.17 ppm (s), -30.24 ppm (br s), -44.40 ppm (br s), -77.15 ppm (br, s) (**Figure S21**). *Anal. Calcd.* for [K(THF)$_{4.5}$][Tb$^{III}$((OSi(O$^t$Bu)$_2$Ar)$_3$-arene)(OSiPh$_3$)], $C_{84}H_{120}KO_{14.5}Si_4Tb$: C: 60.33; H: 7.23; N: 0.00. *Found*: C: 60.04; H: 7.46; N: 0.00. The potassium bound THF was lost during the drying process.

**Synthesis of [K(toluene){Tb$^{III}$((OSiPh$_2$Ar)$_3$-arene)(OSiPh$_3$)}](2-Tb$^{Ph}$):** Following the procedure used for the synthesis of **2-Ce$^{Ph}$**, the addition of KOSiPh$_3$ (54.2 mg, 0.172 mmol, 1.15 equiv.) in THF (1.0 mL) to **1-Tb$^{Ph}$** (191.0 mg, 0.150 mmol, 1.0 equiv.) in THF (2.0 mL) afforded analytically pure **2-Tb$^{Ph}$** as a white powder (138.5 mg, 67%). X-ray quality crystals of **2-Tb$^{Ph}$** were obtained by cooling a concentrated toluene solution to -40 °C. $^1$H NMR (400 MHz, THF-d$_8$, 298 K): δ 40.77 ppm (br s), δ 26.54 ppm (br s), 17.60 ppm (s), δ 16.59 ppm (s), 13.38 ppm (s), δ 10.33 ppm (s), 6.37 ppm (s), -0.59 ppm (s), -20.46 ppm (br s), -47.24 (br s) (**Figure S23**). *Anal. Calcd.* for [K{Tb$^{III}$((OSiPh$_2$Ar)$_3$-arene)(OSiPh$_3$)}], $C_{78}H_{60}KO_4Si_4Tb$: C: 68.30; H: 4.41; N: 0.00. *Found*: C: 67.87; H: 4.89; N: 0.00. The potassium bound toluene was lost during the drying process.
Better quality crystals for X-ray diffraction analysis were also obtained by gas phase diffusion of $Et_2O$ on a concentrated MeCN solution of [K(MeCN){Tb$^{III}$((OSiPh$_2$Ar)$_3$-arene)(OSiPh$_3$)(MeCN)}]$_2$, **2-Tb$^{Ph}$-MeCN** at -40 °C (**Figure S54**).

**Reaction of complex 2-Tb$^{Ph}$ with 1.1 equiv. of 2.2.2-cryptand in MeCN:** A 2.2.2-cryptand (3.7 mg, 0.01 mmol, 1.0 equiv.) solution in MeCN-*d*$_3$ (0.1 mL) was added to a colorless solution of **2-Tb$^{Ph}$** (14.6 mg, 0.01 mmol, 1.0 equiv.) in MeCN-*d*$_3$ (0.4 mL). The $^1$H NMR spectrum of the resulting solution showed the appearance of a new set of resonances, indicating the $K^+$ ion was bound in the **2-Tb$^{Ph}$** in MeCN (**Figure S24**).

**Synthesis [Tb$^{IV}$((OSiPh$_2$Ar)$_3$-arene)(OSiPh$_3$)(MeCN)$_2$] (3-Tb$^{Ph}$):** A large reaction tube equipped with a magnetic stirbar was charged with **2-Tb$^{Ph}$** (81.9 mg, 0.056 mmol, 1.0 equiv.) and 2.2.2-cryptand (21.1 mg, 0.056 mmol, 1.0 equiv.) and dissolved in 0.2 mL of MeCN. A separate 6 mL vial was charged with [N($C_6H_4Br$)][$SbCl_6$] (50.6 mg, 0.062 mmol, 1.1 equiv.) and suspended in 0.9 mL MeCN. Both vials were chilled to -40 °C. The chilled solution of [N($C_6H_4Br$)][$SbCl_6$] was added to the vial containing **2-Tb$^{Ph}$** and 2.2.2-cryptand, where upon addition, an orange solid crashed out. After 15 minutes stirring at -40 °C, the resulting orange solid was warmed to room temperature, then filtered and collected over a porosity 4 filter-frit, in which the solid was further washed with minimal MeCN (4 mL). The orange solid was collected and dried on high vacuum for 1 hour, then dissolved in toluene, resulting in a dark orange supernatant with precipitation of colorless solids. The colorless solids were removed by filtering the solution over a 0.22 μm porosity filter frit. The volatiles were removed under vacuum yielding a dark orange micro-crystalline solid of analytically pure complex **3-Tb$^{Ph}$** (41.8 mg, 53% yield). **Note**: the reaction can also be performed in the absence of 2.2.2-cryptand, however, the yield of **3-Tb$^{Ph}$** was slightly lower (43% yield). $^1$H NMR (400 MHz, toluene-*d*$_8$, 298 K): silent due to the 4f$^7$ electronic configuration of the Tb(IV) ion (**Figure S27**). UV/Vis: $\lambda_{max}$ = 285 and 355 nm. *Anal. Calcd.* for **3-Tb$^{Ph}$**, $C_{82}H_{66}N_2O_4Si_4Tb$: C: 69.62; H: 4.70; N: 1.98. *Found*: C: 69.45; H: 4.97; N: 1.78.
Single crystals suitable for XRD analysis were obtained from a similar, dilute small-scale reaction mixture: A 6 mL vial was charged with **2-Tb$^{Ph}$** (14.6 mg, 0.01 mmol, 1.0 equiv.) and 2.2.2-cryptand (3.7 mg, 0.01 mmol, 1.0 equiv.) and dissolved in 0.5 mL of MeCN. A separate 6 mL vial was charged with [N($C_6H_4Br$)][$SbCl_6$] (8.2 mg, 0.01 mmol, 1.0 equiv.) and dissolved in 1.0 mL MeCN. Both vials were chilled to -40 °C. The chilled solution of [N($C_6H_4Br$)][$SbCl_6$] was added to the vial containing **2-Tb$^{Ph}$**, where upon addition, the solution turned dark orange in color. The solution was allowed to stand at -40 °C for 24 hours resulting in a mixture of dark orange and pale-yellow crystals identified as complexes, **2-Tb$^{Ph}$** and the MeCN solvate analogue of **1-Tb$^{Ph}$**, **1-Tb$^{Ph}$-CH$_3$CN** (**Figure S50**), respectively. Complex **2-Tb$^{Ph}$** was the major product from this reaction mixture; however, isolation of analytically pure material through this pathway proved unsuccessful due to inability to separate the **1-Tb$^{Ph}$-CH$_3$CN** complex.

**Reaction of complex 1-Tb$^{Ph}$ with 1.0 equiv. of KOSi(O$^t$Bu)$_3$ in THF:** A KOSi(O$^t$Bu)$_3$ (2.4 mg, 0.0079 mmol, 1.0 equiv.) solution in THF-*d*$_8$ (0.25 mL) was added to a colorless solution of **1-Tb$^{Ph}$** (10.0 mg, 0.0079 mmol, 1.0 equiv.) in THF-*d*$_8$ (0.25 mL). The $^1$H NMR spectrum of the resulting solution showed the appearance of a new set of resonances, indicating the KOSi(O$^t$Bu)$_3$ was bound to the **1-Tb$^{Ph}$** complex in THF (**Figure S35**).

**Reaction of complex 1-Tb$^{Ph}$ with 1.1 equiv. of KOSiMe$_3$ in THF:** A KOSiMe$_3$ (2.7 mg, 0.021 mmol, 1.1 equiv.) solution in THF-*d*$_8$ (0.25 mL) was added to a colorless solution of **1-Tb$^{Ph}$** (24.0 mg, 0.0188 mmol, 1.0 equiv.) in THF-*d*$_8$ (0.25 mL).

The $^{1}$H NMR spectrum of the resulting solution showed the appearance of a new set of resonances, indicating the $KOSiMe_3$ was bound to the **1-Tb$^{Ph}$** complex in THF (**Figure S36)**.

**Reaction of complex 1-Tb$^{Ph}$ with 1.1 equiv. of 2-KOAd in THF:** A 2-KOAd (3.2 mg, 0.017 mmol, 1.1 equiv.) solution in THF-*d*$_8$ (0.25 mL) was added to a colorless solution of **1-Tb$^{Ph}$** (19.4 mg, 0.0152 mmol, 1.0 equiv.) in THF-*d*$_8$ (0.25 mL). The $^{1}$H NMR spectrum of the resulting solution showed the appearance of a new set of resonances, indicating the 2-KOAd was bound to the **1-Tb$^{Ph}$** complex in THF (**Figure S37**).

**Reaction of complex 1-Tb$^{Ph}$ with 1.0 equiv. of $K(N(SiMe_3)_2)$ in THF:** A $K(N(SiMe_3)_2)$ (1.8 mg, 0.009 mmol, 1.1 equiv.) solution in THF-*d*$_8$ (0.25 mL) was added to a colorless solution of **1-Tb$^{Ph}$** (10.4 mg, 0.0082 mmol, 1.0 equiv.) in THF-*d*$_8$ (0.25 mL). The $^{1}$H NMR spectrum of the resulting solution showed the appearance of a new set of resonances, indicating the $K(N(SiMe_3)_2)$ was bound to the **1-Tb$^{Ph}$** complex in THF (**Figure S38**).

**Reaction of complex 1-Tb$^{Ph}$ with 1.1 equiv. of NaO$^t$Bu in THF:** A NaO$^t$Bu (1.7 mg, 0.0177 mmol, 1.7 equiv.) solution in THF-*d*$_8$ (0.25 mL) was added to a colorless solution of **1-Tb$^{Ph}$** (14.7 mg, 0.0115 mmol, 1.0 equiv.) in THF-*d*$_8$ (0.25 mL). The $^{1}$H NMR spectrum of the resulting solution showed the appearance of a new set of resonances, indicating the NaO$^t$Bu was bound to the **1-Tb$^{Ph}$** complex in THF (**Figure S39**).

### S2.3. Synthesis and reactivity of praseodymium complexes

**Synthesis of $[Pr^{III}((OSiPh_2Ar)_3\text{-arene})(THF)_3]$ (1-Pr$^{Ph}$):** Following the procedure used for the synthesis of **1-Ce$^{Ph}$**, addition of $[Pr(N(SiMe_3)_2)_3]$ (39.3 mg, 0.063 mmol, 1.0 equiv.) in THF (2.0 mL) to the $(HOSiPh_2Ar)_3$-arene ligand (57.0 mg, 0.063 mmol, 1.0 equiv.) in THF (0.5 mL) afforded analytically pure **1-Pr$^{Ph}$** as a white powder (57.8 mg, 73%). X-ray quality crystals of **1-Pr$^{Ph}$** were obtained by cooling a concentrated toluene solution at room temperature. $^{1}$H NMR (400 MHz, THF-d$_8$, 298 K): δ 18.50 ppm (br s, 6H, $-OSiPh_2$), δ 17.46 ppm (br s, 6H, $-OSiPh_2$), δ 11.12 ppm (s, 3H), δ 10.54 ppm (br s, 6H, $-OSiPh_2$), δ 9.24 ppm (br s, 6H, $-OSiPh_2$), δ 7.46 ppm (s, 3H), δ 5.02 ppm (s, 3H), δ -1.27 ppm (s, 3H), -12.10 ppm (s, 3H) (**Figure S31**). *Anal. Calcd.* for **1-Pr$^{Ph}$•**(toluene), $C_{79}H_{77}O_6Si_3Pr$: C: 70.41; H: 5.76; N: 0.00. *Found*: C: 70.33; H: 5.73; N: 0.00. The fractional toluene content is residual co-crystallized solvent left from partial drying.

**Synthesis of $[K(toluene)\{Pr^{III}((OSiPh_2Ar)_3\text{-arene})(OSiPh_3)\}]$: 2-Pr$^{Ph}$** Following the procedure used for the synthesis of **2-Ce$^{Ph}$**, the addition of $KOSiPh_3$ (40.3 mg, 0.128 mmol, 1.15 equiv.) in THF (1.0 mL) to **1-Pr$^{Ph}$** (139.4 mg, 0.111 mmol, 1.0 equiv.) in THF (2.0 mL) afforded ,after recrystallization from toluene, analytically pure **2-Pr$^{Ph}$** as a white powder (128.5 mg, 86%). *Anal. Calcd.* for $[K\{Pr^{III}((OSiPh_2Ar)_3\text{-arene})(OSiPh_3)\}]$, $C_{78}H_{60}KO_4Si_4Pr$: C: 69.21; H: 4.47; N: 0.00. *Found*: C: 69.02; H: 4.74; N: 0.00. The potassium bound toluene was lost during the drying process.
Crystals of complex **2-Pr$^{Ph}$ •0.5 toluene** (isostructural to **2-Tb$^{Ph}$ •0.5 toluene**) could be obtained from toluene but their quality was not sufficient for a publishable structural determination, but X-ray quality crystals of $[K(MeCN)_2\{Pr^{III}((OSiPh_2Ar)_3\text{-arene})(OSiPh_3)(MeCN\}]$•4MeCN, **2-Pr$^{Ph}$-MeCN•4MeCN** were obtained by gas phase diffusion of $Et_2O$ on a concentrated MeCN solution of **2-Pr$^{Ph}$** at -40 °C. $^{1}$H NMR (400 MHz, THF-d$_8$, 298 K): δ 16.49 ppm (br s), 15.97 ppm (br s), 10.31 ppm (s), δ 9.08 ppm (s), δ 8.55 ppm (s), 8.32 ppm (s), 8.22 ppm (s), δ 5.48 ppm (s), 0.96 ppm (br s), -3.73 ppm (br s) (**Figure S33**).

**Reaction of complex 2-Pr$^{Ph}$ with 1.1 equiv. of 2.2.2-cryptand in MeCN:** A 2.2.2-cryptand (3.5 mg, 0.0093 mmol, 1.1 equiv.) solution in MeCN-*d*$_3$ (0.1 mL) was added to a colorless solution of **2-Pr$^{Ph}$** (11.4 mg, 0.0084 mmol, 1.0 equiv.) in MeCN-*d*$_3$ (0.4 mL). The $^{1}$H NMR spectrum of the resulting solution showed the appearance of a new set of resonances, indicating the $K^+$ ion was bound in the **2-Pr$^{Ph}$** in MeCN (**Figure S34**).

**Reaction of complex 2-Pr$^{Ph}$ with 1.1 equiv. of $[N(C_6H_4Br)_3][SbCl_6]$ in MeCN:** A cold (-40 °C) solution of $[N(C_6H_4Br)][SbCl_6]$ (6.7 mg, 0.0082 mmol, 1.05 equiv.) in MeCN-*d*$_3$ (0.2 mL) was added to a cold (-40 °C) suspension of **2-Pr$^{Ph}$** (10.6 mg, 0.0078 mmol, 1.0 equiv.) in MeCN-*d*$_3$ (0.4 mL). The reaction mixture immediately turned dark brown-orange but that color faded to yellow in less than 2 minutes while a white solid started to precipitate and then evolved to colorless after 10 minutes -40 °C. Colorless crystals that grew from this reaction mixture were identified as the MeCN adduct of complex **1-Pr$^{Ph}$** (**Figure S53**)

**Reaction of complex 1-Pr$^{Ph}$ with 1.1 equiv. of KOSiMe$_3$ in THF:** A KOSiMe$_3$ (4.2 mg, 0.0327 mmol, 1.1 equiv.) solution in THF-$d_8$ (0.25 mL) was added to a colorless solution of **1-Pr$^{Ph}$** (37.7 mg, 0.030 mmol, 1.0 equiv.) in THF-$d_8$ (0.25 mL). The $^1$H NMR spectrum of the resulting solution showed the appearance of a new set of resonances, indicating the KOSiMe$_3$was bound to the **1-Pr$^{Ph}$** complex in THF (**Figure S40**).

**Reaction of complex 1-Pr$^{Ph}$ with 1.1 equiv. of 2-KOAd in THF:** A 2-KOAd (2.7 mg, 0.014 mmol, 1.1 equiv.) solution in THF-$d_8$ (0.25 mL) was added to a colorless solution of **1-Pr$^{Ph}$** (16.0 mg, 0.0127 mmol, 1.0 equiv.) in THF-$d_8$ (0.25 mL). The $^1$H NMR spectrum of the resulting solution showed the appearance of a new set of resonances, indicating the 2-KOAd was bound to the **1-Pr$^{Ph}$** complex in THF (**Figure S41**).

### S2.4. Separation experiments

**Oxidation with [N(C$_6$H$_4$Br)][SbCl$_6$] of the in situ prepared 1:1 mixture of 2-Tb $^{Ph}$** and **2-Dy$^{Ph}$:** Following the procedure used for the synthesis of **1-Tb$^{Ph}$**, a solution of [Tb(N(SiMe$_3$)$_2$)$_3$] (45.0 mg, 0.0703 mmol, 1.0 equiv.) and [Dy(N(SiMe$_3$)$_2$)$_3$] (45.2 mg, 0.0703 mmol, 1.0 equiv.) in toluene (2.0 mL) was added to a solution of the (HOSiPh$_2$Ar)$_3$-arene ligand (126.7 mg, 0.1406 mmol, 2.0 equiv.) in toluene (1.0 mL) affording the desired 1:1 mixture of **1Tb $^{Ph}$** and **1-Dy$^{Ph}$** as a white suspension. The suspension was taken to dryness and the residue was washed with hexane to remove the HN(SiMe$_3$)$_2$ byproduct. The resulting residue was suspended in toluene (1.0 mL) and a KOSiPh$_3$ (44.3 mg, 0.141 mmol, 1.0 equiv.) solution in toluene (1.0 mL) was added to the white suspension. The resulting solution was stirred for 30 minutes at room temperature affording a pale-yellow solution. The volatiles were removed under vacuum and the resulting solid was further dried for 2 h to obtain a 1:1 mixture of **2-Tb$^{Ph}$** :**2-Dy$^{Ph}$** (**Figure S43**).

The residue was transferred to a 6 mL vial equipped with a magnetic stir-bar and 2.2.2-cryptand (52.9 mg, 0.141mmol, 1.0 equiv.) and dissolved in 0.3 mL of MeCN. [N(C$_6$H$_4$Br)][SbCl$_6$] (126.6 mg, 0.155 mmol, 1.1 equiv.) was placed in a separate 6 mL vial and suspended in 1.5 mL MeCN. Both vials were chilled at -40 °C for 5 mins. The chilled solution of [N(C$_6$H$_4$Br)][SbCl$_6$] was added to the vial containing the 1:1 mixture of **2-Tb$^{Ph}$** and **2-Dy$^{Ph}$** and 2.2.2-cryptand, where upon addition, an orange solid crashed out. After 25 minutes stirring at -40 °C, the resulting orange solid was filtered and collected over a porosity 4 filter-frit, in which the solid was further washed with minimal MeCN (2 mL). The orange solid was collected and dried on high vacuum for 30 mins, then dissolved in toluene, resulting in a dark orange supernatant and colorless solids. The colorless solids were removed by filtering the solution over a 0.22 μm porosity filter frit. After dissolution in THF the colorless solid shows mainly the presence of **1-Dy$^{Ph}$** .
The volatiles were removed from the dark orange supernatant under vacuum yielding a dark orange micro-crystalline solid (36.0 mg) which contains only 6% **1-Dy$^{Ph}$** as measured by quantitative $^1$H NMR in THF using $CH_2Cl_2$ as a standard. The $^1$H NMR (400 MHz, 298 K) in toluene-$d_8$ of the solid obtained is silent (**Figure S44**) due to presence of Tb(IV), **2-Tb $^{Ph}$** as major species. After dissolution in THF of obtained solid decomposition of the Tb(IV) occurs to yield a mixture of **1-Tb $^{Ph}$** and **1-Dy$^{Ph}$** .

## S3. NMR Spectroscopic Data

### S3.1. NMR spectra for cerium complexes

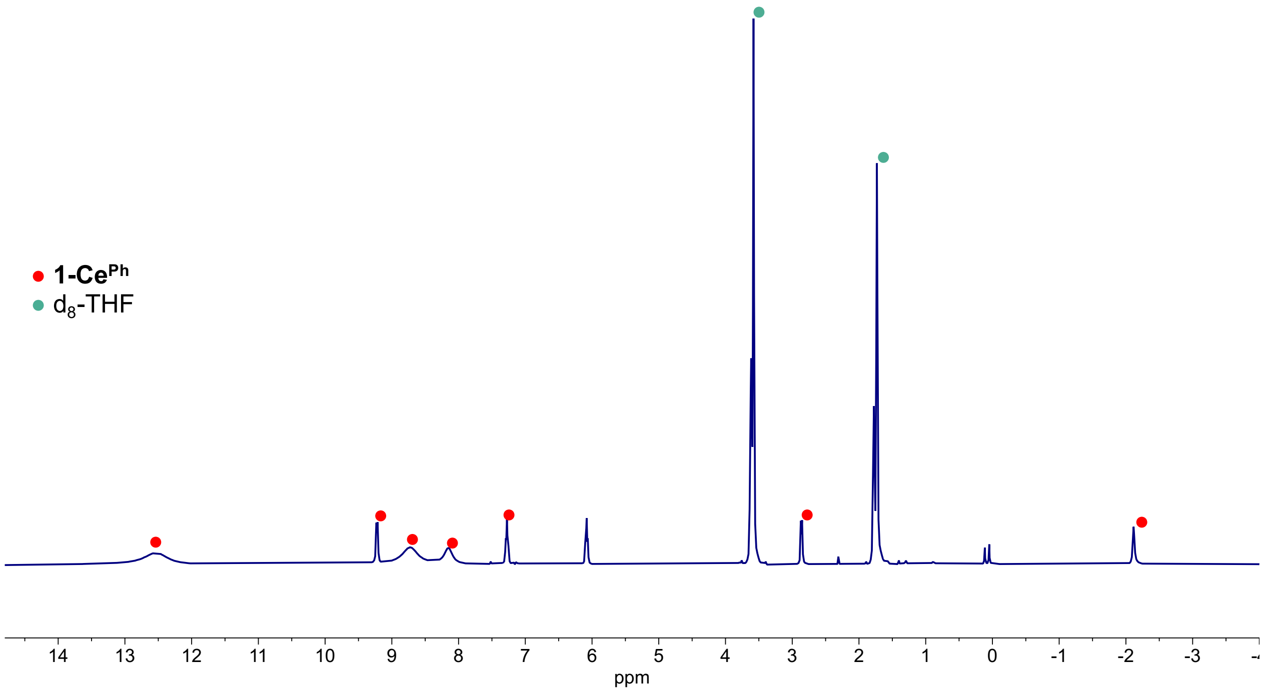


**Figure S1**. $^1H$ NMR spectrum (400 MHz, THF-$d_8$, 298 K) of the reaction mixture obtained after addition of 1.0 equiv. of $[Ce(N(SiMe_3)_2)_3]$ to $(HOSiPh_2Ar)_3$-arene after 16 h, resulting in complex **1-Ce$^{Ph}$**.

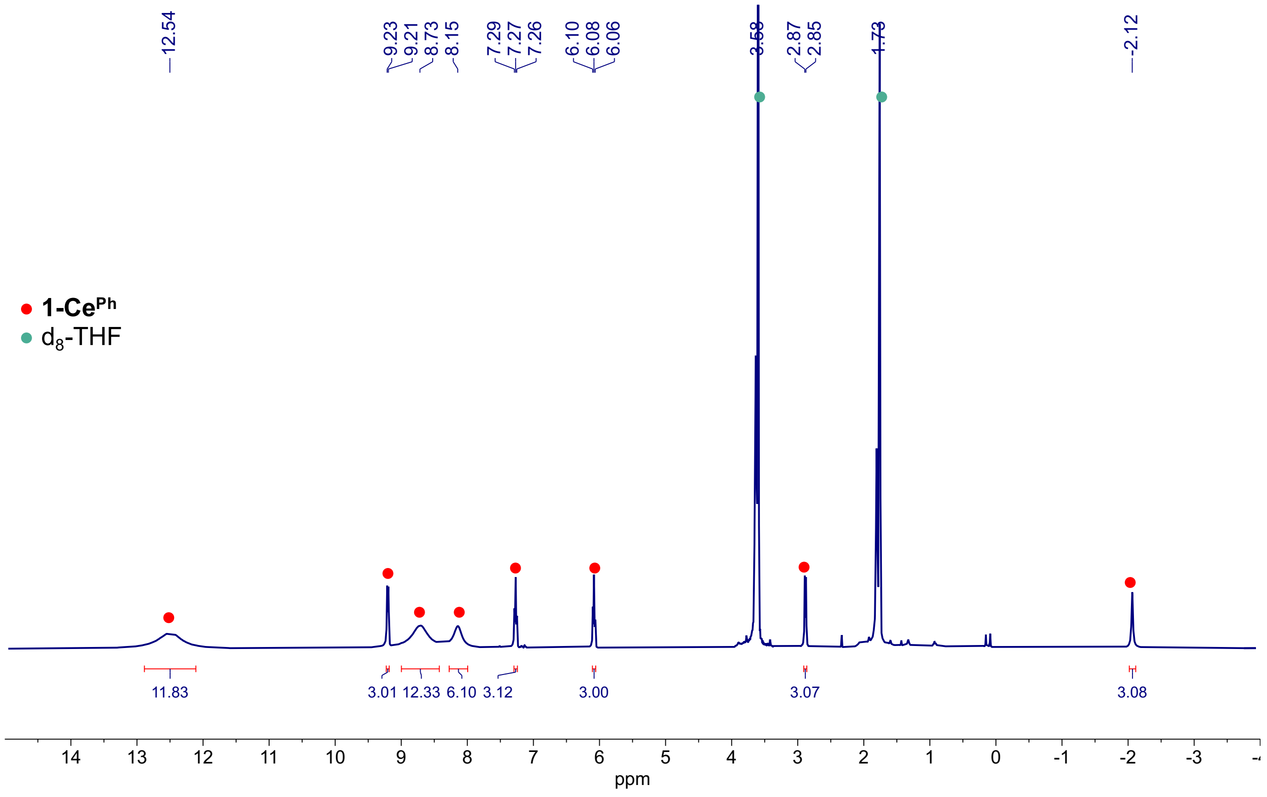


**Figure S2**. $^{1}$H NMR spectrum (400 MHz, THF-*d*$_8$, 298 K) of isolated [Ce((OSiPh$_2$Ar)$_3$-arene)(THF)$_3$], **1-Ce$^{Ph}$**.

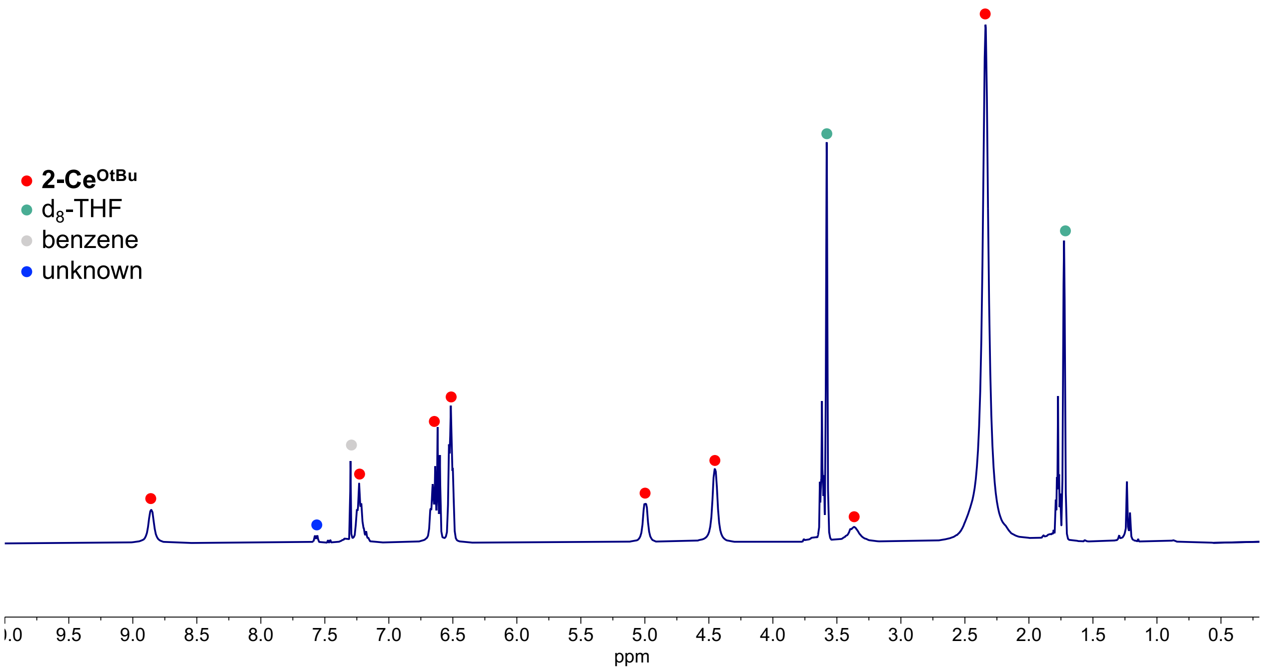


**Figure S3**. $^{1}H$ NMR spectrum (400 MHz, THF-$d_8$, 298 K) of the reaction mixture obtained after addition of 1.0 equiv. of $KOSiPh_3$ to [Ce((OSi(O$^t$Bu)$_2$)$_3$-arene)(THF)$_3$], **1-Ce$^{OtBu}$** after 30 mins, resulting in **2-Ce$^{OtBu}$**.

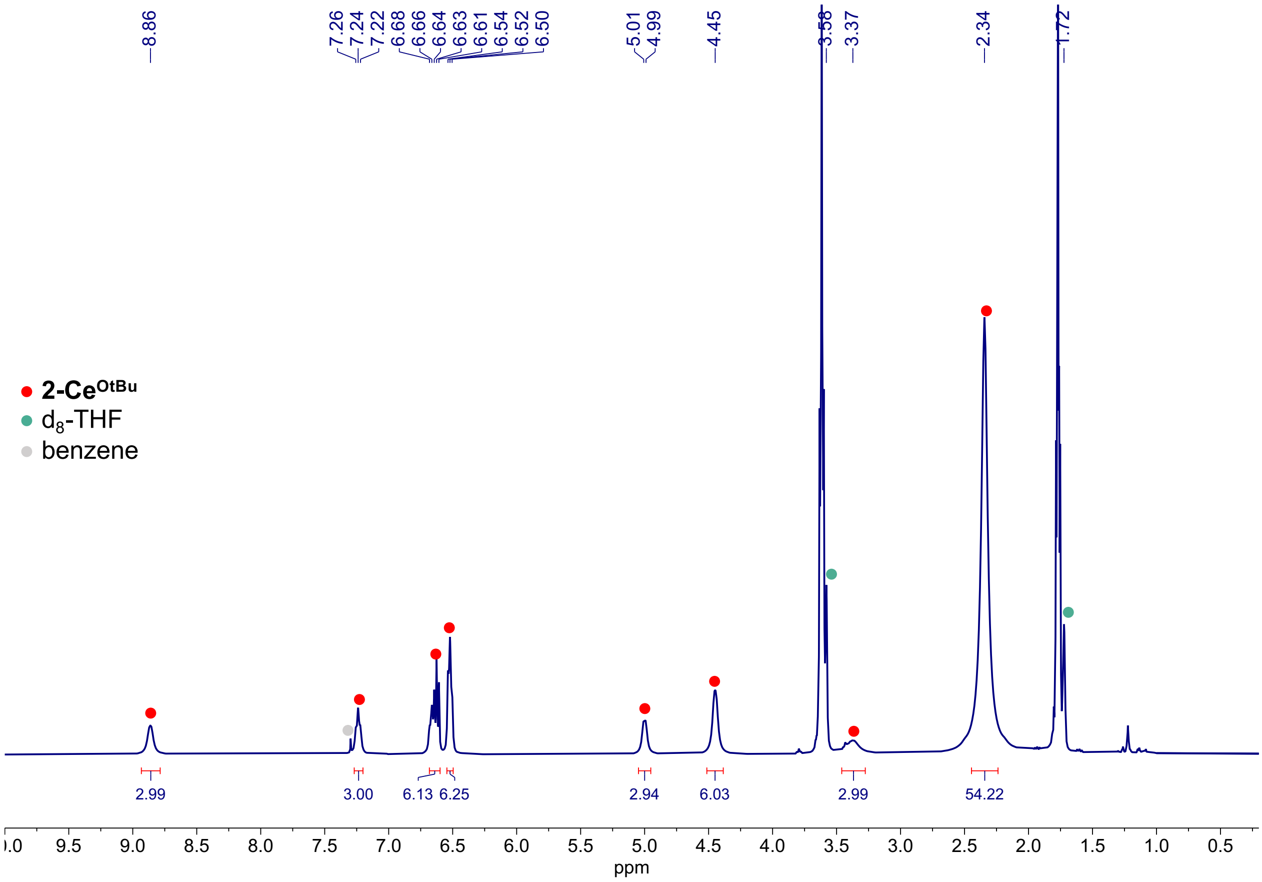


**Figure S4**. $^{1}$H NMR spectrum (400 MHz, THF-$d_8$, 298 K) of isolated [Ce((OSiPh$_2$Ar)$_3$-arene)(THF)$_3$], **2-Ce$^{\mathbf{OtBu}}$**.

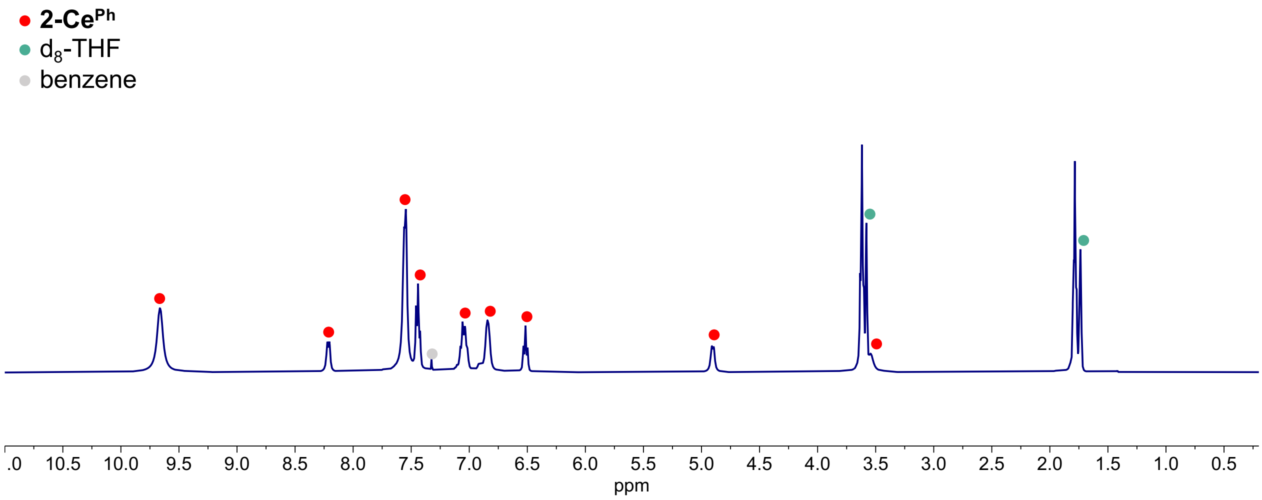


**Figure S5**. $^{1}$H NMR spectrum (400 MHz, THF-$d_8$, 298 K) of the reaction mixture obtained after addition of 1.0 equiv. of $KOSiPh_3$ to $[Ce((OSiPh_2Ar)_3\text{-arene})(THF)_3]$, **1-Ce$^{Ph}$** after 30 mins, resulting in **2-Ce$^{Ph}$**.

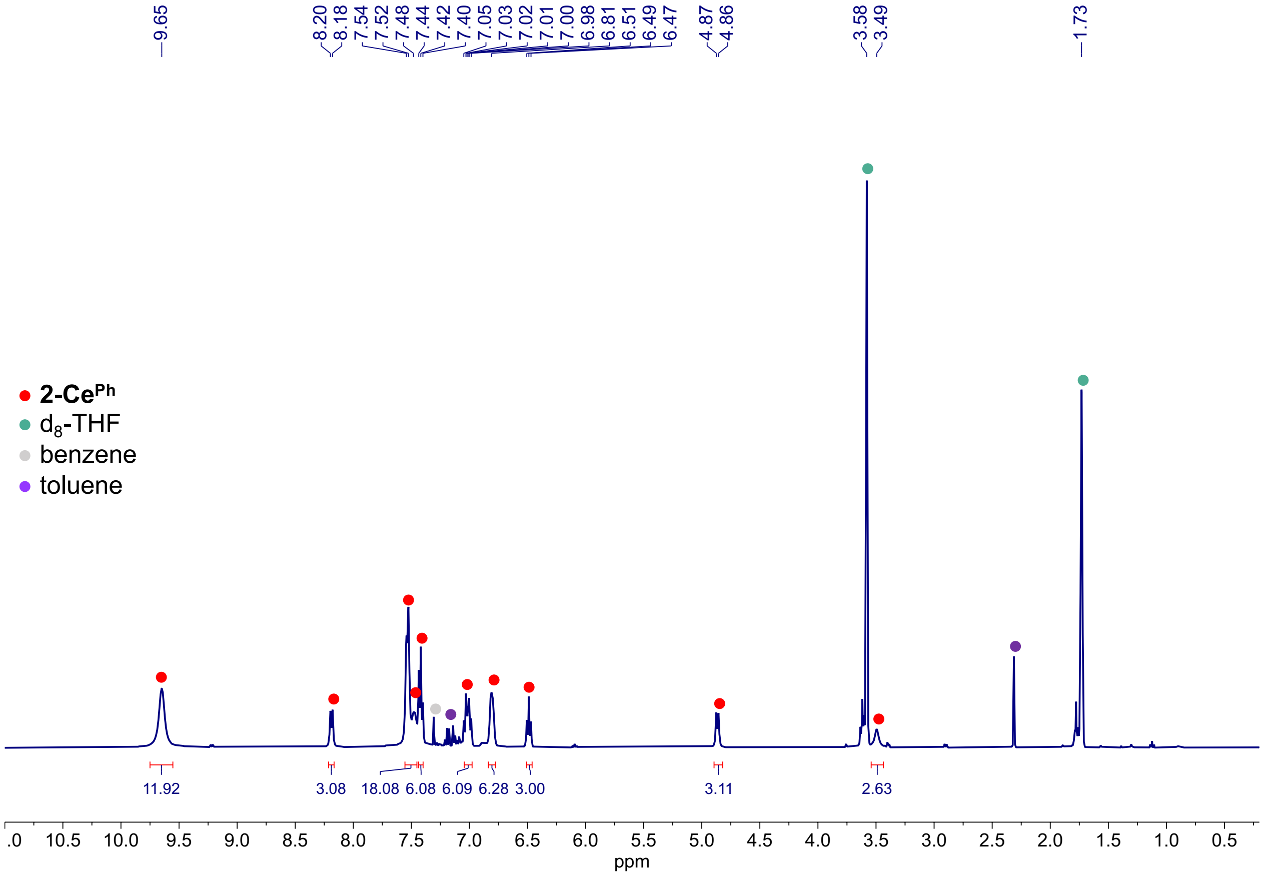


**Figure S6**. $^{1}$H NMR spectrum (400 MHz, THF-*d*8, 298 K) of isolated [K{Ce((OSiPh2Ar)3-arene)(OSiPh3)(toluene)}], **2-Ce$^{Ph}$**.

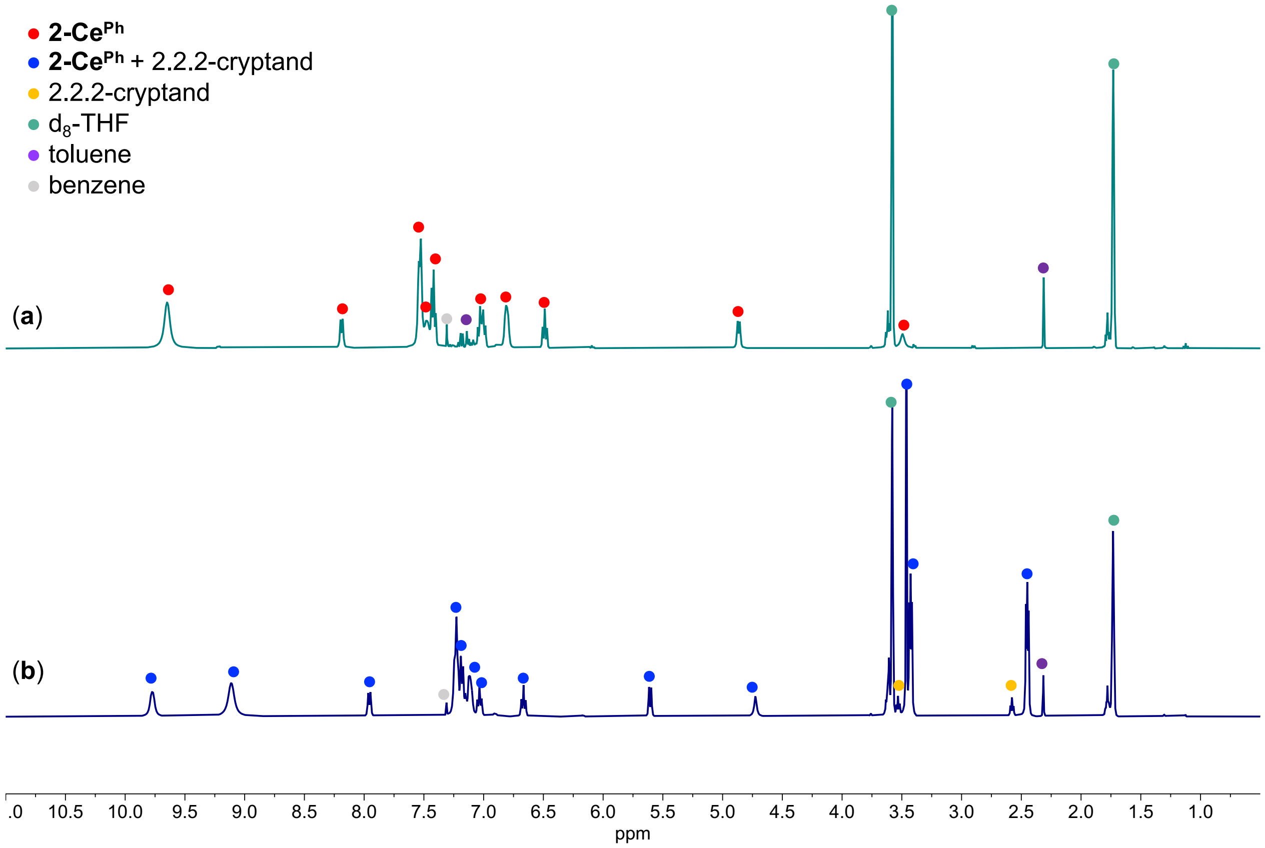


**Figure S7**. $^{1}$H NMR spectra (400 MHz, THF-$d_8$, 298 K) of the reaction mixture obtained after addition of 1.1 equiv. of 2.2.2-cryptand: **2-Ce$^{Ph}$** before a) and immediately after b) addition of 1.1 equiv. of 2.2.2-cryptand.

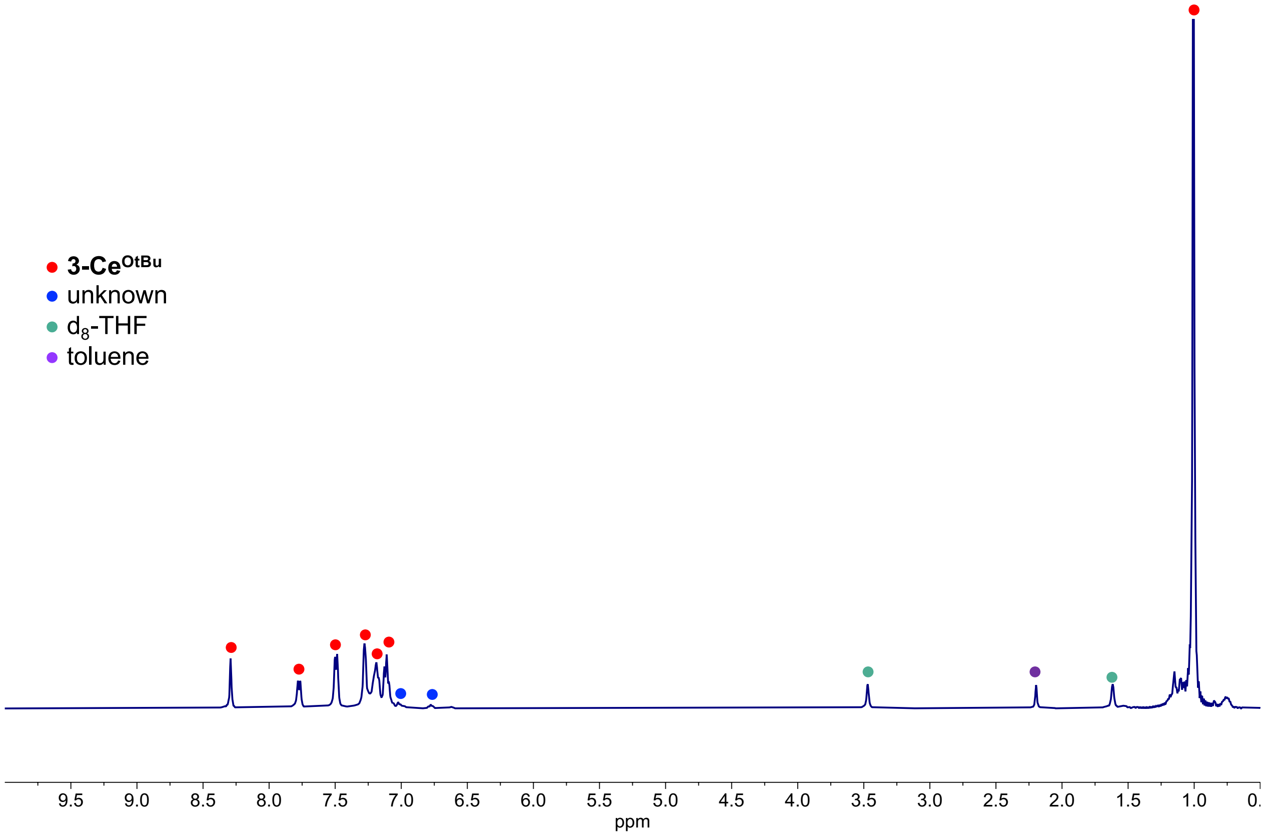


**Figure S8**. $^{1}$H NMR spectrum (400 MHz, THF-$d_8$, 298 K) of the reaction mixture obtained after addition of 1.1 equiv. of $AgBPh_4$ to **2-Ce$^{OtBu}$** after 2 hours.

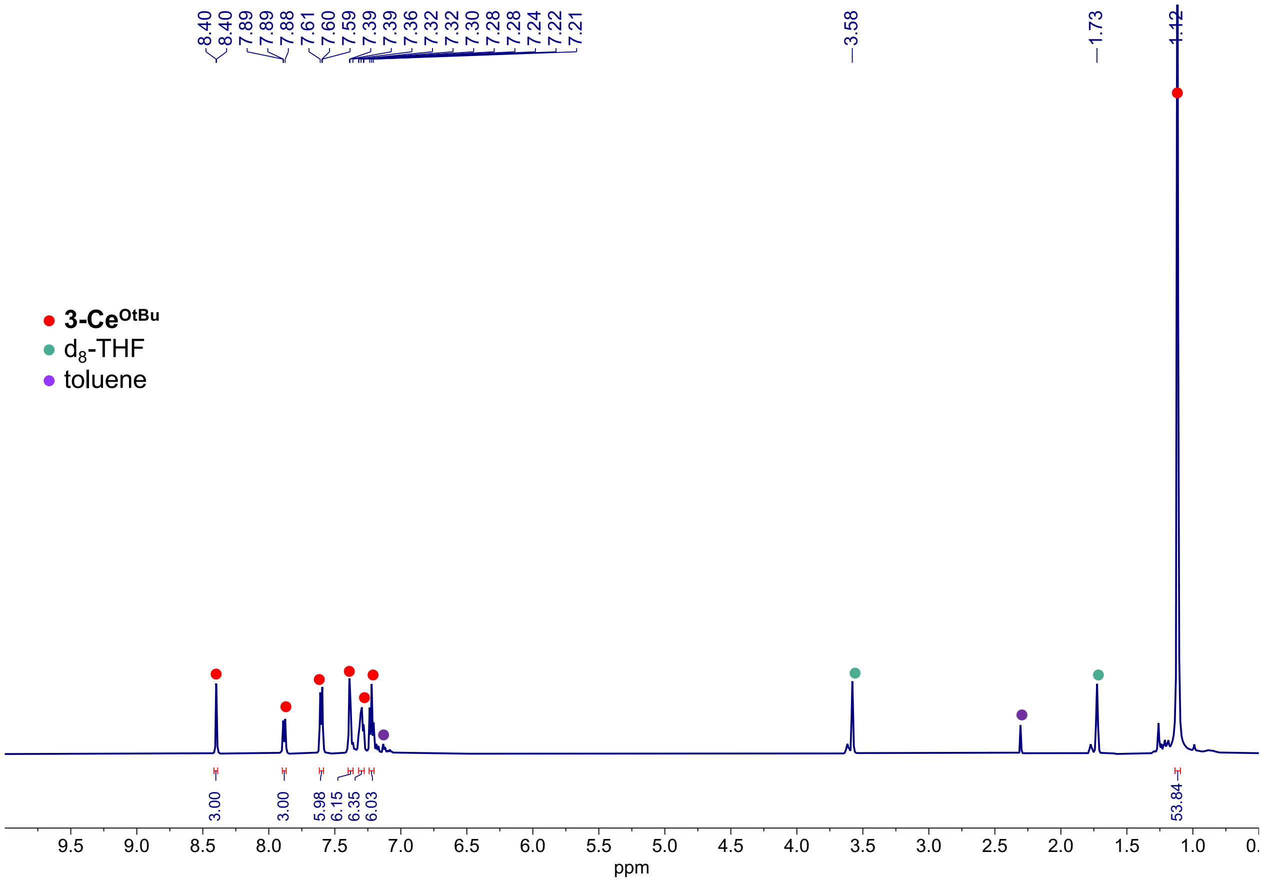


**Figure S9**. $^{1}$H NMR spectrum (500 MHz, THF-$d_8$, 298 K) of isolated [Ce((OSi(O$^t$Bu)$_2$Ar)$_3$-arene)(OSiPh$_3$)], **3-Ce$^{OtBu}$**.

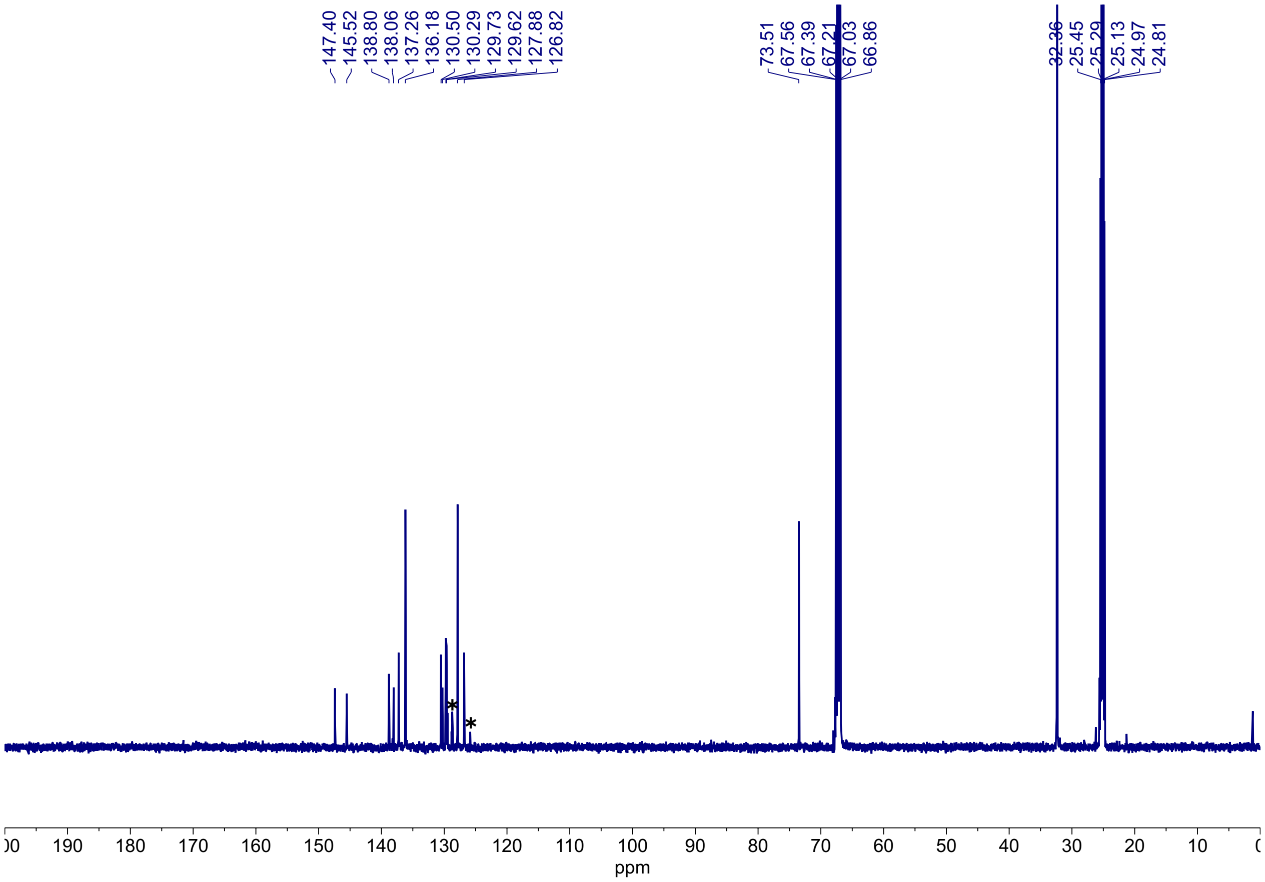


**Figure S10**. $^{13}C\{^{1}H\}$ NMR spectrum (126 MHz, THF-$d_8$, 298 K) of isolated [Ce((OSi(O$^t$Bu)$_2$Ar)$_3$-arene)(OSiPh$_3$)], **3-Ce$^{OtBu}$** (✱toluene).

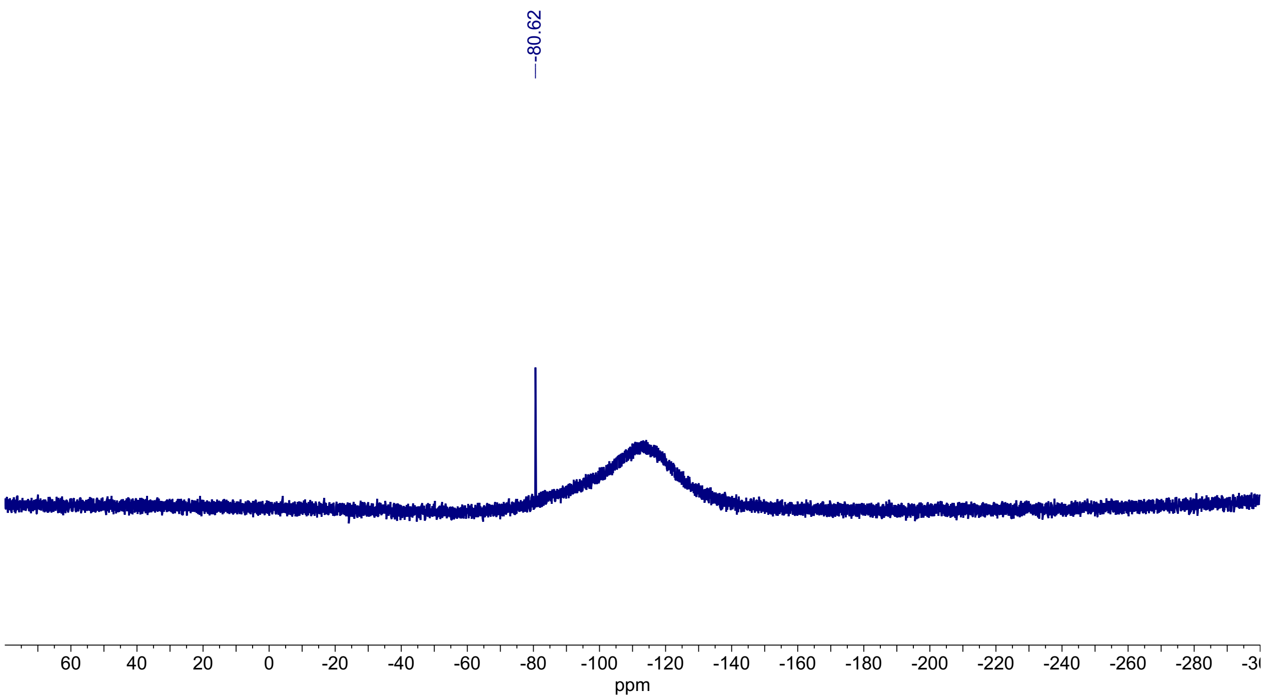


**Figure S11**. $^{29}Si\{^{1}H\}$ NMR spectrum (79.5 MHz, THF-$d_8$, 298 K) of isolated [Ce((OSi(O$^t$Bu)$_2$Ar)$_3$-arene)(OSiPh$_3$)], **3-Ce$^{OtBu}$**.

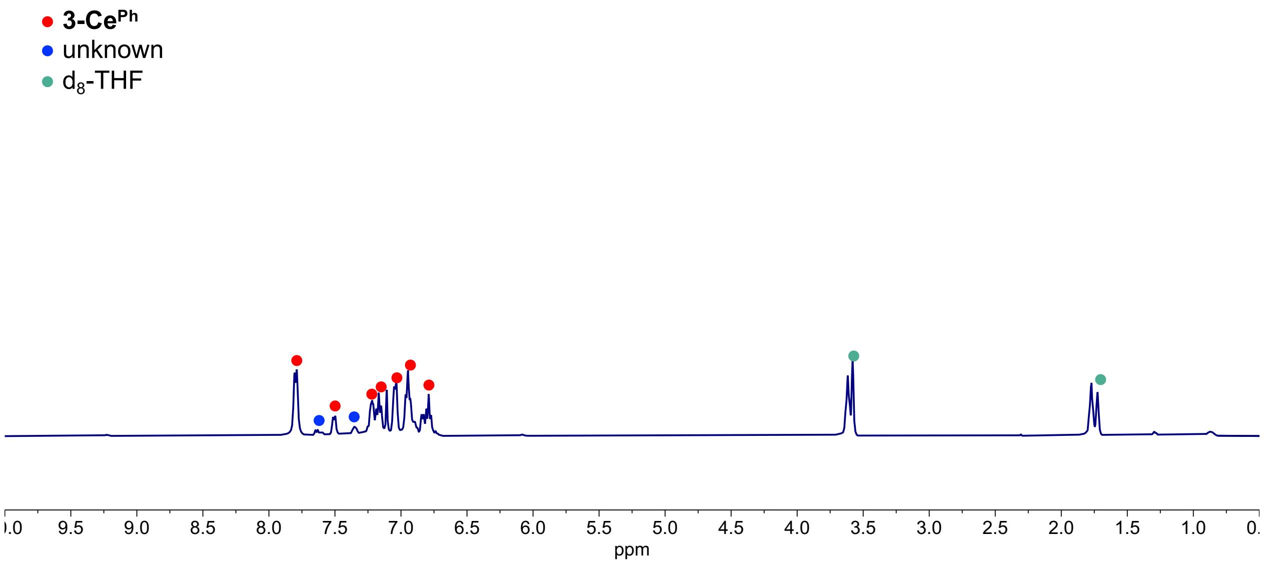


**Figure S12**. $^1$H NMR spectrum (400 MHz, THF-*d*$_8$, 298 K) of the reaction mixture obtained after addition of 1.1 equiv. of $AgBPh_4$ to **2-Ce$^{Ph}$** after 2 hours.

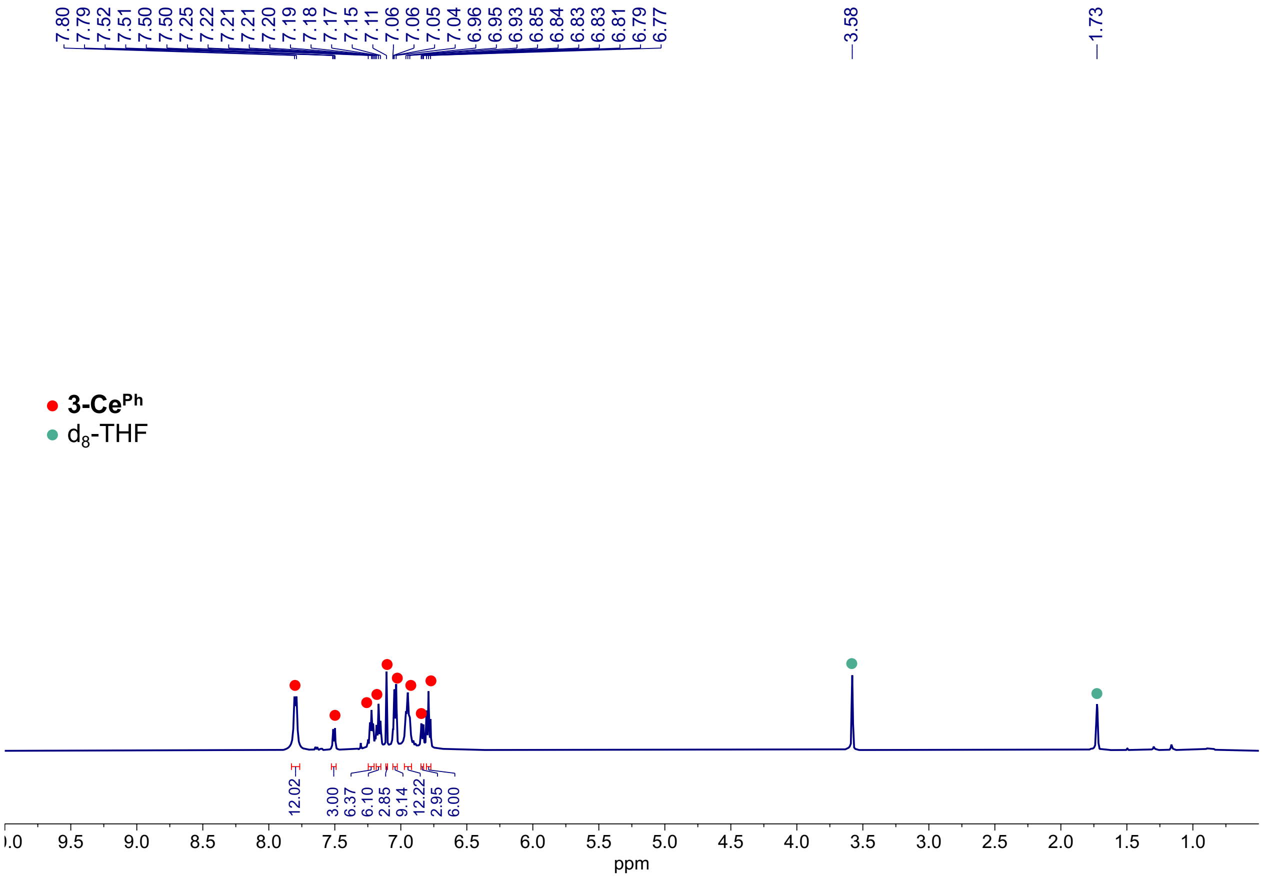


**Figure S13**. $^{1}$H NMR spectrum (500 MHz, THF-$d_8$, 298 K) of isolated [Ce((OSiPh$_2$Ar)$_3$-arene)(OSiPh$_3$)(THF)$_2$], **3-Ce$^{Ph}$**.

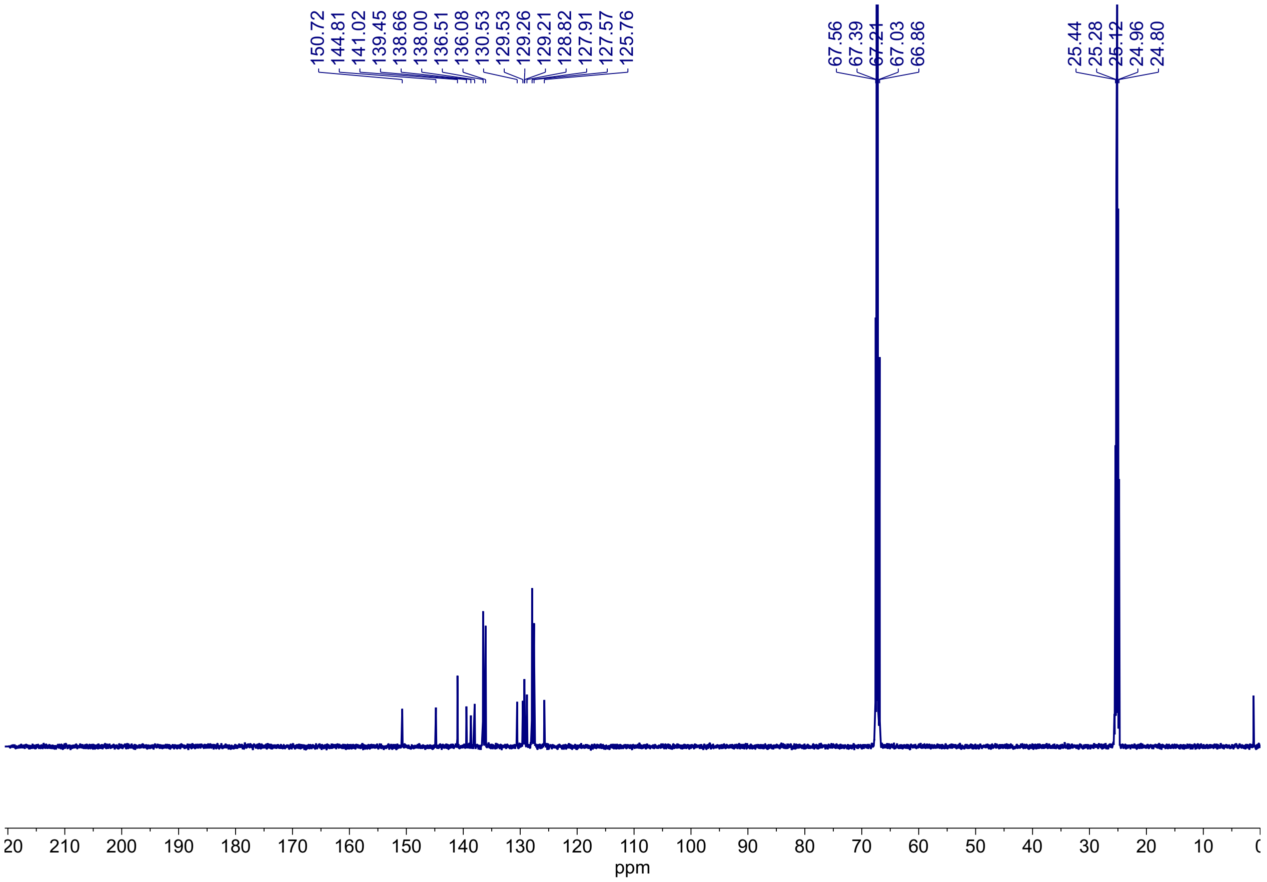


**Figure S14**. $^{13}C\{^{1}H\}$ NMR spectrum (126 MHz, THF-$d_8$, 298 K) of isolated [Ce((OSiPh$_2$Ar)$_3$-arene)(OSiPh$_3$)(THF)$_2$], **3-Ce$^{Ph}$**.

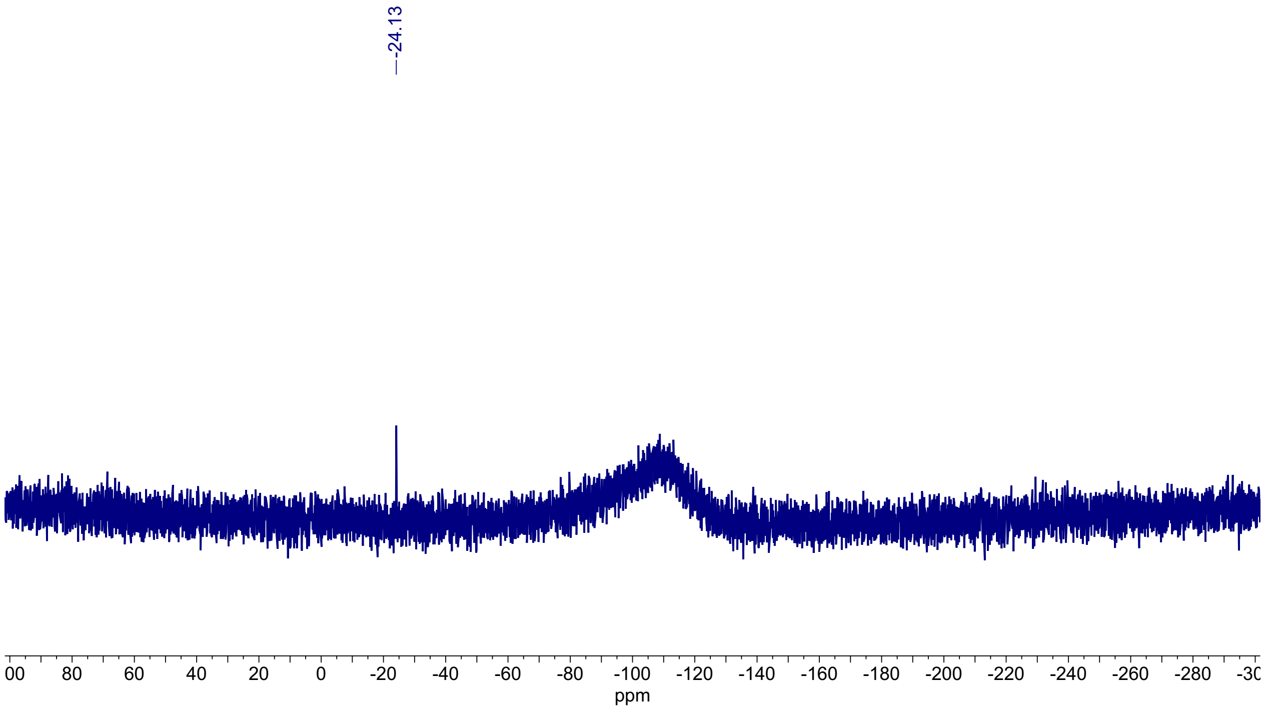


**Figure S15**. $^{29}Si\{^{1}H\}$ NMR spectrum (79.5 MHz, THF-$d_8$, 298 K) of isolated [Ce((OSiPh$_2$Ar)$_3$-arene)(OSiPh$_3$)(THF)$_2$], **3-Ce$^{Ph}$**.

**S3.2. NMR spectra for terbium complexes**

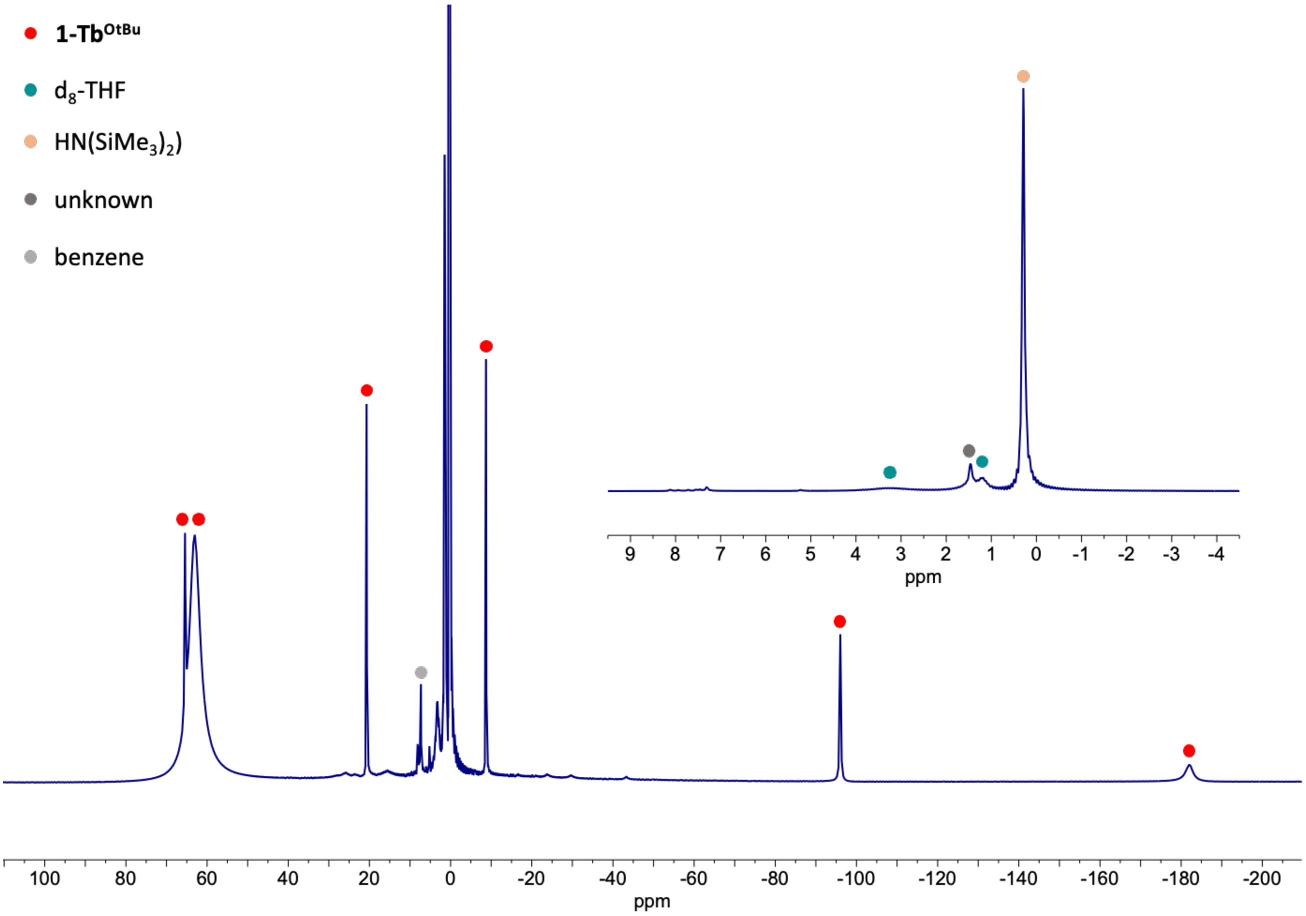


**Figure S16**. $^1$H NMR spectrum (400 MHz, THF-$d_8$, 298 K) of the reaction mixture obtained after addition of 1.0 equiv. of [Tb$^{III}$(N(SiMe$_3$)$_2$)$_3$] to (HOSi(O$^t$Bu)$_2$Ar)$_3$-arene after 15 minutes, resulting in complex **1-Tb$^{OtBu}$**.

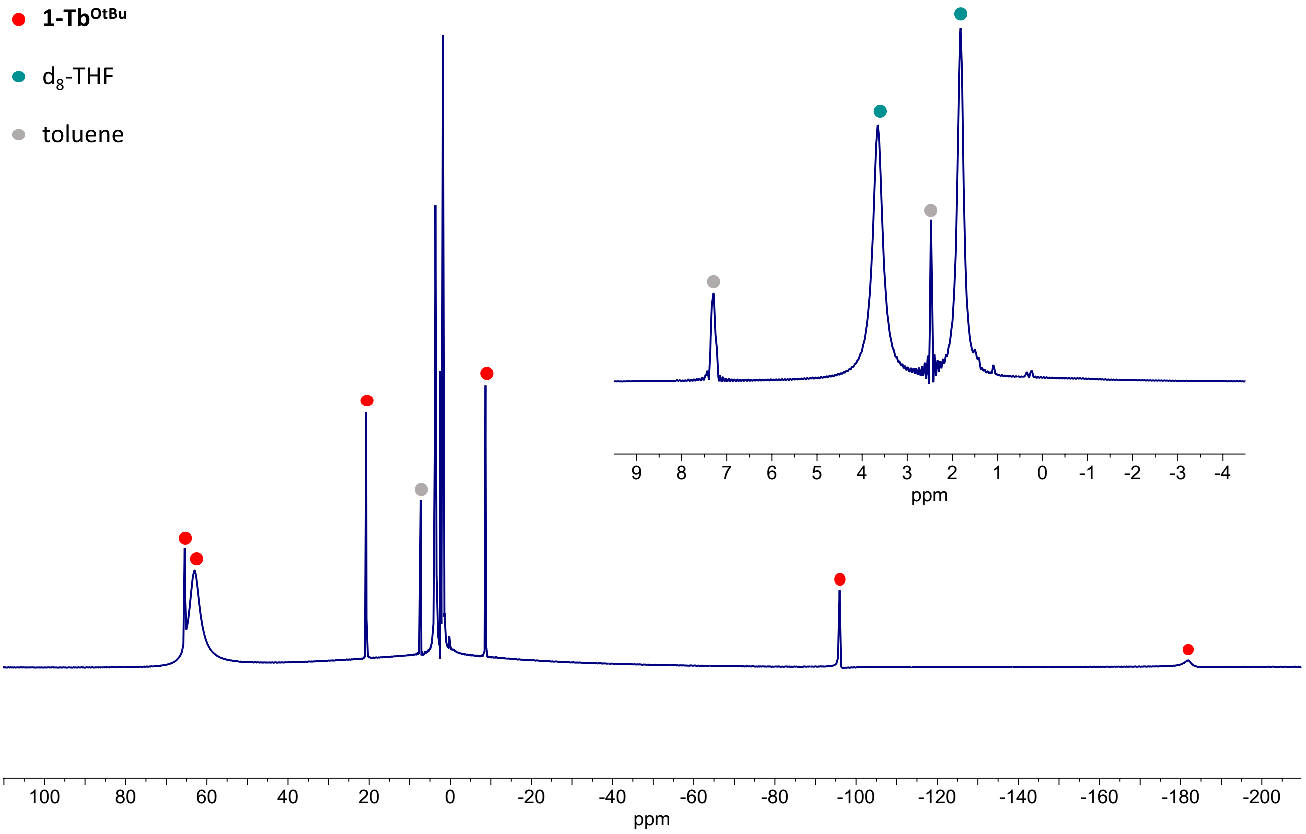


**Figure S17**. $^{1}$H NMR spectrum (400 MHz, THF-$d_8$, 298 K) of isolated [$Tb^{III}$((OSi(O$^t$Bu)$_2$Ar)$_3$-arene)(THF)$_3$], **1-Tb$^{OtBu}$**.

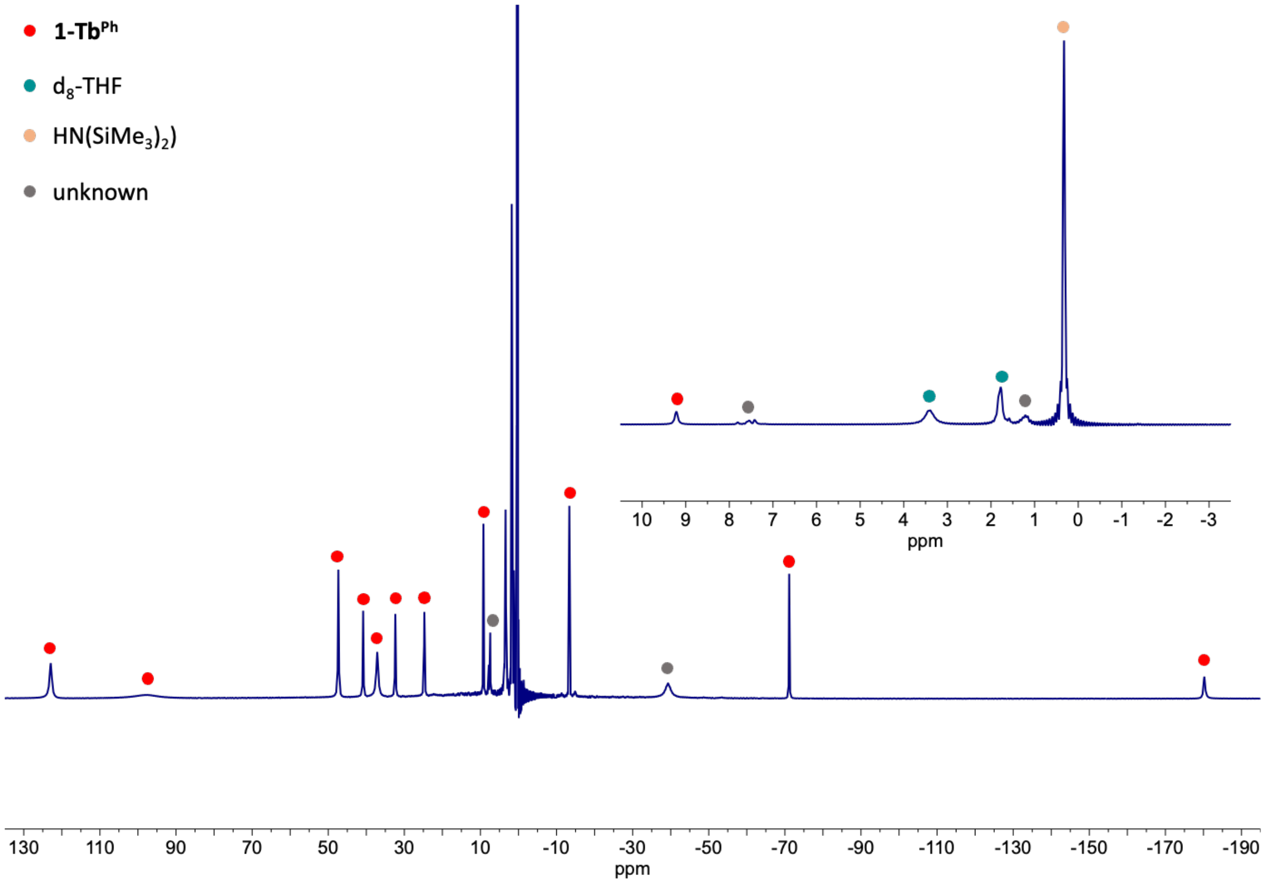


**Figure S18**. $^1$H NMR spectrum (400 MHz, THF-$d_8$, 298 K) of the reaction mixture obtained after addition of 1.0 equiv. of [$Tb^{III}(N(SiMe_3)_2)_3$] to ($HOSiPh_2Ar)_3$-arene after 15 minutes, resulting in complex **1-Tb$^{Ph}$**.

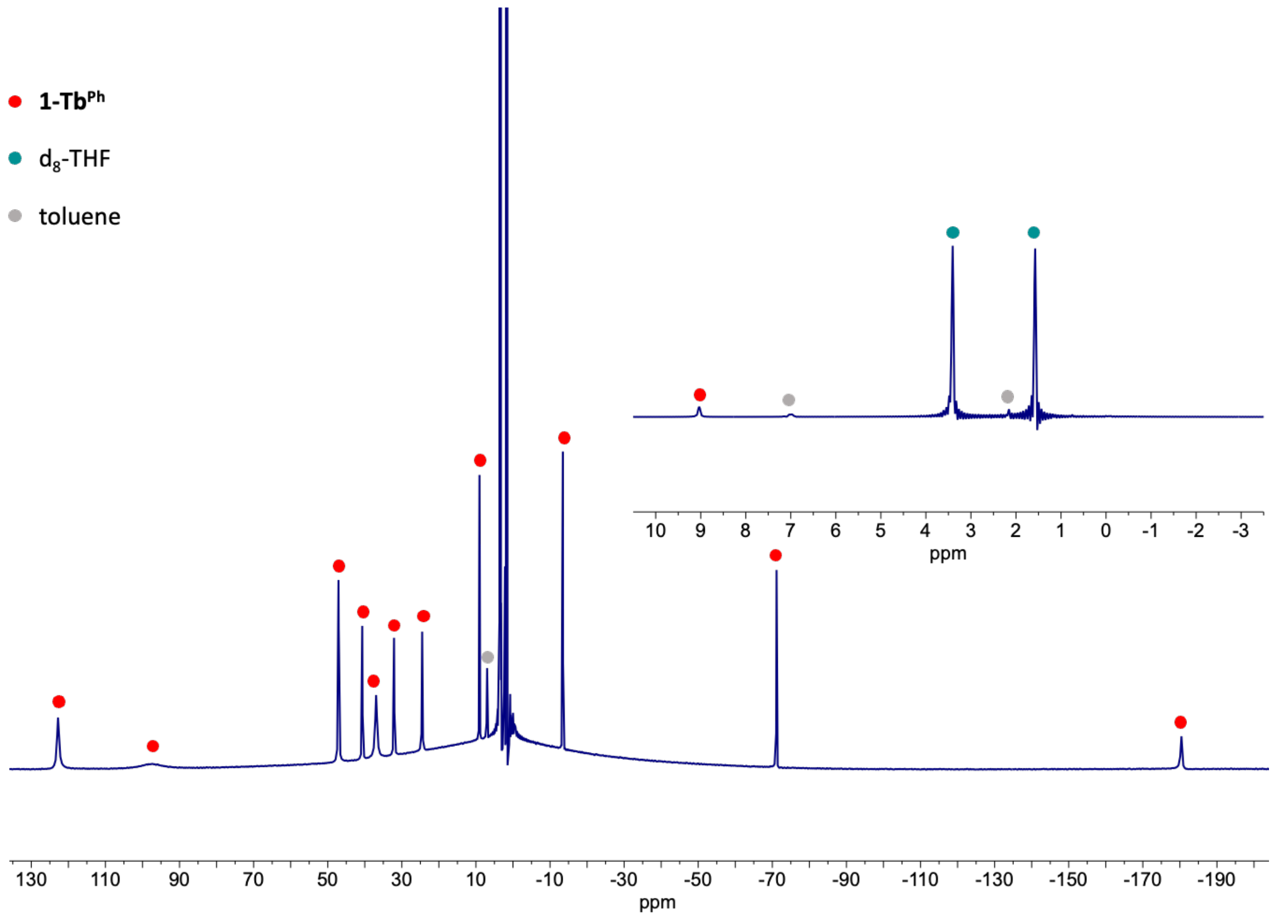


**Figure S19**. $^{1}$H NMR spectrum (400 MHz, THF-$d_8$, 298 K) of isolated [$Tb^{III}((OSiPh_2Ar)_3$-arene)$(THF)_3$], **1-Tb$^{Ph}$**.

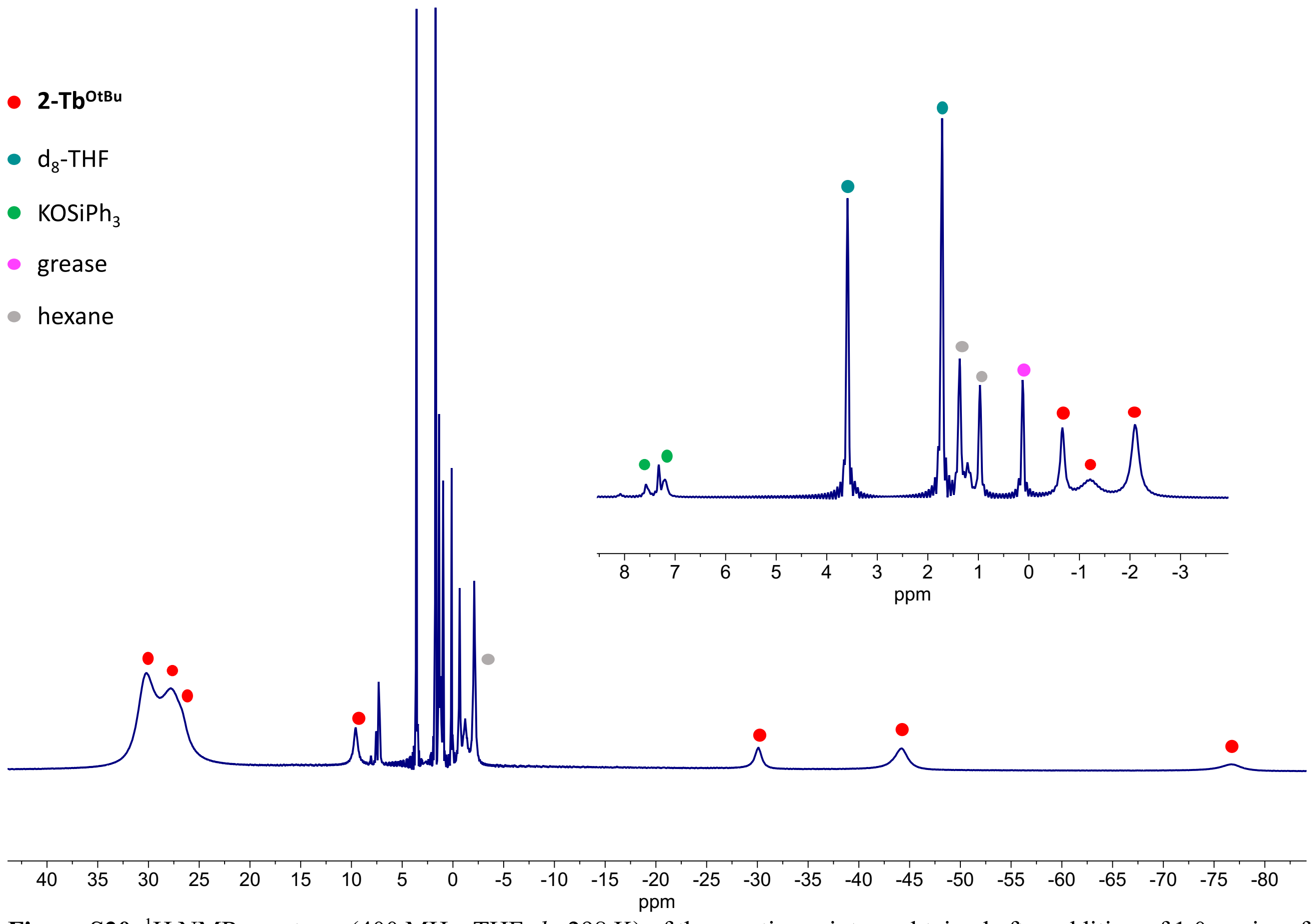


**Figure S20**. $^1$H NMR spectrum (400 MHz, THF-$d_8$, 298 K) of the reaction mixture obtained after addition of 1.0 equiv. of $KOSiPh_3$ to [$Tb^{III}((OSi(O^tBu)_2)_3$-arene)$(THF)_3$], **1-Tb$^{OtBu}$** after 30 mins, resulting in **2-Tb$^{OtBu}$**.

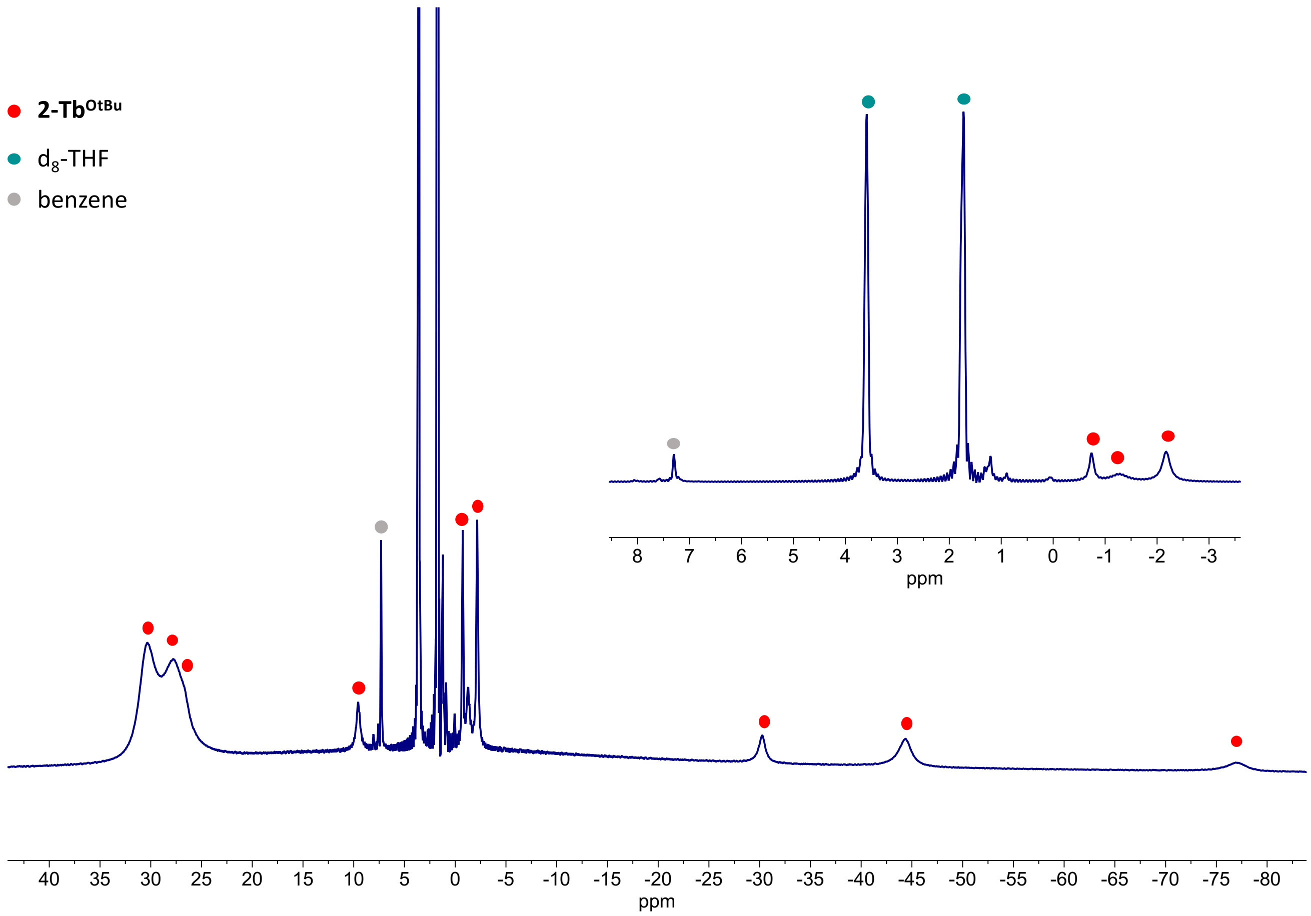


**Figure S21**. $^1$H NMR spectrum (400 MHz, THF-$d_8$, 298 K) of isolated [K(THF)$_6$][Tb$^{III}$((OSi(O$^t$Bu)$_2$Ar)$_3$-arene)(OSiPh$_3$)], **2-Tb$^{OtBu}$**.

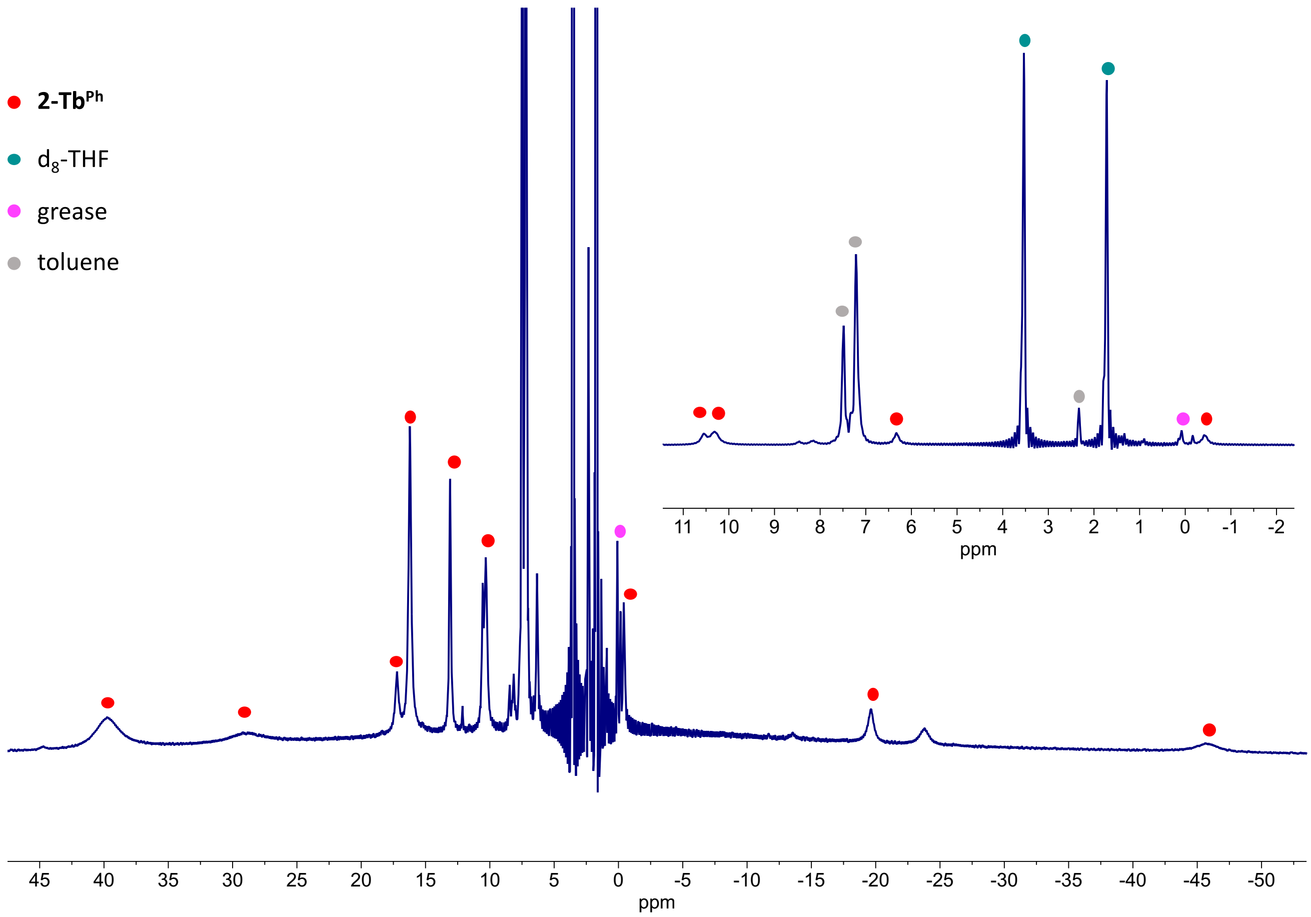


**Figure S22**. $^{1}H$ NMR spectrum (400 MHz, THF-$d_8$, 298 K) of the reaction mixture obtained after addition of 1.0 equiv. of $KOSiPh_3$ to $[Tb^{III}((OSiPh_2)_3\text{-arene})(THF)_3]$, **1-Tb$^{Ph}$** after 15 mins, resulting in **2-Tb$^{Ph}$**.

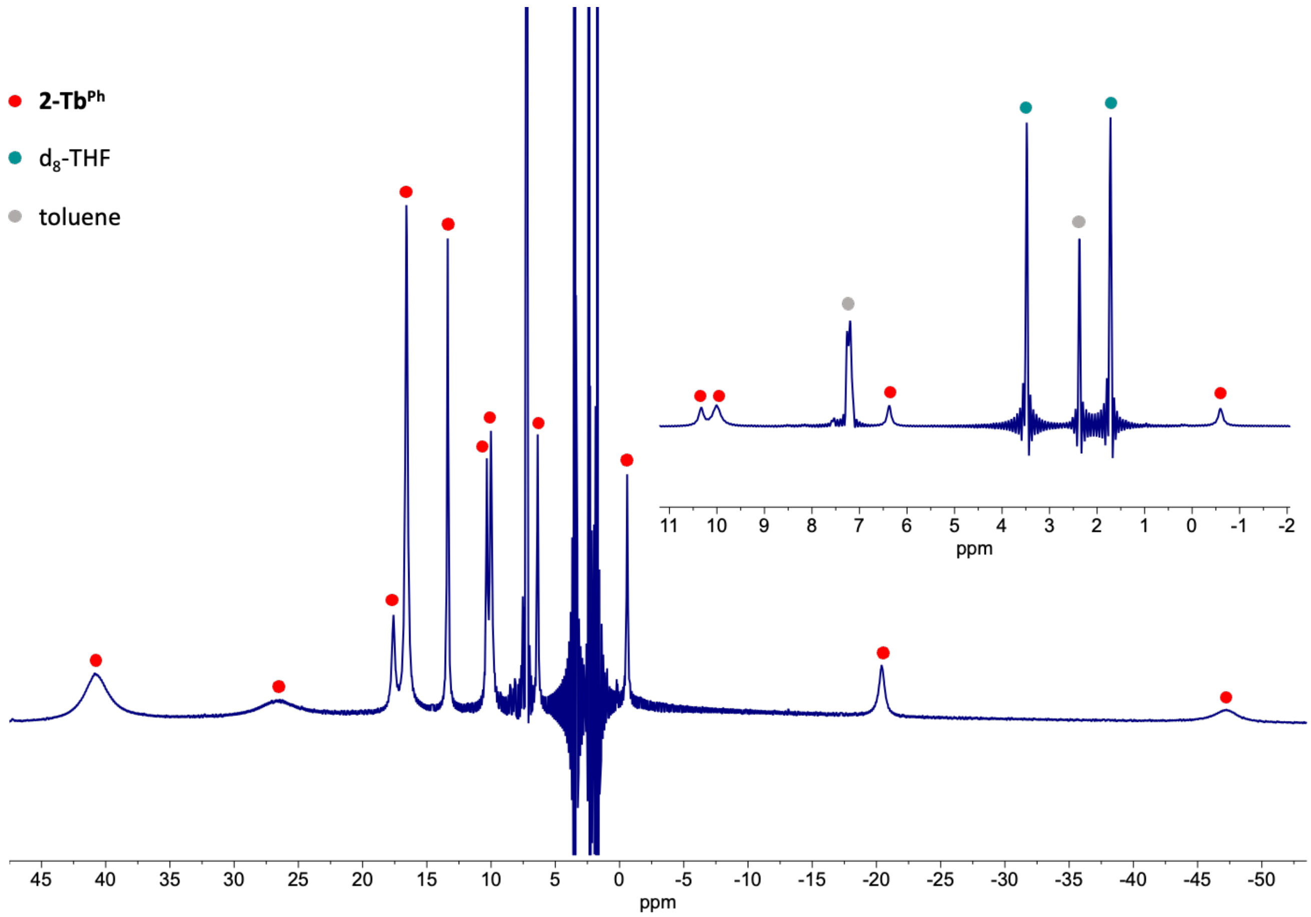


**Figure S23**. $^{1}$H NMR spectrum (400 MHz, THF-$d_8$, 298 K) of isolated [K{$Tb^{III}$((OSiPh$_2$Ar)$_3$-arene)(OSiPh$_3$)(toluene)}], **2-Tb$^{Ph}$**.

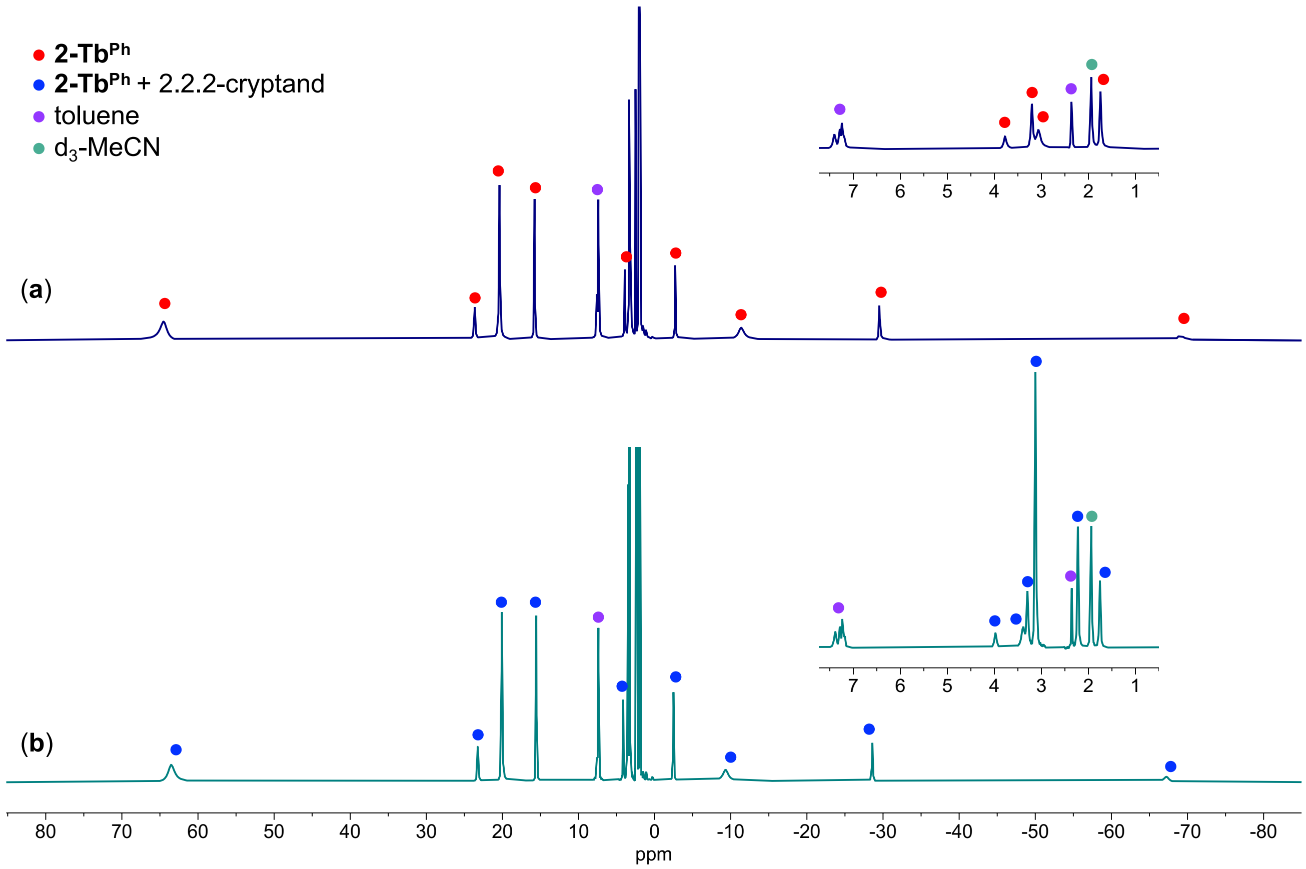


**Figure S24**. $^1$H NMR spectra (400 MHz, MeCN-$d_3$, 298 K) of **2-Tb$^{Ph}$** before a) and immediately after b) addition of 1.1 equiv. of 2.2.2-cryptand.

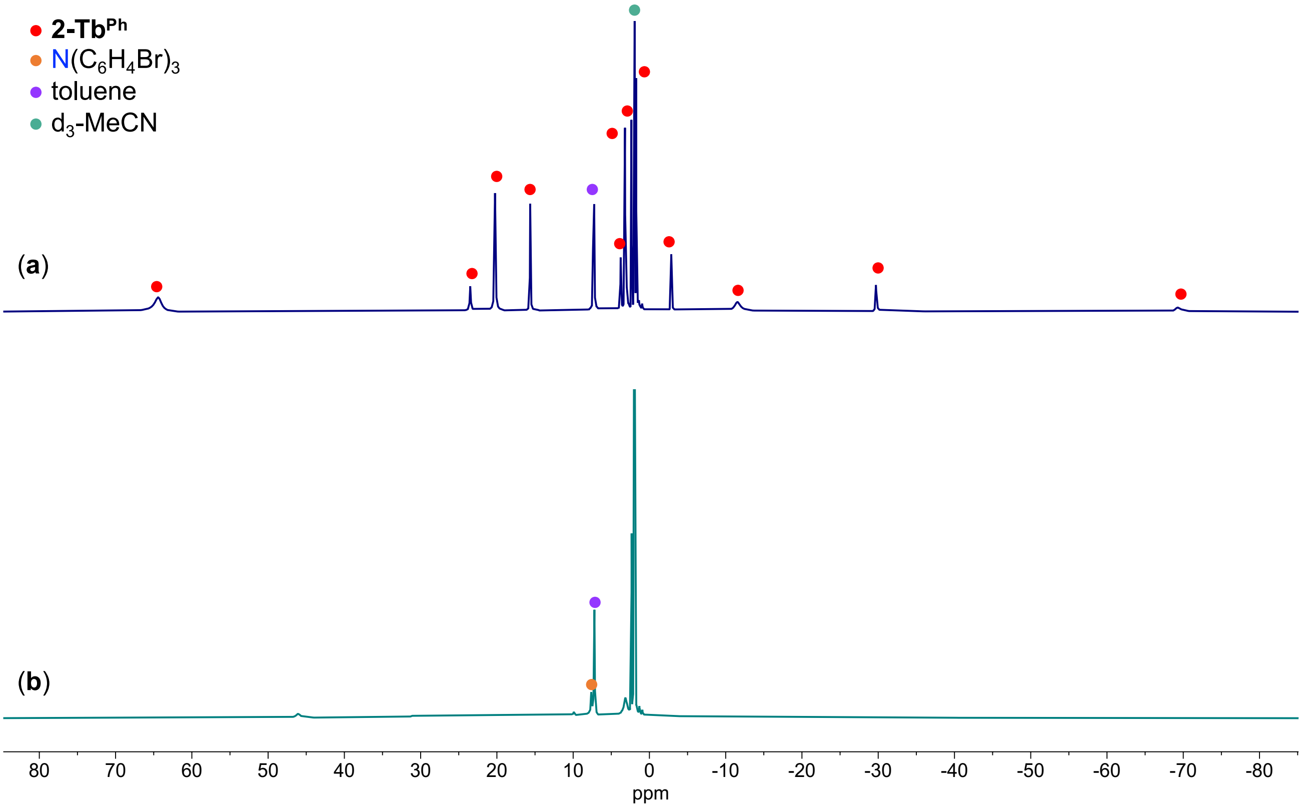


**Figure S25**. $^1$H NMR spectrum (400 MHz, MeCN-$d_3$, 298 K) of the reaction mixture obtained after addition of 1.1 equiv. of [N(C$_6$H$_4$Br)][SbCl$_6$] to **2-Tb$^{Ph}$**, 15 minutes after addition showing complete disappearance of **2-Tb$^{Ph}$** and a silent spectrum.

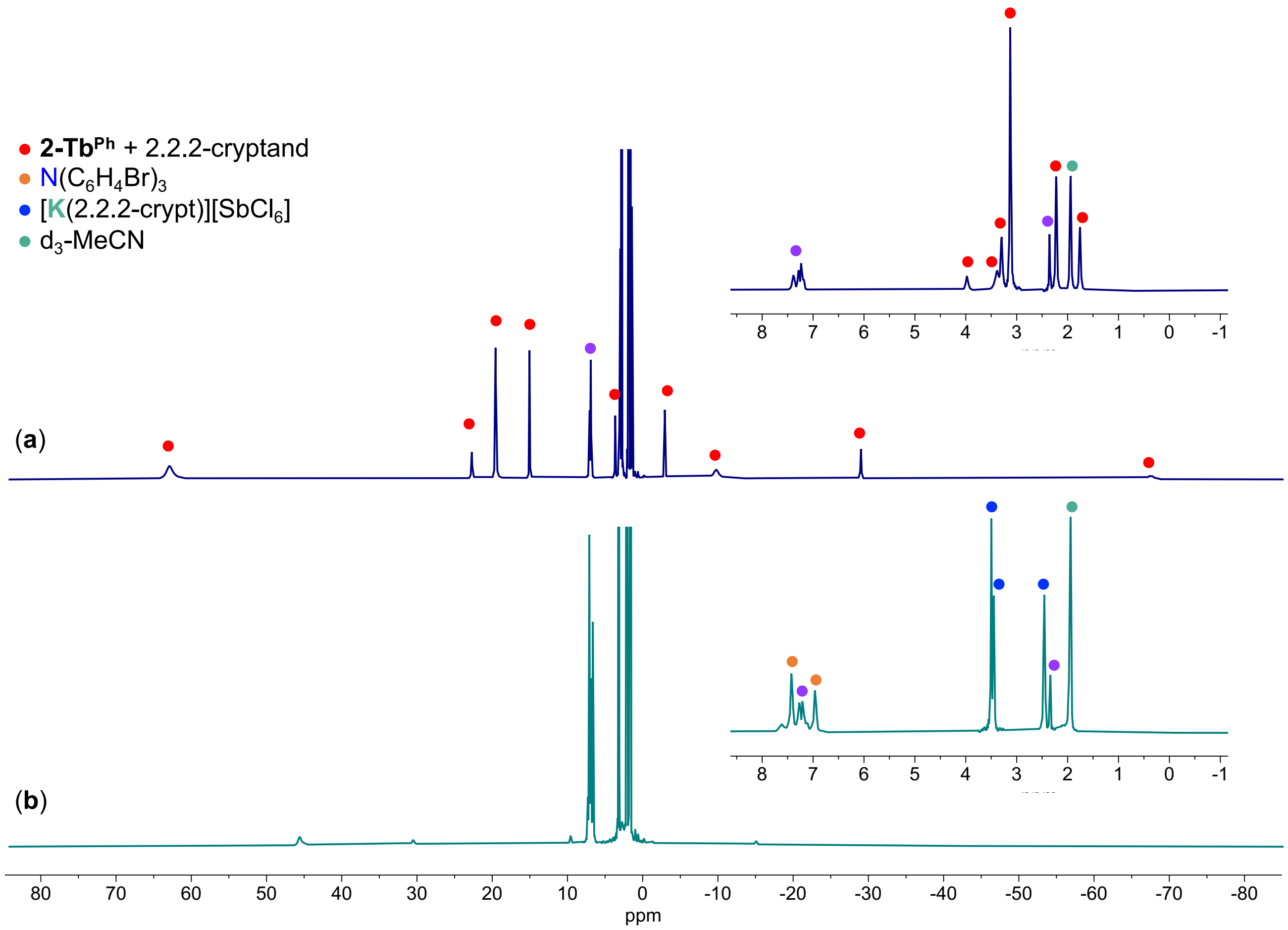


**Figure S26**. $^{1}$H NMR spectrum (400 MHz, MeCN-$d_3$, 298 K) of the reaction mixture obtained after addition of 1.1 equiv. of $[N(C_6H_4Br)][SbCl_6]$ to **2-Tb$^{Ph}$** and 2.2.2-cryptand, 15 minutes after addition.

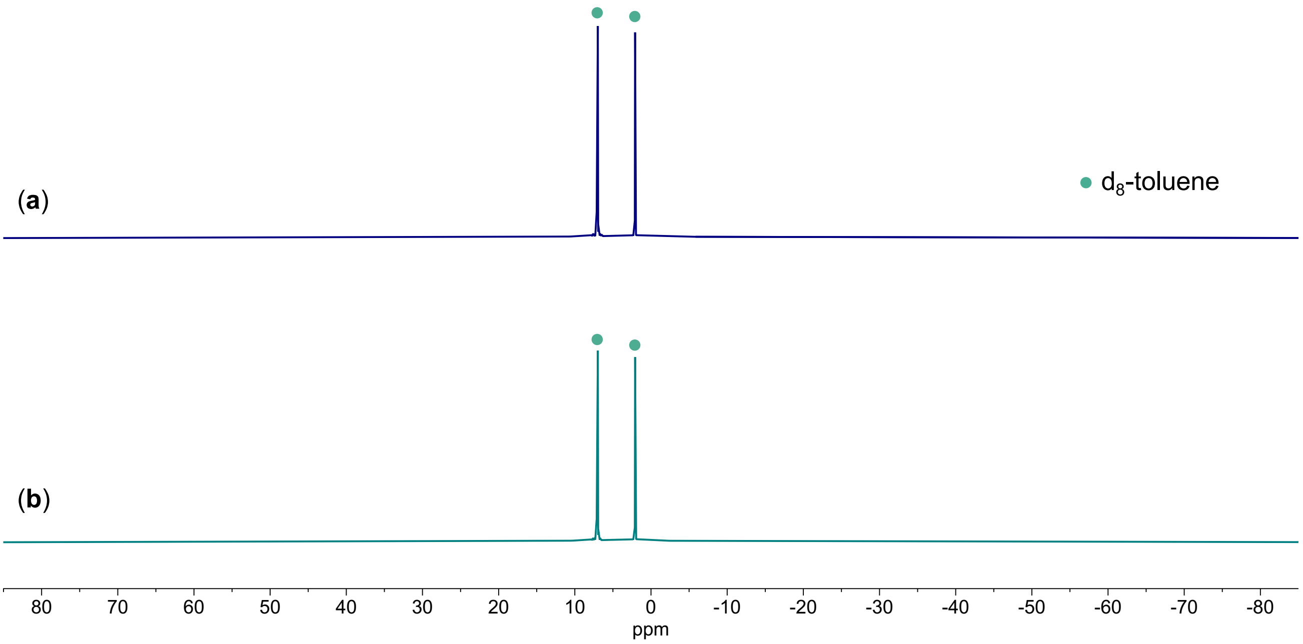


**Figure S27**. $^{1}$H NMR spectrum (400 MHz, toluene-$d_8$, 298 K) of a) isolated [$Tb^{IV}((OSiPh_2Ar)_3$-arene)$(OSiPh_3)(MeCN)_2$], **3-Tb$^{Ph}$**. The spectrum is silent due to the Tb(IV) 4$f^7$ ion. b) $^{1}$H NMR spectrum after 2 days.

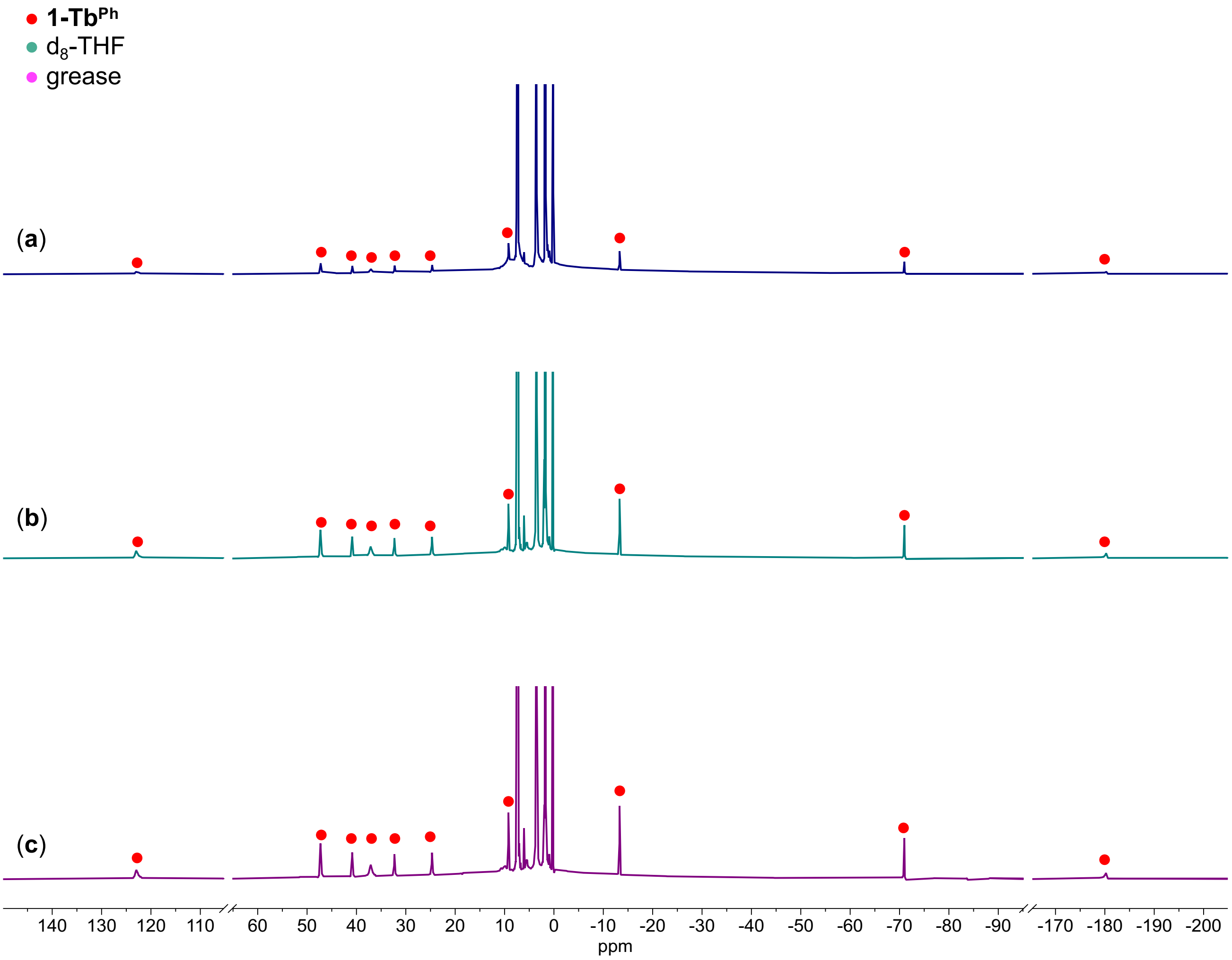


**Figure S28**. $^{1}$H NMR spectrum (400 MHz, THF-$d_8$, 298 K) of isolated [$Tb^{IV}$((OSiPh$_2$Ar)$_3$-arene)(OSiPh$_3$)(MeCN)$_2$], **3-Tb$^{Ph}$** after a) 15 minutes, b) 9 hours, c) 24 hours.

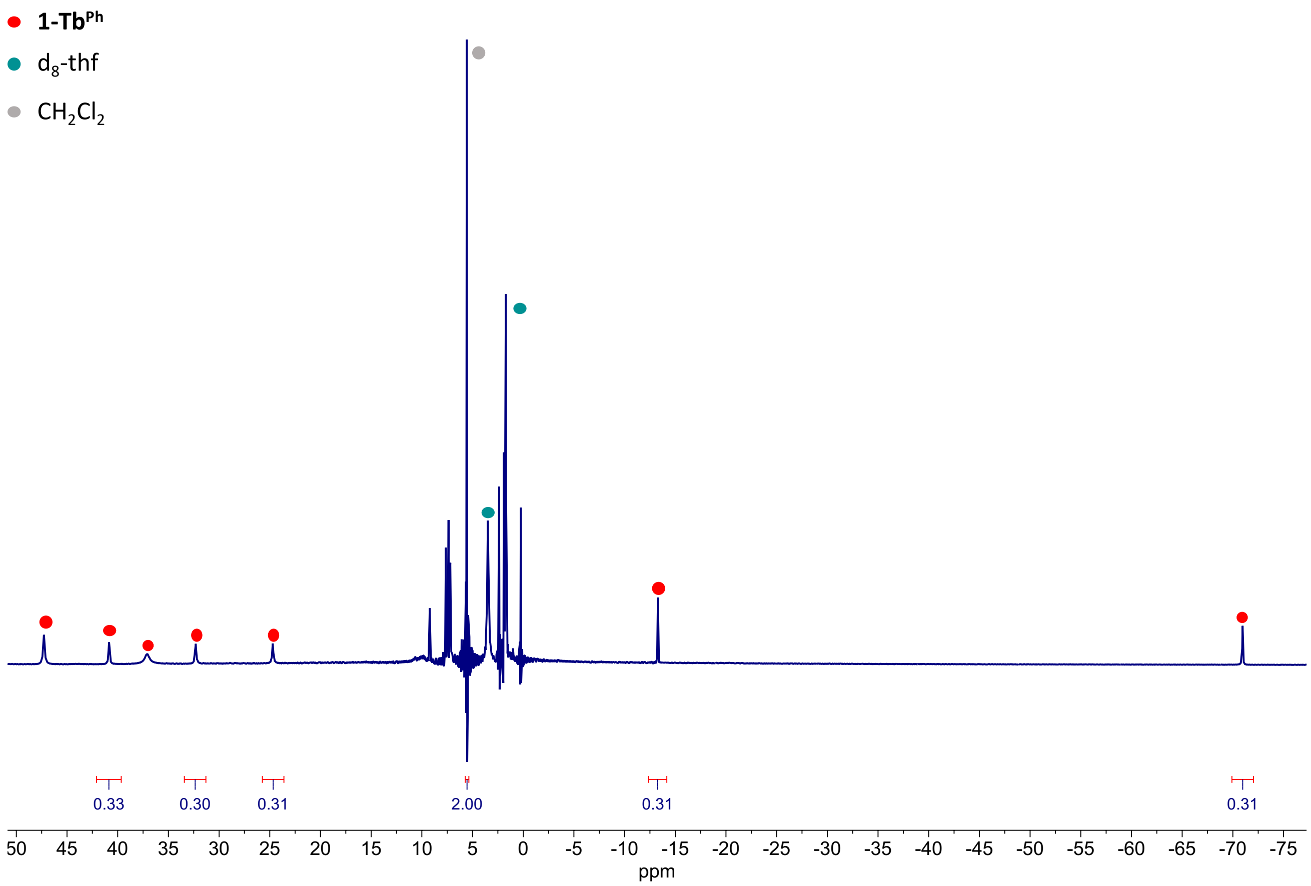


**Figure S29**. $^1$H NMR spectrum (400 MHz, THF-$d_8$, 298 K) of 8.0 mg of isolated [$Tb^{IV}$(($OSiPh_2Ar)_3$-arene)($OSiPh_3$)($MeCN)_2$], **3-Tb$^{Ph}$** after 24 hours at room temperature, 3.0 µL of $CH_2Cl_2$ was added as internal standard to quantify the amount of **1-Tb$^{Ph}$** reformed (83%).

### S3.3. NMR spectra for praseodymium complexes

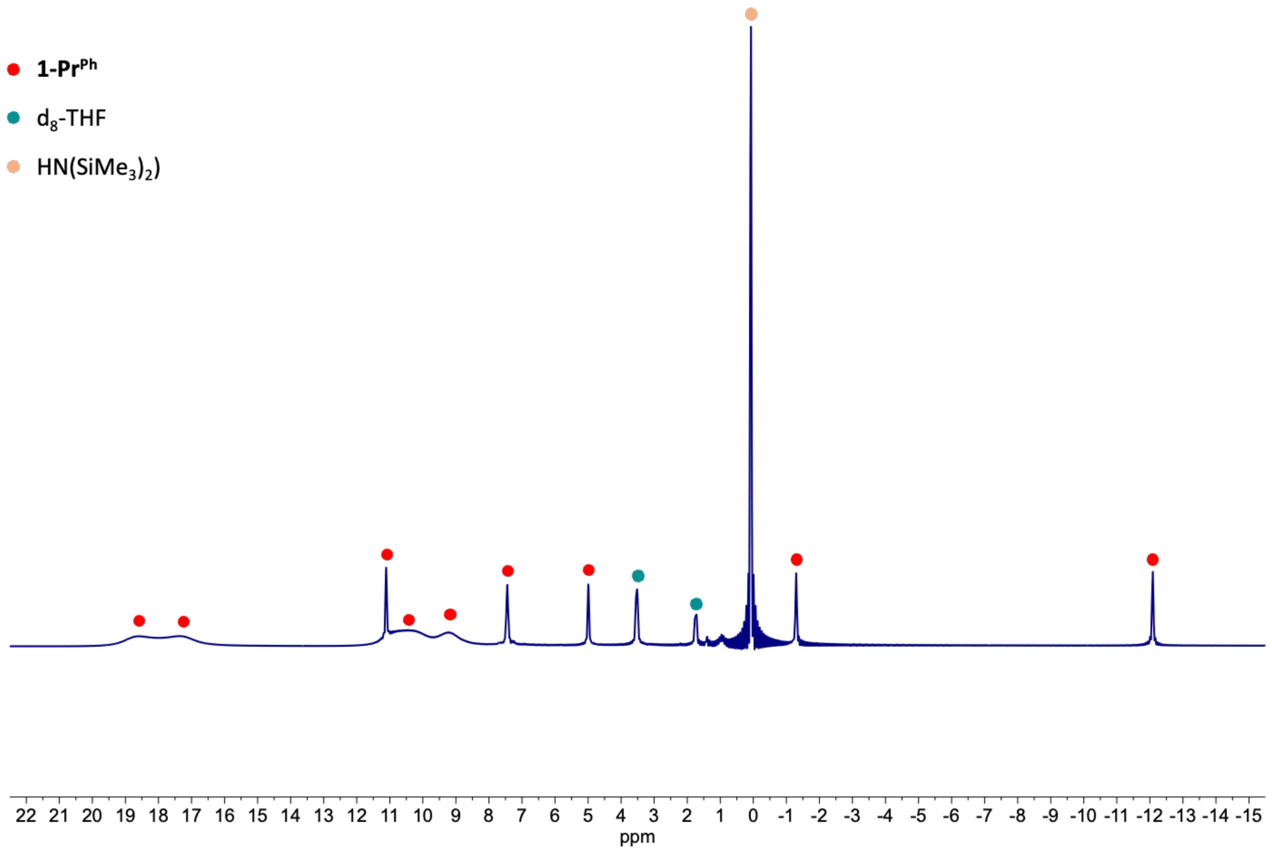


**Figure S30**. $^1$H NMR spectrum (400 MHz, THF-$d_8$, 298 K) of the reaction mixture obtained after addition of 1.0 equiv. of $[Pr^{III}(N(SiMe_3)_2)_3]$ to $(HOSiPh_2Ar)_3$-arene after 15 minutes, resulting in complex **1-Pr$^{Ph}$**.

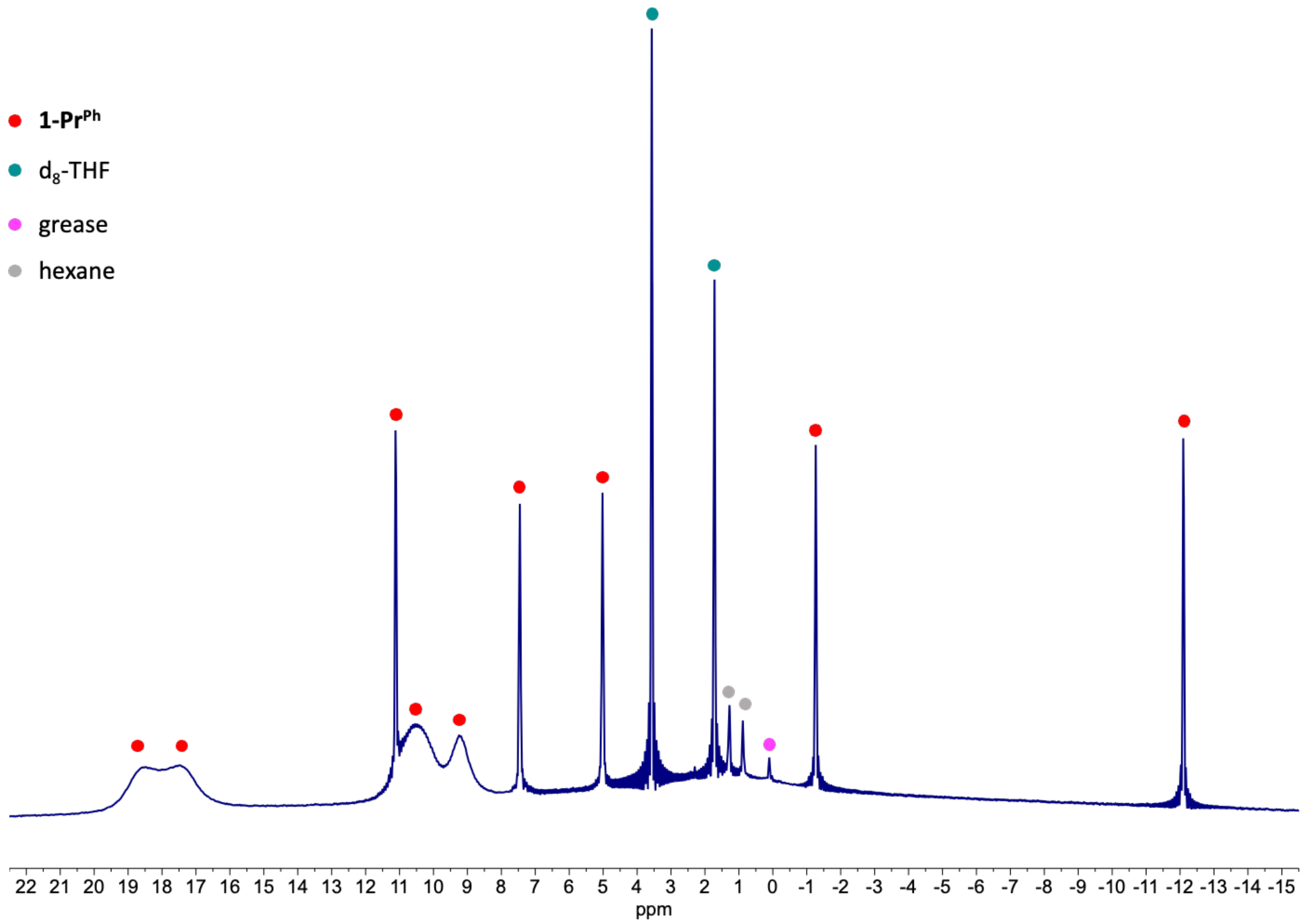


**Figure S31**. $^{1}$H NMR spectrum (400 MHz, THF-$d_8$, 298 K) of isolated [$Pr^{III}$($(OSiPh_2Ar)_3$-arene)$(THF)_3$], **1-Pr$^{Ph}$**.

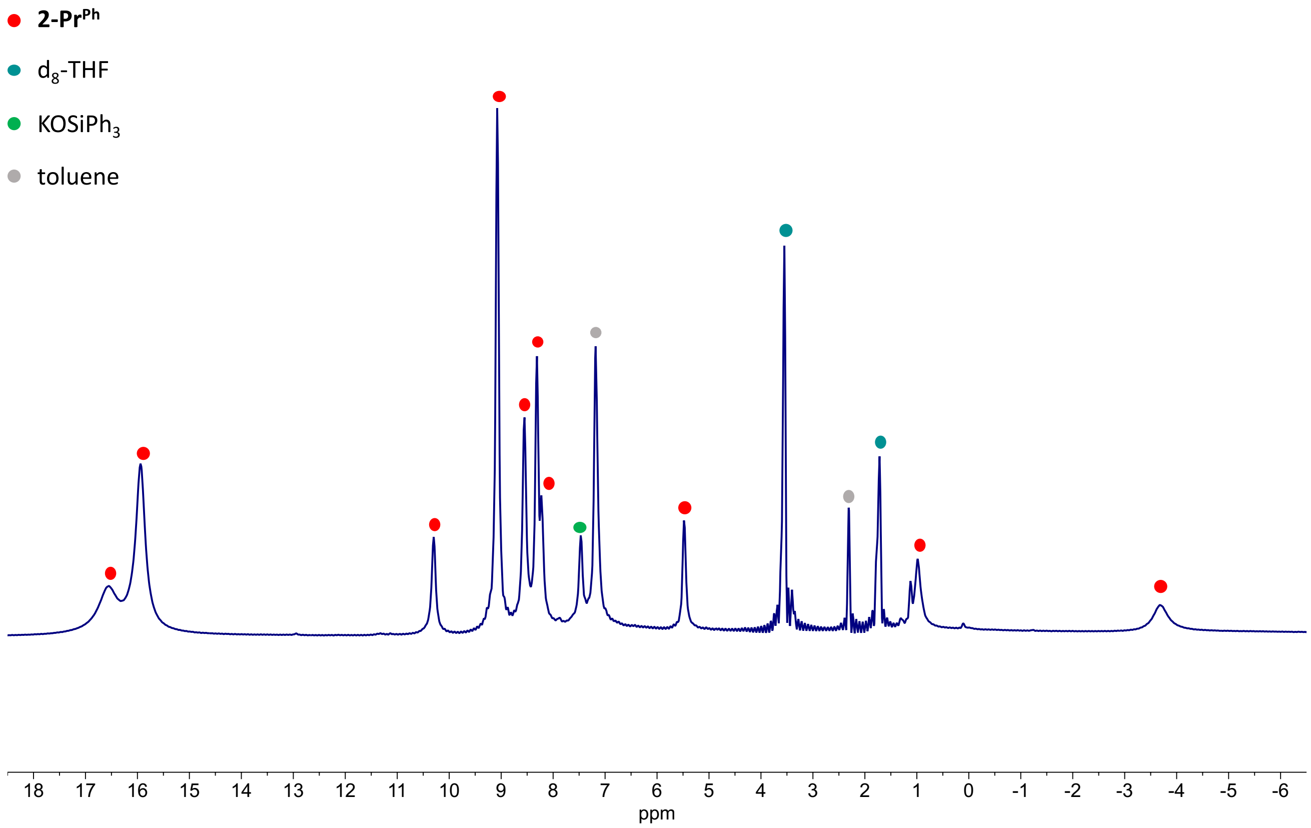


**Figure S32**. $^{1}H$ NMR spectrum (400 MHz, THF-$d_8$, 298 K) of the reaction mixture obtained after addition of 1.0 equiv. of $KOSiPh_3$ to $[Pr^{III}((OSiPh_2)_3\text{-arene})(THF)_3]$, **1-Pr$^{Ph}$** after 30 mins, resulting in **2-Pr$^{Ph}$**.

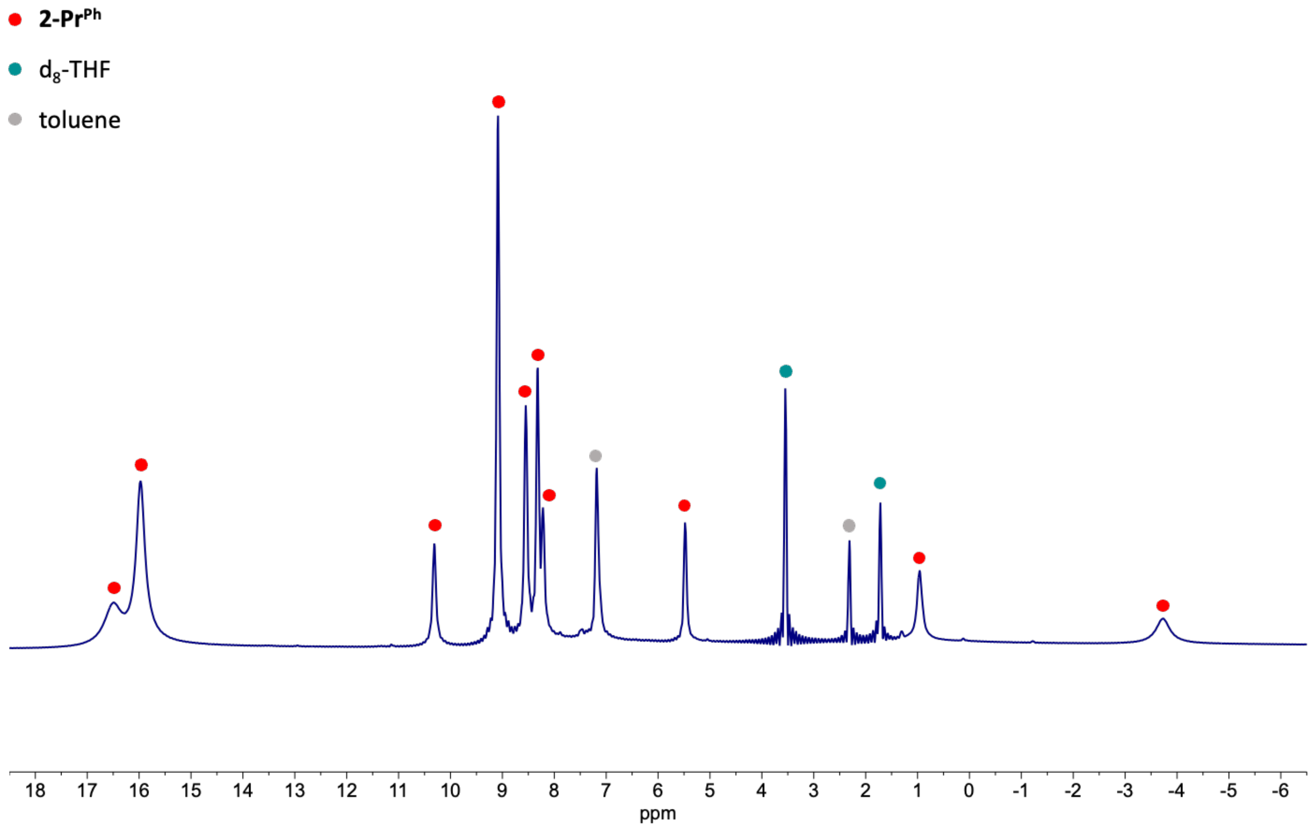


**Figure S33**. $^1$H NMR spectrum (400 MHz, THF-$d_8$, 298 K) of isolated [K{$Pr^{III}$((OSi$Ph_2$Ar)$_3$-arene)(OSi$Ph_3$)(toluene)}], **2-Pr$^{Ph}$**.

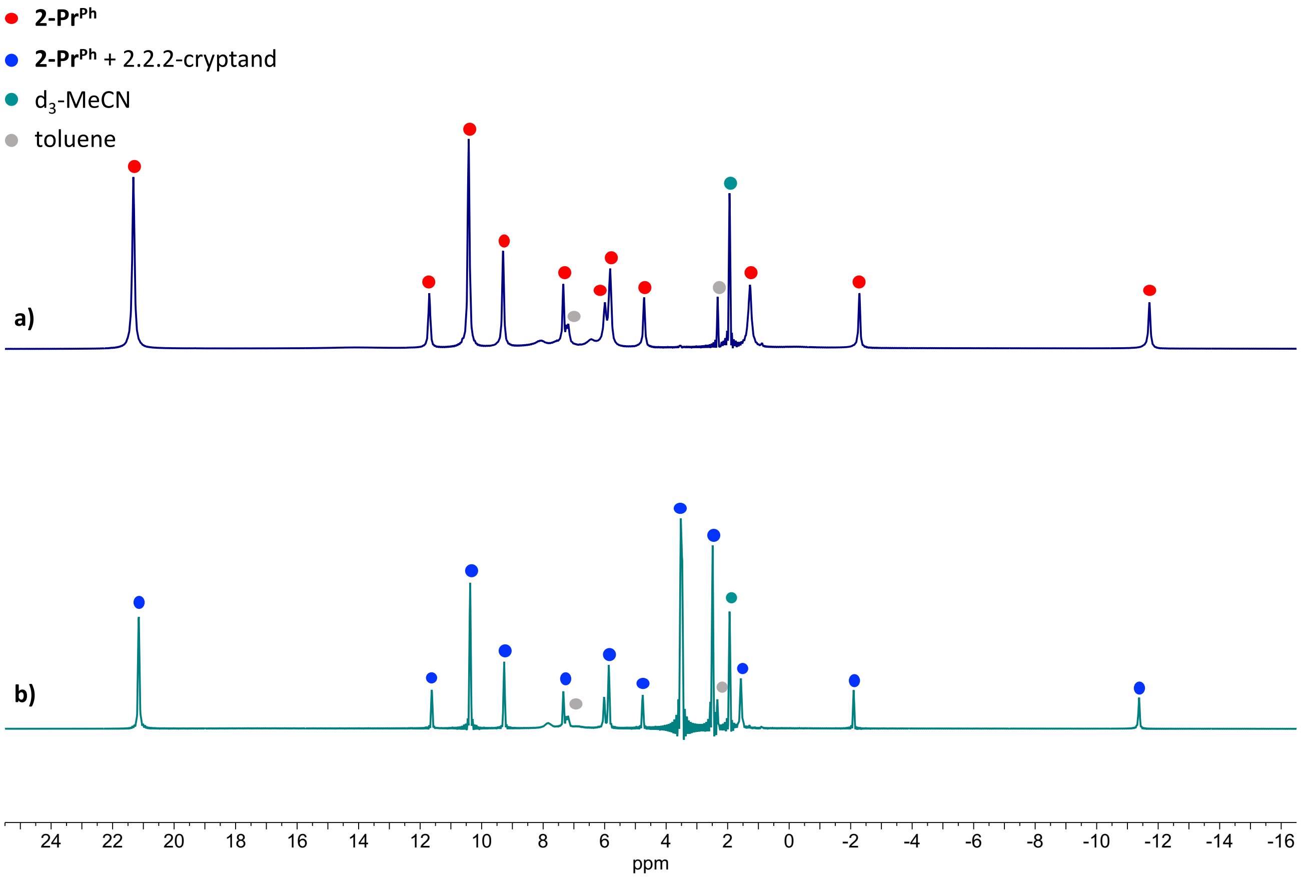


**Figure S34**. $^{1}H$ NMR spectra (400 MHz, MeCN-$d_3$, 298 K) of **2-Pr$^{Ph}$** a) and **2-Pr$^{Ph}$** + 1.1 equiv. of 2.2.2-cryptand b).

**S3.4. NMR spectra of in-situ addition of KOSi(O$^t$Bu)$_3$, KOSiMe$_3$ and 2-KOAd and K(N(SiMe$_3$)$_2$) to 1-Tb$^{Ph}$**

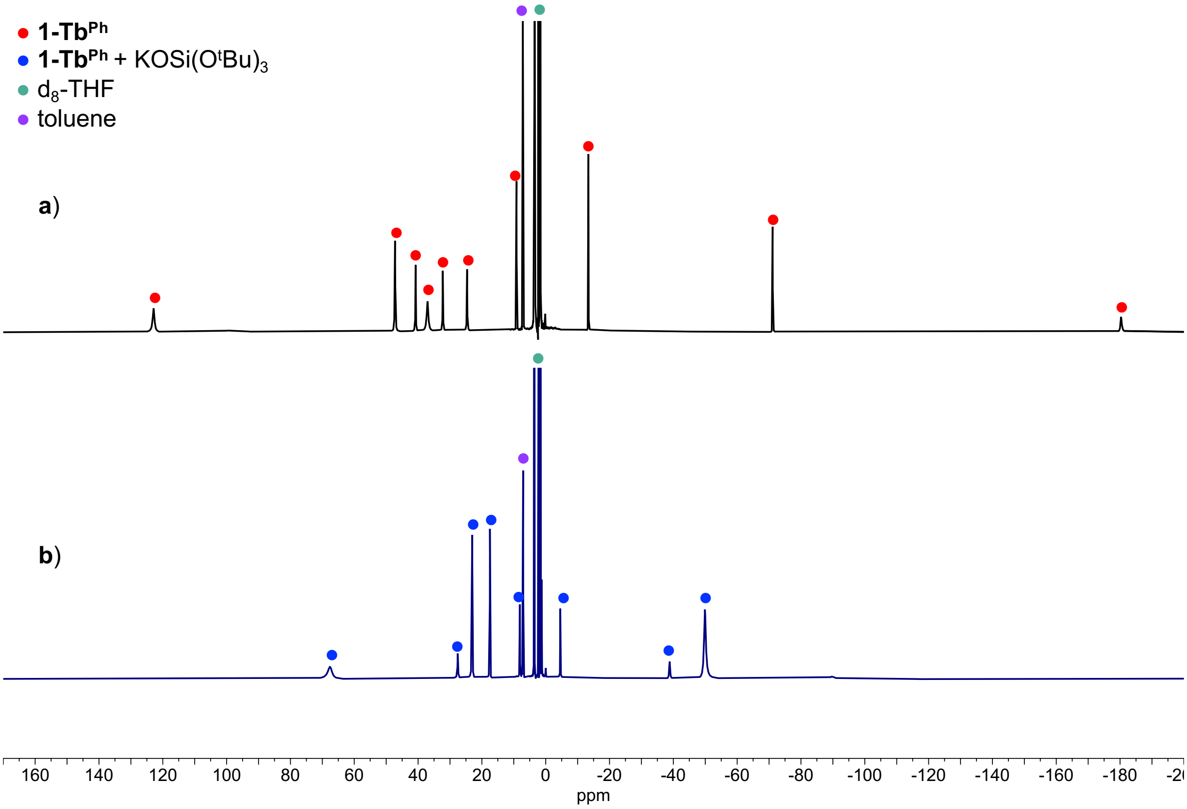


**Figure S35**. $^1$H NMR spectra (400 MHz, THF-$d_8$, 298 K) of **1-Tb$^{Ph}$** a) and **1-Tb$^{Ph}$** + 1.0 equiv. of KOSi(O$^t$Bu)$_3$ after 6 hours, b).

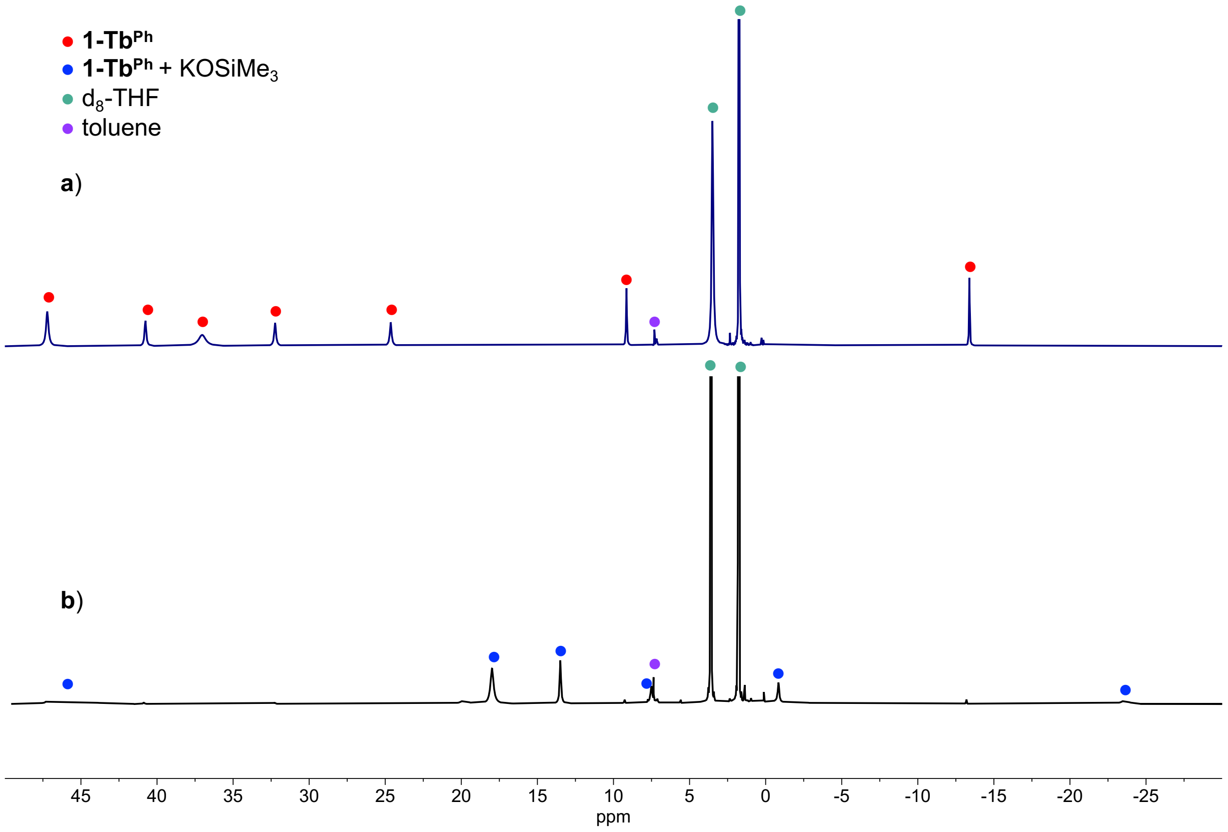


**Figure S36**. $^{1}$H NMR spectra (400 MHz, THF-$d_8$, 298 K) of **1-Tb$^{Ph}$** a) and **1-Tb$^{Ph}$** + 1.1 equiv. of $KOSiMe_3$ after 3 h b).

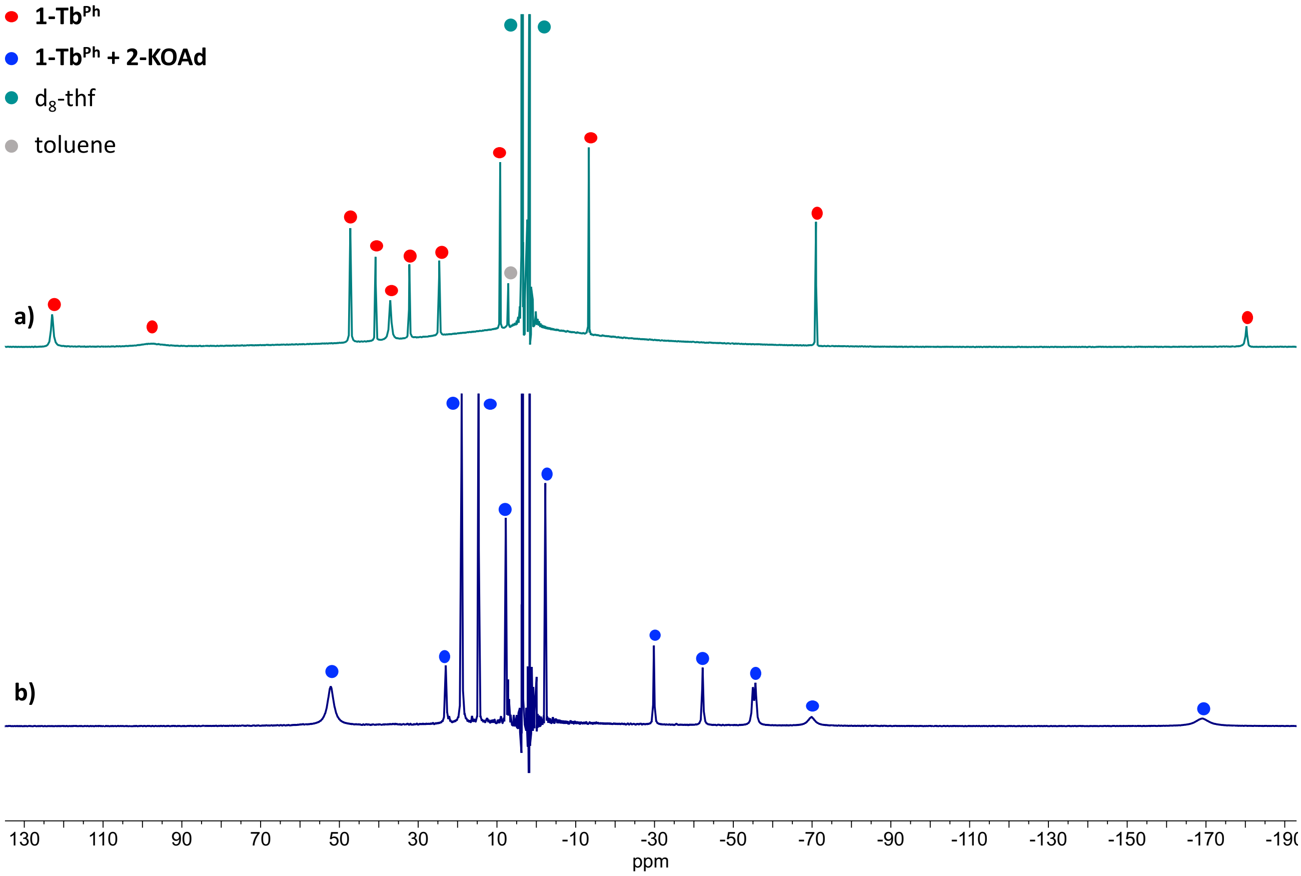


**Figure S37**. $^1$H NMR spectra (400 MHz, THF-$d_8$, 298 K) of **1-Tb$^{Ph}$** a) and **1-Tb$^{Ph}$** + 1.1 equiv. of 2-KOAd after 3 h, b).

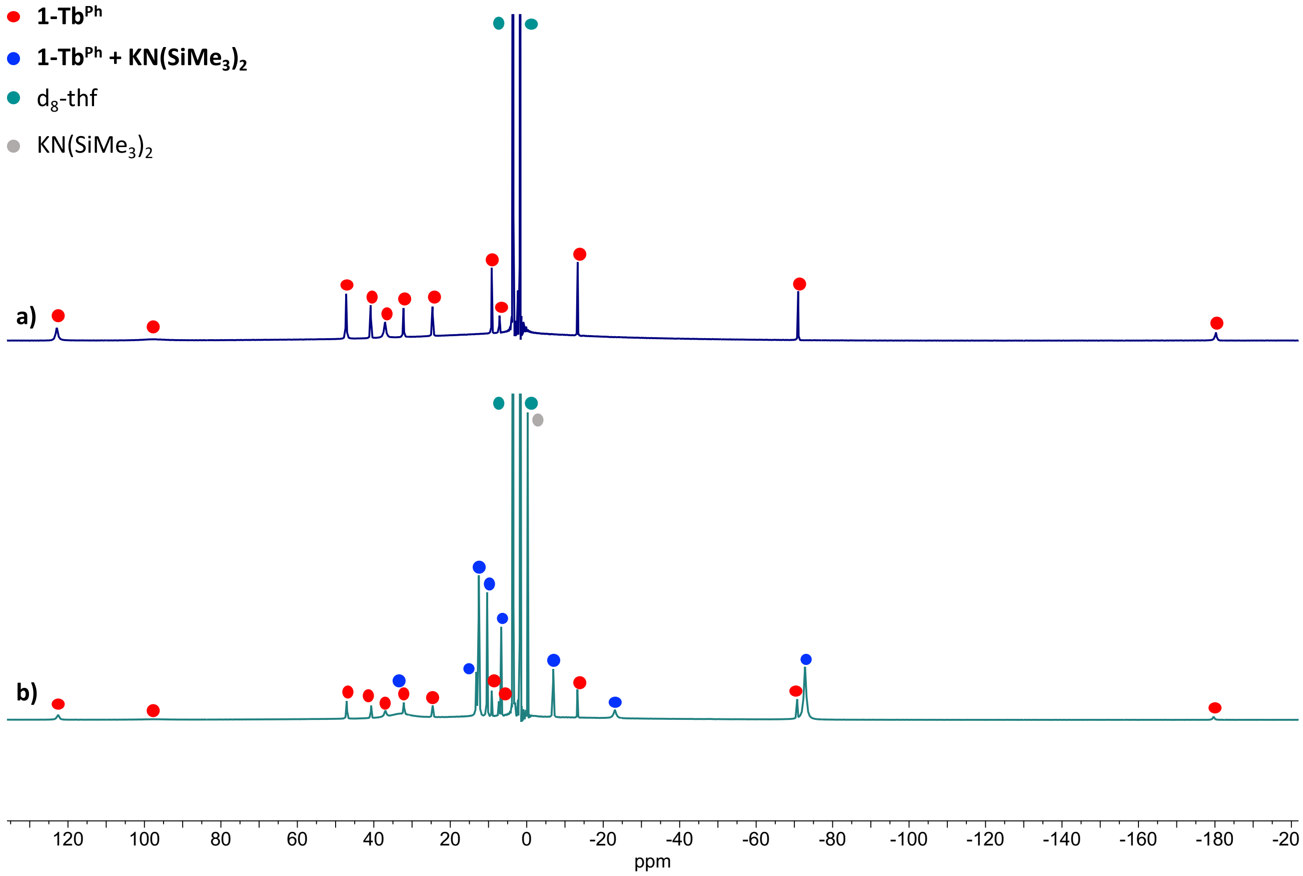


**Figure S38**. $^1$H NMR spectra (400 MHz, THF-$d_8$, 298 K) of **1-Tb$^{Ph}$** a) and **1-Tb$^{Ph}$** + 1.1 equiv. of $K(N(SiMe_3)_2)$ after 16 h, some **1-Tb$^{Ph}$** remained b). Almost no further conversion could be observed by adding an additional equivalent of $K(N(SiMe_3)_2)$.

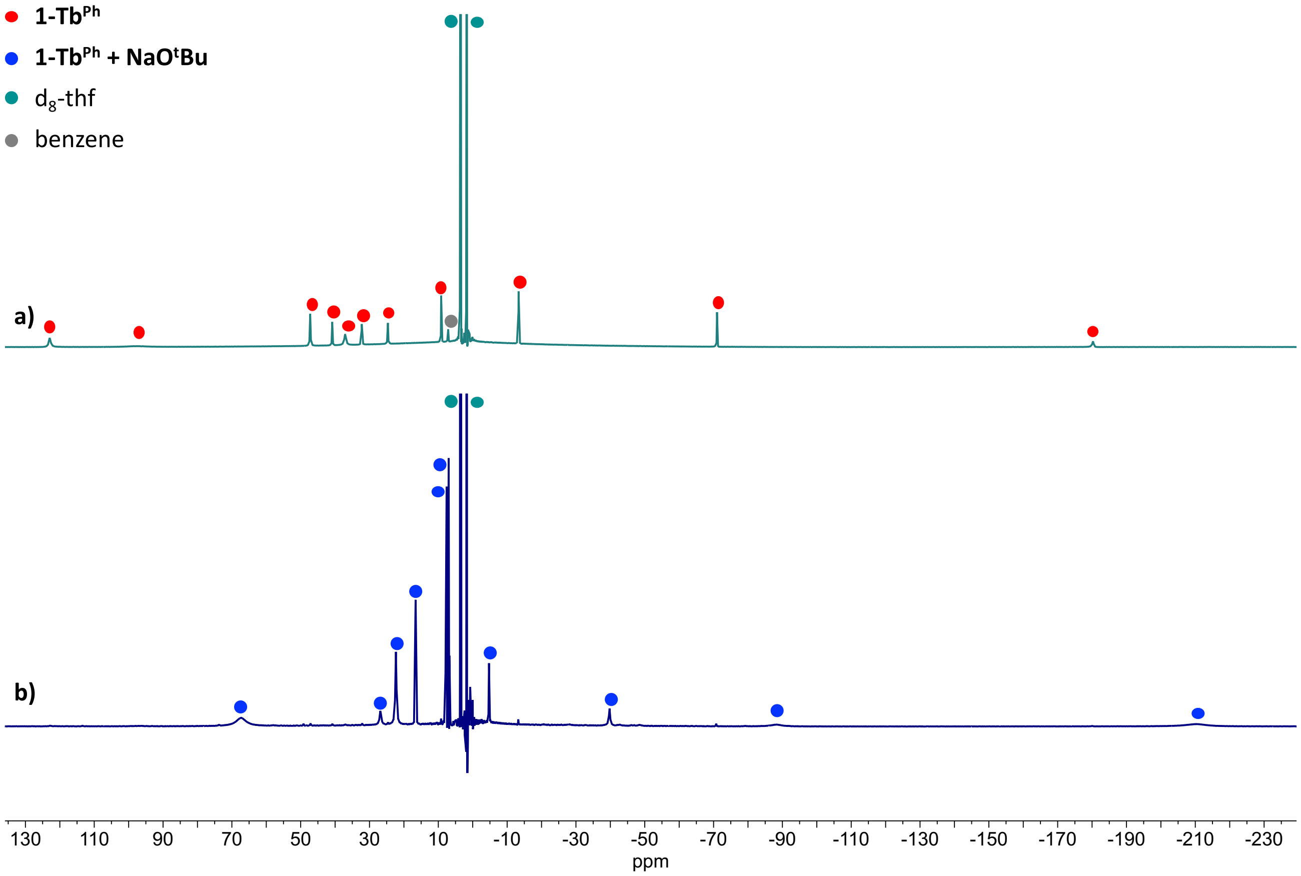


**Figure S39**. $^{1}H$ NMR spectra (400 MHz, THF-$d_8$, 298 K) of **1-Tb$^{Ph}$** a) and b) **1-Tb$^{Ph}$** + 1.5 equiv. of NaO$^{t}$Bu after 16 h.

### S3.5. NMR spectra of in-situ addition of $KOSiMe_3$ and 2-KOAd to 1-Pr$^{Ph}$

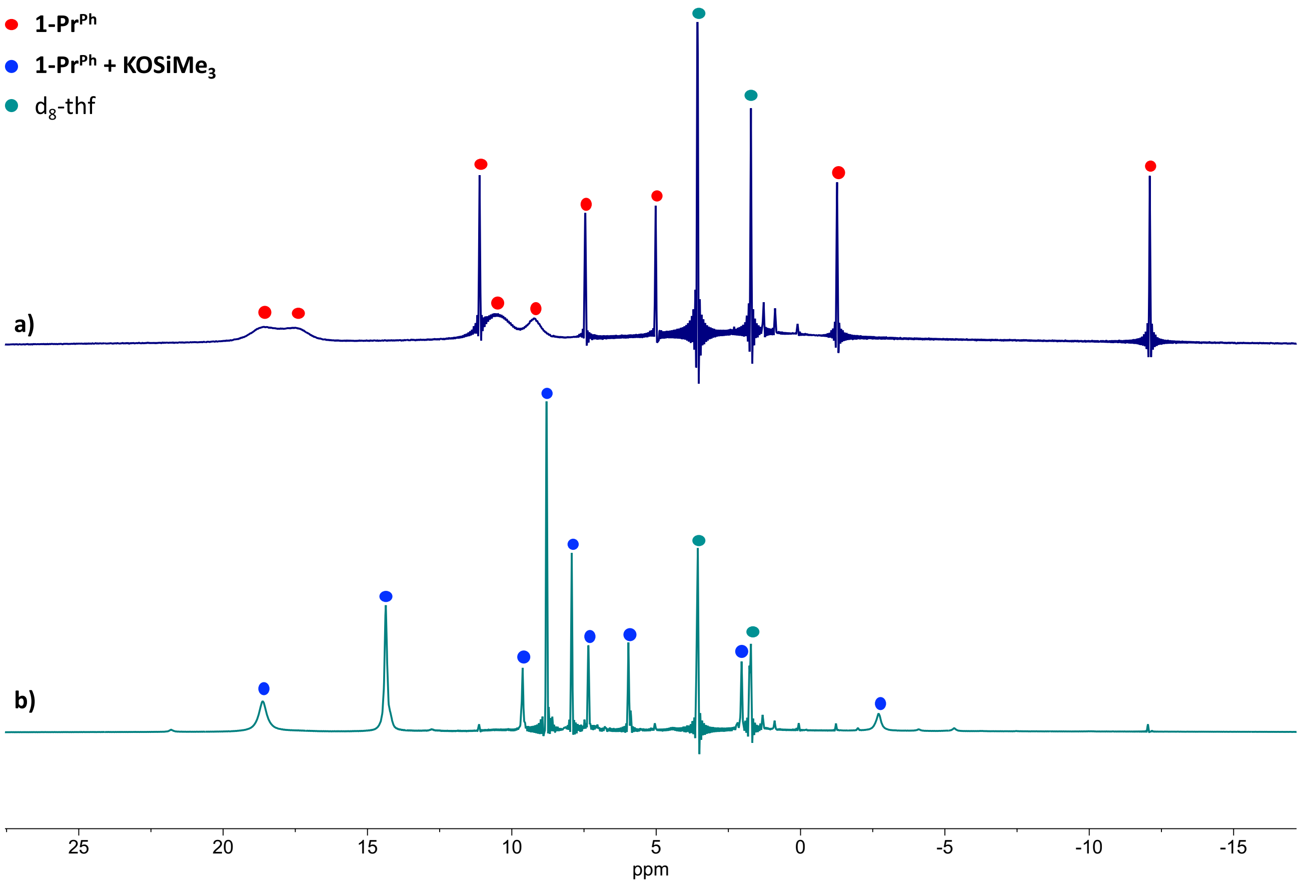


**Figure S40**. $^1$H NMR spectra (400 MHz, THF-$d_8$, 298 K) of **1-Pr$^{Ph}$** a) and b) **1-Pr$^{Ph}$** + 1.1 equiv. of $KOSiMe_3$ after 1 h.

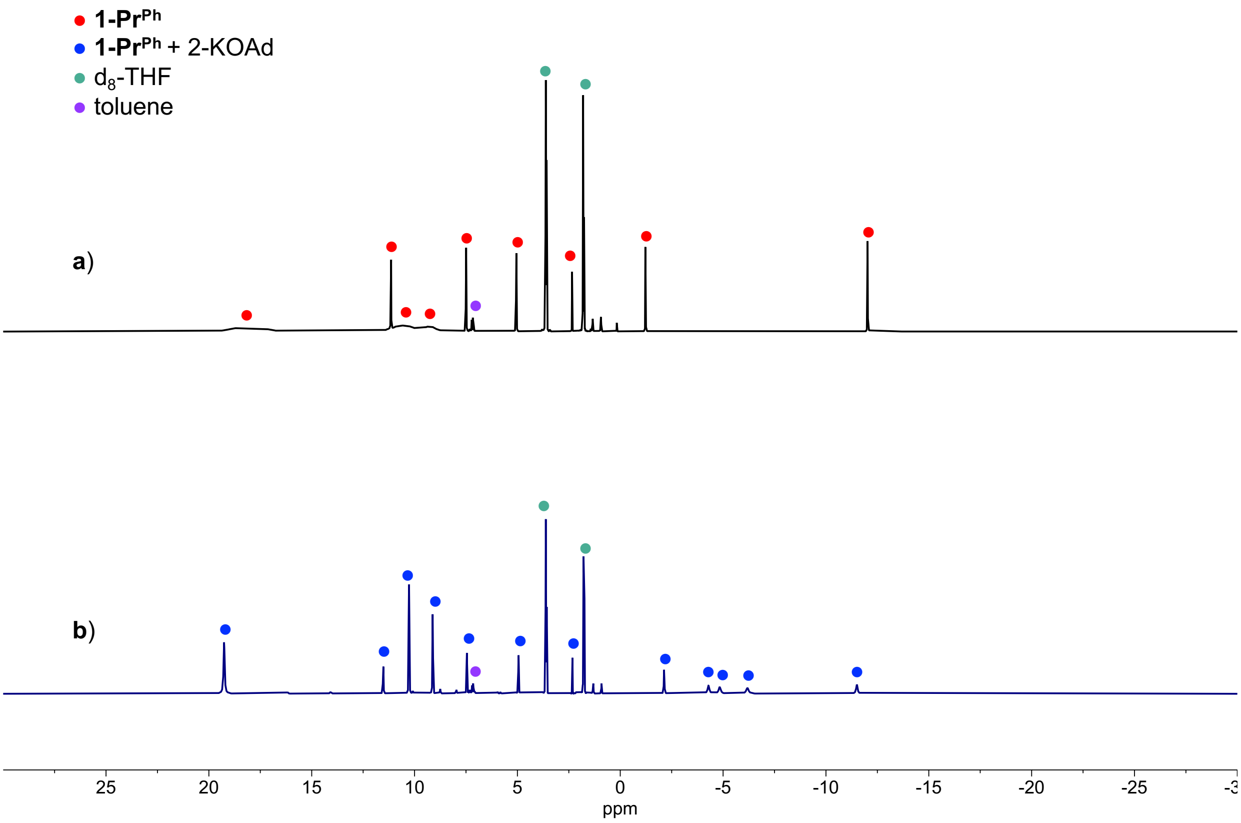


**Figure S41**. $^{1}$H NMR spectra (400 MHz, THF-$d_8$, 298 K) of **1-Pr$^{Ph}$** a) and **1-Pr$^{Ph}$** + 1.1 equiv. of 2-KOAd after 3 h. b).

### S3.6. NMR spectra of the Tb/Dy separation trials

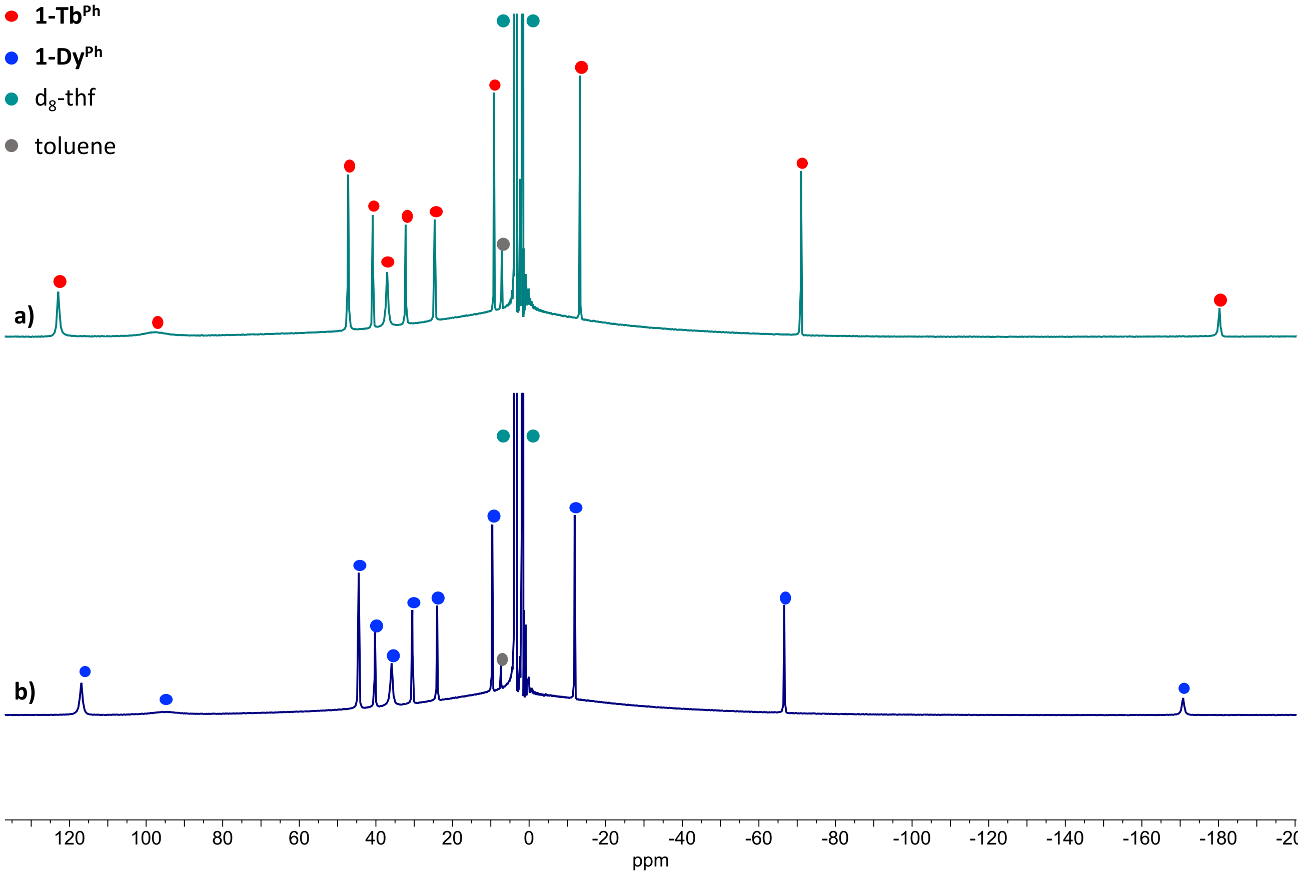


**Figure S42**. $^{1}$H NMR spectrum (400 MHz, THF-$d_8$, 298 K) of a) **1-Tb$^{Ph}$**, b) **1-Dy$^{Ph}$**.

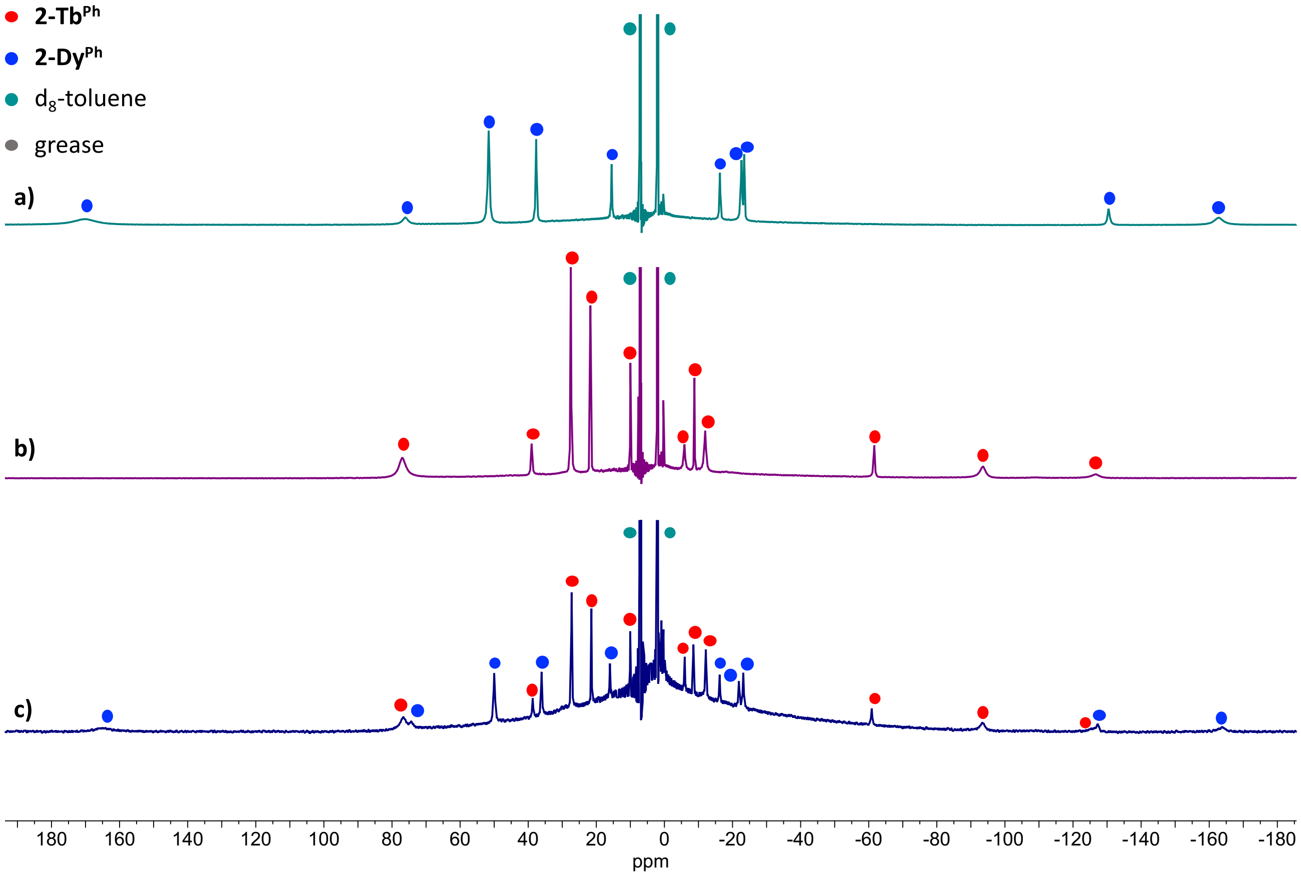


**Figure S43**. $^{1}$H NMR spectrum (400 MHz, toluene-$d_8$, 298 K) of a) in-situ formed **2-Dy$^{Ph}$**, b) **2-Tb$^{Ph}$**, c) in-situ formed 1:1 mixture of **2-Tb$^{Ph}$: 2-Dy$^{Ph}$**.

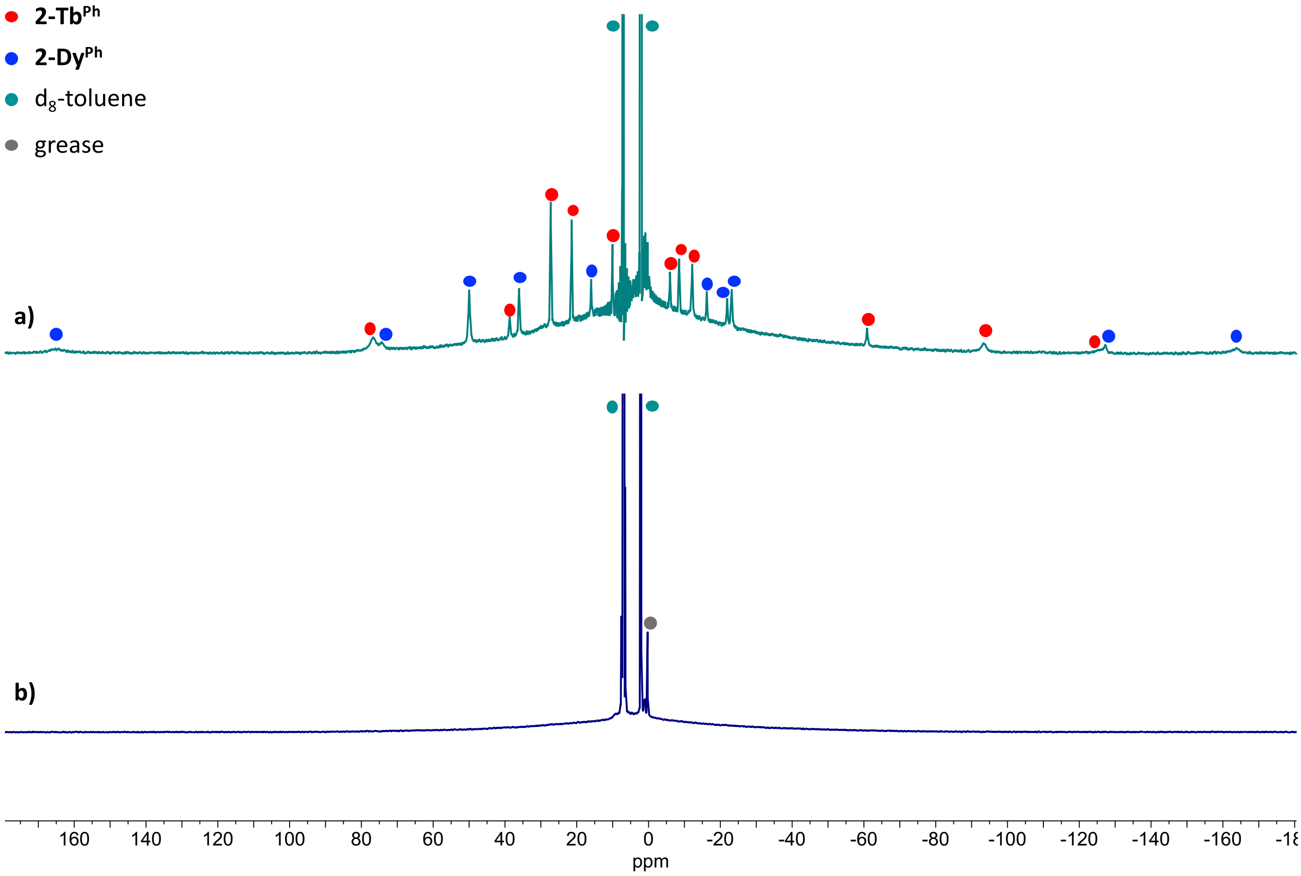


**Figure S44**. $^{1}$H NMR spectrum (400 MHz, toluene-$d_8$, 298 K) of a) in-situ formed 1:1 mixture of **2-Tb$^{Ph}$: 2-Dy$^{Ph}$** b) Toluene solution obtained after extraction of the precipitate formed after oxidation of the in-situ formed 1:1 mixture of **2-Tb$^{Ph}$: 2-Dy$^{Ph}$**.

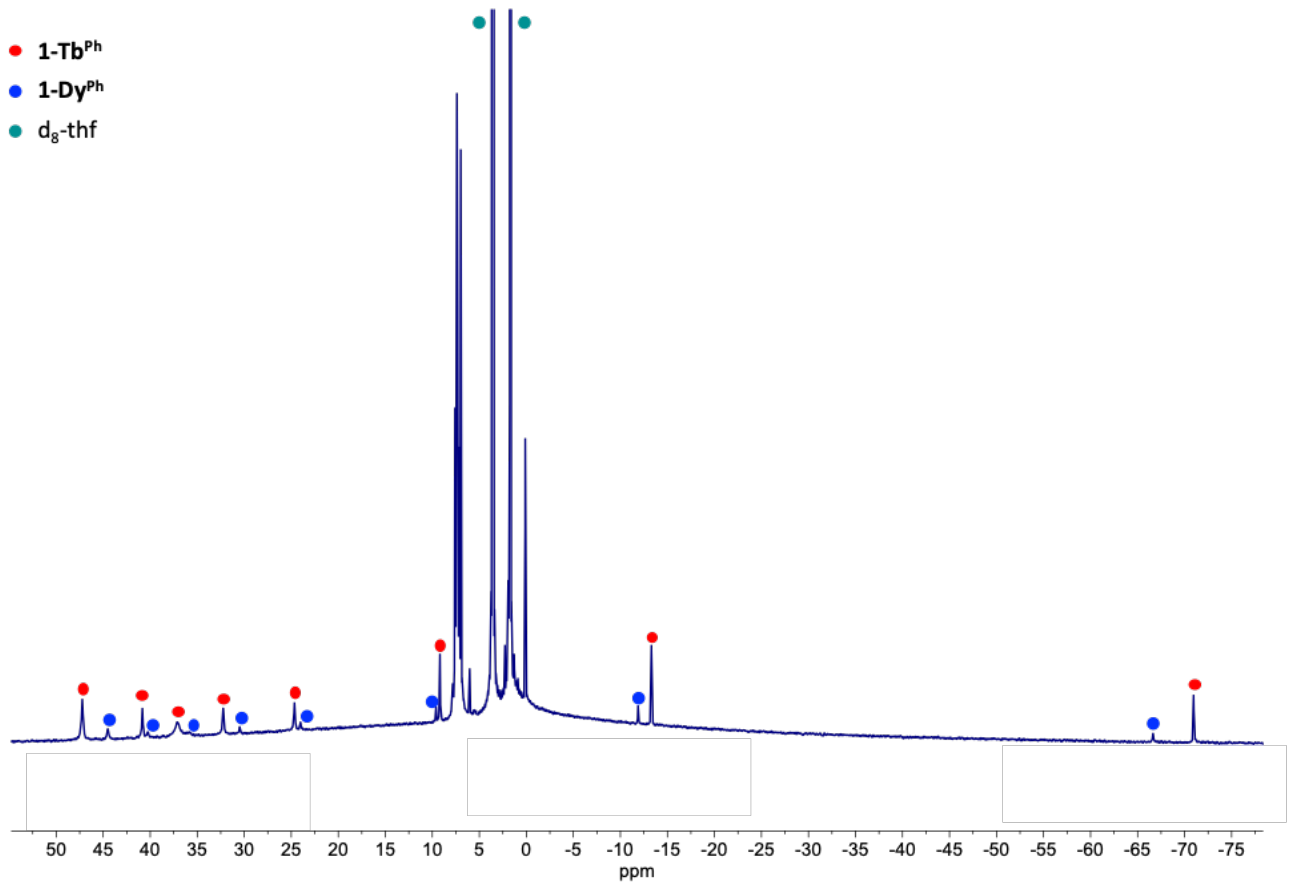


**Figure S45**. $^{1}$H NMR spectrum (400 MHz, THF-*d*8, 298 K) of the residue after oxidation of the in-situ formed 1:1 mixture of **2-Tb$^{Ph}$: 2-Dy$^{Ph}$** and extraction in toluene, in THF. The signals of **1-Dy$^{Ph}$** and **1-Tb$^{h}$** after 14 hours (quantitative integration of **1-Dy$^{Ph}$** showed that only 6% is left in the extracted solid.

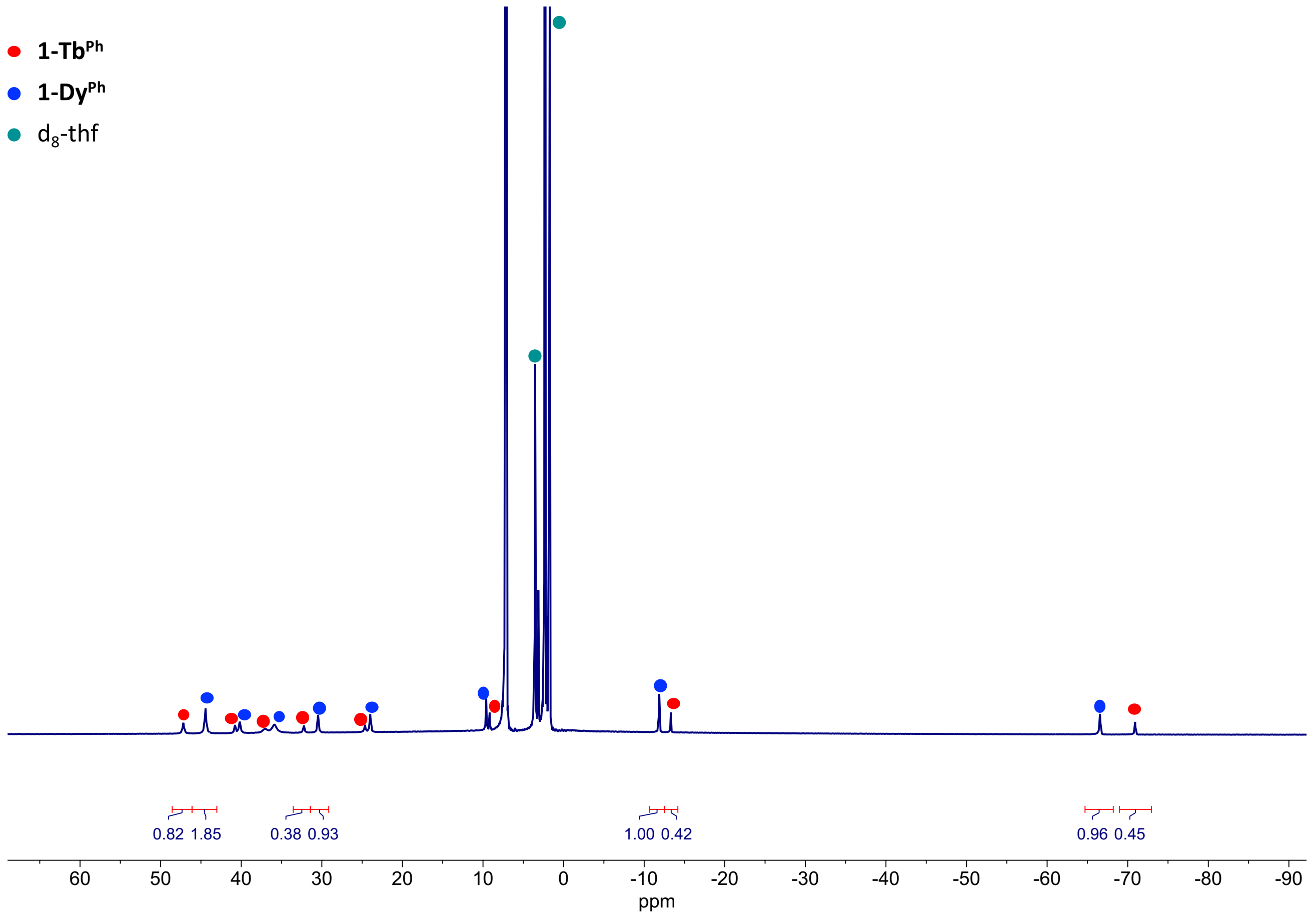


**Figure S46**. $^1$H NMR spectrum (400 MHz, THF-$d_8$, 298 K) of the insoluble material left from the toluene extraction, after 14h in THF. The Tb:Dy ratio is 0.44:1 indicating a higher concentration of Dy in the insoluble material.

## S4. X-ray Crystallography Data

**Table S1.** Crystal data and structural refinement parameters for complexes **1-Tb$^{OtBu}$**, **1-Ce$^{Ph}$·2(toluene)**, **1-Tb$^{Ph}$·2(toluene)**, **1-Pr$^{Ph}$·2(toluene)**

| | **1-Tb$^{OtBu}$** | **1-Ce$^{Ph}$·2(toluene)** | **1-Tb$^{Ph}$·2(toluene)** | **1-Pr$^{Ph}$·2(toluene)** |
|---|---|---|---|---|
| Formula | $C_{52}H_{77}O_{10}Si_3Tb$ | $C_{86}H_{85}CeO_6Si_3$ | $C_{86}H_{85}O_6Si_3Tb$ | $C_{86}H_{85}O_6PrSi_3$ |
| $D_{calc.}$/ g cm$^{-3}$ | 1.331 | 1.289 | 1.338 | 1.294 |
| $\mu$/mm$^{-1}$ | 7.35 | 5.623 | 5.704 | 5.938 |
| Formula Weight | 1105.32 | 1438.92 | 1457.72 | 1439.71 |
| Colour | clear pale colourless | clear pale colourless | clear pale colourless | clear pale colourless |
| Shape | irregular-shaped | prism-shaped | prism-shaped | prism-shaped |
| Size/mm$^3$ | 0.16×0.09×0.06 | 0.29×0.11×0.09 | 0.21×0.06×0.05 | 0.12×0.11×0.06 |
| $T$/K | 140.01(10) | 200.00(11) | 140.00(10) | 200.00(10) |
| Crystal System | triclinic | triclinic | triclinic | triclinic |
| Space Group | $P$-1 | $P$-1 | $P$-1 | $P$-1 |
| $a$/Å | 13.9674(4) | 11.2033(3) | 11.1371(2) | 11.1967(4) |
| $b$/Å | 18.1707(4) | 14.3534(3) | 14.2669(3) | 14.3423(5) |
| $c$/Å | 21.7736(4) | 24.5094(5) | 24.2013(5) | 24.4587(6) |
| $\alpha$/° | 89.0799(15) | 106.2354(18) | 106.0855(17) | 106.237(3) |
| $\beta$/° | 88.765(2) | 99.3584(18) | 99.4980(18) | 99.362(3) |
| $\gamma$/° | 87.017(2) | 93.8891(17) | 93.6846(16) | 93.699(3) |
| V/Å$^3$ | 5516.7(2) | 3706.92(14) | 3619.57(13) | 3695.7(2) |
| $Z$ | 4 | 2 | 2 | 2 |
| $Z'$ | 2 | 1 | 1 | 1 |
| Wavelength/Å | 1.54184 | 1.54184 | 1.54184 | 1.54184 |
| Radiation type | Cu K$_{\alpha}$ | Cu K$_{\alpha}$ | Cu K$_{\alpha}$ | Cu K$_{\alpha}$ |
| $\Theta_{min}$/° | 3.148 | 3.23 | 3.246 | 3.231 |
| $\Theta_{max}$/° | 72.726 | 77.152 | 72.633 | 72.48 |
| Measured Refl's. | 45368 | 40854 | 28022 | 32715 |
| Indep't Refl's | 21318 | 15358 | 13994 | 14258 |
| Refl's I≥2 $\sigma$(I) | 16912 | 13941 | 13011 | 13154 |
| $R_{int}$ | 0.0435 | 0.0401 | 0.0333 | 0.0257 |
| Parameters | 1293 | 933 | 964 | 942 |
| Restraints | 230 | 454 | 420 | 418 |
| Largest Peak | 1.043 | 0.875 | 0.678 | 0.63 |
| Deepest Hole | -1.382 | -0.512 | -0.946 | -0.342 |
| GooF | 1.018 | 1.011 | 1.029 | 1.021 |
| $wR_2$ (all data) | 0.1072 | 0.0947 | 0.0786 | 0.0692 |
| $wR_2$ | 0.0986 | 0.0891 | 0.0762 | 0.0668 |
| $R_1$ (all data) | 0.0585 | 0.0421 | 0.0356 | 0.0312 |
| $R_1$ | 0.0421 | 0.0363 | 0.0316 | 0.027 |
| Flack Parameter | / | / | / | / |
| Hooft Parameter | / | / | / | / |

**Table S2.** Crystal data and structural refinement parameters for complexes **2-Ce$^{OtBu}$·0.9(THF)**, **2-Tb$^{OtBu}$·0.9(THF)**, **2-Tb$^{Ph}$·0.5(toluene)**, **2-Pr$^{Ph}$-MeCN·4(MeCN)**

| | **2-Ce$^{OtBu}$·0.9(THF)** | **2-Tb$^{OtBu}$·0.9(THF)** | **2-Tb$^{Ph}$·0.5(toluene)** | **2-Pr$^{Ph\text{-}MeCN}$·4(MeCN)** |
|---|---|---|---|---|
| Formula | $C_{88}H_{128}CeKO_{15.5}Si_4$ | $C_{98.8}H_{149.6}KO_{18.2}Si_4Tb$ | $C_{88.5}H_{72}KO_4Si_4Tb$ | $C_{92}H_{81}KN_7O_4PrSi_4$ |
| $D_{calc.}$/ g cm$^{-3}$ | 1.079 | 1.221 | 1.346 | 1.316 |
| $\mu$/mm$^{-1}$ | 4.508 | 4.568 | 6.186 | 5.951 |
| Formula Weight | 1725.48 | 1938.96 | 1509.921 | 1641 |
| Colour | clear pale colourless | clear pale colourless | lustrous pale colourless | clear pale colourless |
| Shape | prism-shaped | irregular-shaped | irregular-shaped | prism-shaped |
| Size/mm$^3$ | 0.41×0.28×0.23 | 0.29×0.16×0.08 | 0.22×0.14×0.12 | 0.28×0.09×0.08 |
| $T$/K | 200.00(11) | 199.99(12) | 200.00(11) | 139.99(10) |
| Crystal System | hexagonal | hexagonal | triclinic | monoclinic |
| Space Group | $P6_322$ | $P6_322$ | $P$-1 | $P2_1/n$ |
| $a$/Å | 14.87393(16) | 14.7864(3) | 14.0838(14) | 13.65640(10) |
| $b$/Å | 14.87393(16) | 14.7864(3) | 14.2282(17) | 43.2742(5) |
| $c$/Å | 55.4259(7) | 55.7285(10) | 21.775(3) | 14.24030(10) |
| $\alpha$/° | 90 | 90 | 77.38(1) | 90 |
| $\beta$/° | 90 | 90 | 74.179(9) | 100.1850(10) |
| $\gamma$/° | 120 | 120 | 63.234(11) | 90 |
| V/Å$^3$ | 10619.3(3) | 10551.9(4) | 3724.9(8) | 8282.97(13) |
| $Z$ | 4 | 4 | 2 | 4 |
| $Z'$ | 0.333333 | 0.333333 | 1 | 1 |
| Wavelength/Å | 1.54184 | 1.54184 | 1.54184 | 1.54184 |
| Radiation type | Cu K$_\alpha$ | Cu K$_\alpha$ | Cu K$_\alpha$ | Cu K$_\alpha$ |
| $\Theta_{min}$/° | 3.189 | 3.172 | 3.5 | 3.314 |
| $\Theta_{max}$/° | 72.678 | 72.734 | 73 | 75.748 |
| Measured Refl's. | 86994 | 58647 | 29995 | 84003 |
| Indep't Refl's | 7038 | 6978 | 14275 | 16596 |
| Refl's I≥2 $\sigma$(I) | 6906 | 6285 | 9012 | 14190 |
| $R_{int}$ | 0.0379 | 0.0585 | 0.0757 | 0.0684 |
| Parameters | 400 | 394 | 985 | 906 |
| Restraints | 551 | 484 | 630 | 0 |
| Largest Peak | 0.909 | 0.569 | 1.225 | 1.523 |
| Deepest Hole | -0.6 | -0.689 | -1.2251 | -1.476 |
| GooF | 1.088 | 1.074 | 1.0844 | 1.02 |
| $wR_2$ (all data) | 0.1561 | 0.1747 | 0.2509 | 0.161 |
| $wR_2$ | 0.1554 | 0.1709 | 0.2142 | 0.1539 |
| $R_1$ (all data) | 0.0588 | 0.0751 | 0.1308 | 0.0693 |
| $R_1$ | 0.0581 | 0.0695 | 0.085 | 0.0597 |
| Flack Parameter | 0.071(10) | 0.429(12) | / | / |
| Hooft Parameter | -0.0349(11) | -0.007(2) | / | / |

**Table S3.** Crystal data and structural refinement parameters for complexes **3-Ce$^{OtBu}$·3.5(toluene)**, **3-Ce$^{Ph}$·4(THF)**, **3-Tb$^{Ph}$·2.5(MeCN)** and **1-Tb$^{Ph}$-CH$_3$CN**

| | **3-Ce$^{OtBu}$·3.5(toluene)** | **3-Ce$^{Ph}$·4(THF)** | **3-Tb$^{Ph}$·2.5(MeCN)** | **1-Tb$^{Ph}$-CH$_3$CN** |
|---|---|---|---|---|
| Formula | $C_{90.5}H_{112}CeO_{10}Si_4$ | $C_{102}H_{108}CeO_{10}Si_4$ | $C_{87}H_{73.5}N_{4.5}O_4Si_4Tb$ | $C_{66}H_{54}N_3O_3Si_3Tb$ |
| $D_{calc.}$/ g cm$^{-3}$ | 1.243 | 1.294 | 1.296 | 1.38 |
| $\mu$/mm$^{-1}$ | 5.052 | 4.902 | 5.474 | 7.113 |
| Formula Weight | 1612.27 | 1746.36 | 1517.28 | 1180.31 |
| Colour | clear light yellow | clear pale colourless | clear dark orange | clear light yellow |
| Shape | plate-shaped | irregular-shaped | prism-shaped | prism-shaped |
| Size/mm$^3$ | 0.17×0.12×0.01 | 0.31×0.20×0.07 | 0.31×0.21×0.19 | 0.20×0.15×0.12 |
| $T$/K | 140.00(10) | 139.99(10) | 200.00(10) | 140.00(10) |
| Crystal System | triclinic | orthorhombic | monoclinic | monoclinic |
| Space Group | $P$-1 | $P2_12_12_1$ | $P2_1/c$ | $P2_1/n$ |
| $a$/Å | 14.49307(15) | 15.94855(11) | 17.6194(3) | 13.79296(19) |
| $b$/Å | 14.67810(14) | 22.45159(16) | 13.8469(3) | 22.3194(2) |
| $c$/Å | 20.2591(3) | 25.03370(19) | 31.8914(5) | 18.4603(2) |
| $\alpha$/° | 89.6876(9) | 90 | 90 | 90 |
| $\beta$/° | 88.7540(10) | 90 | 91.3763(14) | 91.7441(12) |
| $\gamma$/° | 89.3655(8) | 90 | 90 | 90 |
| V/Å$^3$ | 4308.40(8) | 8963.82(11) | 7778.4(2) | 5680.36(12) |
| $Z$ | 2 | 4 | 4 | 4 |
| $Z'$ | 1 | 1 | 1 | 1 |
| Wavelength/Å | 1.54184 | 1.54184 | 1.54184 | 1.54184 |
| Radiation type | Cu K$_\alpha$ | Cu K$_\alpha$ | Cu K$_\alpha$ | Cu K$_\alpha$ |
| $\Theta_{min}$/° | 3.011 | 3.286 | 2.772 | 3.107 |
| $\Theta_{max}$/° | 74.677 | 72.628 | 72.539 | 74.747 |
| Measured Refl's. | 69611 | 39009 | 42724 | 57303 |
| Indep't Refl's | 16949 | 17348 | 15006 | 11268 |
| Refl's I≥2$\sigma$(I) | 15368 | 16665 | 11807 | 9232 |
| $R_{int}$ | 0.0278 | 0.0272 | 0.0369 | 0.037 |
| Parameters | 1146 | 1035 | 868 | 784 |
| Restraints | 1139 | 534 | 36 | 421 |
| Largest Peak/e Å$^{-3}$ | 0.753 | 0.592 | 0.73 | 1.732 |
| Deepest Hole/e Å$^{-3}$ | -0.59 | -0.451 | -0.467 | -1.125 |
| GooF | 1.022 | 1.03 | 1.068 | 1.035 |
| $wR_2$ (all data) | 0.0887 | 0.1097 | 0.1069 | 0.1472 |
| $wR_2$ | 0.0863 | 0.1073 | 0.0997 | 0.1396 |
| $R_1$ (all data) | 0.0408 | 0.0431 | 0.0555 | 0.065 |
| $R_1$ | 0.0354 | 0.0409 | 0.0412 | 0.0516 |
| Flack Parameter | / | -0.0133(17) | / | / |
| Hooft Parameter | / | -0.013(2) | / | / |

**Table S4.** Crystal data and structural refinement parameters for complex **2-Tb$^{Ph}$-MeCN·(Et$_2$O)**

| | **2-Tb$^{Ph}$-MeCN·(Et$_2$O)** |
|---|---|
| Formula | $C_{168}H_{142}K_2N_4O_9Si_8Tb_2$ |
| $D_{calc.}$/ g cm$^{-3}$ | 1.391 |
| $\mu$/mm$^{-1}$ | 6.477 |
| Formula Weight | 2981.61 |
| Colour | clear pale colourless |
| Shape | prism |
| Size/mm$^3$ | 0.22×0.13×0.09 |
| *T*/K | 139.99(10) |
| Crystal System | triclinic |
| Space Group | *P*-1 |
| *a*/Å | 14.6865(6) |
| *b*/Å | 15.3137(6) |
| *c*/Å | 18.9586(6) |
| $\alpha$/° | 87.191(3) |
| $\beta$/° | 67.976(3) |
| $\gamma$/° | 65.256(4) |
| V/Å$^3$ | 3560.2(3) |
| *Z* | 1 |
| *Z'* | 0.5 |
| Wavelength/Å | 1.54184 |
| Radiation type | Cu $K_\alpha$ |
| $\Theta_{min}$/° | 2.535 |
| $\Theta_{max}$/° | 72.469 |
| Measured Refl's. | 28996 |
| Indep't Refl's | 13709 |
| Refl's I≥2$\sigma$(I) | 12635 |
| $R_{int}$ | 0.0322 |
| Parameters | 849 |
| Restraints | 0 |
| Largest Peak/e Å$^{-3}$ | 0.634 |
| Deepest Hole/e Å$^{-3}$ | -0.699 |
| GooF | 1.018 |
| $wR_2$ (all data) | 0.0871 |
| $wR_2$ | 0.0841 |
| $R_1$ (all data) | 0.0378 |
| $R_1$ | 0.0335 |
| Flack Parameter | / |
| Hooft Parameter | / |

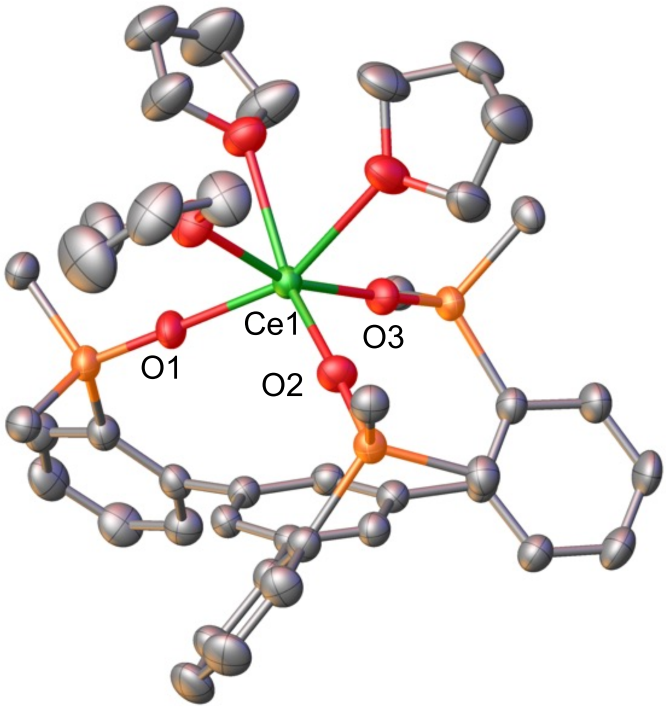


**Figure S47**. Molecular structure of complex, [$Ce^{III}$((OSiPh$_2$Ar)$_3$-arene)(THF)$_3$], **1-Ce$^{Ph}$** with thermal ellipsoids drawn at the 50% probability level. Hydrogen atoms, some disordered substituents and the five carbon atoms of each phenyl groups have been omitted for clarity.

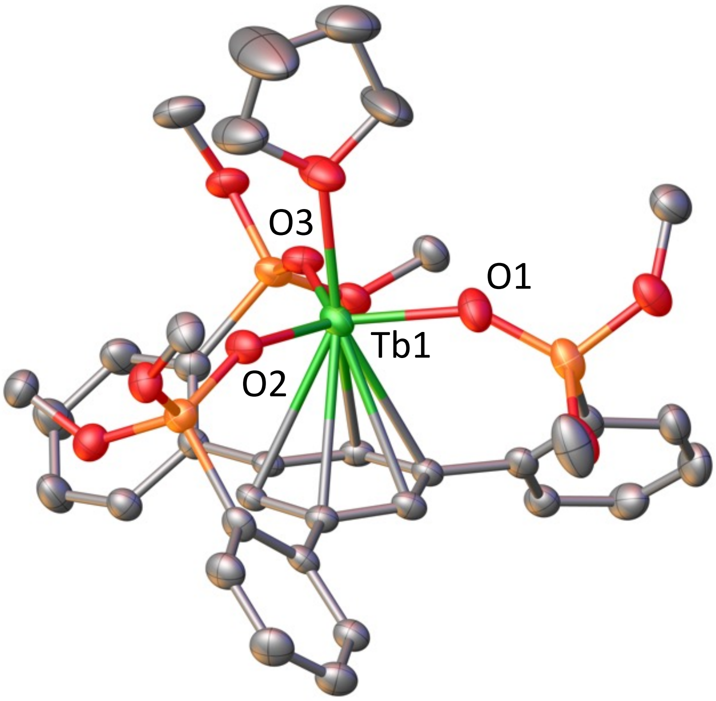


**Figure S48**. Molecular structure of complex, [$Tb^{III}$((OSi(O$^t$Bu)$_2$Ar)$_3$-arene)(THF)], **1-Tb$^{OtBu}$** with thermal ellipsoids drawn at the 50% probability level. Hydrogen atoms, some disordered substituents and the methyl groups on the –O(SiO$^t$Bu)$_2$ arms have been omitted for clarity.

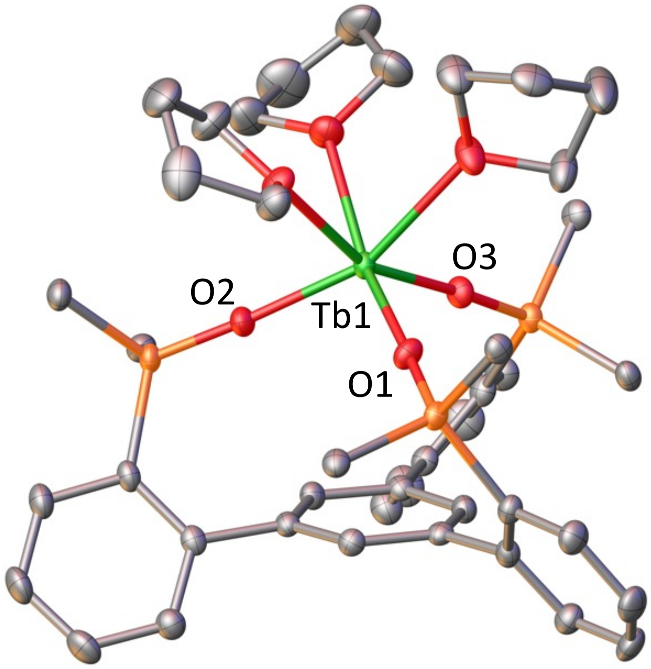


**Figure S49**. Molecular structure of complex, [$Tb^{III}((OSiPh_2Ar)_3$-arene)$(THF)_3$], **1-Tb$^{Ph}$** with thermal ellipsoids drawn at the 50% probability level. Hydrogen atoms, some disordered substituents and the five carbon atoms of each phenyl groups have been omitted for clarity.

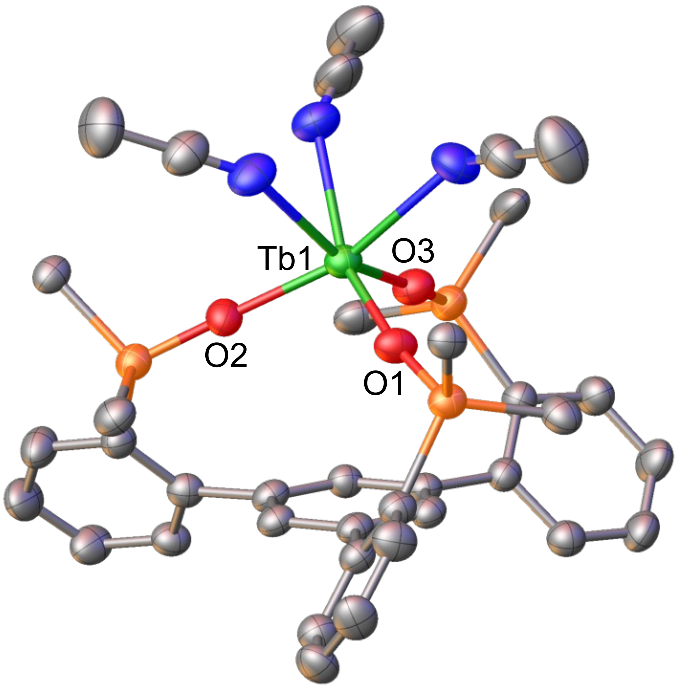


**Figure S50**. Molecular structure of the MeCN adduct of complex **1-Tb$^{Ph}$**, [$Tb^{III}((OSiPh_2Ar)_3$-arene)$(MeCN)_3$], with thermal ellipsoids drawn at the 50% probability level. Hydrogen atoms, some disordered substituents and the five carbon atoms of each phenyl groups have been omitted for clarity. Pertinent bond distances (Å) and angles (°): Tb1–O1: 2.120(3); Tb1–O2: 2.112(3); Tb1–O3: 2.107(3); Tb1–$C_{centroid}$; 3.960(2); O1–Tb1–O2: 102.72(13); O2–Tb1–O3: 102.61(13); O3–Tb1–O1: 103.89(14).

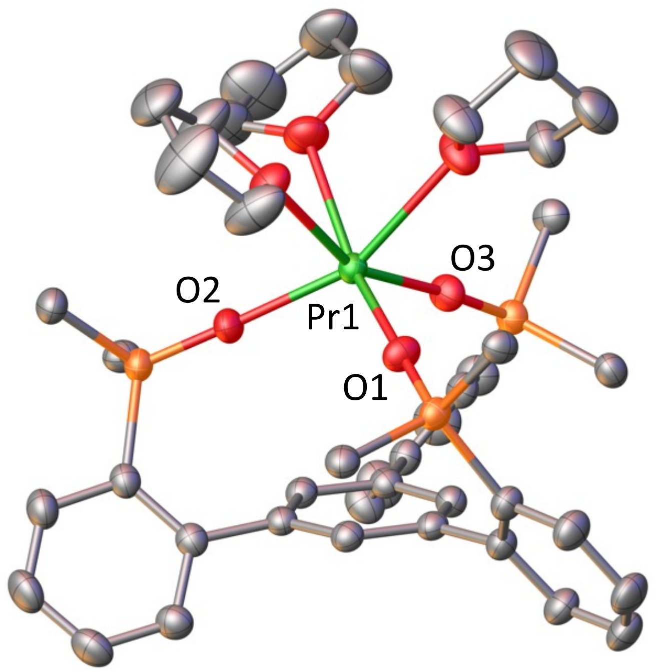


**Figure S51**. Molecular structure of complex, [$Pr^{III}$((OSi$Ph_2$Ar)$_3$-arene)(THF)$_3$], **1-Pr$^{Ph}$** with thermal ellipsoids drawn at the 50% probability level. Hydrogen atoms, some disordered substituents and the five carbon atoms of each phenyl groups have been omitted for clarity.

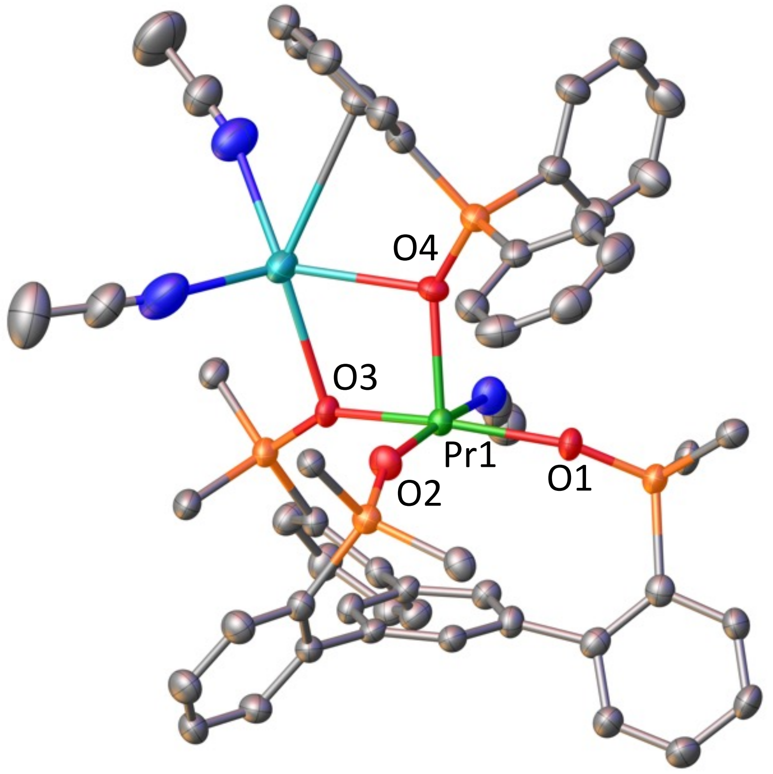


**Figure S52**. Molecular structure of complex, [K(MeCN)$_2${$Pr^{III}$((OSi$Ph_2$Ar)$_3$-arene)(OSi$Ph_3$)(MeCN)}], **2-Pr$^{Ph}$-MeCN·4(MeCN)** with thermal ellipsoids drawn at the 50% probability level. Hydrogen atoms, some disordered substituents and the five carbon atoms of each phenyl groups of the (OSi$Ph_2$Ar)$_3$-arene) ligand have been omitted for clarity.

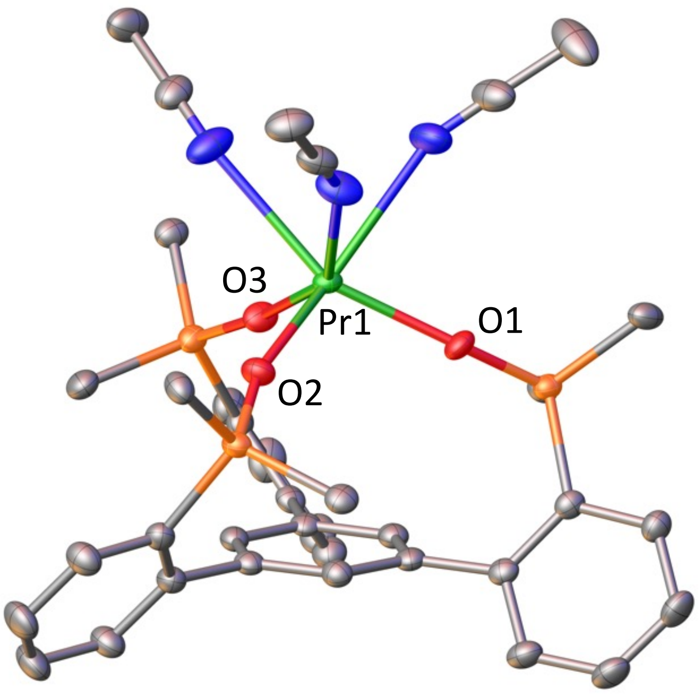


**Figure S53**. Molecular structure of complex [$Pr^{III}$(($OSiPh_2Ar)_3$-arene)$(MeCN)_3$], **1-Pr$^{Ph}$–$CH_3CN$**, with thermal ellipsoids drawn at the 50% probability level. Hydrogen atoms, some disordered substituents and the five carbon atoms of each phenyl groups have been omitted for clarity. Pertinent bond distances (Å) and angles (°): Pr1–O1: 2.202(1); Pr1–O2: 2.197(2); Pr1–O3: 2.191(2); Pr1–$C_{centroid}$; 4.163(1); O1–Pr1–O2: 103.48(8); O2–Pr1–O3: 100.44(9); O3–Pr1–O1: 101.86(8).

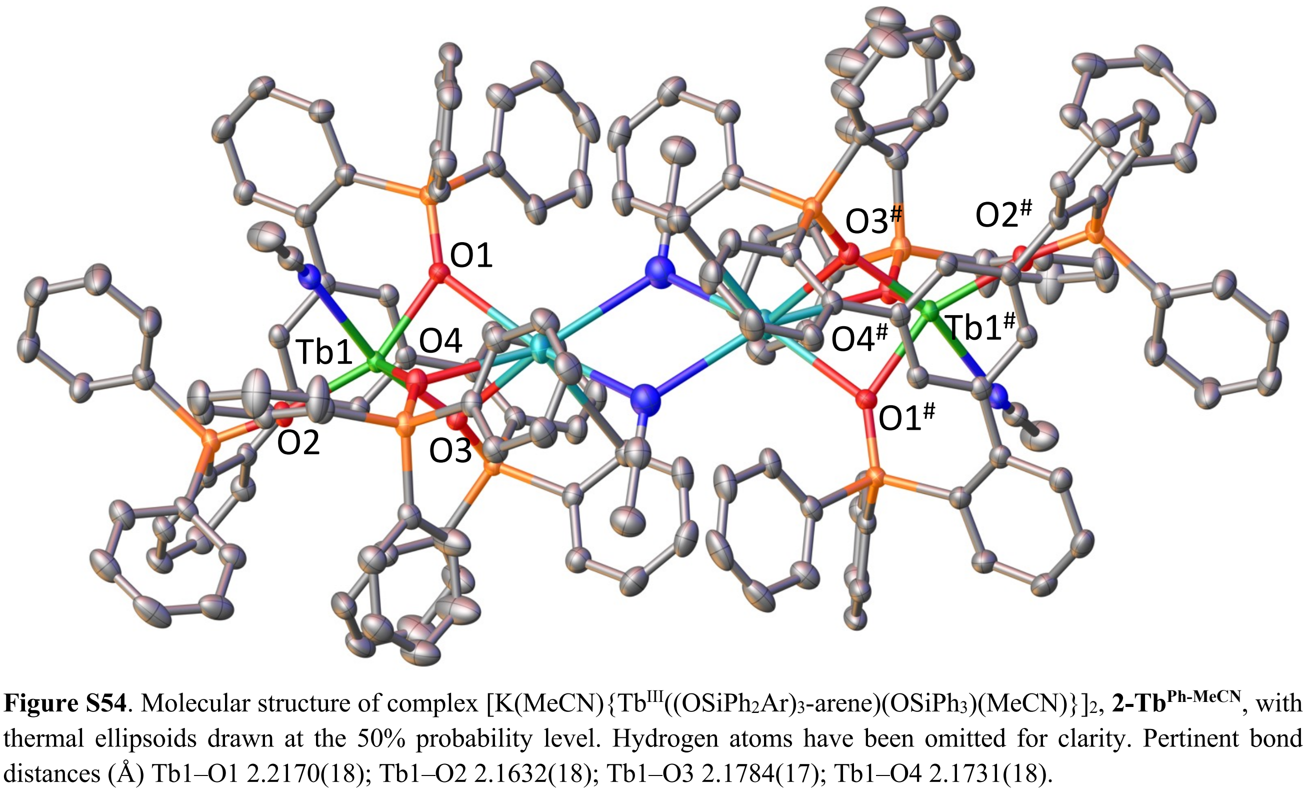


**Figure S54**. Molecular structure of complex [K(MeCN){$Tb^{III}$(($OSiPh_2Ar)_3$-arene)($OSiPh_3$)(MeCN)}$]_2$, **2-Tb$^{Ph\text{-}MeCN}$**, with thermal ellipsoids drawn at the 50% probability level. Hydrogen atoms have been omitted for clarity. Pertinent bond distances (Å) Tb1–O1 2.2170(18); Tb1–O2 2.1632(18); Tb1–O3 2.1784(17); Tb1–O4 2.1731(18).

Complex **2-Tb$^{Ph}$-MeCN·($Et_2O$)** crystalizes in the *P-1* space group. The asymmetric unit is composed of one **2-Tb$^{Ph}$** moiety where which both  K and Tb ions are coordinated by one MeCN molecule each. The overall structure is generated by symmetry and reveals a dimeric form with two MeCN molecules bound to potassium bridging the two **2-Tb$^{Ph}$** fragments.

S5. EPR Data

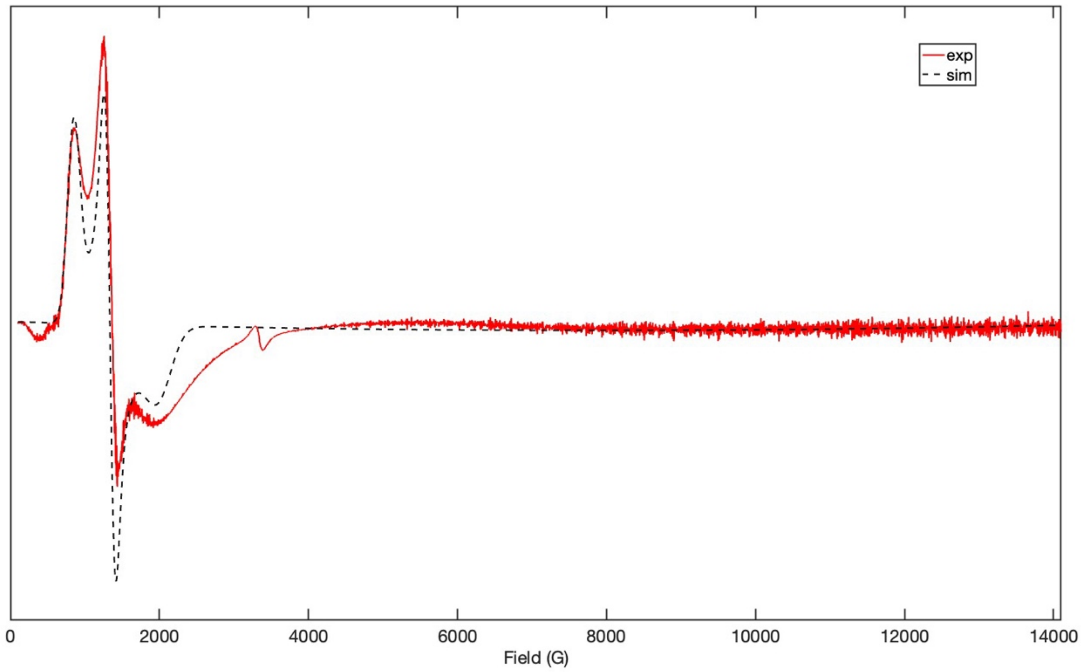


**Figure S55**. X-band (9.4 GHz) EPR spectrum of complex **3-Tb$^{Ph}$** in the solid-state at 6 K (*red line*, experiment; *black dashed line*, fit to 4f$^7$ ion). The plot was fit to a rhombic set of *g*-values [7.90, 5.00, 3.35]

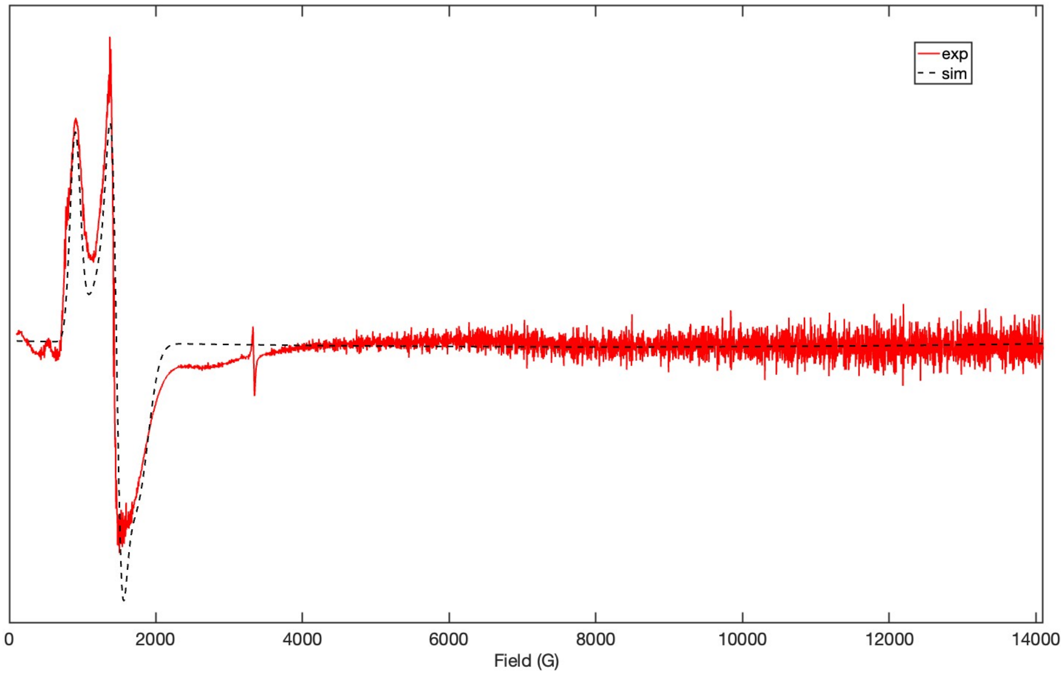


**Figure S56**. X-band (9.4 GHz) EPR spectrum of complex **3-Tb$^{Ph}$** in toluene (20 mM) at 6 K (*red line*, experiment; *black dashed line*, fit to 4f$^7$ ion). The plot was fit to a rhombic set of *g*-values [7.45, 4.55, 3.75]

## S6. Magnetization Data

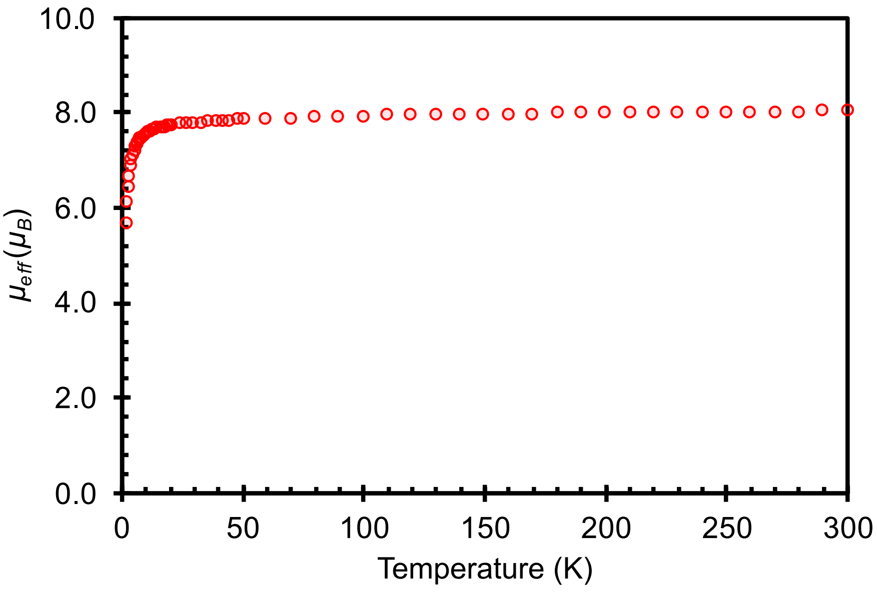


**Figure S57**. Temperature-dependent SQUID magnetization data (1 T) for complex **3-Tb$^{Ph}$**, plotted as the magnetic moment, $\mu_{eff}$.

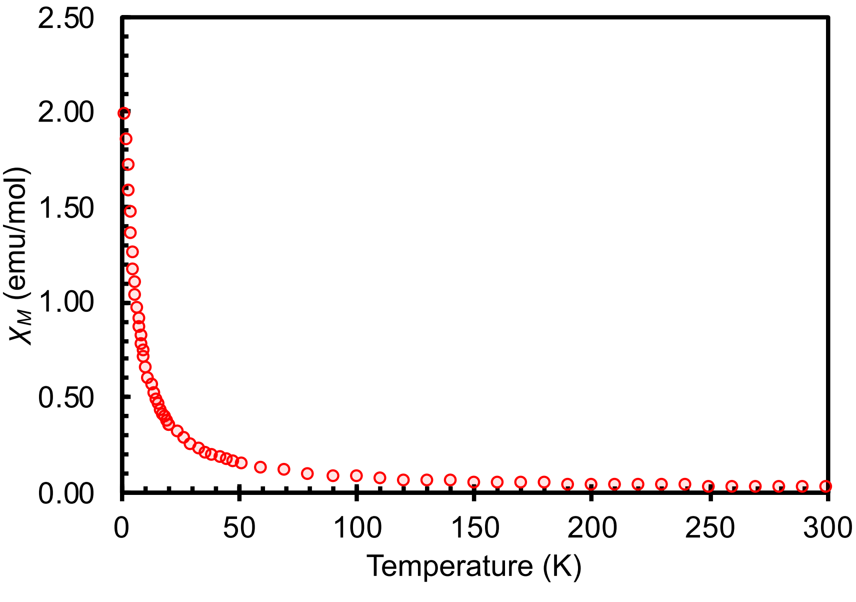


**Figure S58**. Temperature-dependent SQUID magnetization data (1 T) for complex **3-Tb$^{Ph}$**, plotted as the magnetic susceptibility, $\chi_M$.

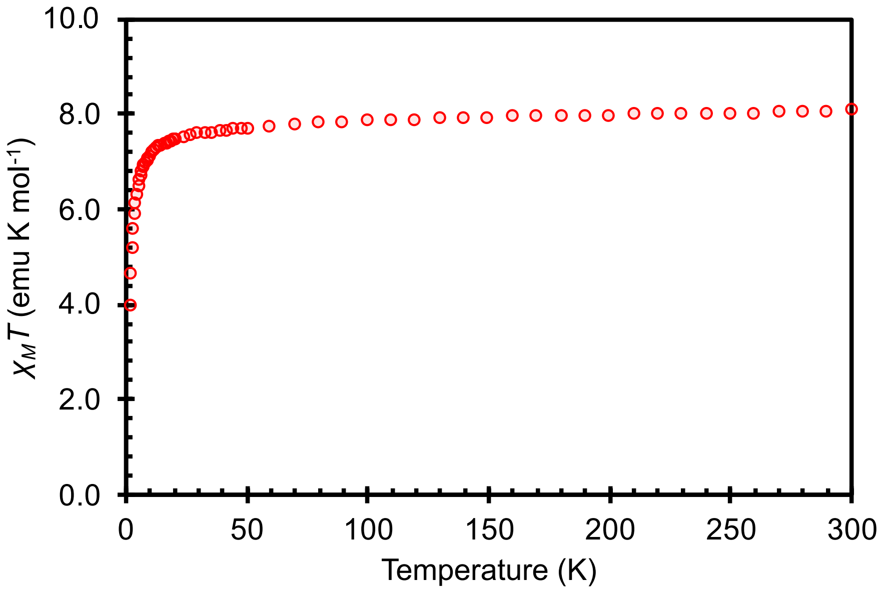


**Figure S59**. Temperature-dependent SQUID magnetization data (1 T) for complex **3-Tb$^{\mathbf{Ph}}$**, plotted as the magnetic susceptibility versus temperature, $\chi_{\mathrm{M}}T$.

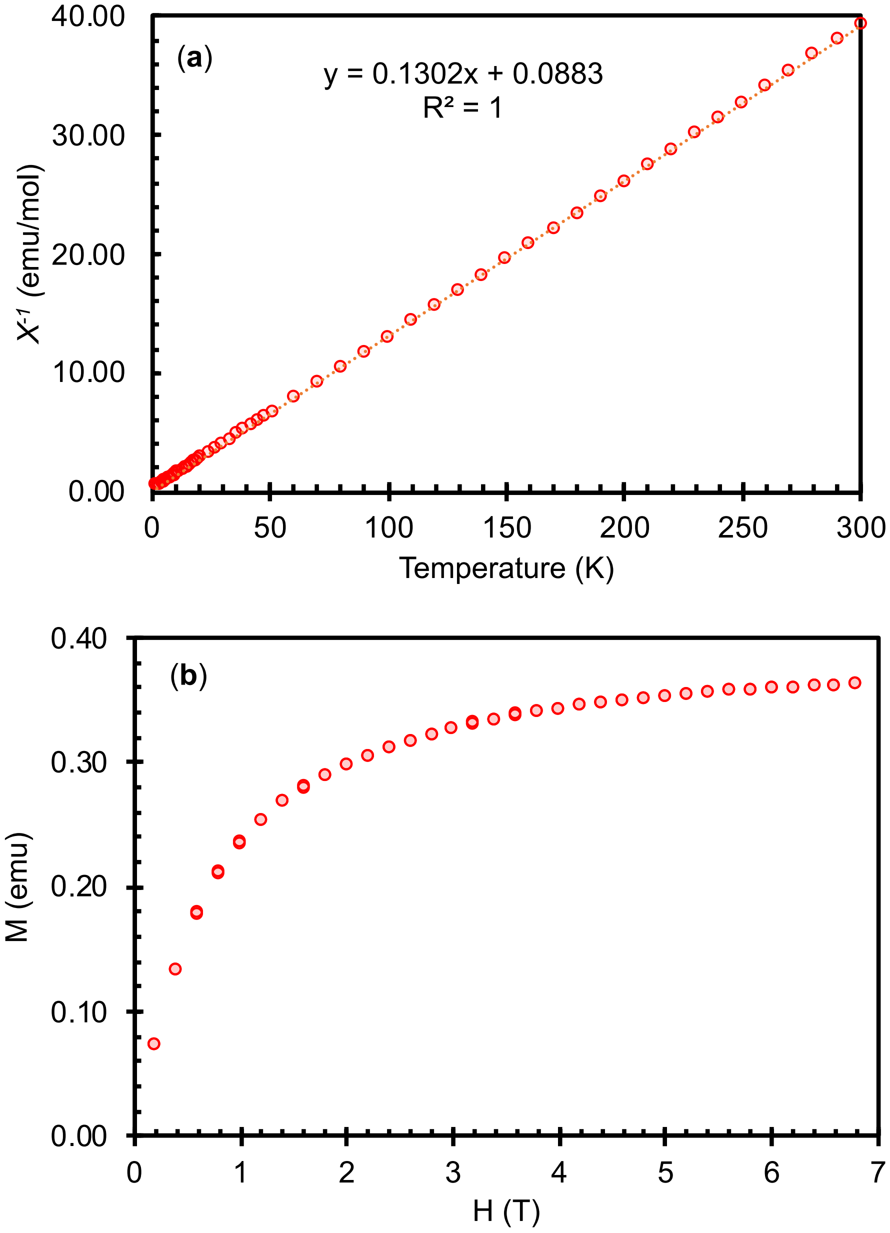


**Figure S60**. (a) Plot of $\chi^{-1}$ versus temperature for **3-Tb$^{Ph}$** under an applied field of 1 T. (b) Magnetization data at 2 K from 0 to 7 T.

**S7. UV/Vis Data**

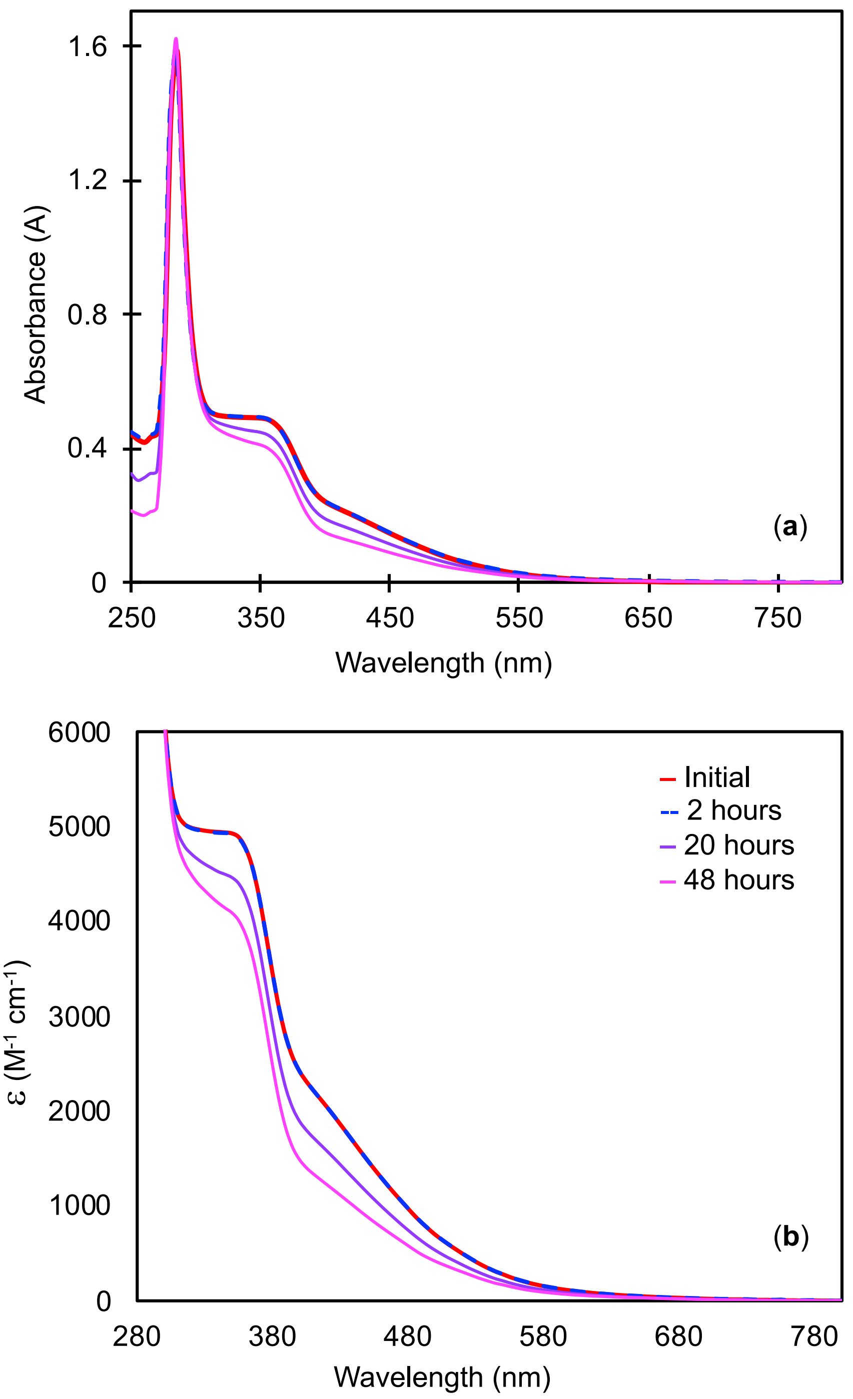


**Figure S61**. (a) UV/Vis spectra for complex **3-Tb$^{Ph}$** (1.0 mM) in toluene (*red*, initial); (*blue-dashed*, after 2 hours); (*purple*, after 20 hours); and (*pink*, after 48 hours. (b) Zoomed-in spectra of the absorption band at $\lambda_{max}$ = 355 nm.

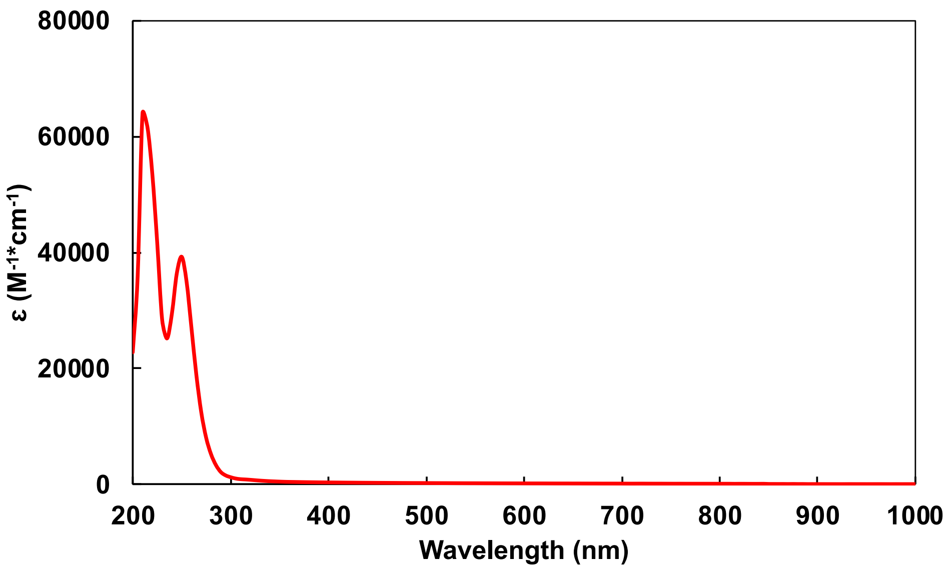


**Figure S62**. UV-Vis spectrum of a 0.4 mM solution of **2-Ce**$^{\mathbf{OtBu}}$ in THF at room temperature.

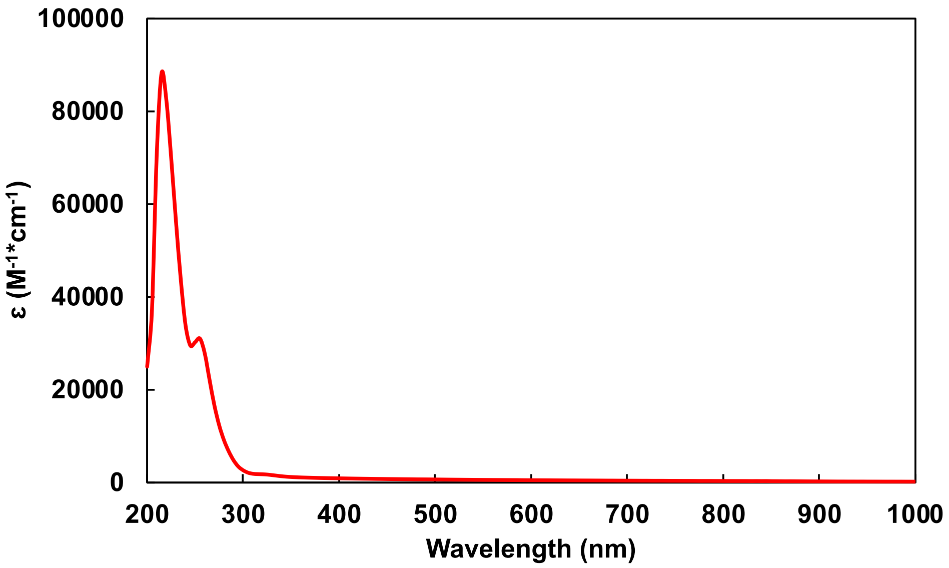


**Figure S63**. UV-Vis spectrum of a 0.4 mM solution of **2-Ce**$^{\mathbf{Ph}}$ in THF at room temperature.

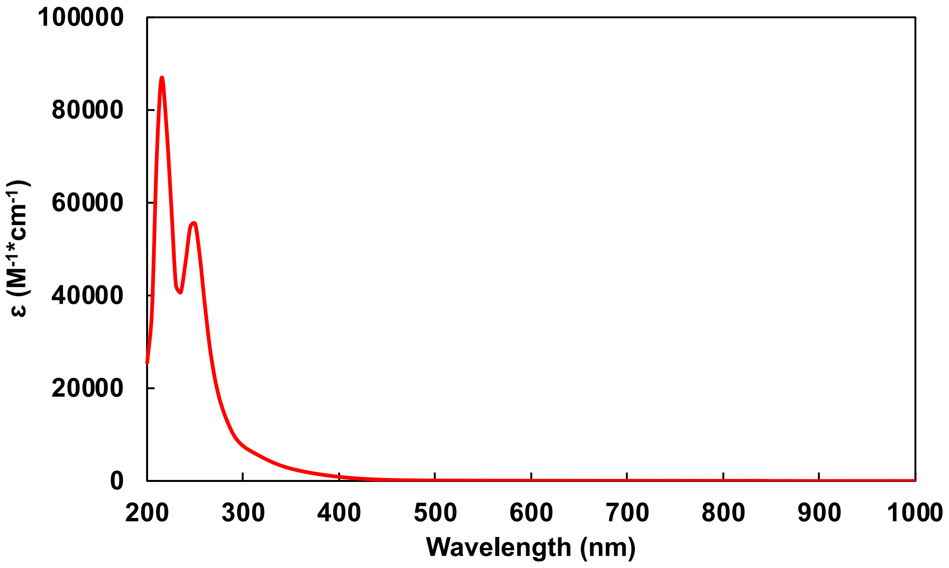


**Figure S64**. UV-Vis spectrum of a 0.4 mM solution of **3-Ce$^{OtBu}$** in THF at room temperature.

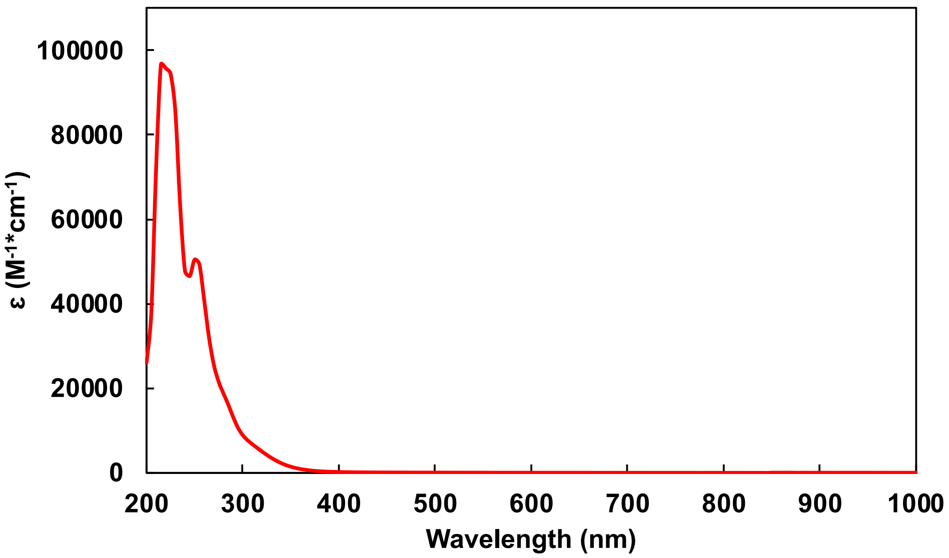


**Figure S65**. UV-Vis spectrum of a 0.4 mM solution of **3-Ce$^{Ph}$** in THF at room temperature.

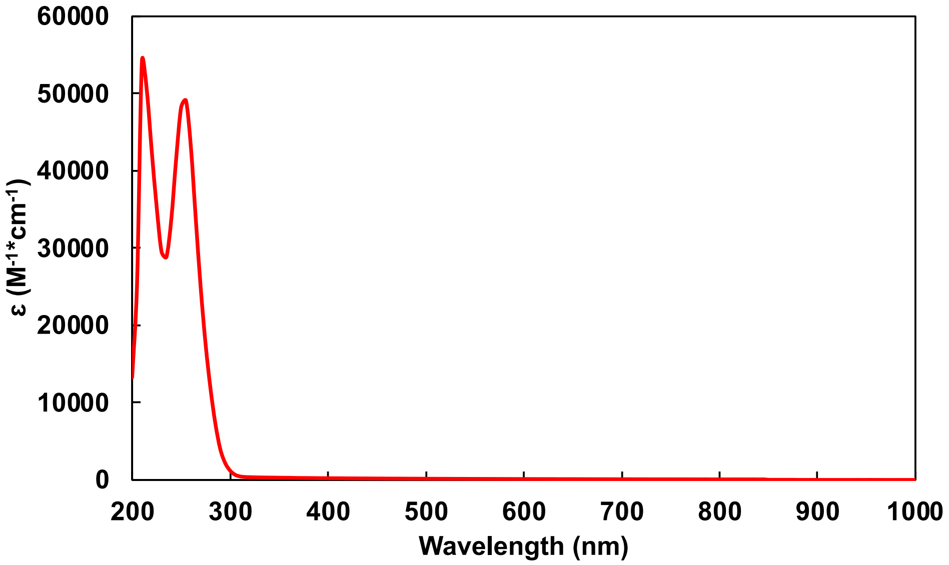


**Figure S66**. UV-Vis spectrum of a 0.4 mM solution of **1-Tb$^{OtBu}$** in THF at room temperature.

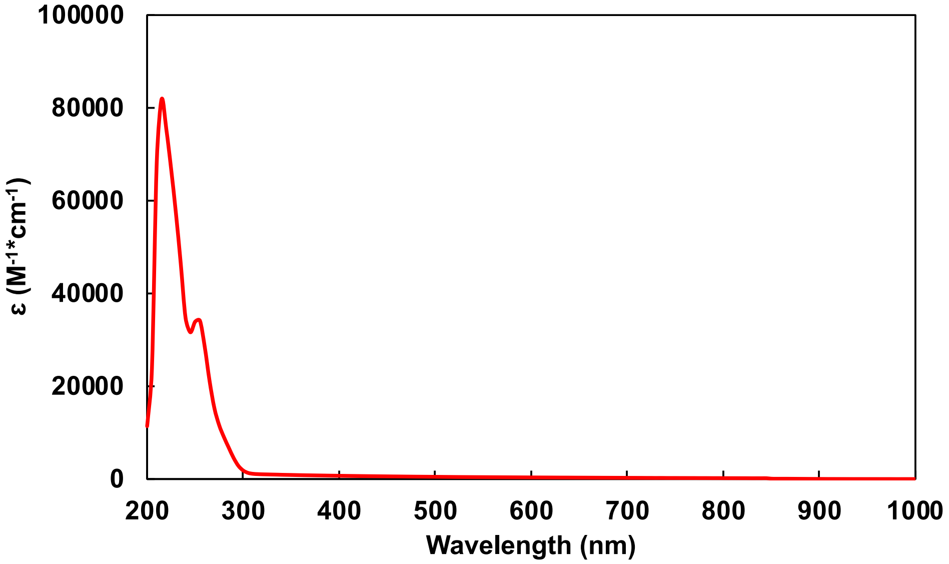


**Figure S67**. UV-Vis spectrum of a 0.4 mM solution of **1-Tb$^{Ph}$** in THF at room temperature.

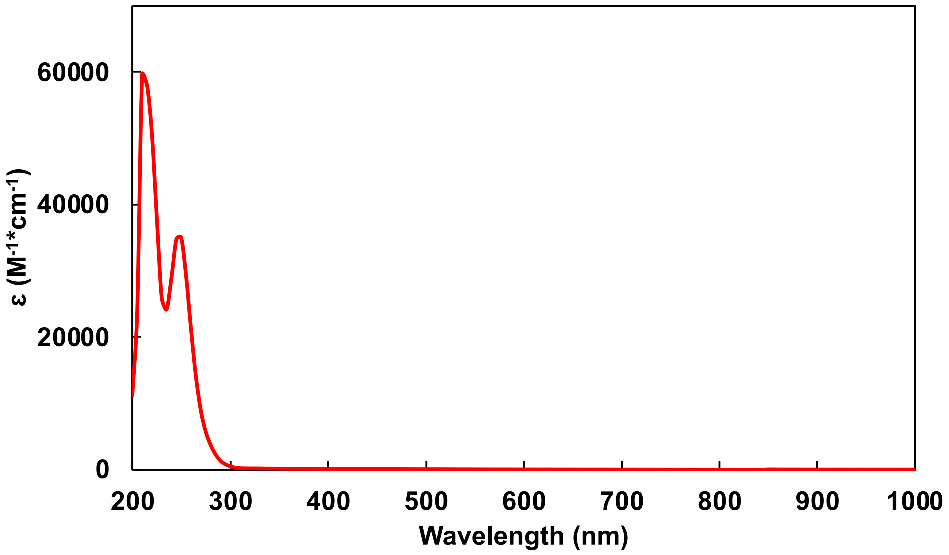


**Figure S68**. UV-Vis spectrum of a 0.4 mM solution of **2-Tb$^{OtBu}$** in THF at room temperature.

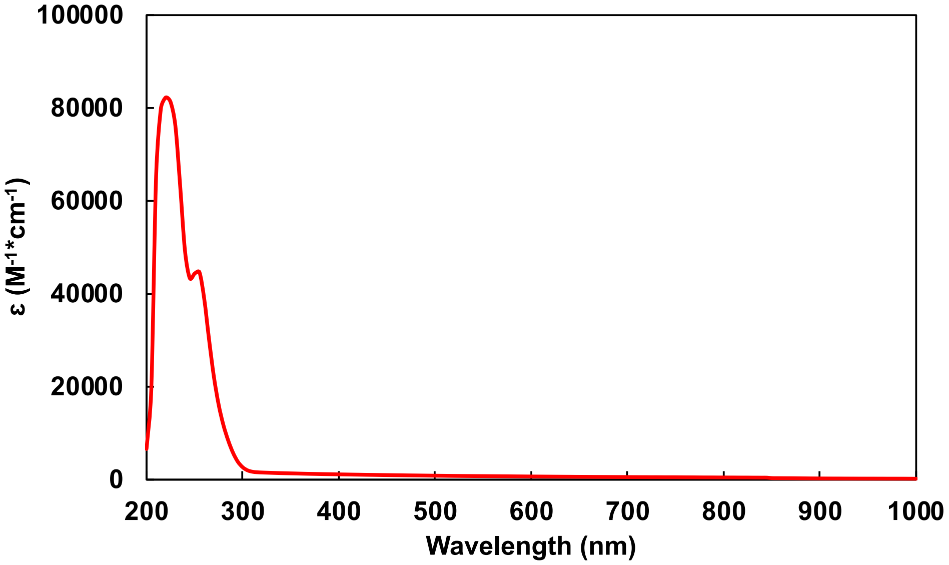


**Figure S69**. UV-Vis spectrum of a 0.4 mM solution of **2-Tb$^{Ph}$** in THF at room temperature.

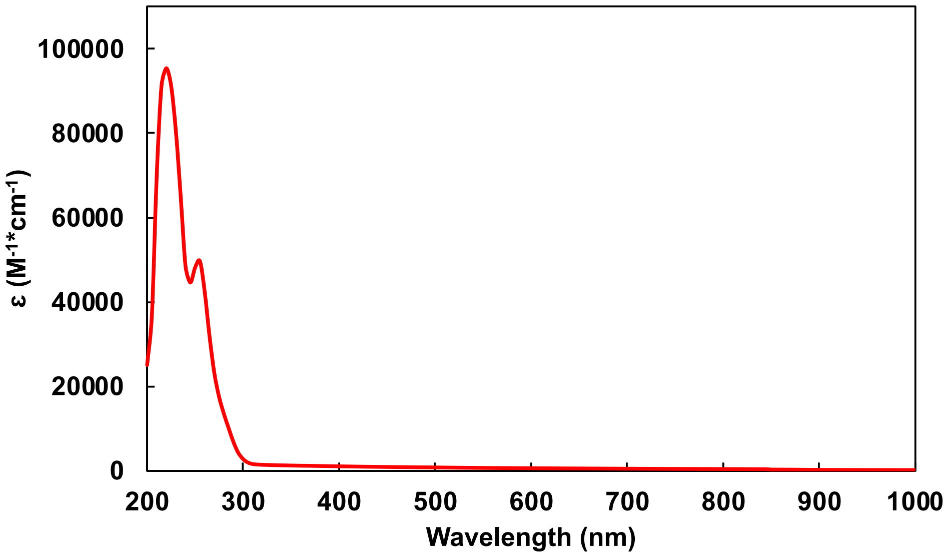


**Figure S70**. UV-Vis spectrum of a 0.4 mM solution of **1-Pr$^{Ph}$** in THF at room temperature.

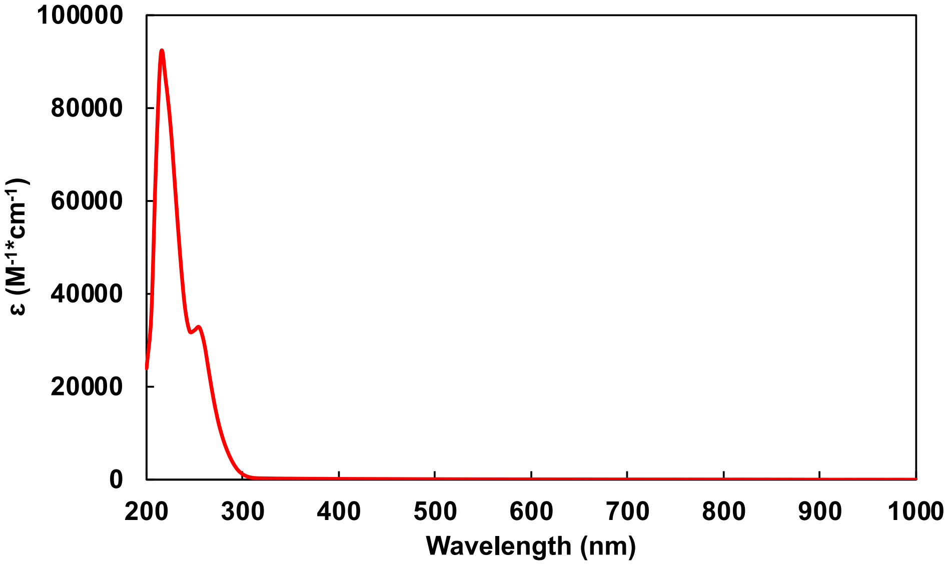


**Figure S71**. UV-Vis spectrum of a 0.4 mM solution of **2-Pr$^{Ph}$** in THF at room temperature.

## S8. Electrochemistry Data

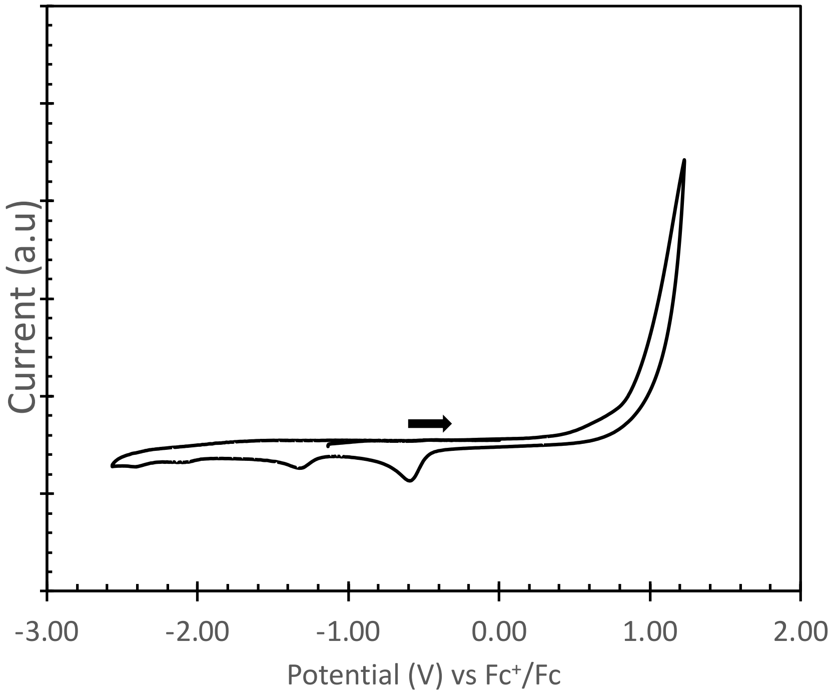


**Figure S72**. Cyclic voltammogram of $NBu_4$ $B(C_6F_5)_4$ electrolyte, in THF at room temperature with a 100mV/s scan rate.

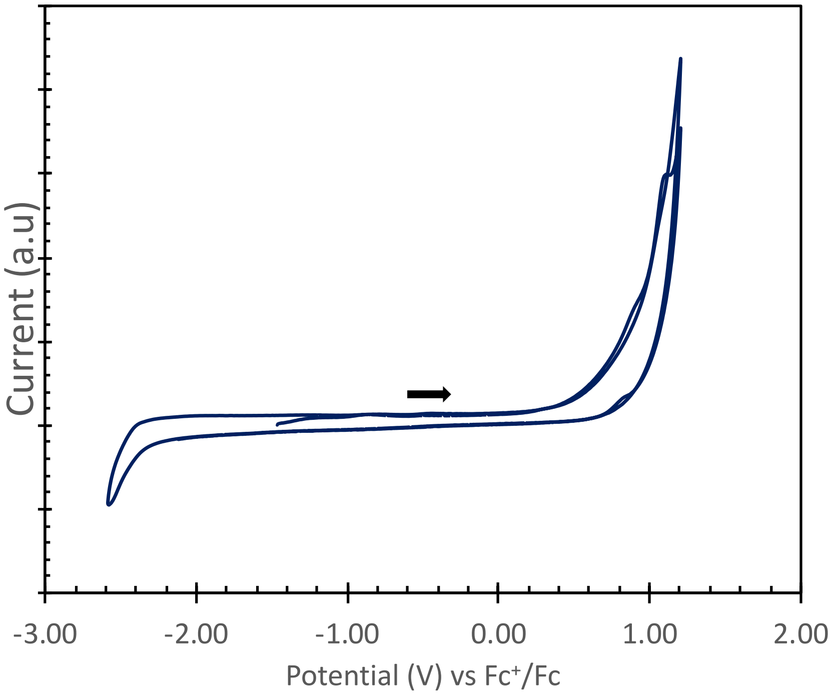


**Figure S73**. Cyclic voltammogram of the deprotonated free ligand $(KOSiPh_2Ar)_3$-arene, in THF at room temperature with a 100mV/s scan rate.

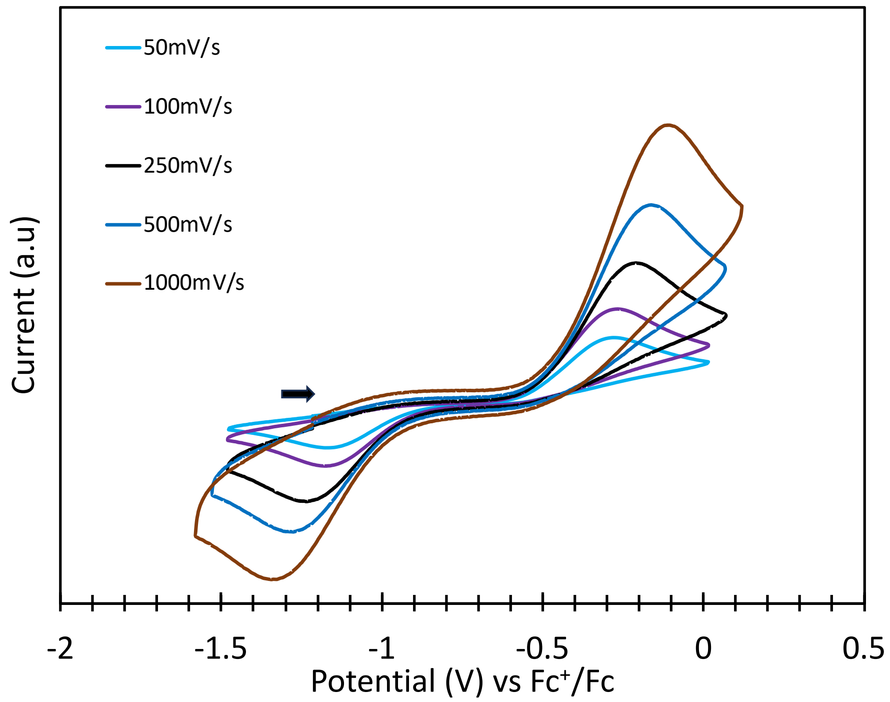


**Figure S74**. Cyclic voltammogram of **2-Ce**$^{\mathbf{OtBu}}$, in THF at room temperature with varying scan rates.

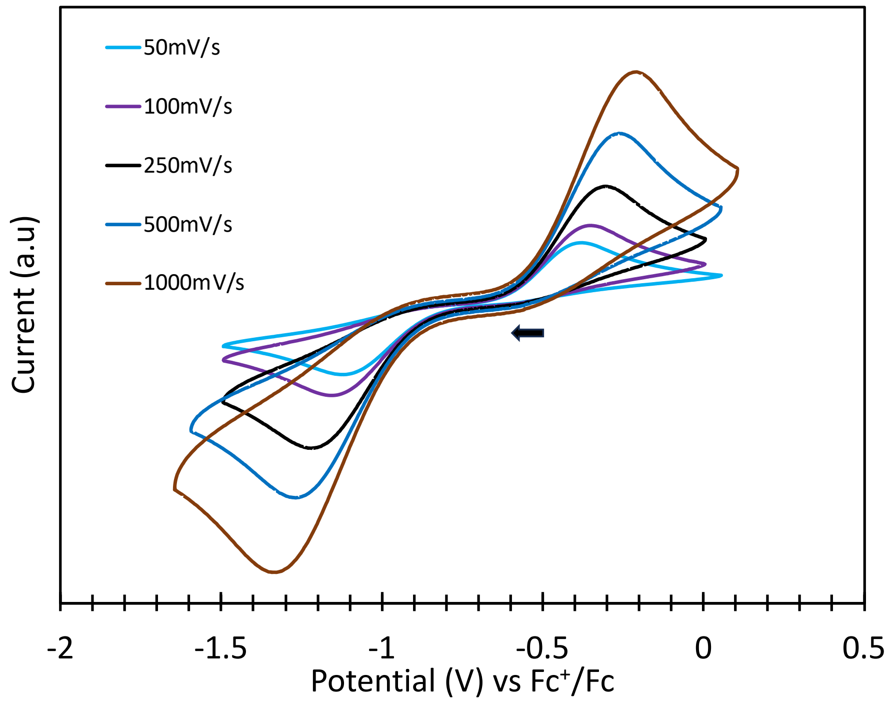


**Figure S75**. Cyclic voltammogram of **3-Ce**$^{\mathbf{OtBu}}$, in THF at room temperature with varying scan rates.

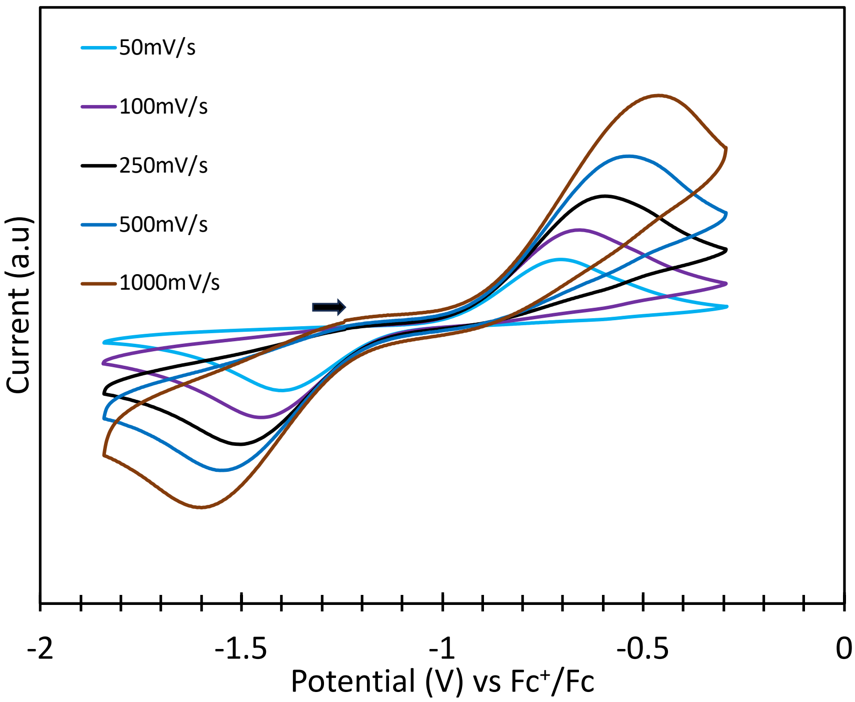


**Figure S76**. Cyclic voltammogram of **2-Ce$^{Ph}$**, in THF at room temperature with varying scan rates.

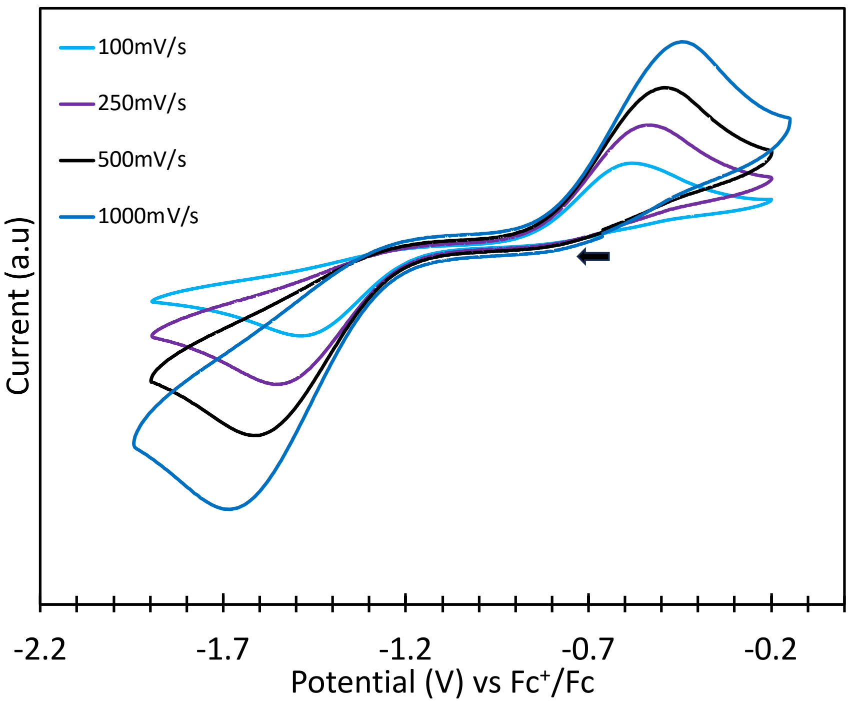


**Figure S77**. Cyclic voltammogram of **3-Ce$^{Ph}$**, in THF at room temperature with varying scan rates.

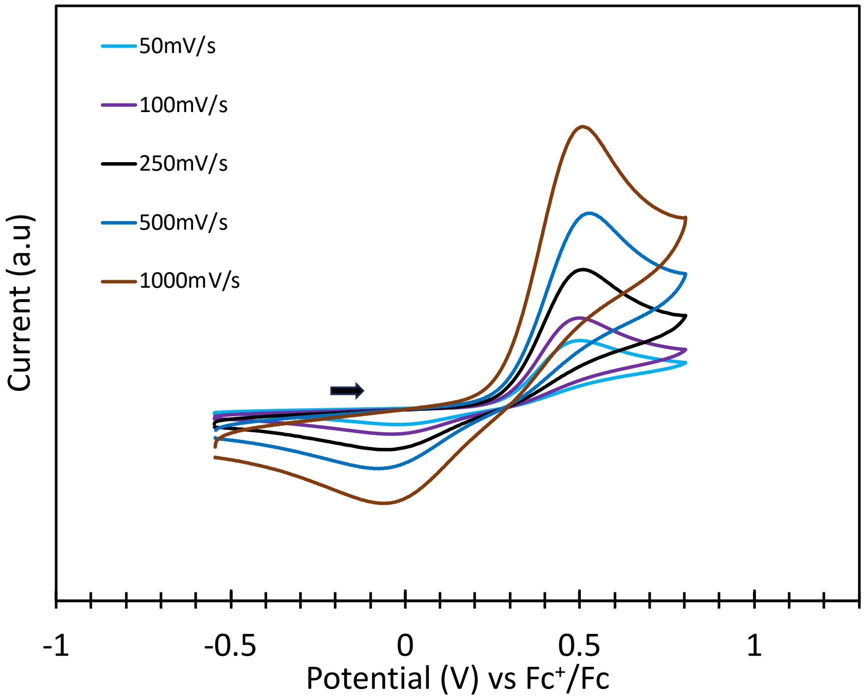


**Figure S78**. Cyclic voltammogram of **2-Tb$^{Ph}$**, in THF at room temperature with varying scan rates.

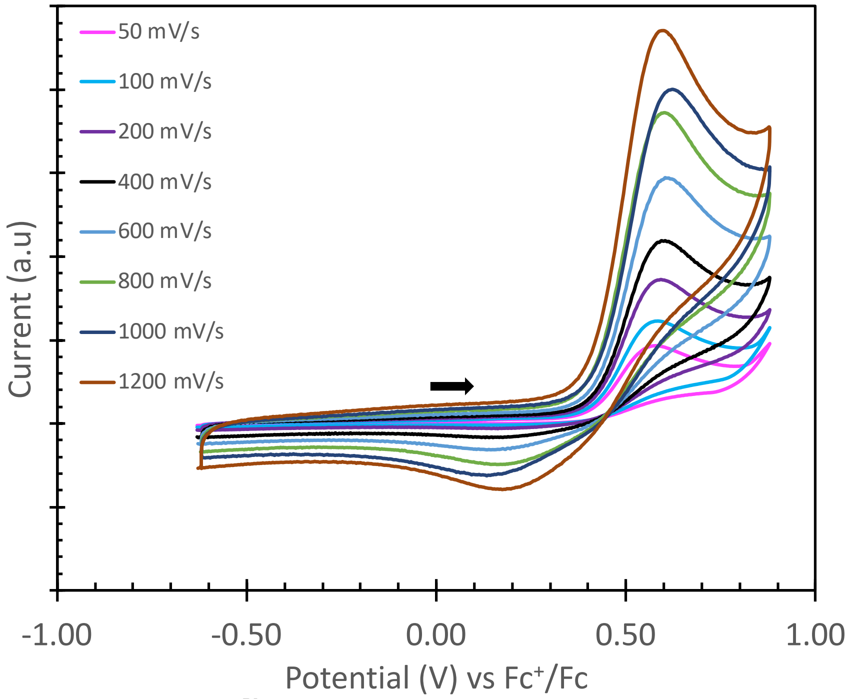


**Figure S79**. Cyclic voltammogram of **2-Pr$^{Ph}$**, in THF at room temperature with varying scan rates.

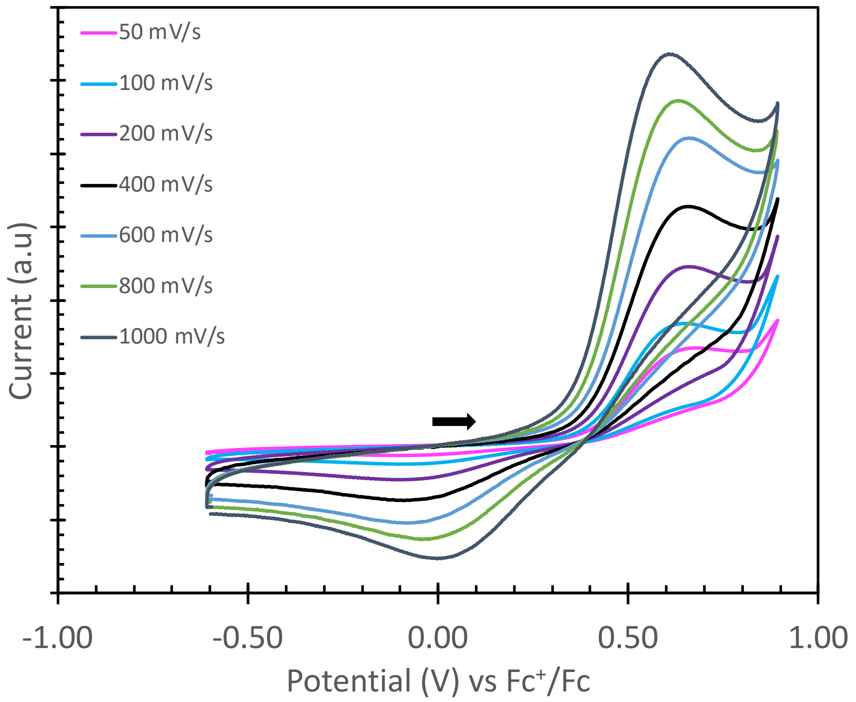


**Figure S80**. Cyclic voltammogram of **1-Tb$^{Ph}$** + 1.1 equiv. of KOSi(O$^t$Bu)$_3$, in THF at room temperature with varying scan rates.

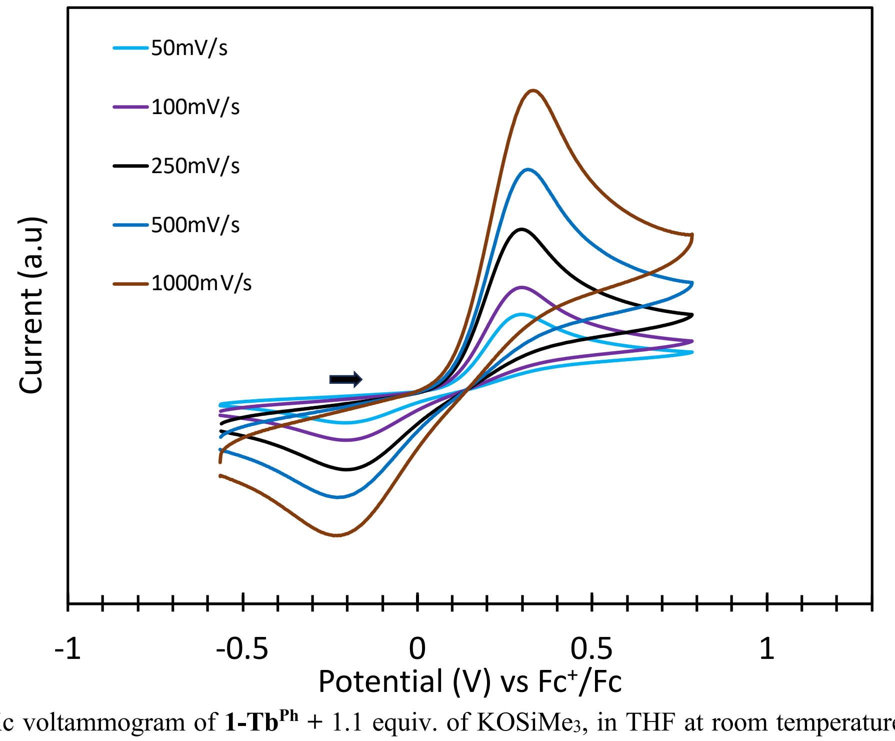


**Figure S81**. Cyclic voltammogram of **1-Tb$^{Ph}$** + 1.1 equiv. of KOSiMe$_3$, in THF at room temperature with varying scan rates.

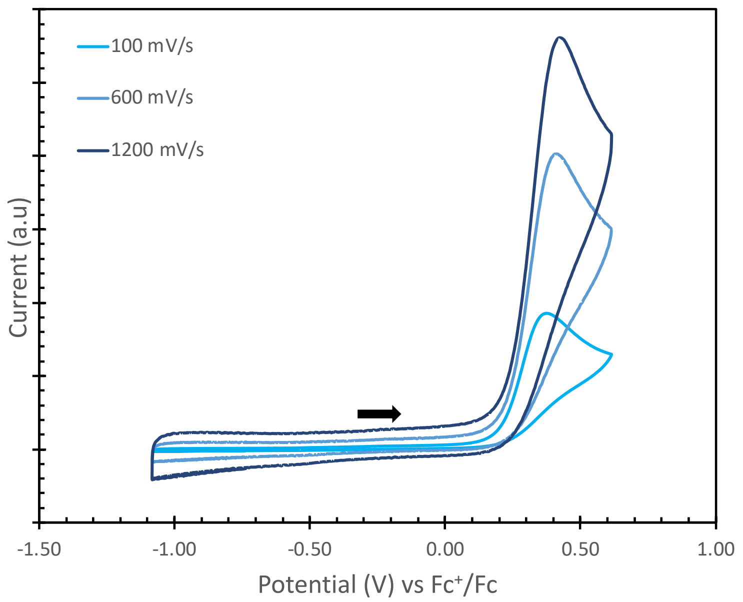


**Figure S82**. Cyclic voltammogram of **1-Tb**$^{\mathbf{Ph}}$ + 1.1 equiv. of $K(N(SiMe_3)_2$, in THF at room temperature with varying scan rates.

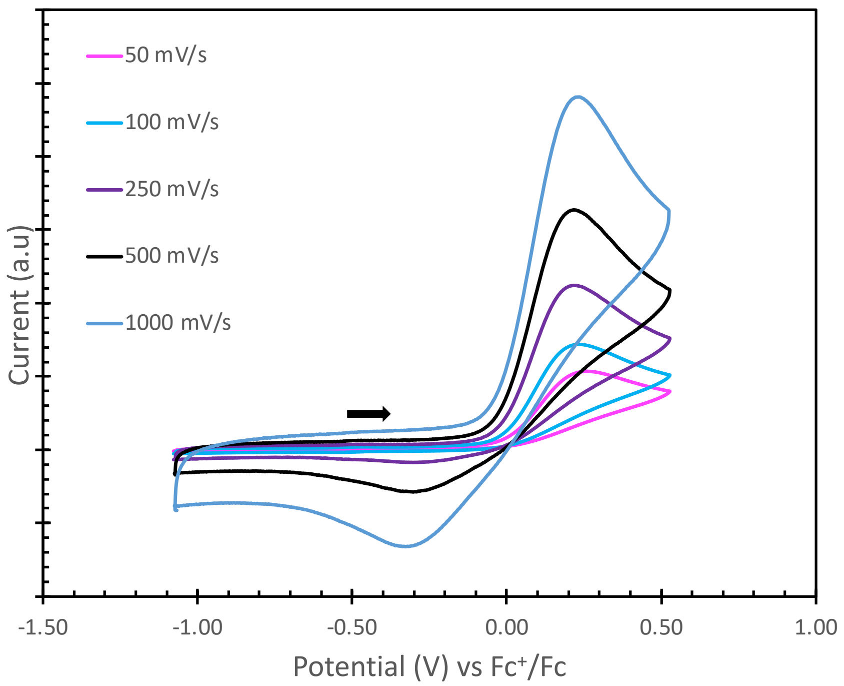


**Figure S83**. Cyclic voltammogram of **1-Tb**$^{\mathbf{Ph}}$ + 1.1 equiv. of 2-KOAd, in THF at room temperature with varying scan rates.

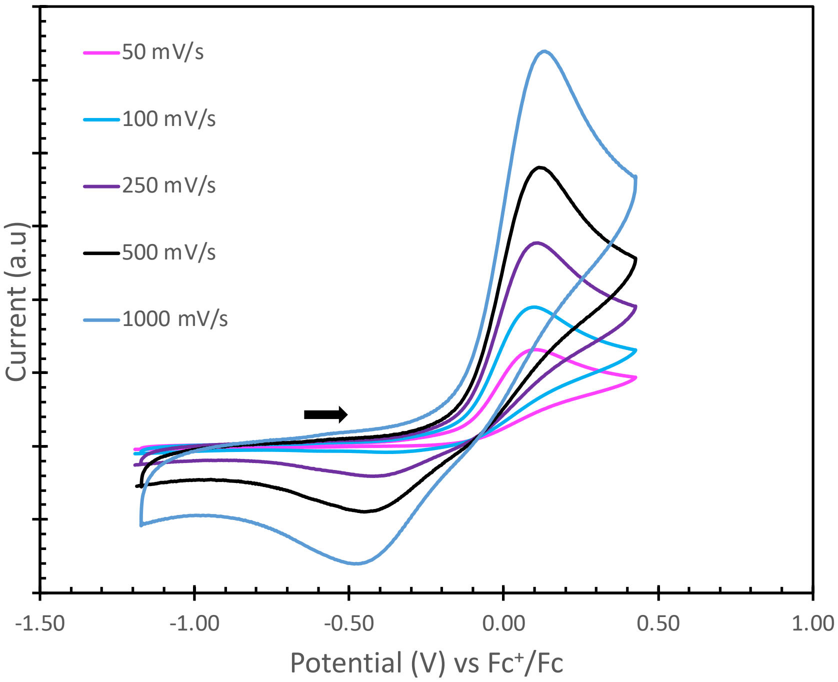


**Figure S84**. Cyclic voltammogram of **1-Tb$^{Ph}$** + 1.1 equiv. of NaO$^t$Bu, in THF at room temperature with varying scan rates.

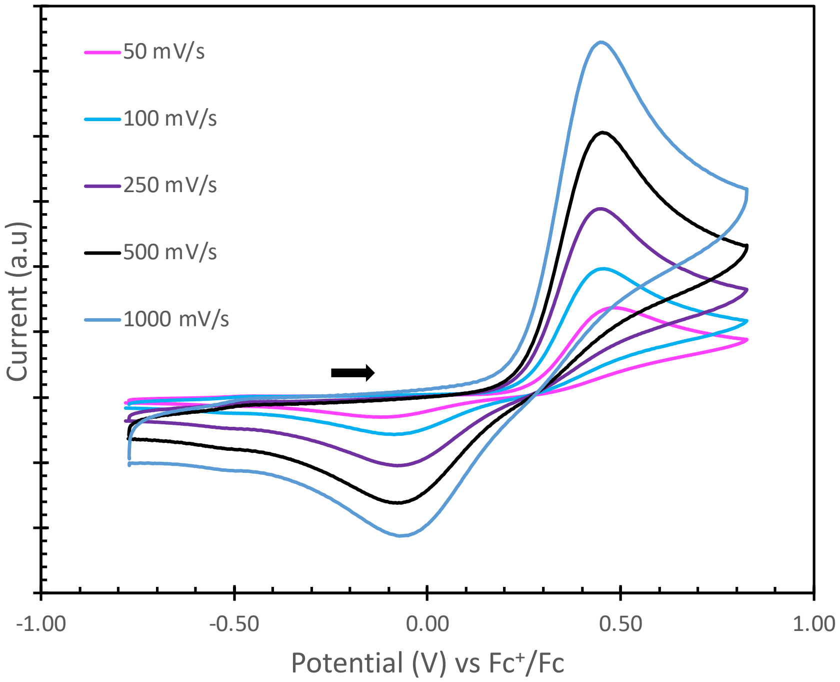


**Figure S85**. Cyclic voltammogram of **1-Pr$^{Ph}$** + 1.1 equiv. of $KOSiMe_3$, in THF at room temperature with varying scan rates.

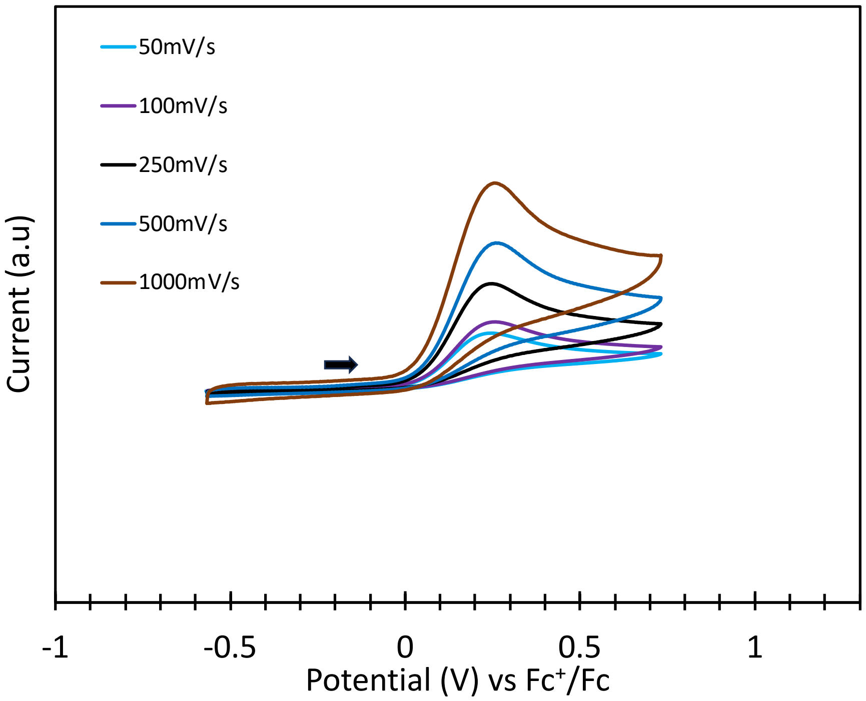


**Figure S86**. Cyclic voltammogram of **1-Pr$^{Ph}$** + 1.1 equiv. of 2-KOAd, in THF at room temperature with varying scan rates.

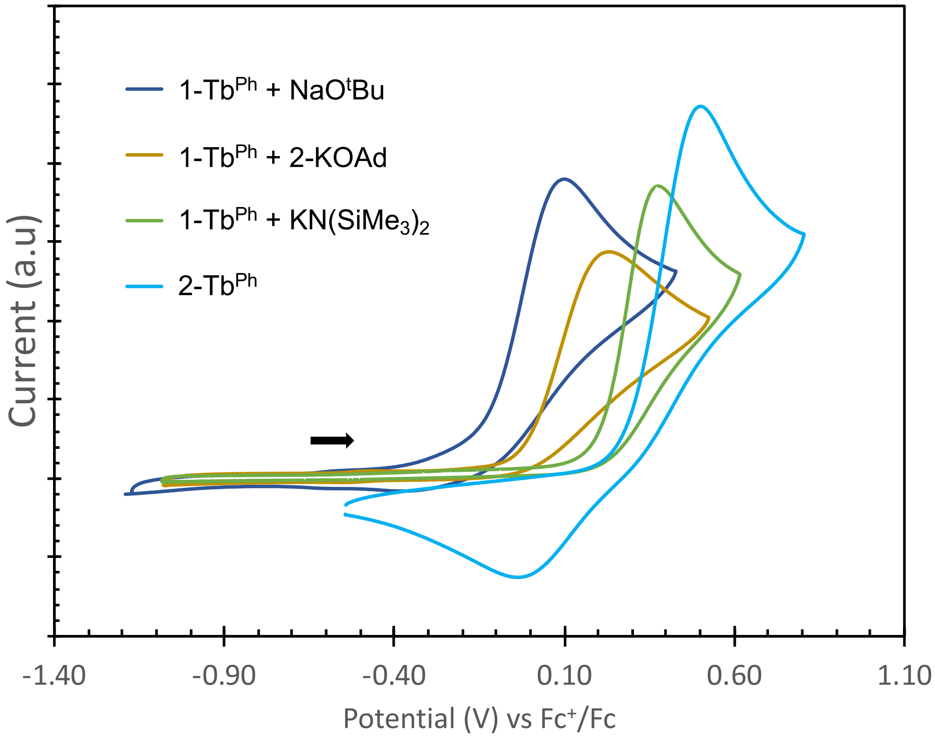


**Figure S87**. Cyclic voltammogram of **2-Tb$^{Ph}$** compared with **1-Tb$^{Ph}$** + 1.1 equiv. of K(N(SiMe$_3$)$_2$, **1-Tb$^{Ph}$** + 1.1 equiv. of 2-KOAd and **1-Tb$^{Ph}$** + 1.1 equiv. of NaO$^t$Bu, in THF at room temperature with a 100 mV/s scan rate.

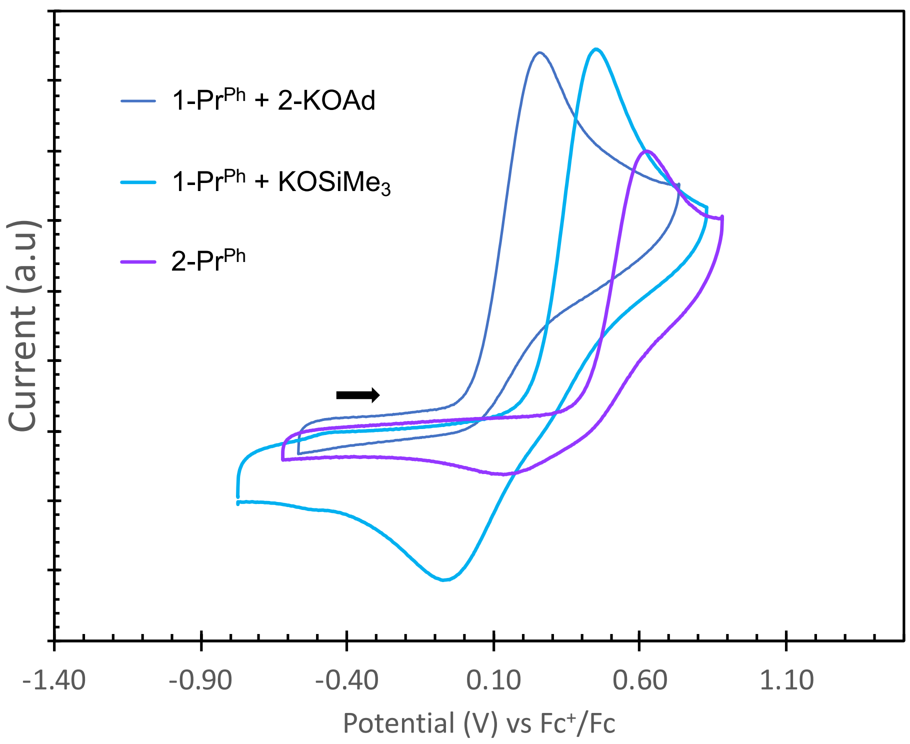


**Figure S88**. Cyclic voltammogram of **2-Pr$^{Ph}$** compared with **1-Pr$^{Ph}$** + 1.1 equiv. of $KOSiMe_3$ and **1-Pr$^{Ph}$** + 1.1 equiv. of 2-KOAd, in THF at room temperature with a 1000 mV/s scan rate.

## S9. Infrared (IR) spectra

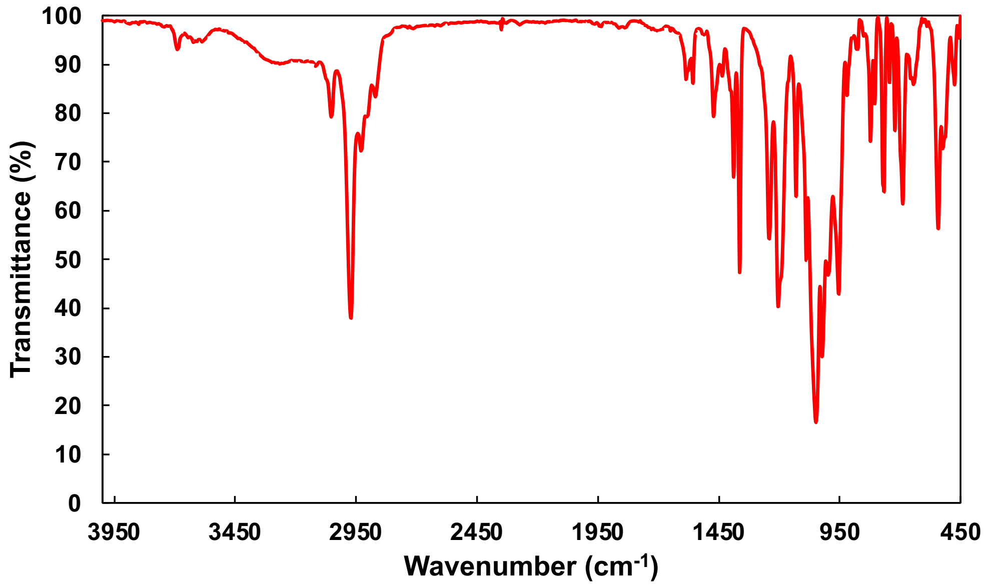


**Figure S89**. Infrared (IR) spectra for **1-Tb$^{OtBu}$** prepared as a KBr pellet.

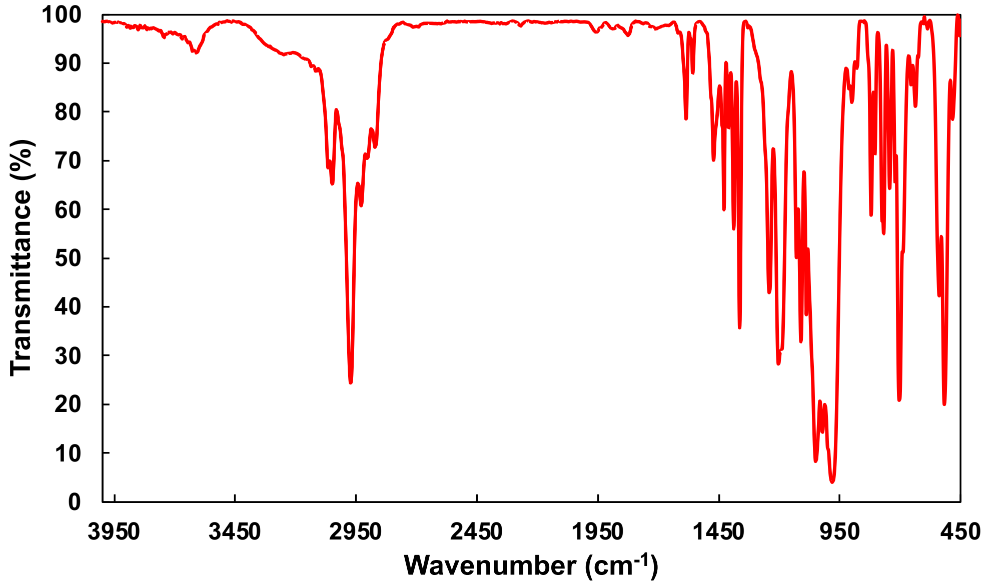


**Figure S90**. Infrared (IR) spectra for **2-Tb$^{OtBu}$** prepared as a KBr pellet.

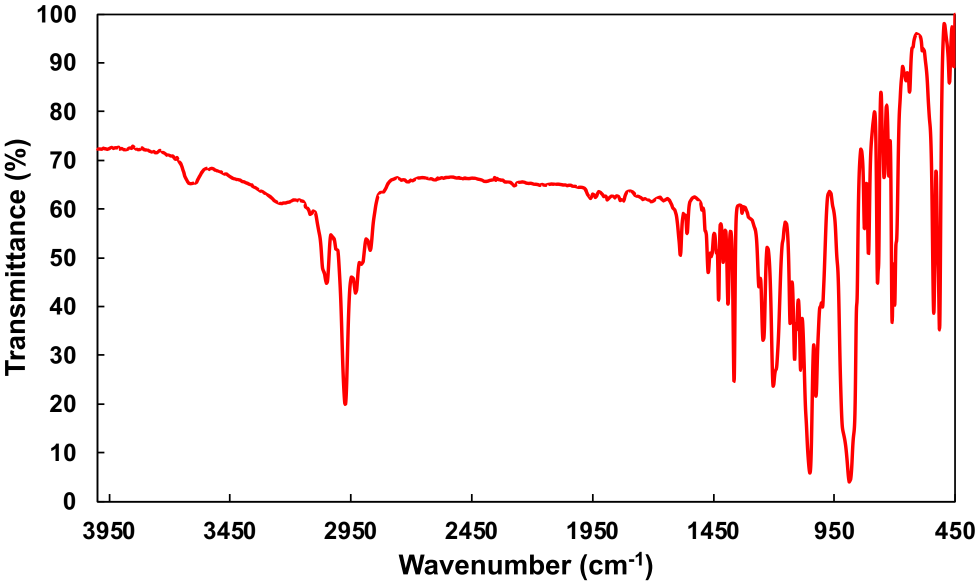


**Figure S91**. Infrared (IR) spectra for **2-Ce**$^{\mathbf{OtBu}}$ prepared as a KBr pellet.

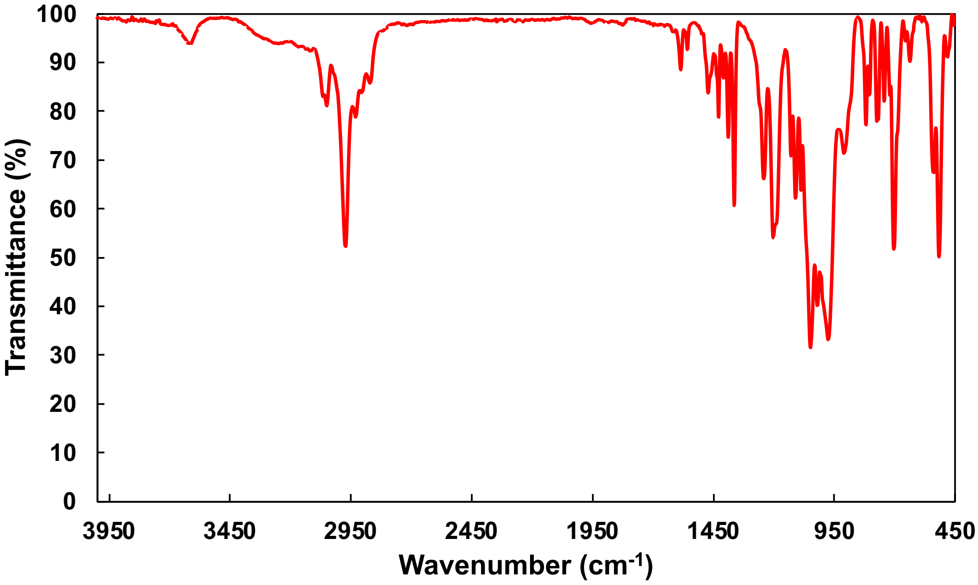


**Figure S92**. Infrared (IR) spectra for **3-Ce**$^{\mathbf{OtBu}}$ prepared as a KBr pellet.

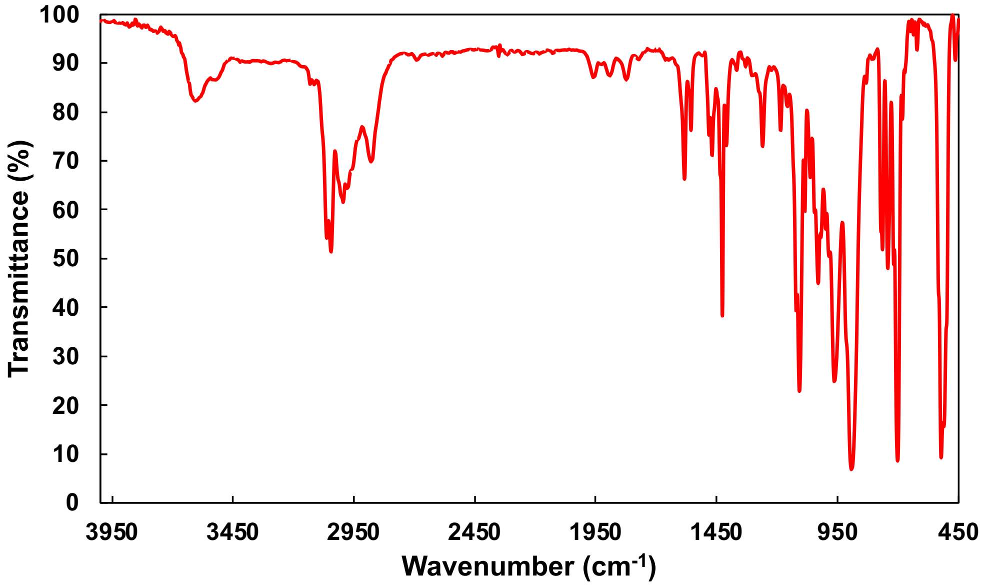


**Figure S93**. Infrared (IR) spectra for **1-Ce**$^{Ph}$ prepared as a KBr pellet.

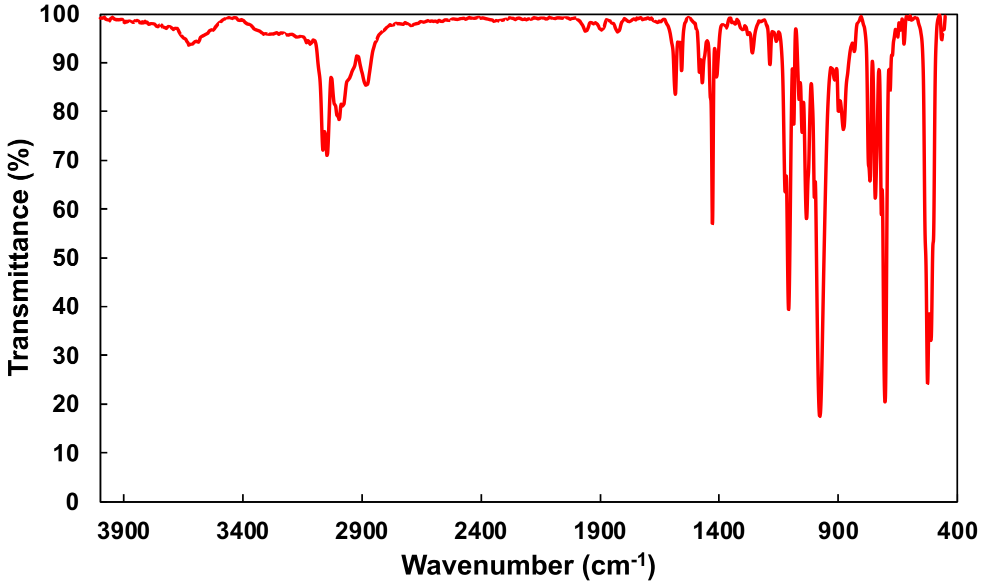


**Figure S94**. Infrared (IR) spectra for **1-Tb**$^{Ph}$ prepared as a KBr pellet.

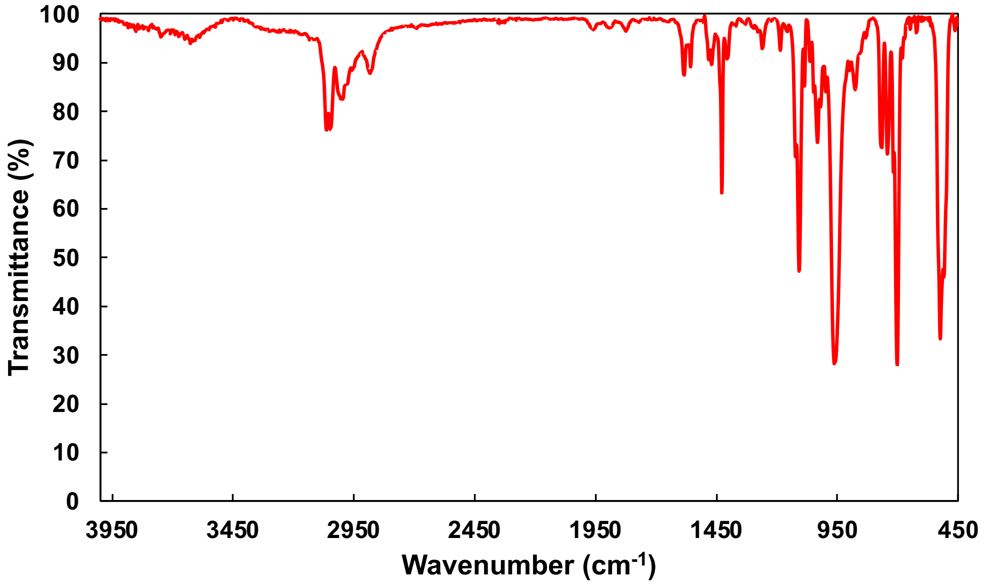


**Figure S95**. Infrared (IR) spectra for **1-Pr$^{Ph}$** prepared as a KBr pellet.

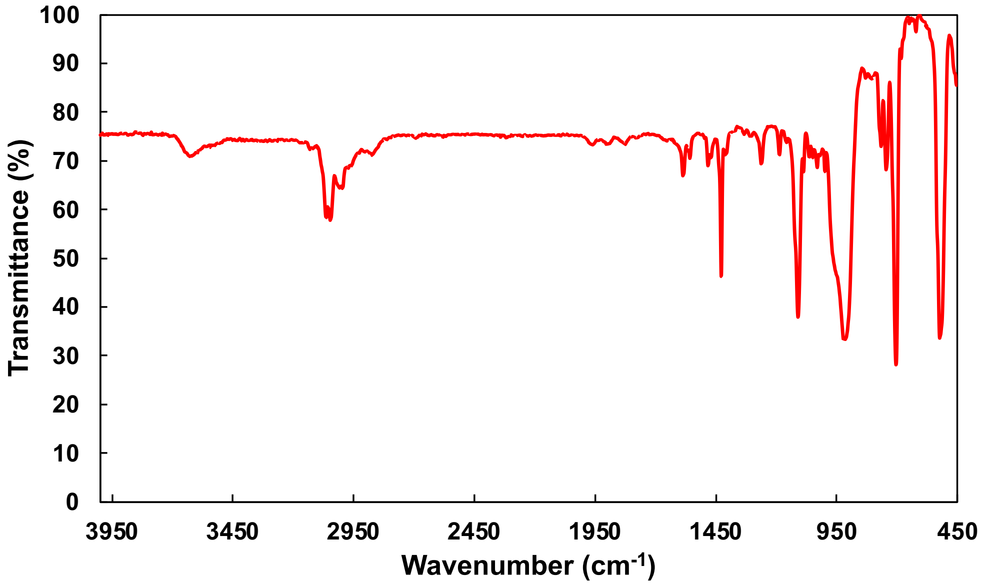


**Figure S96**. Infrared (IR) spectra for **2-Ce$^{Ph}$** prepared as a KBr pellet.

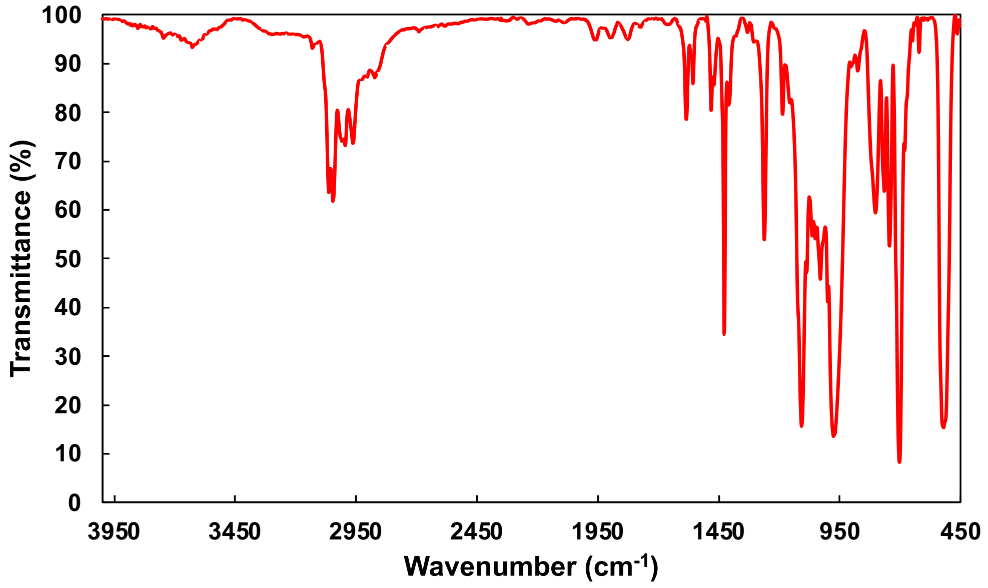


**Figure S97**. Infrared (IR) spectra for **2-Tb$^{Ph}$** prepared as a KBr pellet.

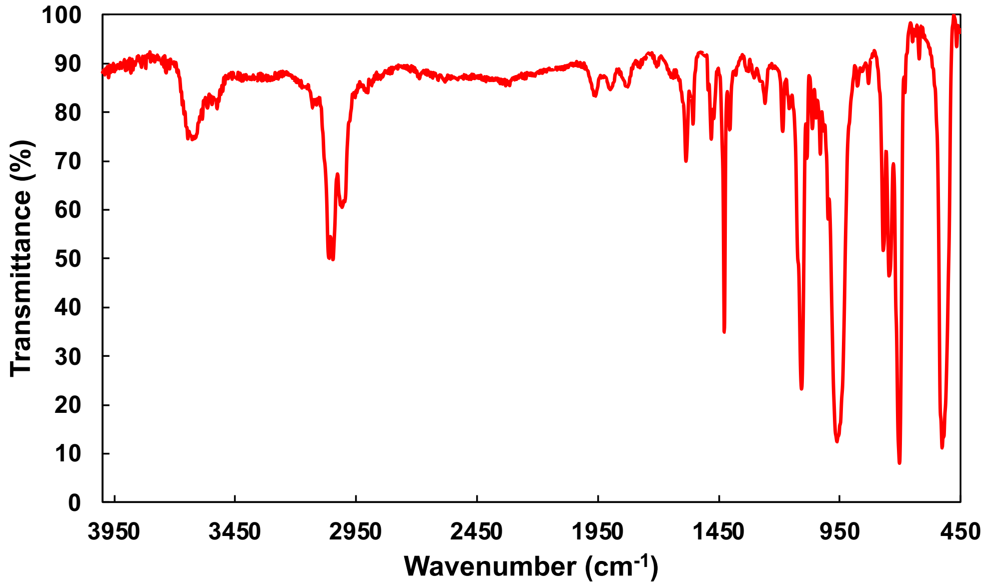


**Figure S98**. Infrared (IR) spectra for **2-Pr$^{Ph}$** prepared as a KBr pellet.

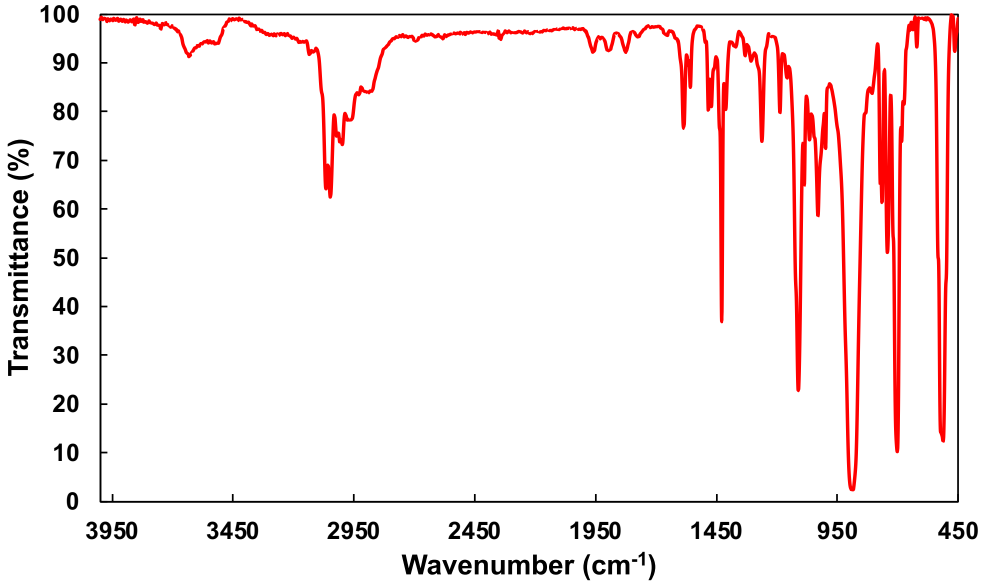


**Figure S99**. Infrared (IR) spectra for **3-Ce$^{Ph}$** prepared as a KBr pellet.

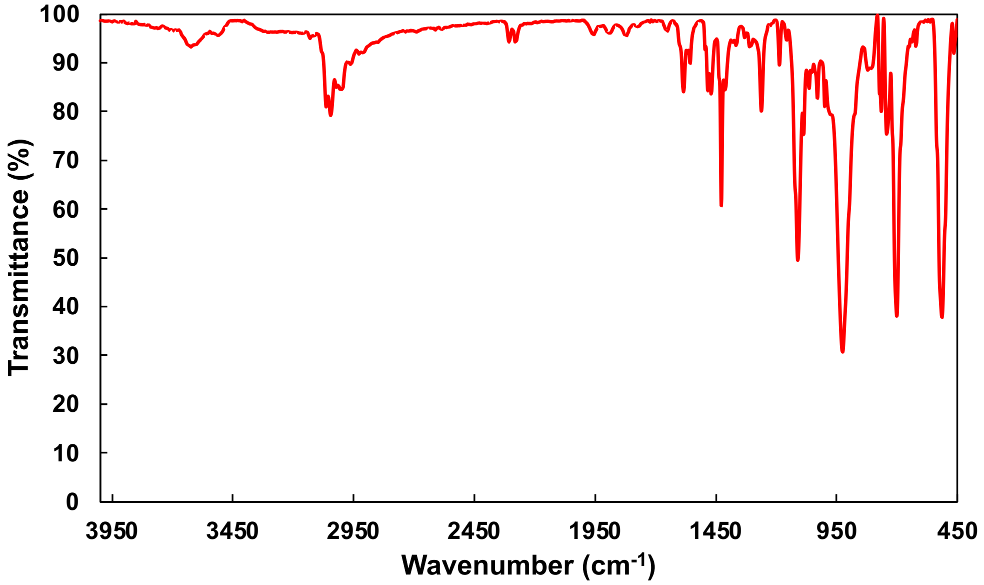


**Figure S100**. Infrared (IR) spectra for **3-Tb$^{Ph}$** prepared as a KBr pellet.

## S10. Determination of Enrichment and Separation factors

The Tb:Dy separations factors were determined by the following equation:

$$S = D_{residualsolid} \cdot D_{extractedsolid} = \frac{\eta_{Tb}}{\eta_{Dy}} \cdot \frac{\eta_{Dy}}{\eta_{Tb}}$$

The molar ratios ($\eta_{Ln}$) were determined both by integration of the Tb and Dy peaks in the thf-$d_8$ $^1$H NMR spectra after 24h or by ICP-MS analyses.

**Table S5.** Enrichment factors for the fraction obtained after extraction in toluene ($\eta_{Tb}/\eta_{Dy}$) and for the solid fraction ($\eta_{Dy}/\eta_{Tb}$).

| | $^1$H NMR | ICP-MS |
|---|---|---|
| $D_{extractedsolid}$ | 4.44 | 3.72 |
| $D_{residualsolid}$ | 2.27 | 2.30 |
| S | 10.1 | 8.56 |

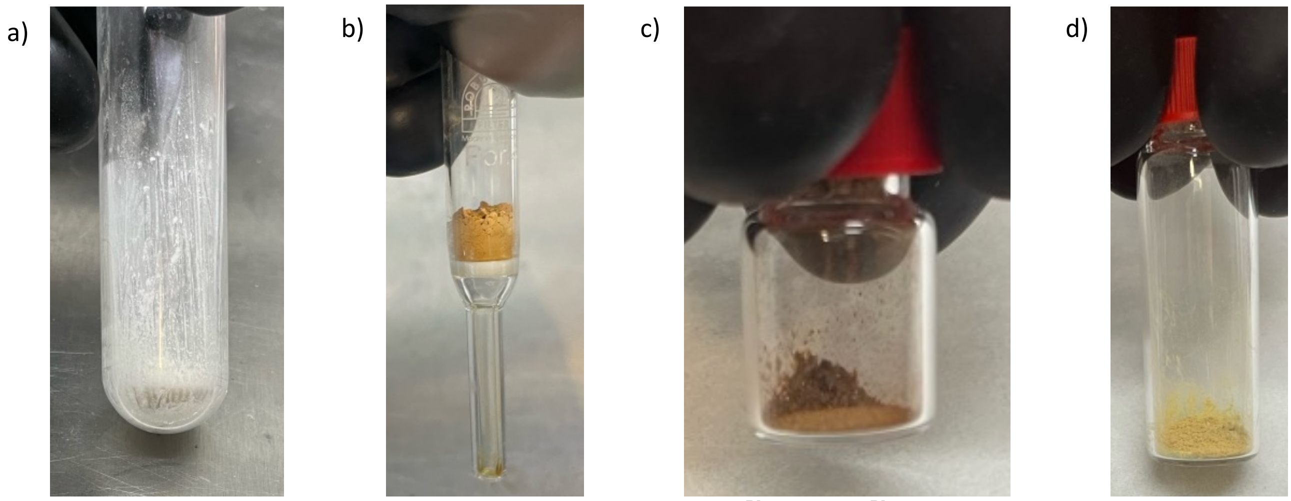


**Figure S101**. Photos of the separation process. (a) 1:1 mixture of **2-Tb$^{Ph}$** and **2-Dy$^{Ph}$** before oxidation, (b) solid obtained after the oxidation, (c) solid obtained after drying the fraction extracted in toluene. (d) residual insoluble in toluene solid after extraction.

## S11. Computational details

The optimization of cerium and terbium complexes were carried out by employing DFT hybrid functional (B3PW91)[14] along with small core pseudopotential Stuttgart basis set[15] for terbium (oxidation state, +3), cerium, potassium and silicon atoms (polarization functions[16] were added for silicon and potassium atoms). Pople basis set[17] (6-31G**) were employed for the rest of the atoms. Large core pseudopotential Stuttgart basis set for terbium was employed to converge the IV oxidation state in complexes **3** and **3$^{OtBu}$**.[18] Frequency calculations were performed to confirm the minima for optimized structures. Disperison corrections were included in our calculations by employing D3 version of Grimme's dispersion with Becke-Johnson damping.[19] All the calculations were performed using Gaussian 09 suite of programs.[20] NBO analysis were carried out using NBO 6.0 version as implemented in the Gaussian program.[21]

**Table S6.** Selected structural parameters of Ce(III) (**1-Ce$^{Ph}$**) for s=1/2

| Atom Label | Bond distance (Å) | |
|---|---|---|
| | DFT | X-ray |
| Ce1-O5 | 2.20 | 2.21 |
| Ce1-O6 | 2.19 | 2.20 |
| Ce1-O7 | 2.19 | 2.21 |
| Ce1-O8 | 2.62 | 2.61 |
| Ce1-O9 | 2.63 | 2.58 |
| Ce1-O10 | 2.62 | 2.60 |
| Ce1-X (center of phenyl ring) | 4.02 | 3.93 |

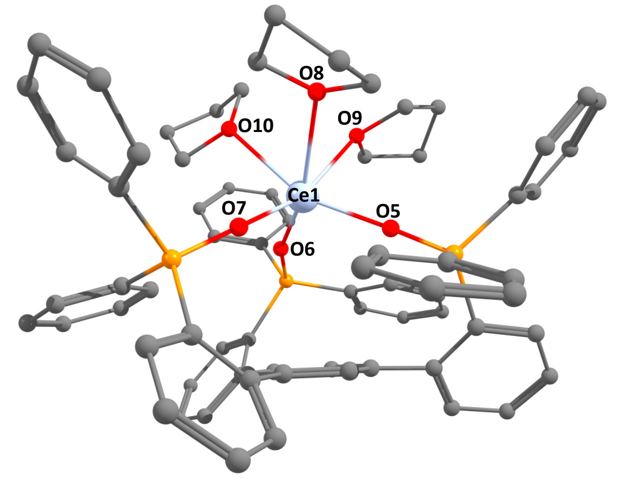


**Table S7.** Computed Natural charges for complex **1-Ce$^{Ph}$** for s=1/2

| Atom Label | Natural charges |
|---|---|
| Ce1 | 1.93891 |
| O5 | -1.27410 |
| O6 | -1.27304 |
| O7 | -1.26882 |
| O8 | -0.63424 |
| O9 | -0.63470 |
| O10 | -0.63002 |

**Table S8.** Computed Wiberg bond index for complex **1-Ce$^{Ph}$** for s=1/2

| Atom Label | Wiberg bond index | Atom Label | Wiberg bond index | Atom Label | Wiberg bond index |
|---|---|---|---|---|---|
| Ce1 | 0.0000 | Ce1 | 0.0000 | Ce1 | 0.0000 |
| O5 | 0.4582 | O6 | 0.4645 | O7 | 0.4761 |
| Atom Label | Wiberg bond index | Atom Label | Wiberg bond index | Atom Label | Wiberg bond index |
| Ce1 | 0.0000 | Ce1 | 0.0000 | Ce1 | 0.0000 |
| O8 | 0.1341 | O9 | 0.1285 | O10 | 0.1362 |

**Table S9.** Bonding orbitals from NBO analysis for complex **1-Ce$^{Ph}$**

(0.99097) BD ( 1)Ce 1- O 7
( 5.08%) 0.2254*Ce 1 s( 0.89%)p 0.80( 0.71%)d73.53( 65.27%) f36.94( 32.79%)g 0.39( 0.34%)
( 94.92%) 0.9743* O 7 s( 55.07%)p 0.82( 44.92%)d 0.00( 0.00%)

**Table S10.** Second order perturbation analysis for complex **1-Ce$^{Ph}$**

| Donor NBO | Acceptor NBO | E(2) kcal/mol |
|---|---|---|
| (0.94276) LP ( 1) O 5 | (0.07843) LV ( 2)Ce 1 | 38.15 |

| | | |
|---|---|---|
| s( 54.91%)p 0.82( 45.08%)d 0.00( 0.01%) | s( 0.84%)p 0.76( 0.63%)d79.59( 66.81%)f37.39( 31.38%)g 0.40( 0.34%) | |
| (0.91935) LP ( 2) O 5<br>s( 0.00%)p 1.00( 99.98%)d 0.00( 0.02%) | (0.08101) LV ( 1)Ce 1<br>s( 0.02%)p 1.50( 0.03%)d99.99( 98.39%)f76.08( 1.37%)g10.58( 0.19%) | 5.49 |
| (0.94260) LP ( 1) O 6<br>s( 55.00%)p 0.82( 44.99%)d 0.00( 0.01%) | (0.07767) LV ( 4)Ce 1<br>s( 0.93%)p 0.77( 0.72%)d70.12( 65.25%)f35.18( 32.74%)g 0.38( 0.36%) | 38.21 |
| (0.91874) LP ( 2) O 6<br>s( 0.01%)p 1.00( 99.97%)d 0.00( 0.02%) | (0.08101) LV ( 1)Ce 1<br>s( 0.02%)p 1.50( 0.03%)d99.99( 98.39%)f76.08( 1.37%)g10.58( 0.19%) | 4.41 |
| (0.91830) LP ( 1) O 7<br>s( 0.01%)p 1.00( 99.98%)d 0.00( 0.02%) | (0.07803) LV ( 3)Ce 1<br>s( 0.00%)p 1.00( 0.02%)d99.99( 97.51%)f94.21( 2.29%)g 6.88( 0.17%) | 7.18 |
| (0.95834) LP ( 2) O 8<br>s( 20.67%)p 3.84( 79.30%)d 0.00( 0.03%) | (0.07767) LV ( 4)Ce 1<br>s( 0.93%)p 0.77( 0.72%)d70.12( 65.25%)f35.18( 32.74%)g 0.38( 0.36%) | 3.25 |
| (0.95834) LP ( 2) O 8<br>s( 20.67%)p 3.84( 79.30%)d 0.00( 0.03%) | (0.02444) LV ( 7)Ce 1<br>s( 0.40%)p 1.55( 0.61%)d36.10( 14.30%)f99.99( 84.35%)g 0.86( 0.34%) | 5.86 |
| (0.95989) LP ( 2) O 9<br>s( 21.30%)p 3.69( 78.68%)d 0.00( 0.02%) | (0.02293) LV ( 8)Ce 1<br>s( 0.16%)p 4.11( 0.67%)d78.65( 12.83%)f99.99( 85.89%)g 2.73( 0.45%) | 3.08 |
| (0.95815) LP ( 2) O 10<br>s( 20.62%)p 3.85( 79.36%)d 0.00( 0.03%) | (0.02293) LV ( 8)Ce 1<br>s( 0.16%)p 4.11( 0.67%)d78.65( 12.83%)f99.99( 85.89%)g 2.73( 0.45%) | 4.04 |

**Table S11.** DFT computed MO's for **1-Ce$^{Ph}$**. (a)AMO-HOMO-1 (b)AMO-HOMO (c) spin density plot (AMO: Alpha Molecular Orbital)

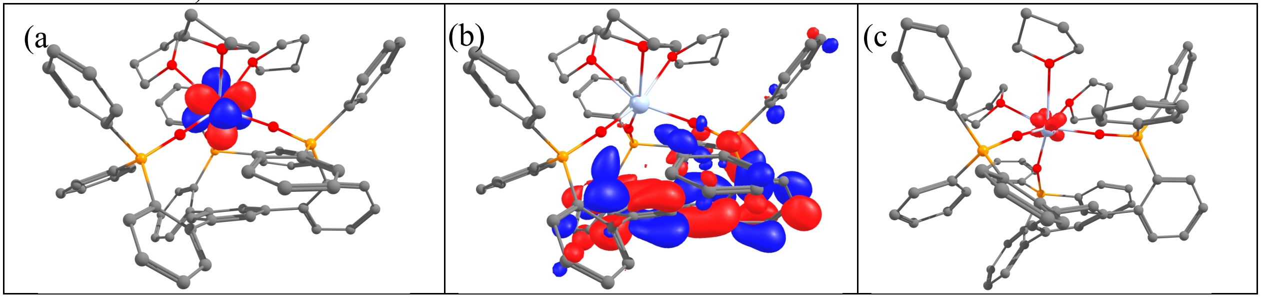


**Table S12.** Selected structural parameters of Tb(III) (**2-Tb$^{Ph}$**) for s=3

| Atom Label | Bond distance (Å) | |
|---|---|---|
| | DFT | X-ray |
| Tb1-O7 | 2.15 | 2.19 |
| Tb1-O8 | 2.09 | 2.14 |
| Tb1-O9 | 2.10 | 2.13 |
| Tb1-O10 | 2.16 | 2.19 |
| Tb1-X (center of phenyl ring) | 3.51 | 3.23 |

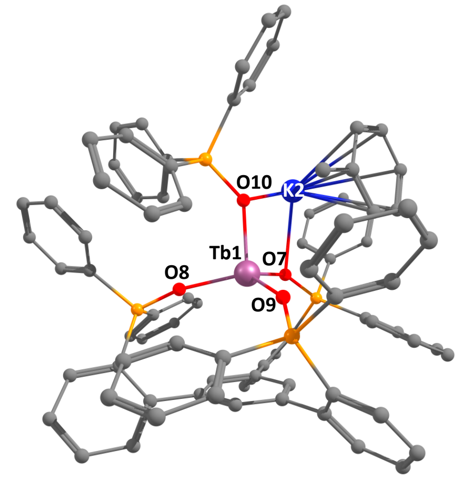

**Table S13.** Computed Natural charges for complex **2-Tb$^{Ph}$** for s=3

| Atom Label | Natural charges |
|---|---|
| Tb1 | 2.01870 |
| K2 | 0.95973 |
| O7 | -1.32287 |
| O8 | -1.30182 |
| O9 | -1.29923 |
| O10 | -1.32332 |

**Table S14.** Computed Wiberg bond index for complex **2-Tb$^{Ph}$** for s=3

| Atom Label | Wiberg bond index | Atom Label | Wiberg bond index | Atom Label | Wiberg bond index |
|---|---|---|---|---|---|
| Tb1 | 0.0000 | Tb1 | 0.0000 | Tb1 | 0.0000 |
| O7 | 0.3874 | O8 | 0.4361 | O9 | 0.4216 |
| Atom Label | Wiberg bond index | | | | |
| Tb1 | 0.0000 | | | | |
| O10 | 0.3985 | | | | |

**Table S15.** Second order perturbation analysis for complex **2-Tb$^{Ph}$**

| Donor NBO | Acceptor NBO | E(2) kcal/mol |
|---|---|---|
| (0.95430) LP ( 1) O 7<br>s( 40.28%)p 1.48( 59.70%)d 0.00( 0.02%) | (0.09748) LV ( 2)Tb 1<br>s( 0.26%)p 1.14( 0.30%)d99.99( 99.36%)f 0.17( 0.04%)g 0.15( 0.04%) | 14.33 |
| (0.95430) LP ( 1) O 7<br>s( 40.28%)p 1.48( 59.70%)d 0.00( 0.02%) | (0.08283) LV ( 5)Tb 1<br>s( 1.67%)p 0.15( 0.24%)d58.71( 97.89%)f 0.11( 0.18%)g 0.01( 0.02%) | 7.16 |
| (0.93008) LP ( 2) O 7<br>s( 0.68%)p99.99( 99.30%)d 0.03( 0.02%) | (0.08466) LV ( 4)Tb 1<br>s( 2.27%)p 0.08( 0.18%)d42.84( 97.39%)f 0.06( 0.13%)g 0.01( 0.02%) | 4.97 |
| (0.95113) LP ( 1) O 8<br>s( 37.26%)p 1.68( 62.72%)d 0.00( 0.01%) | (0.09909) LV ( 1)Tb 1<br>s( 0.00%)p 1.00( 0.53%)d99.99( 99.32%)f 0.22( 0.11%)g 0.07( 0.04%) | 8.89 |
| (0.95113) LP ( 1) O 8<br>s( 37.26%)p 1.68( 62.72%)d 0.00( 0.01%) | (0.09748) LV ( 2)Tb 1<br>s( 0.26%)p 1.14( 0.30%)d99.99( 99.36%)f 0.17( 0.04%)g 0.15( 0.04%) | 11.14 |
| (0.95113) LP ( 1) O 8<br>s( 37.26%)p 1.68( 62.72%)d 0.00( 0.01%) | (0.08283) LV ( 5)Tb 1<br>s( 1.67%)p 0.15( 0.24%)d58.71( 97.89%)f 0.11( 0.18%)g 0.01( 0.02%) | 6.01 |
| (0.92489) LP ( 2) O 8<br>s( 0.97%)p99.99( 99.01%)d 0.02( 0.02%) | (0.08466) LV ( 4)Tb 1<br>s( 2.27%)p 0.08( 0.18%)d42.84( 97.39%)f 0.06( 0.13%)g 0.01( 0.02%) | 4.23 |
| (0.95219) LP ( 1) O 9<br>s( 39.23%)p 1.55( 60.75%)d 0.00( 0.02%) | (0.09909) LV ( 1)Tb 1<br>s( 0.00%)p 1.00( 0.53%)d99.99( 99.32%)f 0.22( 0.11%)g 0.07( 0.04%) | 20.19 |
| (0.92530) LP ( 2) O 9<br>s( 0.82%)p99.99( 99.16%)d 0.03( 0.02%) | (0.09748) LV ( 2)Tb 1<br>s( 0.26%)p 1.14( 0.30%)d99.99( 99.36%)f 0.17( 0.04%)g 0.15( 0.04%) | 4.95 |
| (0.91908) LP ( 3) O 9<br>s( 0.05%)p99.99( 99.93%)d 0.45( 0.02%) | (0.09072) LV ( 3)Tb 1<br>s( 0.08%)p 0.97( 0.08%)d99.99( 99.76%)f 0.69( 0.06%)g 0.29( 0.02%) | 7.56 |
| (0.95553) LP ( 1) O 10<br>s( 42.19%)p 1.37( 57.78%)d 0.00( 0.03%) | (0.09072) LV ( 3)Tb 1<br>s( 0.08%)p 0.97( 0.08%)d99.99( 99.76%)f 0.69( 0.06%)g 0.29( 0.02%) | 4.22 |
| (0.95553) LP ( 1) O 10<br>s( 42.19%)p 1.37( 57.78%)d 0.00( 0.03%) | (0.08466) LV ( 4)Tb 1<br>s( 2.27%)p 0.08( 0.18%)d42.84( 97.39%)f 0.06( 0.13%)g 0.01( 0.02%) | 8.39 |
| (0.95553) LP ( 1) O 10 | (0.08283) LV ( 5)Tb 1 | 11.33 |

| | | |
|---|---|---|
| s( 42.19%)p 1.37( 57.78%)d 0.00( 0.03%) | s( 1.67%)p 0.15( 0.24%)d58.71( 97.89%)f 0.11( 0.18%)g 0.01( 0.02%) | |
| (0.93027) LP ( 2) O 10<br>s( 0.31%)p99.99( 99.66%)d 0.08( 0.03% | (0.08283) LV ( 5)Tb 1<br>s( 1.67%)p 0.15( 0.24%)d58.71( 97.89%)f 0.11( 0.18%)g 0.01( 0.02%) | 7.48 |
| (0.92727) LP ( 3) O 10<br>s( 0.02%)p99.99( 99.94%)d 1.43( 0.03%) | (0.09072) LV ( 3)Tb 1<br>s( 0.08%)p 0.97( 0.08%)d99.99( 99.76%)f 0.69( 0.06%)g 0.29( 0.02%) | 5.69 |

**Table S16.** DFT computed MO's for **2-Tb$^{Ph}$**. (a)AMO-HOMO-90 (b)AMO-HOMO-89 (c)AMO-HOMO-88 (d)AMO-HOMO-87 (e)AMO-HOMO-84 (f)AMO-HOMO-83 (g)AMO-HOMO-81 (h)AMO-HOMO (i)BMO-LUMO-1 (AMO:Alpha Molecular Orbital, BMO: Beta Molecular Orbital)

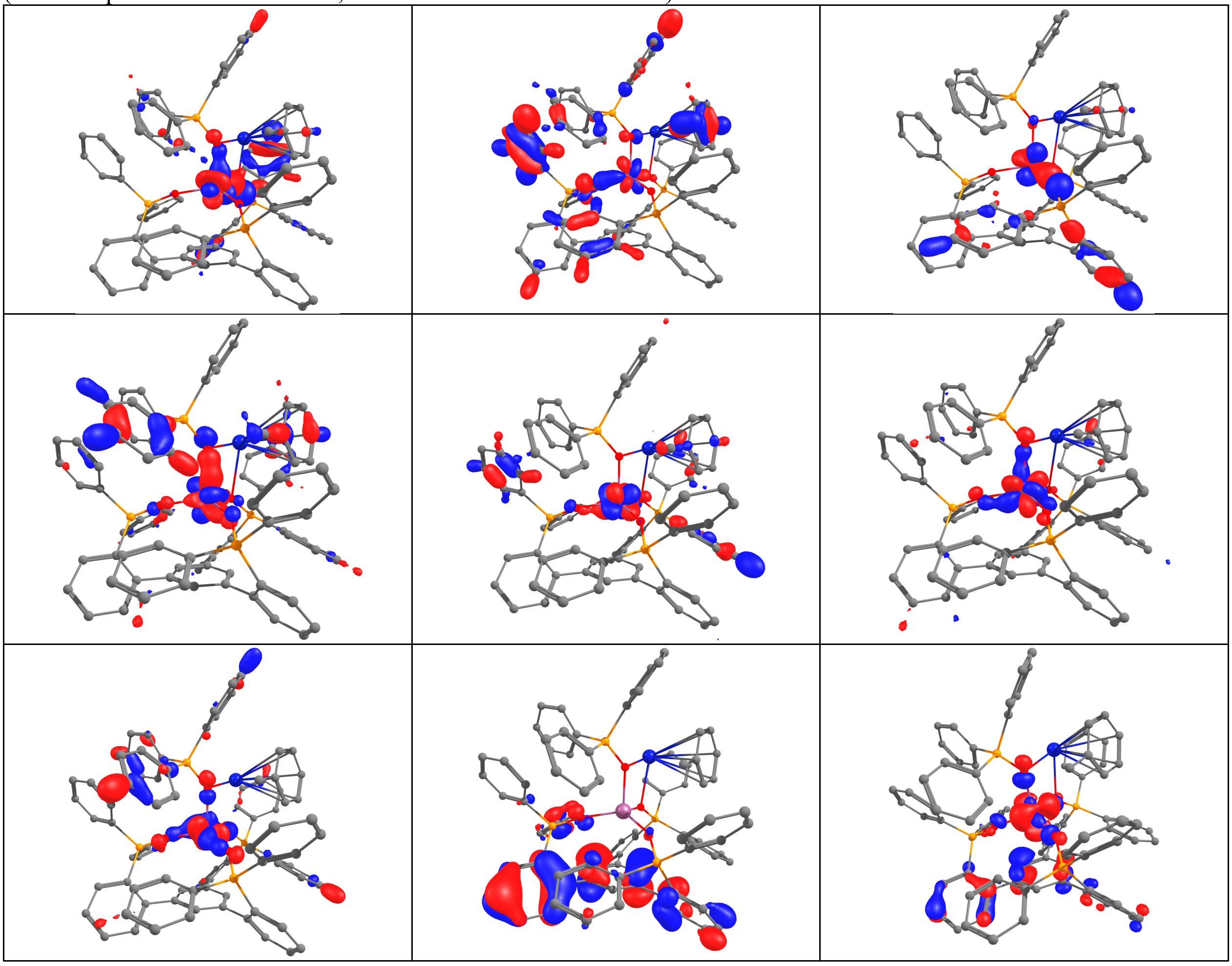

**Table S17.** Selected structural parameters of Tb(IV) (**3-Tb$^{Ph}$**)

| Atom Label | Bond distance (Å) | | |
|---|---|---|---|
| | DFT | X-ray | |
| Tb1-O6 | 2.01 | 2.03 | |
| Tb1-O7 | 2.07 | 2.08 | |
| Tb1-O8 | 2.03 | 2.04 | |
| Tb1-O9 | 2.05 | 2.07 | |
| Tb1-N10 | 2.52 | 2.51 | |
| Tb1-N11 | 2.52 | 2.47 | |

| | | | |
|---|---|---|---|
| Tb1-X (center of phenyl ring) | 4.02 | 4.04 | 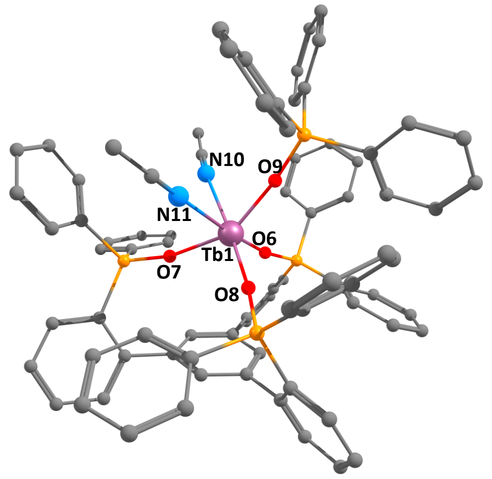 |

**Table S18.** Computed Natural charges for complex **3-Tb$^{Ph}$**

| Atom Label | Natural charges |
|---|---|
| Tb1 | 2.55352 |
| O6 | -1.28907 |
| O7 | -1.30485 |
| O8 | -1.29957 |
| O9 | -1.28604 |
| N10 | -0.43686 |
| N11 | -0.45080 |

**Table S19.** Computed Wiberg bond index for complex **3-Tb$^{Ph}$**

| Atom Label | Wiberg bond index | Atom Label | Wiberg bond index | Atom Label | Wiberg bond index |
|---|---|---|---|---|---|
| Tb1 | 0.0000 | Tb1 | 0.0000 | Tb1 | 0.0000 |
| O6 | 0.5164 | O7 | 0.4419 | O8 | 0.4937 |
| Atom Label | Wiberg bond index | Atom Label | Wiberg bond index | Atom Label | Wiberg bond index |
| Tb1 | 0.0000 | Tb1 | 0.0000 | Tb1 | 0.0000 |
| O9 | 0.5044 | N10 | 0.1864 | N11 | 0.1859 |

**Table S20.** Second order perturbation analysis for complex **3-Tb$^{Ph}$**

| Donor NBO | Acceptor NBO | E(2) kcal/mol |
|---|---|---|
| (1.89505) LP ( 1) O 6<br>s( 36.66%)p 1.73( 63.33%)d 0.00( 0.02%) | (0.28440) LV ( 1)Tb 1<br>s( 0.00%)p 1.00( 0.17%)d99.99( 99.78%)f 0.24( 0.04%) | 41.67 |
| (1.89505) LP ( 1) O 6<br>s( 36.66%)p 1.73( 63.33%)d 0.00( 0.02%) | (0.28093) LV ( 2)Tb 1<br>s( 0.02%)p 9.05( 0.17%)d99.99( 99.74%)f 3.76( 0.07%) | 11.22 |
| (1.89505) LP ( 1) O 6<br>s( 36.66%)p 1.73( 63.33%)d 0.00( 0.02%) | (0.26365) LV ( 3)Tb 1<br>s( 0.30%)p 0.74( 0.22%)d99.99( 99.45%)f 0.09( 0.03%) | 15.37 |
| (1.90710) LP ( 1) O 7<br>s( 34.56%)p 1.89( 65.43%)d 0.00( 0.01%) | (0.26365) LV ( 3)Tb 1<br>s( 0.30%)p 0.74( 0.22%)d99.99( 99.45%)f 0.09( 0.03%) | 49.20 |
| (1.84462) LP ( 2) O 7<br>s( 1.71%)p57.59( 98.28%)d 0.01( 0.01%) | (0.28093) LV ( 2)Tb 1<br>s( 0.02%)p 9.05( 0.17%)d99.99( 99.74%)f 3.76( 0.07%) | 8.32 |
| (1.84211) LP ( 3) O 7<br>s( 1.67%)p58.70( 98.31%)d 0.01( 0.02%) | (0.28440) LV ( 1)Tb 1<br>s( 0.00%)p 1.00( 0.17%)d99.99( 99.78%)f 0.24( 0.04%) | 7.31 |
| (1.89742) LP ( 1) O 8 | (0.28440) LV ( 1)Tb 1 | 31.10 |

| | | |
|---|---|---|
| s( 35.67%)p 1.80( 64.31%)d 0.00( 0.01%) | s( 0.00%)p 1.00( 0.17%)d99.99( 99.78%)f 0.24( 0.04%) | |
| (1.89742) LP ( 1) O 8<br>s( 35.67%)p 1.80( 64.31%)d 0.00( 0.01%) | (0.28093) LV ( 2)Tb 1<br>s( 0.02%)p 9.05( 0.17%)d99.99( 99.74%)f 3.76( 0.07%) | 20.44 |
| (1.89742) LP ( 1) O 8<br>s( 35.67%)p 1.80( 64.31%)d 0.00( 0.01%) | (0.26365) LV ( 3)Tb 1<br>s( 0.30%)p 0.74( 0.22%)d99.99( 99.45%)f 0.09( 0.03%) | 16.27 |
| (1.84728) LP ( 2) O 8<br>s( 0.18%)p99.99( 99.81%)d 0.06( 0.01%) | (0.28440) LV ( 1)Tb 1<br>s( 0.00%)p 1.00( 0.17%)d99.99( 99.78%)f 0.24( 0.04%) | 7.75 |
| (1.84728) LP ( 2) O 8<br>s( 0.18%)p99.99( 99.81%)d 0.06( 0.01%) | (0.28093) LV ( 2)Tb 1<br>s( 0.02%)p 9.05( 0.17%)d99.99( 99.74%)f 3.76( 0.07%) | 15.93 |
| (1.90321) LP ( 1) O 9<br>s( 39.13%)p 1.56( 60.86%)d 0.00( 0.01%) | (0.26365) LV ( 3)Tb 1<br>s( 0.30%)p 0.74( 0.22%)d99.99( 99.45%)f 0.09( 0.03%) | 61.40 |
| (1.84056) LP ( 2) O 9<br>s( 1.11%)p89.29( 98.88%)d 0.01( 0.01%) | (0.28093) LV ( 2)Tb 1<br>s( 0.02%)p 9.05( 0.17%)d99.99( 99.74%)f 3.76( 0.07%) | 26.04 |
| (1.88575) LP ( 1) N 10<br>s( 46.95%)p 1.13( 53.02%)d 0.00( 0.03%) | (0.28440) LV ( 1)Tb 1<br>s( 0.00%)p 1.00( 0.17%)d99.99( 99.78%)f 0.24( 0.04%) | 28.59 |
| (1.88575) LP ( 1) N 10<br>s( 46.95%)p 1.13( 53.02%)d 0.00( 0.03%) | (0.28093) LV ( 2)Tb 1<br>s( 0.02%)p 9.05( 0.17%)d99.99( 99.74%)f 3.76( 0.07%) | 12.22 |
| (1.88575) LP ( 1) N 10<br>s( 46.95%)p 1.13( 53.02%)d 0.00( 0.03%) | (0.26365) LV ( 3)Tb 1<br>s( 0.30%)p 0.74( 0.22%)d99.99( 99.45%)f 0.09( 0.03%) | 16.14 |
| (1.88555) LP ( 1) N 11<br>s( 46.95%)p 1.13( 53.02%)d 0.00( 0.04%) | (0.28440) LV ( 1)Tb 1<br>s( 0.00%)p 1.00( 0.17%)d99.99( 99.78%)f 0.24( 0.04%) | 33.61 |
| (1.88555) LP ( 1) N 11<br>s( 46.95%)p 1.13( 53.02%)d 0.00( 0.04%) | (0.28093) LV ( 2)Tb 1<br>s( 0.02%)p 9.05( 0.17%)d99.99( 99.74%)f 3.76( 0.07%) | 9.71 |
| (1.88555) LP ( 1) N 11<br>s( 46.95%)p 1.13( 53.02%)d 0.00( 0.04%) | (0.26365) LV ( 3)Tb 1<br>s( 0.30%)p 0.74( 0.22%)d99.99( 99.45%)f 0.09( 0.03%) | 12.96 |

**Table S21.** Selected structural parameters of [$Tb^{IV}((OSiPh_2Ar)_3$-arene)($OCMe_3$)($MeCN)_2$] (**3-Tb$^{Ph}$-O$^t$Bu**)

| Atom Label | Bond distance (Å), DFT | |
|---|---|---|
| Tb1-O5 | 2.02 | 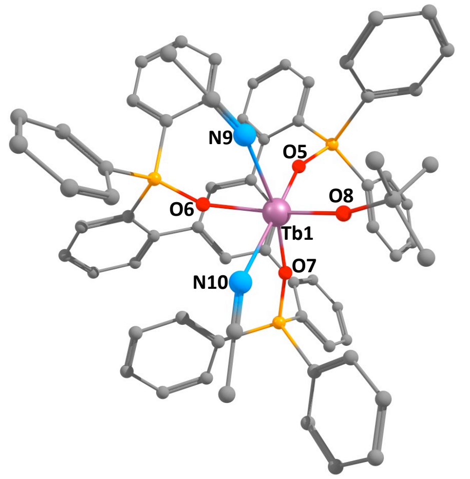 |
| Tb1-O6 | 2.09 | |
| Tb1-O7 | 2.03 | |
| Tb1-O8 | 2.02 | |
| Tb1-N9 | 2.53 | |
| Tb1-N10 | 2.57 | |
| Tb1-X (center of phenyl ring) | 4.15 | |

**Table S22.** Computed Natural charges for complex **3-Tb$^{Ph}$-O$^t$Bu**

| Atom Label | Natural charges |
|---|---|
| Tb1 | 2.47268 |
| O5 | -1.28318 |
| O6 | -1.30415 |
| O7 | -1.29378 |
| O8 | -0.93883 |
| N9 | -0.43942 |
| N10 | -0.43909 |

**Table S23.** Computed Wiberg bond index for complex **3-Tb$^{Ph}$-O$^t$Bu**

| Atom Label | Wiberg bond index | Atom Label | Wiberg bond index | Atom Label | Wiberg bond index |
|---|---|---|---|---|---|
| Tb1 | 0.0000 | Tb1 | 0.0000 | Tb1 | 0.0000 |
| O5 | 0.5257 | O6 | 0.4309 | O7 | 0.5051 |
| Atom Label | Wiberg bond index | Atom Label | Wiberg bond index | Atom Label | Wiberg bond index |
| Tb1 | 0.0000 | Tb1 | 0.0000 | Tb1 | 0.0000 |
| O8 | 0.5983 | N9 | 0.2022 | N10 | 0.1958 |

**Table S24.** Bonding orbitals between Ce and atoms in the first coordination sphere for **3-Tb$^{Ph}$-O$^t$Bu**

(1.93420) BD ( 1)Tb 1- O 7
( 5.52%) 0.2350*Tb 1 s( 0.10%)p 4.27( 0.44%)d99.99( 98.56%)f 8.74( 0.90%)
( 94.48%) 0.9720* O 7 s( 0.30%)p99.99( 99.69%)d 0.04( 0.01%)
(1.95983) BD ( 1)Tb 1- O 8
( 7.08%) 0.2661*Tb 1 s( 0.08%)p 6.66( 0.50%)d99.99( 98.52%)f12.04( 0.90%)
( 92.92%) 0.9640* O 8 s( 1.41%)p70.09( 98.59%)d 0.00( 0.00%)
(1.95619) BD ( 2)Tb 1- O 8
( 6.84%) 0.2616*Tb 1 s( 0.02%)p27.07( 0.54%)d99.99( 98.46%)f48.65( 0.98%)
( 93.16%) 0.9652* O 8 s( 0.05%)p99.99( 99.94%)d 0.13( 0.01%)

**Table S25.** Second order perturbation analysis for complex **3-Tb$^{Ph}$-O$^t$Bu**

| Donor NBO | Acceptor NBO | E(2) kcal/mol |
|---|---|---|
| (1.88881) LP ( 1) O 5 s( 36.19%)p 1.76( 63.80%)d 0.00( 0.02%) | (0.28303) LV ( 1)Tb 1 s( 0.04%)p 6.68( 0.24%)d99.99( 99.67%)f 1.63( 0.06%) | 49.28 |
| (1.88881) LP ( 1) O 5 s( 36.19%)p 1.76( 63.80%)d 0.00( 0.02%) | (0.26227) LV ( 2)Tb 1 s( 0.74%)p 0.24( 0.18%)d99.99( 99.05%)f 0.04( 0.03%) | 6.56 |
| (1.88881) LP ( 1) O 5 s( 36.19%)p 1.76( 63.80%)d 0.00( 0.02%) | (0.10486) LV ( 3)Tb 1 s( 98.54%)p 0.00( 0.10%)d 0.01( 1.32%)f 0.00( 0.05%) | 6.09 |
| (1.85001) LP ( 2) O 5 s( 0.00%)p 1.00( 99.98%)d 0.00( 0.01%) | (0.16527) BD*( 1)Tb 1- O 8 ( 92.92%) 0.9640* Tb 1 s( 0.08%)p 6.66( 0.50%)d99.99( 98.52%)f12.04( 0.90%) ( 7.08%) -0.2661* O 8 s( 1.41%)p70.09( 98.59%)d 0.00( 0.00%) | 7.25 |
| (1.85001) LP ( 2) O 5 s( 0.00%)p 1.00( 99.98%)d 0.00( 0.01%) | (0.17948) BD*( 2)Tb 1- O 8 ( 93.16%) 0.9652* Tb 1 s( 0.02%)p27.07( 0.54%)d99.99( 98.46%)f48.65( 0.98%) ( 6.84%) -0.2616* O 8 s( 0.05%)p99.99( 99.94%)d 0.13( 0.01%) | 6.34 |
| (1.82744) LP ( 3) O 5 s( 0.01%)p 1.00( 99.98%)d 0.00( 0.01%) | (0.14313) BD*( 1)Tb 1- O 7 ( 94.48%) 0.9720* Tb 1 s( 0.10%)p 4.27( 0.44%)d99.99( 98.56%)f 8.74( 0.90%) ( 5.52%) -0.2350* O 7 s( 0.30%)p99.99( 99.69%)d 0.04( 0.01%) | 21.07 |
| (1.90567) LP ( 1) O 6 | (0.26227) LV ( 2)Tb 1 | 43.07 |

| | | |
|---|---|---|
| s( 33.92%)p 1.95( 66.06%)d 0.00( 0.01%) | s( 0.74%)p 0.24( 0.18%)d99.99( 99.05%)f 0.04( 0.03%) | |
| (1.90567) LP ( 1) O 6<br>s( 33.92%)p 1.95( 66.06%)d 0.00( 0.01%) | (0.10486) LV ( 3)Tb 1<br>s( 98.54%)p 0.00( 0.10%)d 0.01( 1.32%)f 0.00( 0.05%) | 12.35 |
| (1.84641) LP ( 2) O 6<br>s( 2.40%)p40.58( 97.58%)d 0.01( 0.02%) | (0.16527) BD*( 1)Tb 1- O 8<br>( 92.92%) 0.9640* Tb 1 s( 0.08%)p 6.66( 0.50%)d99.99( 98.52%)f12.04( 0.90%)<br>( 7.08%) -0.2661* O 8 s( 1.41%)p70.09( 98.59%)d 0.00( 0.00%) | 20.04 |
| (1.84282) LP ( 3) O 6<br>s( 1.18%)p83.45( 98.80%)d 0.01( 0.02%) | (0.17948) BD*( 2)Tb 1- O 8<br>( 93.16%) 0.9652*Tb 1 s( 0.02%)p27.07( 0.54%)d99.99( 98.46%)f48.65( 0.98%)<br>( 6.84%) -0.2616* O 8 s( 0.05%)p99.99( 99.94%)d 0.13( 0.01%) | 13.10 |
| (1.89231) LP ( 1) O 7<br>s( 34.83%)p 1.87( 65.16%)d 0.00( 0.01%) | (0.28303) LV ( 1)Tb 1<br>s( 0.04%)p 6.68( 0.24%)d99.99( 99.67%)f 1.63( 0.06%) | 37.59 |
| (1.89231) LP ( 1) O 7<br>s( 34.83%)p 1.87( 65.16%)d 0.00( 0.01%) | (0.26227) LV ( 2)Tb 1<br>s( 0.74%)p 0.24( 0.18%)d99.99( 99.05%)f 0.04( 0.03%) | 26.58 |
| (1.89231) LP ( 1) O 7<br>s( 34.83%)p 1.87( 65.16%)d 0.00( 0.01%) | (0.10486) LV ( 3)Tb 1<br>s( 98.54%)p 0.00( 0.10%)d 0.01( 1.32%)f 0.00( 0.05%) | 6.61 |
| (1.85031) LP ( 2) O 7<br>s( 0.85%)p99.99( 99.14%)d 0.01( 0.01%) | (0.16527) BD*( 1)Tb 1- O 8<br>( 92.92%) 0.9640* Tb 1 s( 0.08%)p 6.66( 0.50%)d99.99( 98.52%)f12.04( 0.90%)<br>( 7.08%) -0.2661* O 8 s( 1.41%)p70.09( 98.59%)d 0.00( 0.00%) | 8.71 |
| (1.85031) LP ( 2) O 7<br>s( 0.85%)p99.99( 99.14%)d 0.01( 0.01%) | (0.17948) BD*( 2)Tb 1- O 8<br>( 93.16%) 0.9652* Tb 1 s( 0.02%)p27.07( 0.54%)d99.99( 98.46%)f48.65( 0.98%)<br>( 6.84%) -0.2616* O 8 s( 0.05%)p99.99( 99.94%)d 0.13( 0.01%) | 11.70 |
| (1.89106) LP ( 1) O 8<br>s( 58.42%)p 0.71( 41.56%)d 0.00( 0.02%) | (0.26227) LV ( 2)Tb 1<br>s( 0.74%)p 0.24( 0.18%)d99.99( 99.05%)f 0.04( 0.03%) | 58.53 |
| (1.89106) LP ( 1) O 8<br>s( 58.42%)p 0.71( 41.56%)d 0.00( 0.02%) | (0.10486) LV ( 3)Tb 1<br>s( 98.54%)p 0.00( 0.10%)d 0.01( 1.32%)f 0.00( 0.05%) | 14.23 |
| (1.87958) LP ( 1) N 9<br>s( 47.05%)p 1.12( 52.92%)d 0.00( 0.03%) | (0.28303) LV ( 1)Tb 1<br>s( 0.04%)p 6.68( 0.24%)d99.99( 99.67%)f 1.63( 0.06%) | 27.89 |
| (1.87958) LP ( 1) N 9<br>s( 47.05%)p 1.12( 52.92%)d 0.00( 0.03%) | (0.26227) LV ( 2)Tb 1<br>s( 0.74%)p 0.24( 0.18%)d99.99( 99.05%)f 0.04( 0.03%) | 24.82 |
| (1.87958) LP ( 1) N 9<br>s( 47.05%)p 1.12( 52.92%)d 0.00( 0.03%) | (0.10486) LV ( 3)Tb 1<br>s( 98.54%)p 0.00( 0.10%)d 0.01( 1.32%)f 0.00( 0.05%) | 7.75 |
| (1.88015) LP ( 1) N 10<br>s( 48.16%)p 1.08( 51.80%)d 0.00( 0.04%) | (0.28303) LV ( 1)Tb 1<br>s( 0.04%)p 6.68( 0.24%)d99.99( 99.67%)f 1.63( 0.06%) | 42.55 |
| (1.88015) LP ( 1) N 10<br>s( 48.16%)p 1.08( 51.80%)d 0.00( 0.04%) | (0.26227) LV ( 2)Tb 1<br>s( 0.74%)p 0.24( 0.18%)d99.99( 99.05%)f 0.04( 0.03%) | 6.56 |
| (1.88015) LP ( 1) N 10<br>s( 48.16%)p 1.08( 51.80%)d 0.00( 0.04%) | (0.10486) LV ( 3)Tb 1<br>s( 98.54%)p 0.00( 0.10%)d 0.01( 1.32%)f 0.00( 0.05%) | 7.07 |

Optimized geometries

**1-Ce$^{Ph}$**

| | | | |
|---|---|---|---|
| Ce | 3.014189000 | 2.330986000 | 17.375604000 |
| Si | 3.863528000 | 2.428644000 | 21.058055000 |
| Si | 4.356265000 | 5.626556000 | 16.122934000 |
| Si | 5.539612000 | -0.116412000 | 15.990114000 |
| O | 3.399218000 | 2.677203000 | 19.512477000 |
| O | 3.878987000 | 4.090384000 | 16.397393000 |
| O | 4.422653000 | 0.762080000 | 16.796103000 |
| O | 1.319121000 | 0.405680000 | 17.914221000 |
| O | 0.765342000 | 3.603036000 | 17.864625000 |
| O | 1.627724000 | 1.997218000 | 15.178071000 |
| C | 6.450103000 | 3.688948000 | 19.478553000 |
| C | 6.575330000 | 4.477305000 | 18.330325000 |
| H | 6.374656000 | 5.540727000 | 18.390376000 |
| C | 6.970491000 | 3.919802000 | 17.112586000 |
| C | 7.268153000 | 2.554307000 | 17.058470000 |
| H | 7.610931000 | 2.119411000 | 16.126925000 |
| C | 7.140067000 | 1.743350000 | 18.188512000 |
| C | 6.748181000 | 2.326781000 | 19.397710000 |
| H | 6.690724000 | 1.713969000 | 20.289561000 |
| C | 6.086757000 | 4.304099000 | 20.777352000 |
| C | 6.794694000 | 5.443270000 | 21.179427000 |
| H | 7.560127000 | 5.842889000 | 20.520399000 |
| C | 6.547629000 | 6.051551000 | 22.405512000 |
| H | 7.109307000 | 6.935559000 | 22.694120000 |
| C | 5.591096000 | 5.512903000 | 23.261021000 |
| H | 5.399130000 | 5.967227000 | 24.229163000 |
| C | 4.867499000 | 4.391709000 | 22.859075000 |
| H | 4.098995000 | 4.000204000 | 23.519937000 |
| C | 5.076472000 | 3.776365000 | 21.615368000 |
| C | 2.307916000 | 2.575305000 | 22.135884000 |
| C | 1.694723000 | 3.823705000 | 22.331235000 |
| H | 2.183060000 | 4.722427000 | 21.962951000 |
| C | 0.473010000 | 3.937435000 | 22.990658000 |
| H | 0.027129000 | 4.917191000 | 23.142810000 |
| C | -0.176318000 | 2.793306000 | 23.456239000 |
| H | -1.130915000 | 2.877887000 | 23.968357000 |
| C | 0.409106000 | 1.543379000 | 23.265811000 |
| H | -0.089585000 | 0.648460000 | 23.628791000 |
| C | 1.639763000 | 1.440693000 | 22.616848000 |
| H | 2.091155000 | 0.460925000 | 22.481256000 |
| C | 4.545757000 | 0.682606000 | 21.276159000 |
| C | 4.843370000 | 0.173243000 | 22.548238000 |
| H | 4.634860000 | 0.774558000 | 23.431139000 |
| C | 5.410489000 | -1.090988000 | 22.703346000 |
| H | 5.634718000 | -1.467731000 | 23.697842000 |
| C | 5.705572000 | -1.862974000 | 21.580135000 |
| H | 6.169738000 | -2.838634000 | 21.695217000 |
| C | 5.422604000 | -1.370758000 | 20.307373000 |
| H | 5.694759000 | -1.945385000 | 19.426959000 |
| C | 4.837317000 | -0.115224000 | 20.159195000 |
| H | 4.655222000 | 0.260935000 | 19.155074000 |
| C | 7.143311000 | 4.781259000 | 15.917614000 |
| C | 8.366196000 | 4.720812000 | 15.239013000 |
| H | 9.126982000 | 4.031525000 | 15.593986000 |
| C | 8.623552000 | 5.535920000 | 14.141735000 |
| H | 9.580405000 | 5.470376000 | 13.631503000 |
| C | 7.655944000 | 6.440028000 | 13.713000000 |

| | | | |
|---|---|---|---|
| H | 7.848888000 | 7.093349000 | 12.866575000 |
| C | 6.427155000 | 6.489500000 | 14.368706000 |
| H | 5.665414000 | 7.173042000 | 14.003184000 |
| C | 6.132978000 | 5.661089000 | 15.462439000 |
| C | 3.223673000 | 6.326033000 | 14.771436000 |
| C | 3.338356000 | 5.866341000 | 13.449535000 |
| H | 4.155337000 | 5.199648000 | 13.184172000 |
| C | 2.428043000 | 6.248080000 | 12.466758000 |
| H | 2.546429000 | 5.890376000 | 11.447139000 |
| C | 1.363568000 | 7.089247000 | 12.791957000 |
| H | 0.648749000 | 7.384842000 | 12.028945000 |
| C | 1.225670000 | 7.551895000 | 14.099134000 |
| H | 0.401145000 | 8.210868000 | 14.358545000 |
| C | 2.150908000 | 7.176461000 | 15.073608000 |
| H | 2.038765000 | 7.552822000 | 16.087609000 |
| C | 4.130556000 | 6.673667000 | 17.676673000 |
| C | 4.281992000 | 8.067111000 | 17.646506000 |
| H | 4.487091000 | 8.565736000 | 16.701118000 |
| C | 4.181483000 | 8.827700000 | 18.810708000 |
| H | 4.302228000 | 9.906935000 | 18.767080000 |
| C | 3.942891000 | 8.199851000 | 20.032792000 |
| H | 3.887277000 | 8.786699000 | 20.945613000 |
| C | 3.794231000 | 6.814763000 | 20.083375000 |
| H | 3.654663000 | 6.314356000 | 21.037326000 |
| C | 3.874444000 | 6.061697000 | 18.913828000 |
| H | 3.779039000 | 4.980200000 | 18.979377000 |
| C | 7.499513000 | 0.305851000 | 18.124907000 |
| C | 8.410306000 | -0.178196000 | 19.071811000 |
| H | 8.792039000 | 0.504047000 | 19.825988000 |
| C | 8.843522000 | -1.499523000 | 19.048310000 |
| H | 9.549560000 | -1.851139000 | 19.795386000 |
| C | 8.380392000 | -2.357618000 | 18.055216000 |
| H | 8.723962000 | -3.387521000 | 18.012231000 |
| C | 7.457700000 | -1.888872000 | 17.122041000 |
| H | 7.077860000 | -2.576759000 | 16.371578000 |
| C | 6.978916000 | -0.569783000 | 17.142611000 |
| C | 4.692959000 | -1.729143000 | 15.458412000 |
| C | 4.399581000 | -2.728756000 | 16.399851000 |
| H | 4.771393000 | -2.631575000 | 17.416413000 |
| C | 3.644642000 | -3.848737000 | 16.058418000 |
| H | 3.444253000 | -4.615868000 | 16.802200000 |
| C | 3.146608000 | -3.984040000 | 14.762015000 |
| H | 2.555374000 | -4.855015000 | 14.492548000 |
| C | 3.414743000 | -2.998149000 | 13.814659000 |
| H | 3.030865000 | -3.097857000 | 12.802713000 |
| C | 4.183553000 | -1.887247000 | 14.161904000 |
| H | 4.394840000 | -1.128851000 | 13.412023000 |
| C | 6.113574000 | 0.778185000 | 14.430420000 |
| C | 6.854595000 | 0.121083000 | 13.437797000 |
| H | 7.075770000 | -0.939217000 | 13.542862000 |
| C | 7.317977000 | 0.805037000 | 12.314588000 |
| H | 7.890882000 | 0.278253000 | 11.555912000 |
| C | 7.057499000 | 2.167861000 | 12.172933000 |
| H | 7.433251000 | 2.707308000 | 11.307612000 |
| C | 6.328909000 | 2.840203000 | 13.153003000 |
| H | 6.159773000 | 3.910340000 | 13.072932000 |
| C | 5.855848000 | 2.147080000 | 14.264572000 |
| H | 5.314653000 | 2.690302000 | 15.034217000 |
| C | 1.351818000 | -0.138845000 | 19.251297000 |
| H | 0.354531000 | -0.033800000 | 19.697682000 |

| | | | |
|---|---|---|---|
| H | 2.060732000 | 0.447907000 | 19.837859000 |
| C | 1.747122000 | -1.594077000 | 19.076968000 |
| H | 1.426116000 | -2.218187000 | 19.914444000 |
| H | 2.834408000 | -1.673091000 | 18.985729000 |
| C | 1.070682000 | -1.941778000 | 17.751014000 |
| H | -0.000354000 | -2.119609000 | 17.901590000 |
| H | 1.505326000 | -2.812669000 | 17.257398000 |
| C | 1.301087000 | -0.673806000 | 16.943481000 |
| H | 2.271349000 | -0.694343000 | 16.437887000 |
| H | 0.517454000 | -0.457186000 | 16.214070000 |
| C | 0.074530000 | 3.194325000 | 19.071359000 |
| H | 0.826195000 | 2.846232000 | 19.786402000 |
| H | -0.593724000 | 2.369760000 | 18.811723000 |
| C | -0.636744000 | 4.440973000 | 19.575491000 |
| H | -0.755848000 | 4.420052000 | 20.660148000 |
| H | -1.626609000 | 4.538347000 | 19.115195000 |
| C | 0.292073000 | 5.552966000 | 19.086497000 |
| H | -0.183606000 | 6.535596000 | 19.039659000 |
| H | 1.175832000 | 5.628147000 | 19.727154000 |
| C | 0.697903000 | 5.037373000 | 17.717812000 |
| H | -0.047866000 | 5.276253000 | 16.948106000 |
| H | 1.673418000 | 5.389760000 | 17.381522000 |
| C | 0.294580000 | 2.473763000 | 14.925710000 |
| H | -0.080287000 | 2.900841000 | 15.857099000 |
| H | -0.337817000 | 1.622729000 | 14.635594000 |
| C | 0.450186000 | 3.464256000 | 13.784403000 |
| H | 0.822339000 | 4.423321000 | 14.159054000 |
| H | -0.483298000 | 3.638773000 | 13.243366000 |
| C | 1.524946000 | 2.776989000 | 12.941134000 |
| H | 2.041161000 | 3.462906000 | 12.267585000 |
| H | 1.086452000 | 1.967928000 | 12.346376000 |
| C | 2.458459000 | 2.223919000 | 14.009007000 |
| H | 2.943095000 | 1.281369000 | 13.743560000 |
| H | 3.221355000 | 2.955007000 | 14.288881000 |

**2-Tb$^{Ph}$**

| | | | |
|---|---|---|---|
| Tb | 18.889067000 | 9.372392000 | 15.235495000 |
| K | 15.915708000 | 9.141509000 | 17.290434000 |
| Si | 15.817901000 | 7.987571000 | 13.806087000 |
| Si | 21.416720000 | 6.747565000 | 15.530185000 |
| Si | 20.111185000 | 12.097167000 | 13.150717000 |
| Si | 19.181726000 | 10.042595000 | 18.699280000 |
| O | 16.973478000 | 8.467971000 | 14.870102000 |
| O | 20.515487000 | 8.110300000 | 15.570119000 |
| O | 19.244455000 | 11.138626000 | 14.157598000 |
| O | 18.379417000 | 9.893035000 | 17.269255000 |
| C | 18.625411000 | 7.140995000 | 12.239800000 |
| C | 19.941191000 | 6.841094000 | 12.610699000 |
| H | 20.166140000 | 5.863356000 | 13.017053000 |
| C | 20.969731000 | 7.776043000 | 12.460956000 |
| C | 20.671291000 | 9.021685000 | 11.895651000 |
| H | 21.465953000 | 9.744517000 | 11.753012000 |
| C | 19.368358000 | 9.342905000 | 11.500385000 |
| C | 18.355870000 | 8.389590000 | 11.667897000 |
| H | 17.355177000 | 8.613310000 | 11.319348000 |
| C | 17.562894000 | 6.113433000 | 12.363169000 |
| C | 17.850685000 | 4.830333000 | 11.883163000 |
| H | 18.834374000 | 4.636733000 | 11.466265000 |
| C | 16.901912000 | 3.814849000 | 11.916481000 |
| H | 17.153208000 | 2.827948000 | 11.538889000 |

| | | | |
|---|---|---|---|
| C | 15.633495000 | 4.074916000 | 12.424405000 |
| H | 14.876417000 | 3.295992000 | 12.444282000 |
| C | 15.343194000 | 5.344061000 | 12.920569000 |
| H | 14.357530000 | 5.526862000 | 13.339446000 |
| C | 16.289441000 | 6.380723000 | 12.921116000 |
| C | 14.299369000 | 7.619914000 | 14.899542000 |
| C | 14.373716000 | 6.579120000 | 15.845732000 |
| H | 15.212749000 | 5.887294000 | 15.810576000 |
| C | 13.401357000 | 6.420792000 | 16.832898000 |
| H | 13.478944000 | 5.606227000 | 17.548494000 |
| C | 12.321671000 | 7.306357000 | 16.892266000 |
| H | 11.559073000 | 7.186010000 | 17.656757000 |
| C | 12.220259000 | 8.335790000 | 15.956833000 |
| H | 11.377477000 | 9.020910000 | 15.993617000 |
| C | 13.200302000 | 8.489206000 | 14.973276000 |
| H | 13.120778000 | 9.305615000 | 14.260907000 |
| C | 15.353846000 | 9.380450000 | 12.627870000 |
| C | 14.238072000 | 9.278219000 | 11.784973000 |
| H | 13.608592000 | 8.391240000 | 11.826370000 |
| C | 13.923032000 | 10.295243000 | 10.884028000 |
| H | 13.053254000 | 10.199451000 | 10.239716000 |
| C | 14.734504000 | 11.426111000 | 10.800100000 |
| H | 14.503645000 | 12.211053000 | 10.085551000 |
| C | 15.855698000 | 11.538632000 | 11.620807000 |
| H | 16.517477000 | 12.394361000 | 11.528389000 |
| C | 16.153226000 | 10.528783000 | 12.531158000 |
| H | 17.044544000 | 10.627132000 | 13.144019000 |
| C | 22.360384000 | 7.442972000 | 12.855146000 |
| C | 23.386286000 | 7.708947000 | 11.941306000 |
| H | 23.133933000 | 8.142162000 | 10.977527000 |
| C | 24.712392000 | 7.416906000 | 12.244386000 |
| H | 25.492244000 | 7.632508000 | 11.519533000 |
| C | 25.030845000 | 6.851429000 | 13.475446000 |
| H | 26.063122000 | 6.620771000 | 13.723935000 |
| C | 24.016493000 | 6.600884000 | 14.398529000 |
| H | 24.276064000 | 6.203810000 | 15.376797000 |
| C | 22.673249000 | 6.887402000 | 14.118171000 |
| C | 20.300690000 | 5.233894000 | 15.331329000 |
| C | 20.828498000 | 3.978346000 | 14.998484000 |
| H | 21.901310000 | 3.867407000 | 14.853039000 |
| C | 19.999825000 | 2.872241000 | 14.812648000 |
| H | 20.430144000 | 1.909423000 | 14.550012000 |
| C | 18.618882000 | 3.007619000 | 14.944298000 |
| H | 17.966958000 | 2.155248000 | 14.776684000 |
| C | 18.074144000 | 4.250478000 | 15.260468000 |
| H | 16.996124000 | 4.370892000 | 15.310418000 |
| C | 18.908240000 | 5.347651000 | 15.455368000 |
| H | 18.458821000 | 6.316372000 | 15.655391000 |
| C | 22.334069000 | 6.576883000 | 17.162498000 |
| C | 22.728658000 | 5.341985000 | 17.693527000 |
| H | 22.556318000 | 4.429501000 | 17.127933000 |
| C | 23.303103000 | 5.256103000 | 18.959855000 |
| H | 23.595259000 | 4.288797000 | 19.360360000 |
| C | 23.480837000 | 6.410393000 | 19.720645000 |
| H | 23.910087000 | 6.344619000 | 20.716873000 |
| C | 23.097071000 | 7.647396000 | 19.206044000 |
| H | 23.222926000 | 8.550727000 | 19.794126000 |
| C | 22.533918000 | 7.729003000 | 17.935790000 |
| H | 22.210827000 | 8.693039000 | 17.554333000 |
| C | 19.082702000 | 10.635814000 | 10.830061000 |

| | | | |
|---|---|---|---|
| C | 18.425111000 | 10.590608000 | 9.595038000 |
| H | 18.128208000 | 9.625463000 | 9.194442000 |
| C | 18.167323000 | 11.751113000 | 8.872859000 |
| H | 17.655818000 | 11.690935000 | 7.916401000 |
| C | 18.579549000 | 12.980751000 | 9.377354000 |
| H | 18.398338000 | 13.894268000 | 8.817793000 |
| C | 19.215651000 | 13.035024000 | 10.616468000 |
| H | 19.501825000 | 14.003616000 | 11.017049000 |
| C | 19.466887000 | 11.882824000 | 11.376746000 |
| C | 19.795871000 | 13.883970000 | 13.677556000 |
| C | 18.529296000 | 14.462572000 | 13.496674000 |
| H | 17.772607000 | 13.932686000 | 12.923447000 |
| C | 18.213554000 | 15.702074000 | 14.045284000 |
| H | 17.224610000 | 16.126609000 | 13.895600000 |
| C | 19.166428000 | 16.392262000 | 14.795542000 |
| H | 18.923239000 | 17.358574000 | 15.228919000 |
| C | 20.429096000 | 15.835751000 | 14.989451000 |
| H | 21.173768000 | 16.366645000 | 15.576332000 |
| C | 20.737058000 | 14.594439000 | 14.433564000 |
| H | 21.722789000 | 14.167353000 | 14.595682000 |
| C | 21.954973000 | 11.742048000 | 13.310460000 |
| C | 22.895852000 | 12.547012000 | 12.650613000 |
| H | 22.558413000 | 13.398942000 | 12.063360000 |
| C | 24.260164000 | 12.274096000 | 12.733806000 |
| H | 24.973495000 | 12.908894000 | 12.214771000 |
| C | 24.707115000 | 11.181305000 | 13.477050000 |
| H | 25.769013000 | 10.958532000 | 13.534184000 |
| C | 23.788443000 | 10.369462000 | 14.138826000 |
| H | 24.123244000 | 9.507342000 | 14.706960000 |
| C | 22.427766000 | 10.653151000 | 14.056829000 |
| H | 21.737002000 | 9.992072000 | 14.571135000 |
| C | 19.395929000 | 8.339332000 | 19.466620000 |
| C | 19.374982000 | 7.199959000 | 18.651931000 |
| H | 19.215506000 | 7.312057000 | 17.584759000 |
| C | 19.618902000 | 5.931800000 | 19.171703000 |
| H | 19.625450000 | 5.072343000 | 18.508317000 |
| C | 19.892144000 | 5.785088000 | 20.530159000 |
| H | 20.104257000 | 4.801152000 | 20.938731000 |
| C | 19.900438000 | 6.903760000 | 21.363591000 |
| H | 20.106798000 | 6.789248000 | 22.424486000 |
| C | 19.647620000 | 8.167748000 | 20.835457000 |
| H | 19.642489000 | 9.031906000 | 21.496689000 |
| C | 20.841054000 | 10.895511000 | 18.465399000 |
| C | 21.760721000 | 10.931259000 | 19.524711000 |
| H | 21.499439000 | 10.478993000 | 20.478790000 |
| C | 23.013824000 | 11.518044000 | 19.369839000 |
| H | 23.714938000 | 11.530844000 | 20.200124000 |
| C | 23.372178000 | 12.078368000 | 18.143225000 |
| H | 24.354327000 | 12.524393000 | 18.014022000 |
| C | 22.469885000 | 12.061603000 | 17.082762000 |
| H | 22.744869000 | 12.482694000 | 16.121714000 |
| C | 21.213882000 | 11.479211000 | 17.247444000 |
| H | 20.523118000 | 11.491998000 | 16.408439000 |
| C | 18.164818000 | 11.058070000 | 19.934570000 |
| C | 18.494628000 | 12.380361000 | 20.260779000 |
| H | 19.405750000 | 12.814774000 | 19.856927000 |
| C | 17.676727000 | 13.145087000 | 21.092449000 |
| H | 17.949454000 | 14.169922000 | 21.328877000 |
| C | 16.507263000 | 12.596004000 | 21.617389000 |
| H | 15.865255000 | 13.191934000 | 22.259962000 |

| | | | |
|---|---|---|---|
| C | 16.175529000 | 11.270882000 | 21.331862000 |
| H | 15.281022000 | 10.828952000 | 21.763396000 |
| C | 17.005541000 | 10.513089000 | 20.508409000 |
| H | 16.766674000 | 9.463853000 | 20.332136000 |
| C | 16.643279000 | 12.761156000 | 16.672064000 |
| C | 16.118074000 | 12.347358000 | 15.439465000 |
| H | 16.769227000 | 12.347622000 | 14.571677000 |
| C | 14.793468000 | 11.923291000 | 15.322575000 |
| H | 14.420788000 | 11.604291000 | 14.353983000 |
| C | 13.964164000 | 11.901841000 | 16.448115000 |
| H | 12.930751000 | 11.577642000 | 16.361148000 |
| C | 14.478126000 | 12.302778000 | 17.686259000 |
| H | 13.844557000 | 12.292554000 | 18.570148000 |
| C | 15.805114000 | 12.725778000 | 17.796294000 |
| H | 16.197255000 | 13.023980000 | 18.764219000 |
| C | 18.060059000 | 13.242912000 | 16.764215000 |
| H | 18.485732000 | 13.035355000 | 17.746051000 |
| H | 18.677177000 | 12.766216000 | 16.002310000 |
| H | 18.115403000 | 14.323364000 | 16.591628000 |

**3-Tb$^{Ph}$**

| | | | |
|---|---|---|---|
| Tb | 4.215131000 | 6.978173000 | 10.896513000 |
| Si | 5.673217000 | 3.985456000 | 12.256382000 |
| Si | 4.548526000 | 6.482571000 | 7.303562000 |
| Si | 0.681717000 | 6.489440000 | 11.506966000 |
| Si | 5.037030000 | 9.189969000 | 13.675605000 |
| O | 5.085579000 | 5.210974000 | 11.323559000 |
| O | 4.072564000 | 6.750048000 | 8.845711000 |
| O | 2.318844000 | 6.447528000 | 11.369933000 |
| O | 4.829128000 | 8.002669000 | 12.556517000 |
| N | 6.405182000 | 7.826661000 | 9.993121000 |
| N | 3.054756000 | 9.158008000 | 10.409078000 |
| C | 3.936268000 | 2.911996000 | 9.697881000 |
| C | 3.672172000 | 3.512765000 | 8.462121000 |
| H | 4.426553000 | 3.481059000 | 7.684386000 |
| C | 2.459443000 | 4.165209000 | 8.220816000 |
| C | 1.484984000 | 4.172041000 | 9.224275000 |
| H | 0.535744000 | 4.664092000 | 9.041055000 |
| C | 1.718694000 | 3.563723000 | 10.459603000 |
| C | 2.942752000 | 2.924697000 | 10.680099000 |
| H | 3.125539000 | 2.441361000 | 11.633567000 |
| C | 5.241543000 | 2.249481000 | 9.951218000 |
| C | 5.693457000 | 1.304237000 | 9.023282000 |
| H | 5.079933000 | 1.093312000 | 8.151936000 |
| C | 6.896315000 | 0.628930000 | 9.207133000 |
| H | 7.223949000 | -0.100751000 | 8.471950000 |
| C | 7.668715000 | 0.886634000 | 10.336009000 |
| H | 8.605719000 | 0.359984000 | 10.494341000 |
| C | 7.238225000 | 1.842192000 | 11.254868000 |
| H | 7.867513000 | 2.071512000 | 12.111360000 |
| C | 6.037695000 | 2.546390000 | 11.084583000 |
| C | 4.470155000 | 3.570154000 | 13.639818000 |
| C | 3.198120000 | 4.157863000 | 13.679734000 |
| H | 2.908077000 | 4.860287000 | 12.904563000 |
| C | 2.285982000 | 3.828098000 | 14.679481000 |
| H | 1.297288000 | 4.276210000 | 14.674600000 |
| C | 2.639898000 | 2.911012000 | 15.667884000 |
| H | 1.928368000 | 2.649155000 | 16.446248000 |
| C | 3.906802000 | 2.326503000 | 15.653327000 |
| H | 4.186898000 | 1.612713000 | 16.423350000 |

| | | | |
|---|---|---|---|
| C | 4.811054000 | 2.652304000 | 14.643616000 |
| H | 5.793810000 | 2.185019000 | 14.639136000 |
| C | 7.271747000 | 4.620167000 | 13.024535000 |
| C | 7.321395000 | 5.023030000 | 14.366065000 |
| H | 6.460850000 | 4.858344000 | 15.007894000 |
| C | 8.451323000 | 5.651322000 | 14.887178000 |
| H | 8.462400000 | 5.971695000 | 15.924807000 |
| C | 9.558418000 | 5.879285000 | 14.073784000 |
| H | 10.437843000 | 6.371901000 | 14.478251000 |
| C | 9.526363000 | 5.492863000 | 12.733524000 |
| H | 10.386052000 | 5.674966000 | 12.093393000 |
| C | 8.389477000 | 4.878386000 | 12.215478000 |
| H | 8.365598000 | 4.601342000 | 11.164723000 |
| C | 2.187606000 | 4.830833000 | 6.921152000 |
| C | 0.997784000 | 4.519254000 | 6.252600000 |
| H | 0.323140000 | 3.792838000 | 6.696898000 |
| C | 0.678141000 | 5.111024000 | 5.034211000 |
| H | -0.251499000 | 4.851556000 | 4.535478000 |
| C | 1.552685000 | 6.028821000 | 4.459454000 |
| H | 1.313882000 | 6.494887000 | 3.507505000 |
| C | 2.730152000 | 6.361516000 | 5.127126000 |
| H | 3.387013000 | 7.114198000 | 4.697560000 |
| C | 3.069086000 | 5.784683000 | 6.358954000 |
| C | 6.071303000 | 5.378282000 | 7.198501000 |
| C | 6.628251000 | 5.041464000 | 5.955971000 |
| H | 6.162634000 | 5.397988000 | 5.039197000 |
| C | 7.758117000 | 4.228827000 | 5.868461000 |
| H | 8.175310000 | 3.980884000 | 4.896119000 |
| C | 8.336530000 | 3.717282000 | 7.029486000 |
| H | 9.204326000 | 3.066663000 | 6.965663000 |
| C | 7.776210000 | 4.014494000 | 8.271680000 |
| H | 8.183552000 | 3.565665000 | 9.172646000 |
| C | 6.659609000 | 4.843515000 | 8.355040000 |
| H | 6.212305000 | 5.036813000 | 9.325269000 |
| C | 4.983512000 | 8.172182000 | 6.572193000 |
| C | 6.034154000 | 8.421805000 | 5.679388000 |
| H | 6.656028000 | 7.599500000 | 5.334768000 |
| C | 6.312765000 | 9.715758000 | 5.236268000 |
| H | 7.131721000 | 9.887995000 | 4.542504000 |
| C | 5.541190000 | 10.786600000 | 5.684402000 |
| H | 5.759091000 | 11.795294000 | 5.344343000 |
| C | 4.491640000 | 10.557982000 | 6.574314000 |
| H | 3.892195000 | 11.390367000 | 6.933402000 |
| C | 4.218255000 | 9.264346000 | 7.010415000 |
| H | 3.415585000 | 9.092132000 | 7.721553000 |
| C | 0.678497000 | 3.569772000 | 11.517340000 |
| C | 0.306079000 | 2.345486000 | 12.084696000 |
| H | 0.789074000 | 1.438253000 | 11.732871000 |
| C | -0.671889000 | 2.276361000 | 13.071784000 |
| H | -0.944554000 | 1.314180000 | 13.496104000 |
| C | -1.296980000 | 3.440927000 | 13.508928000 |
| H | -2.062584000 | 3.399208000 | 14.278558000 |
| C | -0.912754000 | 4.666863000 | 12.969938000 |
| H | -1.363191000 | 5.577405000 | 13.357222000 |
| C | 0.074718000 | 4.762284000 | 11.978362000 |
| C | -0.057543000 | 7.162283000 | 9.911647000 |
| C | 0.775662000 | 7.344449000 | 8.795928000 |
| H | 1.823257000 | 7.065504000 | 8.853849000 |
| C | 0.276460000 | 7.857441000 | 7.601099000 |
| H | 0.936516000 | 7.958527000 | 6.744513000 |

| | | | |
|---|---|---|---|
| C | -1.069918000 | 8.211044000 | 7.504798000 |
| H | -1.462985000 | 8.612593000 | 6.574704000 |
| C | -1.917028000 | 8.023917000 | 8.597739000 |
| H | -2.970583000 | 8.278881000 | 8.520048000 |
| C | -1.414328000 | 7.493674000 | 9.785977000 |
| H | -2.094589000 | 7.325557000 | 10.618132000 |
| C | 0.297795000 | 7.615707000 | 12.972946000 |
| C | 1.070412000 | 7.406717000 | 14.127522000 |
| H | 1.859634000 | 6.661774000 | 14.121975000 |
| C | 0.860210000 | 8.149759000 | 15.285334000 |
| H | 1.480944000 | 7.970910000 | 16.157870000 |
| C | -0.127708000 | 9.135646000 | 15.304647000 |
| H | -0.291922000 | 9.723665000 | 16.203427000 |
| C | -0.894310000 | 9.372194000 | 14.164589000 |
| H | -1.662884000 | 10.140956000 | 14.175049000 |
| C | -0.683304000 | 8.613962000 | 13.011228000 |
| H | -1.295135000 | 8.804676000 | 12.133081000 |
| C | 6.850598000 | 9.683117000 | 13.659375000 |
| C | 7.792982000 | 8.803208000 | 13.107863000 |
| H | 7.465291000 | 7.844029000 | 12.717934000 |
| C | 9.141770000 | 9.148410000 | 13.053545000 |
| H | 9.856630000 | 8.452315000 | 12.624584000 |
| C | 9.571508000 | 10.375797000 | 13.556002000 |
| H | 10.623762000 | 10.645070000 | 13.512788000 |
| C | 8.648361000 | 11.258143000 | 14.116750000 |
| H | 8.979170000 | 12.215615000 | 14.510525000 |
| C | 7.299645000 | 10.911403000 | 14.164604000 |
| H | 6.583858000 | 11.613358000 | 14.587373000 |
| C | 4.488403000 | 8.459940000 | 15.318907000 |
| C | 4.329890000 | 7.069465000 | 15.402147000 |
| H | 4.518027000 | 6.463597000 | 14.521965000 |
| C | 3.917949000 | 6.459244000 | 16.584737000 |
| H | 3.789385000 | 5.381231000 | 16.616992000 |
| C | 3.657849000 | 7.241676000 | 17.710675000 |
| H | 3.333704000 | 6.773372000 | 18.636311000 |
| C | 3.807695000 | 8.628056000 | 17.645971000 |
| H | 3.598659000 | 9.238522000 | 18.520604000 |
| C | 4.218868000 | 9.229898000 | 16.457384000 |
| H | 4.316925000 | 10.312563000 | 16.413827000 |
| C | 3.985125000 | 10.693814000 | 13.250238000 |
| C | 4.461330000 | 11.683494000 | 12.378998000 |
| H | 5.485152000 | 11.629932000 | 12.017404000 |
| C | 3.640987000 | 12.732276000 | 11.965932000 |
| H | 4.028148000 | 13.492905000 | 11.292830000 |
| C | 2.325578000 | 12.809187000 | 12.424534000 |
| H | 1.688025000 | 13.633577000 | 12.114019000 |
| C | 1.833464000 | 11.829633000 | 13.287889000 |
| H | 0.809373000 | 11.875882000 | 13.648252000 |
| C | 2.657979000 | 10.782012000 | 13.692794000 |
| H | 2.260625000 | 10.020117000 | 14.354680000 |
| C | 7.301314000 | 7.884237000 | 9.265835000 |
| C | 8.397360000 | 7.916741000 | 8.322470000 |
| H | 8.088272000 | 8.489759000 | 7.443784000 |
| H | 8.617590000 | 6.889342000 | 8.016679000 |
| H | 9.279841000 | 8.372180000 | 8.778030000 |
| C | 2.081203000 | 9.779562000 | 10.354942000 |
| C | 0.836361000 | 10.509184000 | 10.268013000 |
| H | 0.231801000 | 10.081317000 | 9.462829000 |
| H | 1.036904000 | 11.566666000 | 10.086058000 |
| H | 0.310794000 | 10.403571000 | 11.220059000 |

**3-Tb$^{OtBu}$**

| | | | |
|---|---|---|---|
| Tb | 4.237172000 | 7.141480000 | 11.017272000 |
| Si | 5.752516000 | 4.099915000 | 12.214780000 |
| Si | 4.470934000 | 6.548198000 | 7.408833000 |
| Si | 0.693271000 | 6.500949000 | 11.493416000 |
| O | 5.193796000 | 5.393622000 | 11.356448000 |
| O | 4.063578000 | 6.916686000 | 8.949795000 |
| O | 2.331669000 | 6.601681000 | 11.464234000 |
| O | 4.767558000 | 8.099879000 | 12.715527000 |
| N | 6.349362000 | 8.130465000 | 10.049686000 |
| N | 3.042842000 | 9.364314000 | 10.535757000 |
| C | 3.972415000 | 2.979636000 | 9.735275000 |
| C | 3.655325000 | 3.579896000 | 8.513506000 |
| H | 4.384403000 | 3.562335000 | 7.712638000 |
| C | 2.422882000 | 4.205861000 | 8.315996000 |
| C | 1.478951000 | 4.180311000 | 9.347530000 |
| H | 0.507866000 | 4.633107000 | 9.187129000 |
| C | 1.775549000 | 3.592979000 | 10.580041000 |
| C | 3.020285000 | 2.980441000 | 10.757049000 |
| H | 3.247121000 | 2.508188000 | 11.705651000 |
| C | 5.301170000 | 2.350615000 | 9.927676000 |
| C | 5.747230000 | 1.422681000 | 8.980740000 |
| H | 5.106597000 | 1.186897000 | 8.135658000 |
| C | 6.982424000 | 0.795973000 | 9.115461000 |
| H | 7.307541000 | 0.076950000 | 8.368654000 |
| C | 7.792781000 | 1.090518000 | 10.208165000 |
| H | 8.756705000 | 0.602954000 | 10.325405000 |
| C | 7.367420000 | 2.034224000 | 11.141428000 |
| H | 8.024977000 | 2.297777000 | 11.966214000 |
| C | 6.131759000 | 2.685247000 | 11.021374000 |
| C | 4.529231000 | 3.600429000 | 13.546544000 |
| C | 3.436995000 | 4.419363000 | 13.864416000 |
| H | 3.300313000 | 5.364265000 | 13.350383000 |
| C | 2.482500000 | 4.010140000 | 14.791170000 |
| H | 1.622406000 | 4.640971000 | 14.992399000 |
| C | 2.610477000 | 2.774922000 | 15.423677000 |
| H | 1.856879000 | 2.448813000 | 16.134972000 |
| C | 3.694741000 | 1.950180000 | 15.126141000 |
| H | 3.793040000 | 0.982741000 | 15.611568000 |
| C | 4.643261000 | 2.359883000 | 14.190179000 |
| H | 5.466470000 | 1.693920000 | 13.938108000 |
| C | 7.378475000 | 4.639503000 | 12.995287000 |
| C | 7.715697000 | 4.363492000 | 14.324965000 |
| H | 7.033814000 | 3.781787000 | 14.940344000 |
| C | 8.898311000 | 4.851909000 | 14.880404000 |
| H | 9.140667000 | 4.636782000 | 15.917619000 |
| C | 9.761438000 | 5.628024000 | 14.109759000 |
| H | 10.678857000 | 6.016273000 | 14.543746000 |
| C | 9.441800000 | 5.911913000 | 12.781350000 |
| H | 10.110674000 | 6.521824000 | 12.179618000 |
| C | 8.260963000 | 5.420082000 | 12.232361000 |
| H | 8.005551000 | 5.663414000 | 11.204047000 |
| C | 2.114956000 | 4.880415000 | 7.031579000 |
| C | 0.926552000 | 4.555185000 | 6.369699000 |
| H | 0.264759000 | 3.817489000 | 6.814567000 |
| C | 0.593000000 | 5.148844000 | 5.156213000 |
| H | -0.334994000 | 4.879042000 | 4.659938000 |
| C | 1.452317000 | 6.081480000 | 4.583084000 |
| H | 1.203827000 | 6.547814000 | 3.633671000 |

| | | | |
|---|---|---|---|
| C | 2.626369000 | 6.431266000 | 5.248632000 |
| H | 3.267728000 | 7.196293000 | 4.818171000 |
| C | 2.979235000 | 5.854077000 | 6.476170000 |
| C | 5.963636000 | 5.402447000 | 7.298996000 |
| C | 6.342800000 | 4.844015000 | 6.069003000 |
| H | 5.746035000 | 5.039466000 | 5.179784000 |
| C | 7.450729000 | 4.003328000 | 5.971586000 |
| H | 7.723407000 | 3.573485000 | 5.011305000 |
| C | 8.195593000 | 3.699315000 | 7.110611000 |
| H | 9.048757000 | 3.030368000 | 7.042580000 |
| C | 7.821463000 | 4.229163000 | 8.344067000 |
| H | 8.364739000 | 3.954768000 | 9.242991000 |
| C | 6.714509000 | 5.069581000 | 8.435753000 |
| H | 6.392518000 | 5.420408000 | 9.410241000 |
| C | 5.000647000 | 8.176255000 | 6.592742000 |
| C | 5.801868000 | 8.247935000 | 5.444293000 |
| H | 6.111759000 | 7.333485000 | 4.945381000 |
| C | 6.242359000 | 9.473059000 | 4.945025000 |
| H | 6.864895000 | 9.505602000 | 4.054753000 |
| C | 5.895333000 | 10.656246000 | 5.598515000 |
| H | 6.245907000 | 11.611982000 | 5.218376000 |
| C | 5.096809000 | 10.605916000 | 6.741765000 |
| H | 4.825915000 | 11.523924000 | 7.256788000 |
| C | 4.651597000 | 9.377549000 | 7.227691000 |
| H | 4.047229000 | 9.336979000 | 8.129116000 |
| C | 0.791917000 | 3.607395000 | 11.690039000 |
| C | 0.494678000 | 2.404011000 | 12.337898000 |
| H | 0.986301000 | 1.494447000 | 12.005036000 |
| C | -0.419681000 | 2.357300000 | 13.385692000 |
| H | -0.634951000 | 1.410181000 | 13.872332000 |
| C | -1.052247000 | 3.523528000 | 13.805871000 |
| H | -1.766247000 | 3.498074000 | 14.624365000 |
| C | -0.744946000 | 4.731848000 | 13.182694000 |
| H | -1.204385000 | 5.646400000 | 13.549610000 |
| C | 0.173519000 | 4.802770000 | 12.126477000 |
| C | -0.044521000 | 6.944462000 | 9.817757000 |
| C | 0.775443000 | 7.419929000 | 8.783411000 |
| H | 1.851227000 | 7.452702000 | 8.922242000 |
| C | 0.234530000 | 7.788678000 | 7.554184000 |
| H | 0.892204000 | 8.122037000 | 6.757650000 |
| C | -1.137090000 | 7.679600000 | 7.333376000 |
| H | -1.556513000 | 7.953003000 | 6.369169000 |
| C | -1.965248000 | 7.188941000 | 8.342545000 |
| H | -3.032404000 | 7.080465000 | 8.167562000 |
| C | -1.420294000 | 6.822675000 | 9.572463000 |
| H | -2.073425000 | 6.409899000 | 10.339047000 |
| C | 0.120890000 | 7.846175000 | 12.693991000 |
| C | 1.084444000 | 8.458988000 | 13.509607000 |
| H | 2.111817000 | 8.107191000 | 13.474029000 |
| C | 0.750134000 | 9.535495000 | 14.330727000 |
| H | 1.513752000 | 10.001072000 | 14.947166000 |
| C | -0.559250000 | 10.016386000 | 14.354625000 |
| H | -0.820774000 | 10.856745000 | 14.992036000 |
| C | -1.532579000 | 9.414075000 | 13.556213000 |
| H | -2.553805000 | 9.785571000 | 13.568926000 |
| C | -1.189994000 | 8.343117000 | 12.732827000 |
| H | -1.953153000 | 7.907599000 | 12.093140000 |
| C | 7.111469000 | 8.302785000 | 9.196985000 |
| C | 8.036249000 | 8.473892000 | 8.098120000 |
| H | 7.633104000 | 9.213550000 | 7.400356000 |

| | | | |
|---|---|---|---|
| H | 8.128988000 | 7.514084000 | 7.581435000 |
| H | 9.013511000 | 8.795404000 | 8.466504000 |
| C | 2.006035000 | 9.876413000 | 10.597827000 |
| C | 0.692590000 | 10.479184000 | 10.670917000 |
| H | -0.024508000 | 9.788531000 | 10.218575000 |
| H | 0.682462000 | 11.429690000 | 10.131583000 |
| H | 0.422431000 | 10.635859000 | 11.718993000 |
| C | 5.475560000 | 8.767338000 | 13.726142000 |
| C | 6.478309000 | 9.726689000 | 13.081551000 |
| H | 7.039883000 | 10.283236000 | 13.839593000 |
| H | 7.186028000 | 9.164264000 | 12.466527000 |
| H | 5.955308000 | 10.440532000 | 12.437113000 |
| C | 6.205578000 | 7.721815000 | 14.571025000 |
| H | 6.730302000 | 8.184114000 | 15.414201000 |
| H | 5.490635000 | 6.991364000 | 14.961127000 |
| H | 6.934550000 | 7.184918000 | 13.960192000 |
| C | 4.469459000 | 9.543465000 | 14.579671000 |
| H | 3.898150000 | 10.230226000 | 13.947113000 |
| H | 3.771276000 | 8.848683000 | 15.056679000 |
| H | 4.970219000 | 10.120996000 | 15.363839000 |

## S12. References


1. R. J. LeSuer, C. Buttolph, and W. E. Geiger, *Analytical Chemistry*, 2004, **76** (21), 6395-6401.
2. R. F. Jordan, C. S. Bajgur, W. E. Dasher and A. L. Rheingold, *Organometallics*, 1987, **6**, 1041–1051.
3. A. R. Willauer, C. T. Palumbo, R. Scopelliti, I. Zivkovic, I. Douair, L. Maron and M. Mazzanti, *Angew. Chem. Int. Ed.*, 2020, **59**, 3549–3553.
4. D. C. Bradley, J. S. Ghotra and F. A. Hart, *J. Chem. Soc., Dalton Trans.*, 1973, 1021–1023.
5. P. B. Hitchcok, A. G Hulkes, M. F. Lappert, and Z. Li, *Dalton Trans.*, 2004, 129–136.
6. J. Hillenbrand, M. Leutzsch, E. Yiannakas, C. P. Gordon, C. Wille, N. Nöthling, C. Copéret and A. Fürstner, *J. Am. Chem. Soc.* 2020, **142**, 11279-11294.
7. F.-C. Hsueh,T. Rajeshkumar, R. Scopelliti, A. Sienkiewicz, L. Maron and M. Mazzanti, *Chem. Sci.* 2023, **14**, 6011-6021.
8. S. Stoll and A. Schweiger, *J. Magn. Reson.*, 2006, **178** (1), 42–55.
9. G. A. Bain and J. F. Berry, *Journal of Chemical Education*, 2008, **85** (4), 532.
10. **CrysAlisPro** (Rigaku, V1.171.42.72a, 2022)
11. G.M. Sheldrick, ShelXT-Integrated space-group and crystal-structure determination, *Acta Cryst.* 2015, **A71**, 3-8.
12. O.V. Dolomanov, L. J. Bourhis, R. J. Gildea, J. A. K. Howard and H. Puschmann, Olex2: A complete structure solution, refinement and analysis program, *J. Appl. Cryst.* 2009, **42**, 339-341.
13. G.M. Sheldrick, Crystal structure refinement with ShelXL, *Acta Cryst.* 2015, **C71**, 3-8.
14. A. D. Becke, *J. Chem. Phys*., 1993, **98**, 5648
15. A. Bergner, M. Dolg, W. Kuechle, H. Stoll, H. Preuss, *Mol. Phys*. 1993, **80**, 1431. M. Dolg, H. Stoll, A. Savin, H. Preuss, *Theor. Chim. Acta* 1989, **75**, 173; M. Dolg, H. Stoll, H. Preuss, *Theor. Chim. Acta* 1993, **85**, 441. T. Leininger, A. Nicklass, W. Kuechle, H. Stoll, M. Dolg and A. Bergner, *Chem. Phys. Lett*. 1996, **255**, 274.
16. A. Hollwarth, M. Bohme, S. Dapprich, A.W. Ehlers, A. Gobbi, V. Jonas, K.F. Kohler, R. Stegmann, A. Veldkamp, G. Frenking *J. Chem. Phys*. 1993, **208**, 237.
17. (a) R. Ditchfield, W. J. Hehre and J. A. Pople, *J. Chem. Phys*., 1971, **54**, 724; (b) W. J. Hehre, R. Ditchfield and J. A. Pople, *J. Chem. Phys*., 1972, **56**, 2257; (c) P. C. Hariharan and J. A. Pople, *Theor. Chem. Acc*., 1973, **28**, 213.
18. M. Huelsen, A. Weigand, M. Dolg, *Theor. Chem. Acc.* 2009, **122**, 23.
19. S. Grimme, S. Ehrlich, L. Goerigk, J. Comp. Chem., 2011, 32, 1456
20. Gaussian 09, Revision D.01, M. J. Frisch, G. W. Trucks, H. B. Schlegel, G. E. Scuseria, M. A. Robb, J. R. Cheeseman, G. Scalmani, V. Barone, G. A. Petersson, H. Nakatsuji, X. Li, M. Caricato, A. Marenich, J. Bloino, B. G. Janesko, R. Gomperts, B. Mennucci, H. P. Hratchian, J. V. Ortiz, A. F. Izmaylov, J. L. Sonnenberg, D. Williams-Young, F. Ding, F. Lipparini, F. Egidi, J. Goings, B. Peng, A. Petrone, T. Henderson, D. Ranasinghe, V. G. Zakrzewski, J. Gao, N. Rega, G. Zheng, W. Liang, M. Hada, M. Ehara, K. Toyota, R. Fukuda, J. Hasegawa, M. Ishida, T. Nakajima, Y. Honda, O. Kitao, H. Nakai, T. Vreven, K. Throssell, J. A. Montgomery, Jr., J. E. Peralta, F. Ogliaro, M. Bearpark, J. J. Heyd, E. Brothers, K. N. Kudin, V. N. Staroverov, T. Keith, R. Kobayashi, J. Normand, K. Raghavachari, A. Rendell, J. C. Burant, S. S. Iyengar, J. Tomasi, M. Cossi, J. M. Millam, M. Klene, C. Adamo, R. Cammi, J. W. Ochterski, R. L. Martin, K. Morokuma, O. Farkas, J. B. Foresman, and D. J. Fox, Gaussian, Inc., Wallingford CT, 2016.
21. NBO 6.0. E. D. Glendening, J. K. Badenhoop, A. E. Reed, J. E. Carpenter, J. A. Bohmann, C. M. Morales, C. R. Landis, F. Weinhold (Theoretical Chemistry Institute, University of Wisconsin, Madison, WI, 2013)
22. K. L. Mears, C. R. Stennett, J. C. Fettinger, P. Vasko and P. P. Power, *Angew. Chem. Int. Ed*, 2022, **61**, e202201318.
